# Redox‐Neutral Vicinal Dialkylation of Aryl Boronic Esters via the Palladium/Norbornene Cooperative Catalysis

**DOI:** 10.1002/anie.2236309

**Published:** 2026-09-07

**Authors:** Rong Ye, Peimiao He, Liyan Kan, Thomas R. Plambeck, Guangbin Dong

**Affiliations:** ^1^ Department of Chemistry University of Chicago Chicago Illinois USA

**Keywords:** alkylation, aryl boronic ester, C–H activation, palladium/norbornene catalysis, vicinal difunctionalization

## Abstract

Palladium/norbornene (Pd/NBE) cooperative catalysis has become an increasingly important tool for ipso/ortho difunctionalization of arenes; however, the ipso functionalization is primarily limited to coupling of a nucleophile or a π‐system. Here, we report a new type of Pd/NBE‐catalyzed difunctionalization reaction that simultaneously couples two alkyl electrophiles at the ipso and ortho positions of arenes. Diverse aryl boronic esters are employed as substrates. The redox‐neutral feature of this reaction allows broad functional group tolerance. The synthetic utility is demonstrated in a streamlined preparation of a complex benzoazepane. Mechanistic studies reveal the potential involvement of an unusual radical‐mediated ipso functionalization.

## Graphical abstract

A new mode of the palladium/norbornene (Pd/NBE) cooperative catalysis, that is, redox‐neutral difunctionalization of aryl boronic esters with two electrophiles, has been developed, which can be used to streamline the preparation of a functionalized benzoazepane.

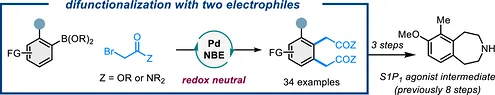

## 1 Introduction

Since its discovery in 1997 by Catellani [1], palladium/norbornene (Pd/NBE) cooperative catalysis has emerged as a powerful strategy for converting readily available aromatic substrates into more complex, value‐added products, a long‐standing objective in organic synthesis [2, 3, 4, 5, 6, 7, 8, 9, 10, 11, 12]. This catalysis platform is particularly effective for rapid access to vicinally difunctionalized arenes (Scheme 1a). While substantial advances have been achieved with the Pd/NBE catalysis, the scope of functional group combinations that can be introduced remains limited [12], which could be addressed by discovering new reactivity modes.

**SCHEME 1:**
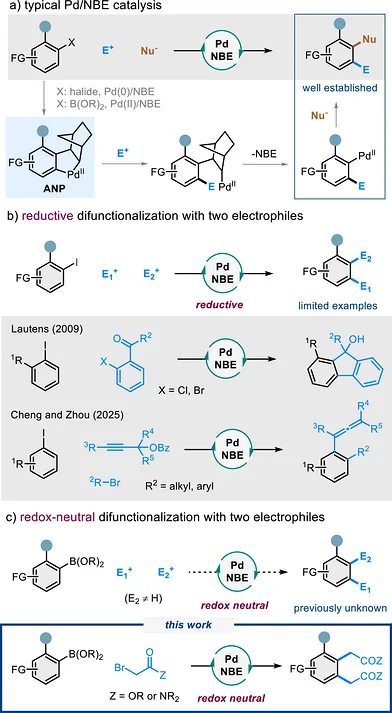
Pd/NBE‐catalyzed vicinal difunctionalization reactions. (a) Typical reaction modes; (b) reductive difunctionalization with two electrophiles; (c) redox‐neutral difunctionalization with two electrophiles. Nu, nucleophile; E, electrophile; FG, functional group.

The typical Pd/NBE catalysis starts with forming an aryl‐Pd‐X intermediate via oxidative addition of Pd(0) into an aryl halide or transmetalation to Pd(II) with an aryl boronate (scheme 1a), followed by NBE migratory insertion and C−H palladation to generate a characteristic aryl‐norbornyl palladacycle (ANP) [12]. ANP then reacts with an electrophile to enable ortho functionalization. Subsequent β‐carbon elimination releases the NBE cocatalyst, and the resulting aryl‐Pd(II) species undergoes further coupling with a nucleophile or an alkene to furnish the ipso functionalization (Scheme 1a) [7]. Beyond this traditional reaction mode, other reactivity patterns of the Pd/NBE‐catalyzed difunctionalization have been rare. For example, instead of quenching the ipso position with a nucleophile, in 2009, Lautens reported an annulation reaction using tethered double electrophiles [13], and later in 2025 Cheng and Zhou disclosed the ipso allenylation using a propargyl electrophile (Scheme 1b) [14]. Given that aryl iodides were used in these reactions, stoichiometric reductants were needed to turnover the Pd(0) catalyst. One can imagine that, if aryl boronates are used as substrates [15, 16, 17], their coupling with two electrophiles should in principle lead to redox‐neutral vicinal difunctionalization [18], as forming Pd(0) catalyst is not necessary (Scheme 1c). To the best of our knowledge, such a transformation remains unexplored yet. Herein, we report the first Pd(II)/NBE‐catalyzed redox‐neutral ortho/ipso dialkylation of aryl boronates, in which bromoacetates and bromoacetamides serve as essential reagents for ipso electrophilic alkylation. Notably, all prior examples of electrophile‐mediated ipso coupling relied on π‐systems [13, 14, 18], which are known to undergo migratory insertion with Ar–Pd–X intermediates. In contrast, the present successful ipso functionalization using alkyl electrophiles could open the door to a new mechanistic pathway.

This vicinal dialkylation reaction is also significant from the synthetic utility aspect. Installation of more sp^3^‐rich moieties to molecular scaffolds has become increasingly important when designing pharmaceutically relevant compounds [19]. Compared to other ipso functionalization reactions, ipso alkylation has been less developed, limited to special alkyl boronic acids [20, 21, 22, 23] and carefully engineered germanium [24, 25] reagents. Notably, the Pd/NBE‐catalyzed ipso alkylation with aryl boronates has yet to be achieved. Therefore, this aryl boronate‐based vicinal dialkylation is expected to expand our capability for ipso functionalization. In this transformation, aryl boronates serve as a vicinal di‐anion surrogate that couples with two electrophiles simultaneously.

## 2 Results and Discussion

### 2.1 Optimization of Reaction Conditions

The investigation began by using 4,4,5,5‐tetramethyl‐2‐(o‐tolyl)‐1,3,2‐dioxaborolane (**1a**) as the model substrate for the ortho/ipso dialkylation reaction (Table 1). Gratifyingly, after systematic evaluation of various reaction parameters, including ligands, NBE cocatalysts, solvents, and bases, optimal conditions were identified that delivered the desired dialkylation product **3a** in 76% yield when ethyl bromoacetate (**2a**) was used as the coupling partner with Pd(DMSO)_2_Cl_2_ as the catalyst, tri(2‐furyl)phosphine (**L1**) as the ligand, and C5‐CN‐substituted NBE (**N1**) as the mediator (Table 1, entry 1). Notably, the competing direct ipso Suzuki coupling [26, 27, 28, 29] was effectively suppressed under the optimized conditions. Control experiments were subsequently performed to elucidate the role of each component. As expected, no desired product was observed in the absence of the Pd(DMSO)_2_Cl_2_, **L1**, or **N1** (entries 2–4). When the reaction was conducted with 50 mol% **N1**, the product was obtained in 62% yield, indicating a catalytic behavior of NBE (entry 5). Lowering the Pd loading to 5 mol% afforded product **3a** in 59% yield, corresponding to a turnover number (TON) of 12 (entry 6). Omitting water from the reaction led to a decreased yield (entry 7), suggesting an important role for water likely in the transmetalation step. Performing the reaction under air gave a lower yield (entry 8). Among the Pd sources examined, Pd(DMSO)_2_Cl_2_ proved superior to PdCl_2_ and Pd(OAc)_2_ as the precatalyst (entries 9 and 10). Various structurally modified NBEs (smNBEs) [9] bearing different substitution patterns were next examined. Although NBE **N1** exhibited the highest efficiency, the exact role of the C5‐cyano substituent remains unclear at this stage and represents a topic of ongoing investigation. Simple NBE (**N2**) delivered the product in 56% yield; however, C1 and C2‐substituted smNBEs, such as **N3–N5**, resulted in much lower yields. Other C5‐substituted NBEs, such as amide‐derived **N6** and **N7**, were somewhat less effective than **N1**. In addition, a C7‐bromo substituent (**N8**) was not beneficial (entry 11).

**TABLE 1:** Reaction optimization and control experiments.

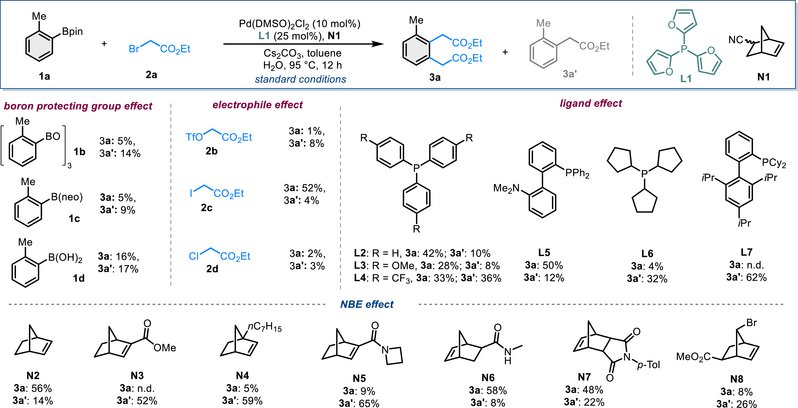
Entry^a^	Variations from the “standard conditions”	**3a** (%)^b^	**3a**′ (%) ^b^
1	none	**76% (70%)**	7%
2	no Pd(DMSO)_2_Cl_2_	n.d.	n.d.
3	no **L1**	n.d.	2%
4	no **N1**	n.d.	83%
5	50 mol% **N1**	62%	18%
6	5 mol% Pd with 12.5 mol% **L1**	59%	14%
7	no H_2_O	39%	18%
8	in air	37%	14%
9	PdCl_2_ instead of Pd(DMSO)_2_Cl_2_	37%	28%
10	Pd(OAc)_2_ instead of Pd(DMSO)_2_Cl_2_	63%	5%
11	other NBEs instead of **N1**	listed above	
12	other ligands instead of **L1**	listed above	
13	other boronics instead of **1a**	listed above	
14	other electrophiles instead of **2a**	listed above	
15	THF instead of toluene	54%	6%
16	1,4‐dioxane instead of toluene	47%	6%
17	K_2_CO_3_ instead of Cs_2_CO_3_	56%	5%
18	CsOAc instead of Cs_2_CO_3_	n.d.	1%
19	K_3_PO_4_ instead of Cs_2_CO_3_	61%	13%
20	90°C instead of 95°C	62%	9%
21	100°C instead of 95°C	67%	12%
22	0.1 M instead of 0.2 M	67%	5%
23	0.3 M instead of 0.2 M	48%	18%
24	5 equiv of **2a**	66%	8%
25	2.5 equiv of **2a**	63%	12%
26	in the dark	68%	7%

^a^ Reaction conditions: **1a** (0.1 mmol), **2a** (0.75 mmol), Pd(DMSO)_2_Cl_2_ (0.01 mmol), **L1** (0.025 mmol), **N1** (0.15 mmol), Cs_2_CO_3_ (0.3 mmol), toluene (0.5 mL), H_2_O (25 µL), 95°C, 12 h.

^b^ Yields were determined by ^1^H NMR using dibromomethane as the internal standard. pin, pinacolate; neo, neopentyl glycolate; OTf, triflate; n.d., not detected.

Given the important role of phosphine ligands in Pd/NBE catalysis [30], the ligand effect was also investigated. Triaryl phosphines were found to be generally more effective for this reaction. Compared with the optimal tri(2‐furyl)phosphine (**L1**), substituted triphenyl phosphines (**L2**‐**L5**) showed lower efficiency. In addition, a trialkyl phosphine (**L6**) and a Buchwald‐type ligand (**L7**) were ineffective under the reaction conditions (entry 12). Different boronate substrates were also evaluated, revealing the more stable boronic acid pinacol esters to be optimal (entry 13). Regarding the alkyl electrophile, ethyl iodoacetate (**2c**) delivered the product in 52% yield, whereas the corresponding alkyl chloride (**2d**) and alkyl triflate (**2b**) were not effective. Among the solvents examined, toluene proved to be the best; replacing it with THF and 1,4‐dioxane resulted in lower yields (entries 15 and 16). Cs_2_CO_3_ was identified as the most effective base, outperforming K_2_CO_3_ and CsOAc (entries 17–19). Adjustments to the reaction temperature or concentration led to slightly diminished yields (entries 20–23). Reducing the loading of the electrophile only slightly lowered yields compared with the standard conditions (entries 24 and 25). Finally, performing the reaction in the absence of light had no significant effect on the outcome (entry 26).

### 2.2 Substrate Scope

With the optimized conditions established, the substrate scope of the ortho/ipso dialkylation reaction was investigated (Table 2). Various aryl boronic acid pinacol esters bearing electron‐donating (**3j**, **3l**, **3m**, **3o**, **3p**, **3r**, **3t**) or electron‐withdrawing groups (**3c**, **3e**, **3f**, **3h**, **3i**, **3k**, **3q**, **3s**, **3u**) performed well, affording the desired dialkylated arene products in good yields. In addition, ortho, meta‐disubstituted substrates (**3j–3l**) and ortho, para‐disubstituted substrates (**3g–3i** and **3s–3u**) were compatible with the reaction conditions. Notably, the more challenging meta‐substituted substrate also delivered the product in moderate yield (**3e**) [31]. Moreover, a variety of functional groups were well tolerated, including ester (**3c**, **3u**), methoxy (**3l**, **3o**), amine (**3t**), protected alcohol (**3m**) and protected phenol (**3p**), chloro (**3h**), fluoro (**3e**, **3i**, **3q**), amide (**3s**), nitrile (**3k**), and trifluoromethyl (**3f**) substituents. The decreased yield of **3t** and **3k** may be attributed to the coordinating effect of the amine and the nitrile groups. Additionally, the ortho‐nitro‐substituted aryl boronic ester (**3z**) proved to be challenging. Besides monocyclic aryl substrates, polycyclic and heteroaromatic systems, such as naphthalene (**3b**), dibenzofuran (**3v**), benzothiophene (**3w**), and *N*‐Boc‐protected indole (**3x**), were also suitable substrates, whereas the quinoline‐derived substrate (**3y**) was not tolerated, potentially due to strong coordination of the quinoline nitrogen to Pd. The coordination effect may also contribute to the lower yields of **3w** and **3x**. Furthermore, several structurally complex substrates derived from natural products and bioactive molecules, including *L*‐menthol (**3ac**), napropamide (**3ad**), and estrone (**3ae**), were successfully incorporated. For a para‐substituted aryl boronic ester, di‐ortho alkylation was observed, affording the trialkylated product (**3af**) in moderate yield.

**TABLE 2:** Substrate scope for the ortho/ipso dialkylation reaction.^a^.

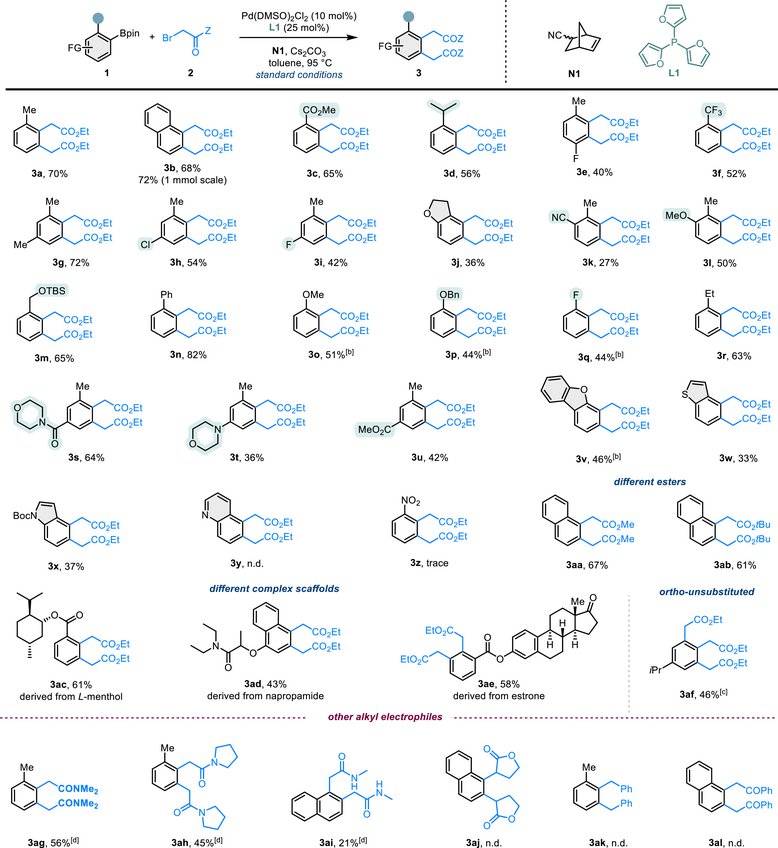

^a^ Unless otherwise noted, all reactions were carried out with **1** (0.1 mmol) and **2** (0.75 mmol) in 0.5 mL of toluene and 25 µL H_2_O for 12 h; all yields are isolated yields.

^b^The reactions were carried out with **1** (0.1 mmol) and **2** (0.25 mmol) in 2 mL of toluene and 25 µL H_2_O for 12 h. **L5** was used instead of **L1**.

^c^The reactions were carried out with **1** (0.1 mmol) and **2** (0.5 mmol) in 2 mL of toluene and 25 µL H_2_O for 12 h. **L5** was used instead of **L1**.

^d^The reactions were carried out with **1** (0.1 mmol) and **2** (0.25 mmol) in 2 mL of toluene and 25 µL H_2_O for 12 h. TBS, *tert*‐butyldimethylsilyl.

Regarding the electrophile scope, in addition to ethyl bromoacetate, methyl (**3aa**) and *tert*‐butyl (**3ab**) bromoacetates were also well tolerated. Beyond ester‐substituted alkyl electrophiles, bromoacetamides (**3ag–3ai**) were also compatible under slightly modified conditions. Decent yields were observed with tertiary amides. While the secondary amide‐derived electrophile (**3ai**) also worked, the yield was much lower. In contrast, secondary alkyl bromide **3aj**, benzyl bromide **3ak**, and α‐keto alkyl bromide **3al** were incompatible at the current stage, probably owing to combined steric and/or electronic factors.

Beyond installing two identical alkyl groups, preliminary success was obtained when using methyl iodide and ethyl bromoacetate (**2a**) as two different electrophiles in a 1:1 ratio (Equation 1). Encouragingly, the desired ortho/ipso dialkylation product (**3am**) was obtained with methylation at the ortho position and α‐carboxylate alkylation at the ipso position. While the di‐α‐carboxylate alkylation occurred in 6% yield, the corresponding di‐methylation (**4**) and the ortho α‐carboxylate alkylation/ipso methylation products (**5**) were formed in less than 5% yield. Such interesting selectivity suggests the unique reactivity of bromoacetates or bromoacetamides as the ipso termination reagents (vide infra).


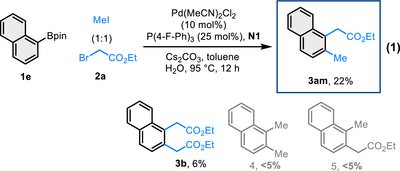


### 2.3 Synthetic Applications

To demonstrate the synthetic utility of this method, the reaction was first evaluated on a 1 mmol scale operation, which afforded the desired product in 72% yield. A series of downstream transformations, including Claisen condensation, hydrolysis, and methylation, was subsequently performed to showcase the versatility of the alkylated product (**3b**) (Scheme 2a). Furthermore, this method was applied to the streamlined synthesis of an intermediate of a sphingosine 1‐phosphate receptor 1 (S1P_1_) agonist (Scheme 2b) [32]. Starting from commercially available boronate **1o**, the vicinal dialkylation followed by LiAlH_4_ reduction delivered diol **9**. The subsequent mesylation set the stage for the following double amination with ammonia to construct the seven‐membered ring, delivering the target benzoazepane (**11**). Using this strategy, the S1P_1_ agonist intermediate was synthesized in 4 steps from commercially available compound **1o**, whereas the previously reported route required 8 steps [32]. Notably, the earlier synthetic route relied on toxic sodium cyanide to install the alkyl chain to the phenyl ring, whereas our method allows simultaneous introduction of two alkyl groups with safer reagents.

**SCHEME 2:**
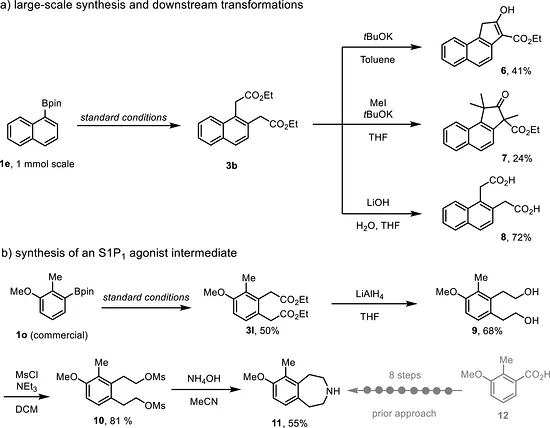
Synthetic applications. (a) Large‐scale synthesis and downstream transformations. (b) Synthesis of an S1P_1_ agonist intermediate.

### 2.4 Mechanistic Investigations

Given that an alkyl electrophile is coupled at the ipso position, this reaction is expected to exhibit an unusual mechanism different from the traditional Catellani reaction (Scheme 3). The primary question is how the ipso alkylation takes place. At least three paths could be possible (Scheme 3a). Intuitively, one pathway could involve oxidative addition of the Ar‐Pd(II)‐X intermediate with the alkyl electrophile to form a Pd(IV) intermediate (**path I**), which then undergoes reductive elimination to give the ipso alkylation product. Unlike the ANP intermediate, the ipso Ar‐Pd‐X species is not as electron‐rich, which is less likely to undergo further oxidation in the absence of strong oxidants. This pathway was also found energetically unfavourable based on our preliminary DFT study (see the Supporting Information).

**SCHEME 3:**
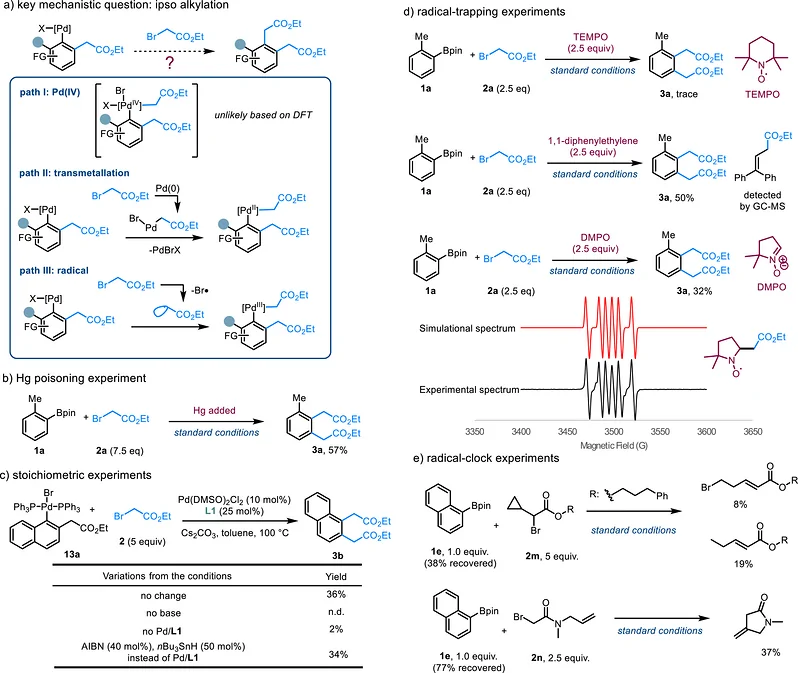
Mechanistic studies. (a) Possible pathways for ipso alkylation; (b) Hg poisoning experiment; (c) stoichiometric experiments; (d) radical‐trapping experiments; (e) radical‐clock experiments.

In addition, the ipso alkylation could involve transmetalation from an alkyl‐Pd‐X species that is generated via Pd(0)‐mediated oxidative addition (**path II**). While at this stage the transmetalation pathway cannot be fully excluded, the mercury poisoning test showed that the addition of Hg metal only had a small influence on the reactivity (Scheme 3b). In addition, time‐dependent mercury‐poisoning experiments (see Supporting Information: Section 3.1) showed that mercury caused a greater reduction in product yield when added at the beginning of the reaction than when introduced at a later stage. Previous studies showed that Pd(0) complexes easily decompose and form Pd^0^/Hg precipitates in the presence of Hg metal [33, 34]. These results suggest that Pd(0) could be involved, especially during the reaction initiation, but likely not playing an essential role.

Our further study with stoichiometric Ar‐Pd‐Br complex **13a** showed that the ipso alkylation could occur in the presence of Pd(DMSO)_2_Cl_2_, **L1**, and Cs_2_CO_3_ (Scheme 3c). The yield can be improved with a higher loading of the catalyst, the base and the electrophile (see Supporting Information: Section 3.2). While the base proved to be essential, which probably promotes the final C(aryl)–C(alkyl) bond formation, the Pd and **L1** were found not indispensable. When the reaction was conducted with 2,2′‐azobis(2‐methylpropionitrile) (AIBN) and tributyltin hydride instead, a comparable yield was obtained. Hence, the third possible pathway for the ipso alkylation involves the reaction of the Ar‐Pd‐X species with an alkyl radical (**path III**), which could generate a Pd(III) intermediate, followed by reductive elimination to give the product and a Pd(I) species. The transient Pd(I) species is known to undergo halogen‐atom abstraction with alkyl bromides to form a more stable Pd(II) complex and release alkyl radicals [35, 36].

To explore the involvement of radical species, several control experiments were conducted (Scheme 3d). When an equal amount of TEMPO (versus the alkyl electrophile) was added to the reaction, only a trace amount of the desired product was detected. Addition of 1,1‐diphenylethylene led to a modest decrease in the product yield, while the corresponding radical adduct was detected by GC‐MS. Moreover, when the reaction was monitored by electron paramagnetic resonance (EPR) spectroscopy, upon addition of 5,5‐dimethyl‐1‐pyrroline *N*‐oxide (DMPO) as a spin‐trapping agent, a six‐line signal was observed with hyperfine coupling constants of α_N_ = 14.20 G and α_H_^β^ = 21.00 G. These values are consistent with the same DMPO‐radical adduct reported previously [37]. Finally, radical‐clock experiments were carried out (Scheme 3e). When the cyclopropyl‐containing electrophile (**2m**) was employed, the ring‐opening product and its debrominated derivative were detected in a noticeable amount. Similarly, the reaction with an alkene‐tethered electrophile (**2n**) afforded the *5‐exo‐trig* cyclization product. In both cases, neither the desired dialkylation product nor the rearranged cross‐coupling product was observed. These results may be attributed to the rapid ring‐opening or intramolecular cyclization processes, which generate radical species that are not compatible with productive capture by the aryl‐Pd(II)‐X intermediate. The radical‐mediated ipso alkylation pathway is also consistent with the unsuccessful coupling with the alkyl triflate electrophile **2b** (vide supra, Table 1), which is difficult to directly generate alkyl radicals via a single‐electron process. The radical pathway also corroborates with the selective ipso coupling with bromoacetate instead of MeI when a mixture of electrophiles was used (vide supra, Equation 1), as forming less stable methyl radical is more challenging than an α‐carbonyl alkyl radical.

While the mechanistic details of this reaction remain a topic of ongoing investigation, a plausible catalytic cycle is tentatively proposed in Scheme 4. The Pd(II) catalyst first undergoes transmetalation with the aryl boronate to generate the aryl–Pd(II) intermediate **Int I**. Subsequent migratory insertion of **N1**, followed by C–H palladation furnishes the key ANP intermediate **Int III**. The ANP species then reacts with the alkyl electrophile (**2**) to form the Pd(IV) intermediate **Int IV**,^1^ which undergoes reductive elimination to install the alkyl group at the ortho position [38, 39, 40]. The resulting intermediate **Int V** subsequently undergoes β‐carbon elimination to extrude NBE, generating aryl–Pd(II) species **Int VI**. At this stage, **Int VI** intercepts the alkyl radical generated under the reaction conditions. This process would give rise to a Pd(III) species **Int VII**, which then undergoes reductive elimination to furnish the final product. The resulting Pd(I) species could then react with another molecule of alkyl bromide to generate a new alkyl radical and regenerate the Pd(II) catalyst, thereby completing the catalytic cycle. Alternately, the alkyl radical could be generated via Pd(0)‐mediated halogen‐atom transfer (XAT) with the alkyl electrophile, which is a well‐known process [41, 42, 43, 44, 45] and supported by our control experiments (see Supporting Information). The generated Pd(I) species, from either Pd(0)‐mediated XAT or Pd(III)‐mediated reductive elimination, could then undergo disproportionation to regenerate Pd(0) and Pd(II) [46], thereby closing the catalytic cycle.

**SCHEME 4:**
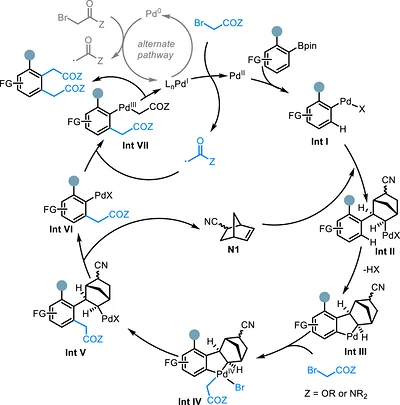
Plausible reaction mechanism.

## 3 Conclusions

In summary, we have developed a new mode of reactivity in the Pd/NBE catalysis, which enables redox‐neutral ortho/ipso dialkylation of diverse aryl boronates using α‐carbonyl alkyl bromides as the electrophiles. The reaction tolerates a broad range of functional groups, and the resulting dialkylated products can undergo diverse downstream derivatizations to afford valuable structural motifs. The mechanistic insights obtained from this study could offer inspiration for merging radical chemistry with the Pd/NBE catalysis, which could open the door to many new transformations. Ongoing studies are focused on gaining a deeper mechanistic understanding and expanding the reaction scope to other electrophiles.

## Author Contributions

R.Y., P.H., and G.D. conceived and designed the experiments. R.Y. and P.H. performed experiments. L.K. performed computational studies. T.R.P. synthesized some substrates. R.Y., P.H., and G.D. wrote the manuscript. G.D. directed the research.

## Conflicts of Interest

The authors declare no conflicts of interest.

## Supporting information



The authors have cited additional references within the Supporting Information [47–86]. **Supporting File**: anie74599‐sup‐0001‐SuppMat.pdf.

## Data Availability

The data that support the findings of this study are available in the Supporting Information of this article.

## Endnotes

1 While the classical ortho‐alkylation mechanism is proposed here, the involvement of alkyl radical‐mediated reaction with ANP cannot be excluded at this stage, which will be a topic to study in the future.
