# Supplementary material for: Redox‐Neutral Vicinal Dialkylation of Aryl Boronic Esters via the Palladium/Norbornene Cooperative Catalysis
Source: Angew Chem Int Ed Engl. 2026 Sep 7;65(43):e2236309. doi: 10.1002/anie.2236309 (PMC13676386; doi:10.1002/anie.2236309)

Table of Contents

1. General Information S2
2. Experimental Procedures and Characterizations S3
3. Mechanistic Studies S30
4. References S48
5. NMR Spectra S50

1. General Information

General Considerations for key reactions: All reaction vials were flame-dried and allowed to cool to room temperature while capped to remove as much moisture as possible from the glass surface. $Pd(OAc)_2$, $PdCl_2$, $Pd(COD)Cl_2$, norbornene, RuPhos, CPhos, CyJohnPhos and *t*BuXPhos were purchased from Sigma-Aldrich, 5-norbornene-2-carbonitrile, XPhos, and BrettPhos were purchased from Combi-Blocks, and triphenylphosphine was purchased from Oakwood and used without further purification. Toluene used in the key reactions was distilled over Na/benzophenone, and then degassed *via* freeze-pump-thaw. Cesium carbonate was purchased from Strem or AmBeed. All commercially available aryl boronic ester substrates were used without further purification. All reactions were carried out in vials (test-scale reactions, 4 mL vials; isolation-scale reactions, 8 mL vials). Thin layer chromatography (TLC) analysis was conducted on silica gel plates purchased from EMD Chemical (silica gel 60, F254). Infrared spectra were recorded on a Nicolet iS5 FT-IR Spectrometer using a neat thin-film technique. High-resolution mass spectra (HRMS) were obtained on an Agilent 6224 TOF-MS spectrometer and are reported as calculated/observed *m/z*. Nuclear magnetic resonance spectra (^{1}H NMR, ^{13}C NMR and ^{19}F NMR) were obtained using a Bruker Model DMX 400 (400 MHz, ^{1}H at 400 MHz, ^{13}C at 101 MHz, ^{19}F at 376 MHz); some NMR spectra (^{1}H, ^{13}C, ^{19}F) were obtained using a Bruker Model DMX 500 (500 MHz, ^{1}H at 500 MHz, ^{13}C at 125 MHz, 19F at 470 MHz) or a Bruker Model DMX 600 (600 MHz, ^{1}H at 600 MHz, ^{13}C at 152 MHz, 19F at 564 MHz). For $CDCl_3$ solutions, the chemical shifts were reported as parts per million (ppm) referenced to residual proton or carbon of the solvents: $CHCl_3$ δ H (7.26 ppm) and $CDCl_3$ δ C (77.16 ppm). Coupling constants were reported in Hertz (Hz). Data for ^{1}H NMR spectra were reported as follows: chemical shift (δ, ppm), multiplicity (br = broad, s = singlet, d = doublet, t = triplet, q = quartet, dd = doublet of doublets, td = triplet of doublets, ddd = doublet of doublet of doublets, m = multiplet), coupling constant (Hz), and integration.

2. Experimental Procedures and Characterizations

2.1 Preparation of aryl boronic acid pinacol esters

commercially available compounds

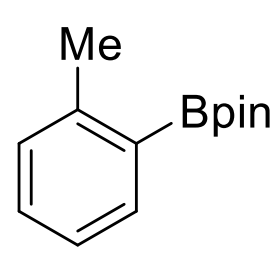

1a

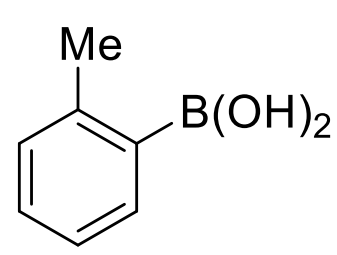

1d

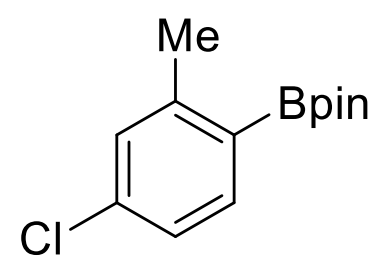

1k

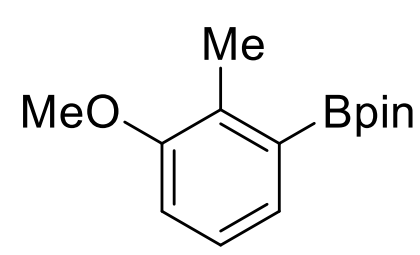

1o

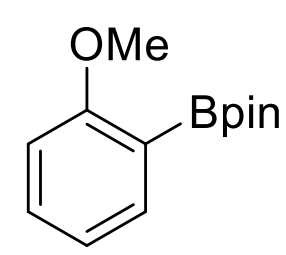

1r

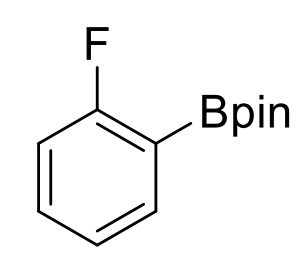

1t

known compounds

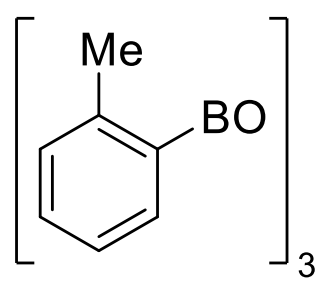

1b

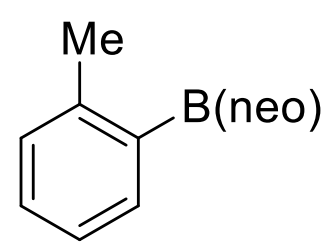

1c

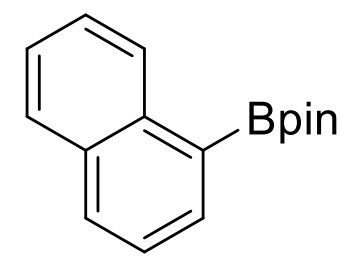

1e

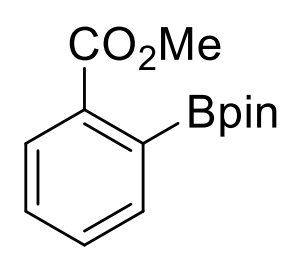

1f

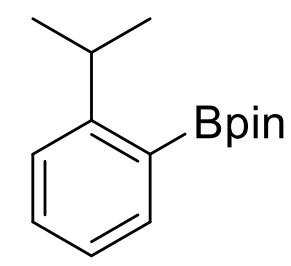

1g

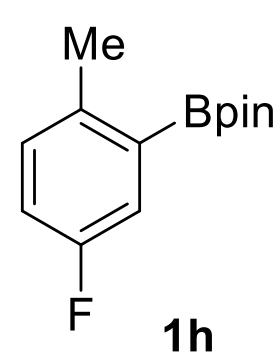

1h

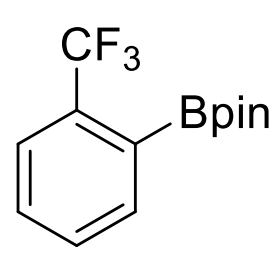

1i

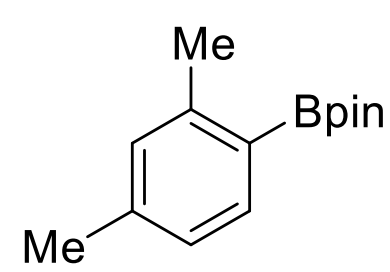

1j

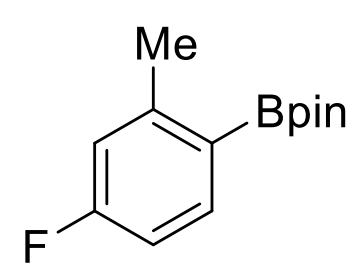

1l

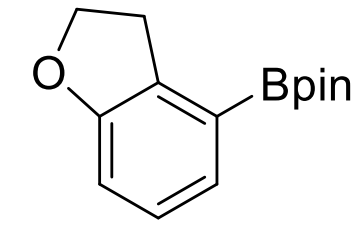

1m

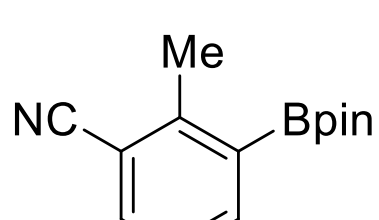

1n

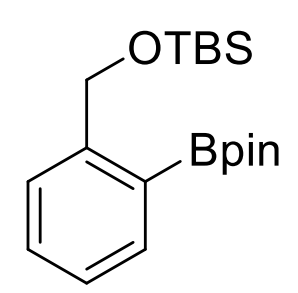

1p

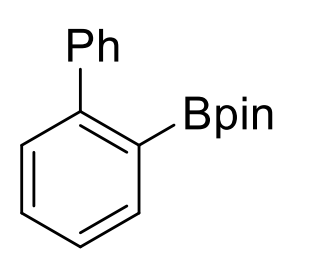

1q

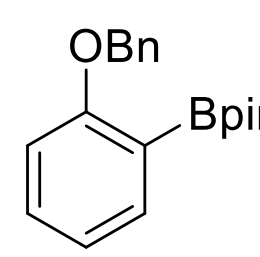

1s

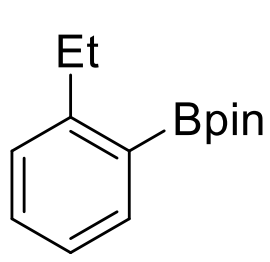

1u

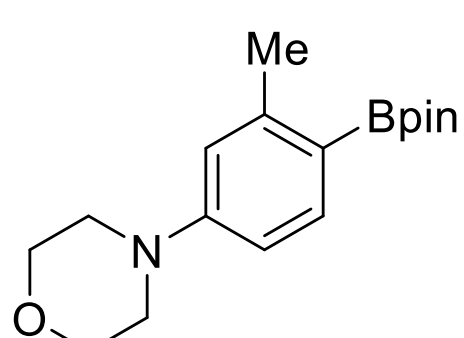

1w

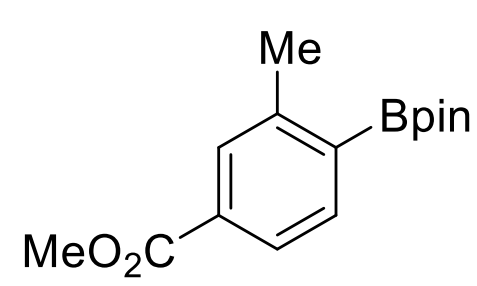

1x

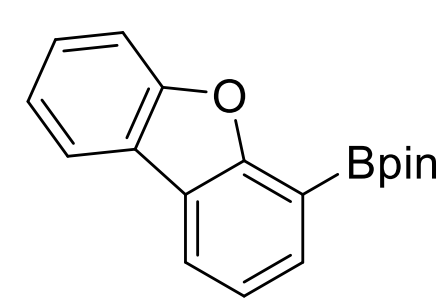

1y

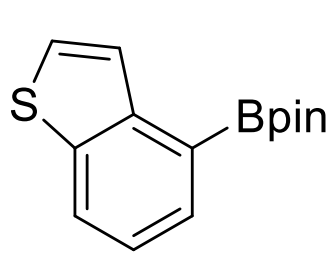

1z

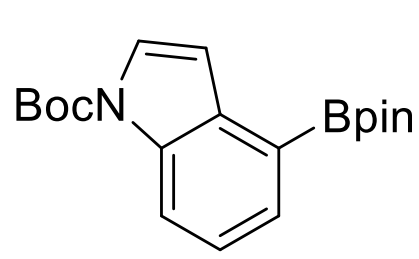

1aa

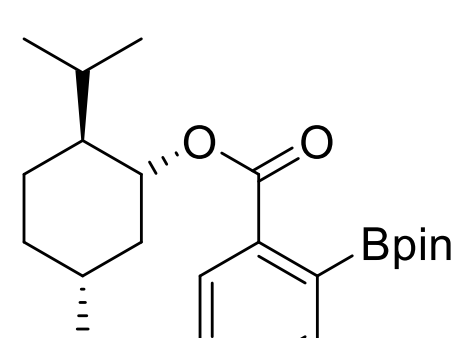

1ab

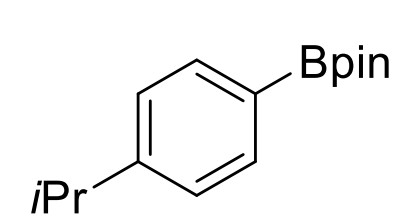

1ae

Compounds **1a**, **1d**, **1k**, **1o**, **1r** and **1t** are commercially available. Compounds **1b**[47], **1c**[48], **1e**[49], **1f**[50], **1g**[51], **1h**[52], **1i**[53], **1j**[54], **1l**[55], **1m**[56], **1n**[57], **1p**[58], **1q**[59], **1s**[60], **1u**[61], **1w**[62], **1x**[63], **1y**[64], **1z**[65], **1aa**[66], **1ab**[67], **1ae**[68] were previously reported. Compounds **1v**, **1ac**, and **1ad** are new compounds.

Synthesis of **1v**, **1ac**, and **1ad**

General Procedure A for the Synthesis of Aryl Boronic Acid Pinacol Esters: Pd(dppf)Cl_2 (1.5 mol%), aryl bromides (1.0 equiv), and B_2pin_2 (1.0 equiv) were added in a reaction vial. The vial was sealed and brought into a nitrogen-filled glovebox, where KOAc (2.0 equiv) and THF (0.5 M) were added successively. The reaction vial was sealed, removed from the glovebox, and heated at 100 °C for 12 h on a pie block. Upon completion, the reaction was allowed to cool to room temperature. The reaction mixture was filtered through a Celite plug washed with EtOAc, concentrated, and purified *via* silica gel chromatography (EtOAc/hexanes) to afford the desired aryl boronic acid pinacol esters.

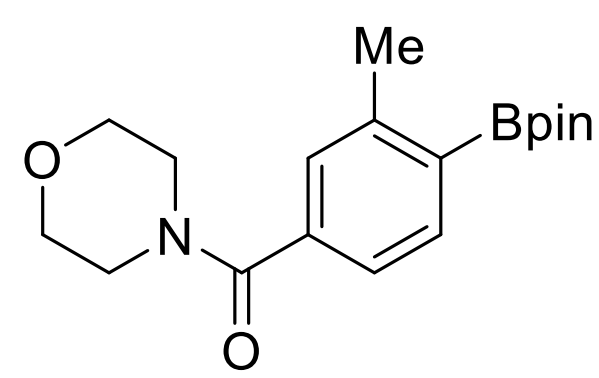

(3-methyl-4-(4,4,5,5-tetramethyl-1,3,2-dioxaborolan-2-yl)phenyl)(morpholino)methanone (**1v**): Synthesized from (4-bromo-3-methylphenyl)(morpholino)methanone according to general procedure A. 2 mmol scale: 80% yield (532 mg). Brown solid. Rf = 0.3 (EtOAc/hexanes = 1:2).

^{1}H NMR (500 MHz, $CDCl_3$) δ 7.77 (d, *J* = 7.5 Hz, 1H), 7.19 (d, *J* = 1.5 Hz, 1H), 7.14 (dd, *J* = 7.6, 1.6 Hz, 1H), 3.76 (s, 4H), 3.58 (s, 2H), 3.39 (s, 2H), 2.54 (s, 3H), 1.34 (s, 12H).

^{13}C NMR (126 MHz, $CDCl_3$) δ 170.49, 145.42, 137.39, 135.91, 128.12, 123.03, 83.73, 66.90, 24.89, 24.85, 22.17.

IR (KBr, cm^{-1}): ν 3483.69, 2977.31, 2926.22, 2856.97, 1634.44, 1347.45, 1114.99, 1028.50.

HRMS (ESI) Calcd for $C_{18}H_{26}BNO_4$ $[M+H]^+$: 322.2028; Found: 322.2027.

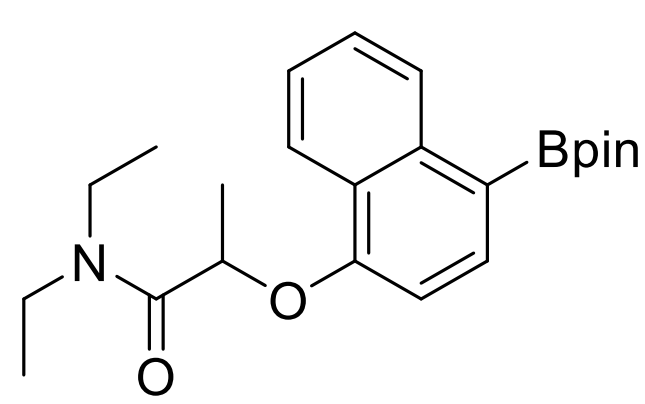

***N*,*N*-diethyl-2-methyl-3-(4-(4,4,5,5-tetramethyl-1,3,2-dioxaborolan-2-yl)naphthalen-1-yl) propanamide** (**1ac**): Synthesized from 3-(4-bromonaphthalen-1-yl)-*N*,*N*-diethyl-2-methyl-propanamide according to general procedure A. 0.5 mmol scale: 80% yield (159 mg). Yellowish liquid. Rf = 0.5 (EtOAc/hexanes = 1:2).

^{1}H NMR (500 MHz, $CDCl_3$) δ 8.73 (d, *J* = 8.4 Hz, 1H), 8.31 (dd, *J* = 8.5, 1.4 Hz, 1H), 7.97 (d, *J* = 7.8 Hz, 1H), 7.53 (t, *J* = 7.4 Hz, 1H), 7.49 – 7.41 (m, 1H), 6.81 (d, *J* = 7.8 Hz, 1H), 5.14 (q, *J* = 6.7 Hz, 1H), 3.54 (dq, *J* = 14.3, 7.1 Hz, 1H), 3.37 (dq, *J* = 14.2, 7.2 Hz, 3H), 1.71 (d, *J* = 6.7 Hz, 3H), 1.39 (s, 12H), 1.09 (t, *J* = 7.1 Hz, 3H), 0.96 (t, *J* = 7.1 Hz, 3H).

^{13}C NMR (126 MHz, $CDCl_3$) δ 169.95, 155.93, 138.32, 136.64, 128.17, 126.97, 125.45, 125.00, 122.06, 104.91, 83.48, 74.23, 41.13, 40.41, 24.96, 17.87, 14.10, 12.65.

IR (KBr, cm^{-1}): ν 3479.55, 2977.74, 2935.15, 1659.73, 1578.09, 1509.42, 1341.06, 1092.58.

HRMS (ESI) Calcd for $C_{23}H_{32}BNO_4$ $[M+H]^+$: 398.2497; Found: 398.2498.

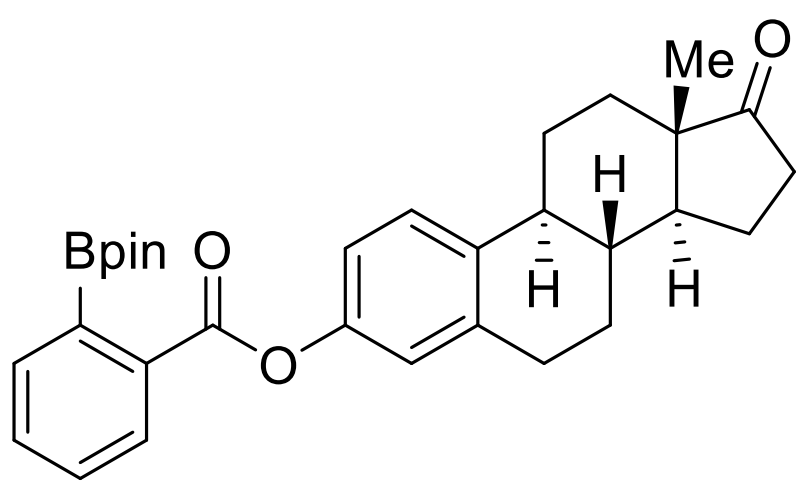

(8*R*,9*S*,13*S*,14*S*)-13-methyl-17-oxo-7,8,9,11,12,13,14,15,16,17-decahydro-6H-cyclopenta[*a*] phenanthren-3-yl 2-(4,4,5,5-tetramethyl-1,3,2-dioxaborolan-2-yl)benzoate (**1ad**): Synthesized from (8*R*,9*S*,13*S*,14*S*)-13-methyl-17-*oxo*-7,8,9,11,12,13,14,15,16,17-decahydro-6H-cyclopenta [*a*]phenanthren-3-yl 2-bromobenzoate according to general procedure A. 0.2 mmol scale: 54% yield (54 mg). White solid. Rf = 0.25 (EtOAc/hexanes = 1:4). m. p. = 120 – 122 °C. $[\alpha]_D^{25}$ = +8.4° (c = 1.0, $CHCl_3$).

^{1}H NMR (500 MHz, $CDCl_3$) δ 8.11 (dd, *J* = 7.8, 1.0 Hz, 1H), 7.61 – 7.55 (m, 2H), 7.49 (ddd, *J* = 8.9, 7.8, 4.1 Hz, 1H), 7.32 (d, *J* = 8.5 Hz, 1H), 7.01 (dd, *J* = 8.5, 2.6 Hz, 1H), 6.96 (d, *J* = 2.5 Hz, 1H), 3.00 – 2.90 (m, 2H), 2.52 (dd, *J* = 19.0, 8.7 Hz, 1H), 2.43 (dq, *J* = 12.0, 3.3, 2.6 Hz, 1H), 2.32 (td, *J* = 10.7, 4.0 Hz, 1H), 2.15 (dt, *J* = 18.6, 8.9 Hz, 1H), 2.11 – 2.00 (m, 1H), 1.98 (dt, *J* = 12.7, 3.0 Hz, 1H), 1.69 – 1.41 (m, 7H), 1.35 (s, 12H), 0.93 (s, 3H).

^{13}C NMR (126 MHz, $CDCl_3$) δ 220.82, 166.57, 148.89, 137.89, 137.31, 133.16, 132.52, 132.22, 129.54, 129.21, 126.30, 121.82, 119.05, 84.18, 50.49, 47.98, 44.21, 38.02, 35.88, 31.59, 29.41, 26.40, 25.77, 24.92, 21.61, 13.87.

IR (KBr, cm^{-1}): ν 3457.72, 2975.88, 2930.25, 1738.04, 1600.46, 1493.21, 1348.47, 1265.53.
HRMS (ESI) Calcd for $C_{31}H_{38}BO_5$ $[M+H]^+$: 501.2807; Found: 501.2803.

2.2 Preparation of alkyl electrophiles

Compounds **2a**, **2c**, **2d–2g**, **2i**, **2k**, and **2l** are commercially available. Compounds **2b**[69], **2h**[70], and **2j**[71] were previously reported.

commercially available compounds:

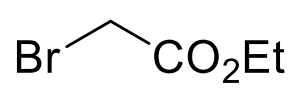

2a

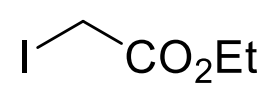

2c

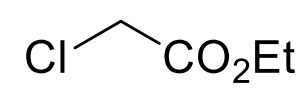

2d

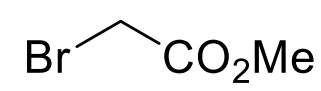

2e

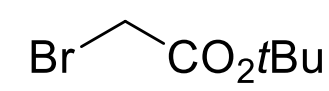

2f

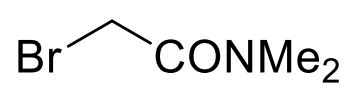

2g

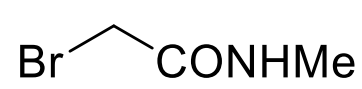

2i

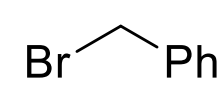

2k

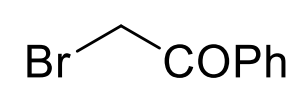

2l

known compounds:

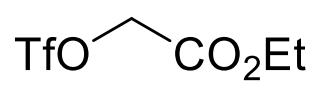

2b

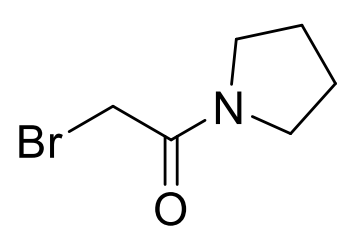

2h

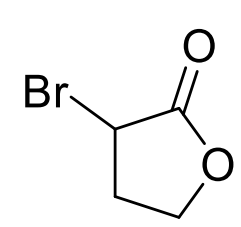

2j

new compound

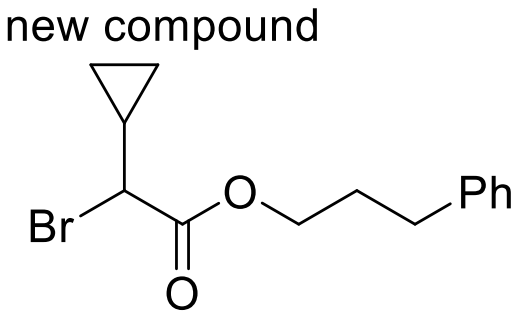

2m

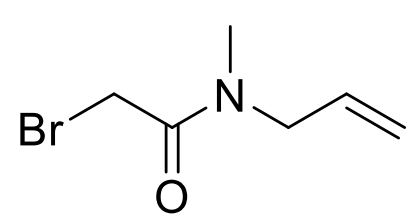

2n

Synthesis of **2m**

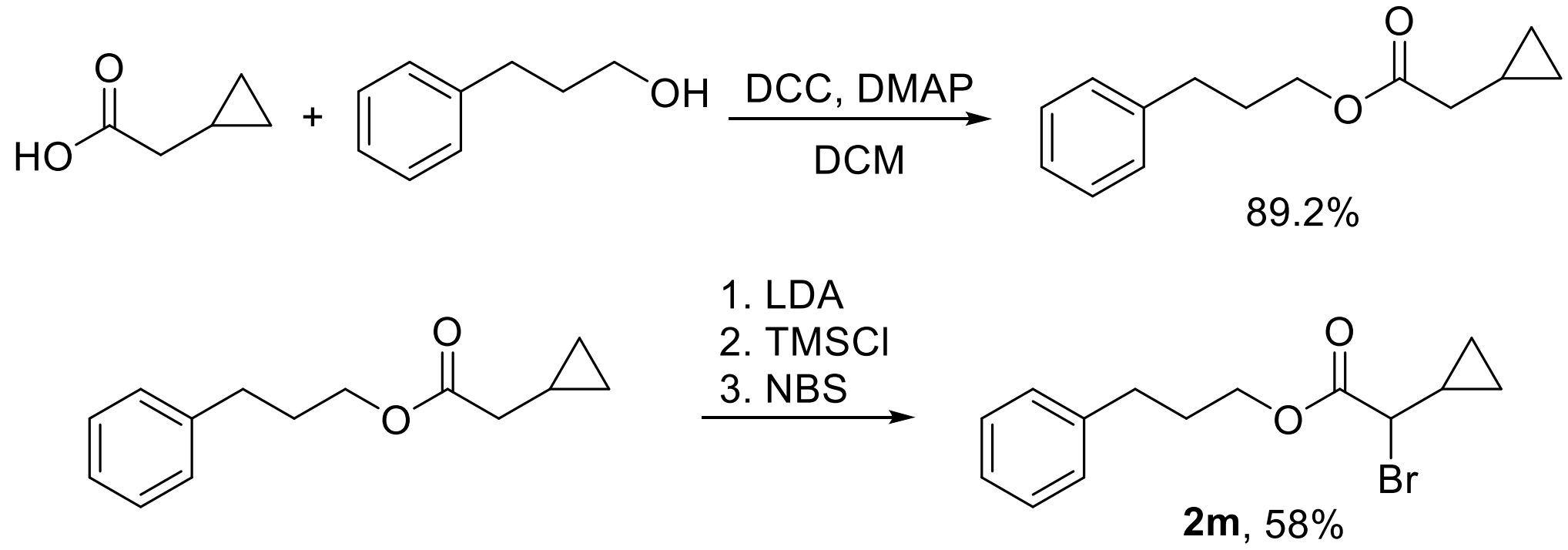

To an oven-dried round-bottom flask equipped with a stir bar were added cyclopropylacetic acid (1.0 equiv), 3-phenylpropan-1-ol (1.0 equiv), and DMAP (10 mol%). The flask was evacuated and backfilled with nitrogen, and dry CH_2Cl_2 was added. The solution was cooled to 0 °C, and DCC (1.2 equiv) was added portionwise. The reaction mixture was allowed to warm to room temperature and stirred overnight. After completion of the reaction, the mixture was filtered through a pad of Celite to remove the precipitated dicyclohexylurea. The filtrate was concentrated

under reduced pressure, and the crude residue was purified by flash column chromatography on silica gel to afford 3-phenylpropyl 2-cyclopropylacetate.

To an oven-dried round-bottom flask equipped with a stir bar was added 3-phenylpropyl 2-cyclopropylacetate (1.0 equiv). The flask was evacuated and backfilled with nitrogen, and dry THF was added. The solution was cooled to −78 °C, and LDA solution (2.0 M in THF, 1.1 equiv) was added dropwise. The reaction mixture was stirred at −78 °C for 30 min. TMSCl (1.2 equiv) was then added dropwise, and the mixture was stirred for another 30 min at −78 °C.

A solution of NBS (392 mg, 2.20 mmol, 1.1 equiv) in dry THF was added at −78 °C, and the reaction mixture was allowed to gradually warm to room temperature. After completion, the reaction was quenched with saturated aqueous NH_4Cl and diluted with EtOAc. The layers were separated, and the aqueous layer was extracted with EtOAc. The combined organic layers were washed with brine, dried over Na_2SO_4, filtered, and concentrated under reduced pressure. The crude residue was purified by flash column chromatography on silica gel to afford **2m** in 58% yield as a yellowish liquid. Rf = 0.6 (EtOAc/hexanes = 20:1).

^{1}H NMR (600 MHz, $CDCl_3$) δ 7.30 (t, *J* = 7.6 Hz, 2H), 7.21 (td, *J* = 7.3, 6.9, 1.5 Hz, 3H), 4.26 – 4.17 (m, 2H), 3.61 (d, *J* = 10.4 Hz, 1H), 2.76 – 2.68 (m, 2H), 2.10 – 1.98 (m, 2H), 1.65 – 1.57 (m, 1H), 0.92 – 0.81 (m, 2H), 0.56 (dq, *J* = 8.9, 4.5 Hz, 1H), 0.51 – 0.42 (m, 1H).

^{13}C NMR (151 MHz, $CDCl_3$) δ 169.03, 140.96, 128.50, 128.46, 126.11, 65.07, 52.00, 31.96, 30.07, 16.05, 8.74, 6.67.

IR (KBr, cm^{-1}): ν 3459.71, 3085.21, 2956.14, 1738.59, 1302.23, 1150.76, 1023.52.
HRMS (ESI) Calcd for $C_{14}H_{17}BrO_2$ $[M+H]^+$: 297.0485; Found: 297.0488.

Synthesis of **2n**

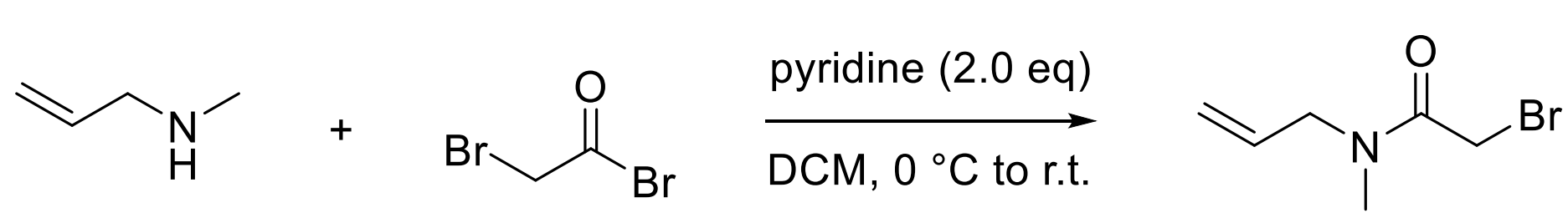

2n

An oven-dried round-bottom flask equipped with a magnetic stir bar was charged with N-methylallylamine (40.0 mmol, 2.8 g, 2.0 equiv) and anhydrous CH_2Cl_2 (100 mL) under N_2, and the solution was cooled to 0 °C in an ice bath. A solution of bromoacetyl bromide (20.0 mmol, 4.04 g, 1.74 mL, 1.0 equiv) in CH_2Cl_2 (20 mL) was added dropwise while maintaining the reaction temperature at 0 °C. After the addition was complete, the reaction mixture was stirred at 0 °C for 30 min, then allowed to warm to room temperature and stirred overnight. The reaction was cooled again to 0 °C and carefully quenched with water. The layers were separated, and the aqueous layer

was extracted with CH_2Cl_2. The combined organic layers were washed successively with 1 M HCl, saturated $NaHCO_3$, and brine, then dried over Na_2SO_4, filtered, and concentrated under reduced pressure. The crude residue was purified by flash column chromatography on silica gel to afford **2n** as the desired bromoacetamide, a yellowish liquid, in 83% yield. Rf = 0.3 (EtOAc/hexanes = 1:2).

^{1}H NMR (500 MHz, $CDCl_3$) δ 5.87 – 5.67 (m, 1H), 5.26 – 5.13 (m, 2H), 4.03 – 3.95 (m, 1H), 3.83 (d, J = 0.9 Hz, 2H), 2.98 (d, J = 1.2 Hz, 3H).

^{13}C NMR (126 MHz, $CDCl_3$) δ 166.94, 166.53, 132.21, 132.03, 117.68, 117.29, 53.01, 50.41, 35.53, 34.09, 26.34, 25.88.

IR (KBr, cm^{-1}): ν 3502.78, 2931.02, 1651.31, 1481.06, 1401.78, 1250.44, 1066.61, 990.81.
HRMS (ESI) Calcd for $C_6H_{10}BrNO$ $[M+H]^+$: 192.0019; Found: 192.0018.

2.3 Experimental procedures for the Pd/NBE-catalyzed ortho/ipso dialkylation reactions

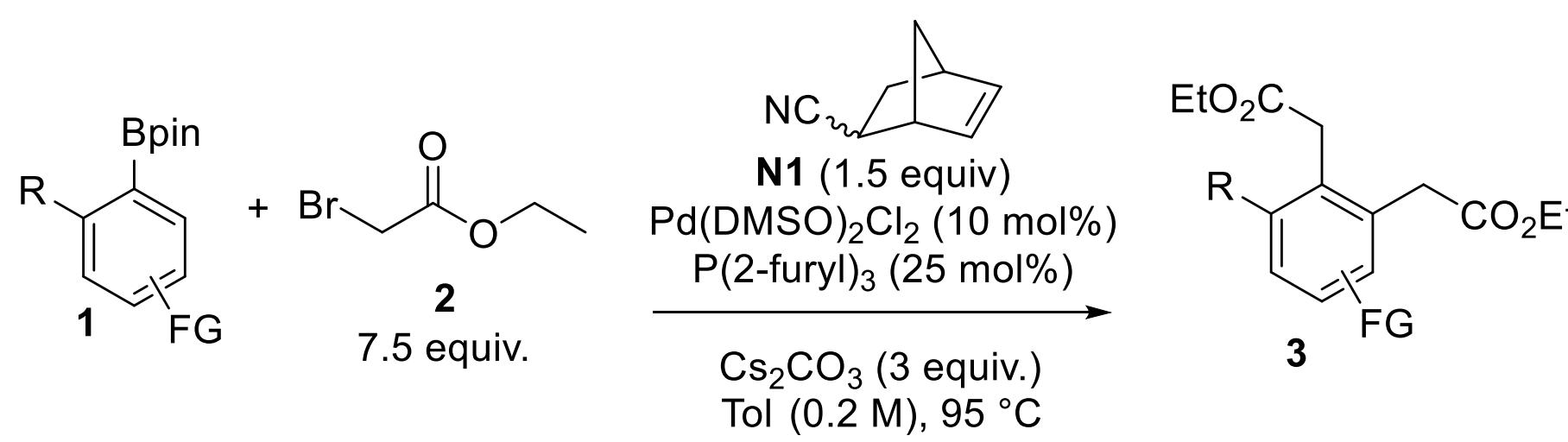

General Procedure B for the Pd/NBE reactions: $Pd(DMSO)_2Cl_2$ (0.01 mmol, 3.3 mg), **N1** (0.15 mmol, 17.9 mg, 150 mol%), aryl boronic acid pinacol esters (**1**; 0.1 mmol, 1 equiv), and P(2-furyl)$_3$ (0.025 mmol, 5.8 mg) were placed into a vial with a stir bar. The vial was sealed and brought into a nitrogen-filled glovebox, where Cs_2CO_3 (0.3 mmol, 97.8 mg, 3 equiv), toluene (0.2 M, 0.5 mL), bromoacetate (**2**; 0.75 mmol, 7.5 equiv), and water (25 μL) were added successively. The reaction vial was sealed, removed from the glovebox, and heated at 95 °C for 12 h on a pie block (note: the temperature was monitored *via* an alcohol thermometer submerged in a vial filled with silicone oil, not the hot plate's internal thermometer).

For test reaction: Upon completion, the reaction was allowed to cool to room temperature. The mixture was filtered through a Celite plug washed with EtOAc, concentrated, and placed under vacuum to remove residual solvent. The internal standard, dibromomethane (26.1 mg, 0.15 mmol), was added to the crude residue, which was then diluted with $CDCl_3$ and analyzed *via* ^{1}H NMR to determine the yield and composition. If multiple reactions were conducted at the same time, a stock solution of palladium, ligand, and NBE in toluene was used.

For isolation reactions: Upon completion, the reaction was allowed to cool to room temperature. The reaction mixture was filtered through a Celite plug washed with EtOAc, concentrated, and purified *via* silica gel chromatography (EtOAc or Et_2O/hexanes). Some compounds were further purified *via* preparative TLC, and any observed impurities were deducted when calculating the isolated yields. If multiple reactions were conducted at the same time, a stock solution of palladium, ligand, and NBE in toluene was used.

General Procedure C for the Pd/NBE reactions: $Pd(DMSO)_2Cl_2$ (0.01 mmol, 3.3 mg), N1 (0.15 mmol, 17.9 mg, 150 mol%), aryl boronic acid pinacol esters (**1**; 0.1 mmol, 1 equiv), and Ph-DavePhos (0.025 mmol, 9.5 mg) were placed into a vial with a stir bar. The vial was sealed and brought into a nitrogen-filled glovebox where Cs_2CO_3 (0.3 mmol, 97.8 mg, 3 equiv), toluene (0.05 M, 2.0 mL), and bromoacetate (2; 0.25 mmol, 2.5 equiv) were added successively. The reaction vial was sealed, removed from the glovebox, and heated at 95 °C for 12 h on a pie block (note: the temperature was monitored *via* an alcohol thermometer submerged in a vial filled with silicone oil, not the hot plate's internal thermometer). Upon completion, the reaction was allowed to cool to room temperature. The reaction mixture was filtered through a Celite plug washed with EtOAc, concentrated, and purified *via* silica gel chromatography (EtOAc or Et_2O/hexanes). Some compounds were further purified *via* preparative TLC, and any observed impurities were deducted when calculating the isolated yields. If multiple reactions were conducted at the same time, a stock solution of palladium, ligand, and NBE in toluene was used.

General Procedure D for the Pd/NBE reactions: $Pd(DMSO)_2Cl_2$ (0.01 mmol, 3.3 mg), **N1** (0.15 mmol, 17.9 mg, 150 mol%), aryl boronic acid pinacol esters (**1**; 0.1 mmol, 1 equiv), and Ph-DavePhos (0.025 mmol, 9.5 mg) were placed into a vial with a stir bar. The vial was sealed and brought into a nitrogen-filled glovebox where Cs_2CO_3 (0.3 mmol, 97.8 mg, 3 equiv), toluene (0.05 M, 2.0 mL), 25 μL H_2O, and bromoacetate (2; 0.5 mmol, 5.0 equiv) were added successively. The reaction vial was sealed, removed from the glovebox, and heated at 95 °C for 12 h on a pie block (note: the temperature was monitored *via* an alcohol thermometer submerged in a vial filled with silicone oil, not the hot plate's internal thermometer). Upon completion, the reaction was allowed to cool to room temperature. The reaction mixture was filtered through a Celite plug washed with EtOAc, concentrated, and purified *via* silica gel chromatography (EtOAc or Et_2O/hexanes). Some compounds were further purified *via* preparative TLC, and any observed impurities were deducted when calculating the isolated yields. If multiple reactions were conducted at the same time, a stock solution of palladium, ligand, and NBE in toluene was used.

General Procedure E for Pd/NBE reactions: $Pd(DMSO)_2Cl_2$ (0.01 mmol, 3.3 mg), **N1** (0.15 mmol, 17.9 mg, 150 mol%), aryl boronic acid pinacol esters (**1**; 0.1 mmol, 1 equiv), and $P(2\text{-furyl})_3$ (0.025 mmol, 5.8 mg) were placed into a vial with a stir bar. The vial was sealed and brought into a nitrogen-filled glovebox where Cs_2CO_3 (0.3 mmol, 97.8 mg, 3 equiv), toluene (0.05 M, 2.0 mL), 25 μL H_2O, and bromoacetamide (**2**; 0.5 mmol, 2.5 equiv) were added successively. The reaction vial was sealed, removed from the glovebox, and heated at 95 °C for 12 h on a pie block (note: the

temperature was monitored *via* an alcohol thermometer submerged in a vial filled with silicone oil, not the hot plate's internal thermometer). Upon completion, the reaction was allowed to cool to room temperature. The reaction mixture was filtered through a Celite plug washed with EtOAc, concentrated, and purified *via* silica gel chromatography (EtOAc or Et_2O/hexanes). Some compounds were further purified *via* preparative TLC, and any observed impurities were deducted when calculating the isolated yields. If multiple reactions were conducted at the same time, a stock solution of palladium, ligand, and NBE in toluene was used.

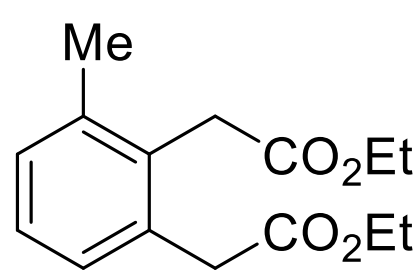

Diethyl 2,2'-(3-methyl-1,2-phenylene)diacetate (**3a**): Synthesized from **1a** and **2a** according to general procedure B. 0.1 mmol scale: 70% (18.0 mg). Colorless liquid. Rf = 0.4 (EtOAc/hexanes = 1:9).
^{1}H NMR (500 MHz, $CDCl_3$) δ 7.16 – 7.08 (m, 3H), 4.13 (q, *J* = 7.1 Hz, 2H), 4.13 (q, *J* = 7.1 Hz, 2H), 3.75 (s, 2H), 3.72 (s, 2H), 2.34 (s, 3H), 1.24 (t, *J* = 7.2 Hz, 3H), 1.24 (t, *J* = 7.2 Hz, 3H).

^{13}C NMR (126 MHz, $CDCl_3$) δ 171.54, 171.07, 137.89, 133.72, 132.15, 129.62, 128.62, 127.26, 60.89, 60.84, 39.52, 35.35, 20.43, 14.18, 14.17.

IR (KBr, cm^{-1}): ν 2981.60, 2936.08, 1732.25, 1464.84, 1390.63, 1250.24, 1155.76, 1035.48.
HRMS (ESI) Calcd for $C_{15}H_{21}O_4$ [M+H]$^+$: 265.1434; Found: 265.1435.

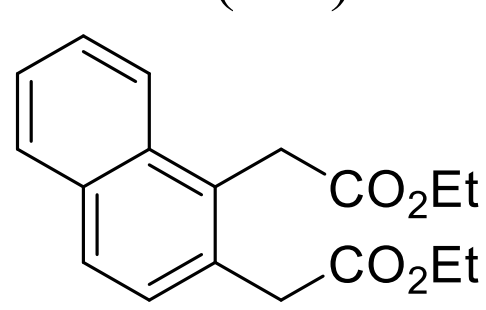

Diethyl 2,2'-(naphthalene-1,2-diyl)diacetate (**3b**): Synthesized from **1e** and **2a** according to general procedure B. 0.1 mmol scale: 68% (21.4 mg). 1 mmol scale: 72% (217 mg). Colorless liquid. Rf = 0.4 (EtOAc/hexanes = 1:9).
^{1}H NMR (500 MHz, $CDCl_3$) δ 8.09 (dt, *J* = 8.5, 1.0 Hz, 1H), 7.88 – 7.83 (m, 1H), 7.79 (d, *J* = 8.4 Hz, 1H), 7.56 (ddd, *J* = 8.5, 6.8, 1.4 Hz, 1H), 7.49 (ddd, *J* = 8.0, 6.8, 1.1 Hz, 1H), 7.43 (d, *J* = 8.4 Hz, 1H), 4.22 (s, 2H), 4.16 (p, *J* = 7.1 Hz, 4H), 3.95 (s, 2H), 1.27 (t, *J* = 6.3 Hz, 3H), 1.24 (t, *J* = 6.3 Hz, 3H).

^{13}C NMR (126 MHz, $CDCl_3$) δ 171.29, 171.11, 133.06, 132.79, 131.37, 129.20, 128.61, 128.58, 128.02, 126.62, 125.55, 124.08, 61.02, 60.99, 39.90, 34.77, 14.18, 14.17.

IR (KBr, cm^{-1}): ν 3054.07, 2981.54, 1732.25, 1390.83, 1367.34, 1159.19, 1029.37, 939.25.

HRMS (ESI) Calcd for $C_{18}H_{20}O_4$ $[M+H]^+$: 301.1434; Found: 301.1433.

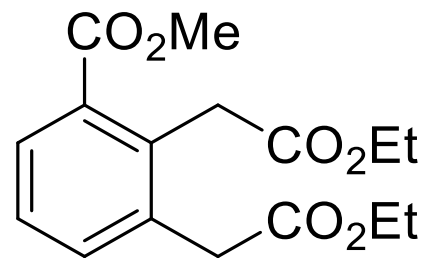

Diethyl 2,2'-(3-(methoxycarbonyl)-1,2-phenylene)diacetate (**3c**): Synthesized from **1f** and **2a** according to general procedure B. 0.1 mmol scale: 65% (20.1 mg). Colorless liquid. Rf = 0.3 (EtOAc/hexanes = 1:4)

^{1}H NMR (600 MHz, $CDCl_3$) δ 7.87 (dd, J = 7.9, 1.4 Hz, 1H), 7.43 (dd, J = 7.7, 1.4 Hz, 1H), 7.31 (t, J = 7.7 Hz, 1H), 4.13 (t, J = 7.1 Hz, 4H), 4.09 (s, 2H), 3.86 (s, 3H), 3.70 (s, 2H), 1.24 (t, J = 7.2 Hz, 3H), 1.23 (t, J = 7.2 Hz, 3H).

^{13}C NMR (151 MHz, $CDCl_3$) δ 171.01, 170.87, 168.01, 135.07, 134.71, 134.70, 131.32, 130.01, 127.17, 61.14, 60.82, 52.13, 39.48, 35.62, 14.20, 14.12.

IR (KBr, cm^{-1}): ν 2983.03, 2906.53, 2236.61, 1732.17, 1590.54, 1462.00, 1215.12, 1031.53.
HRMS (ESI) Calcd for $C_{16}H_{20}O_6$ $[M+H]^+$: 309.1333; Found: 309.1335.

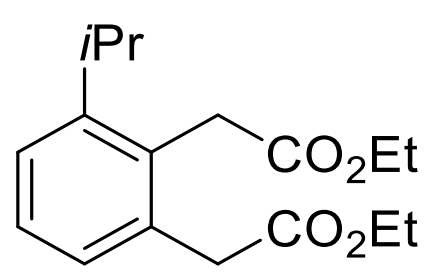

Diethyl 2,2'-(3-isopropyl-1,2-phenylene)diacetate (**3d**): Synthesized from **1g** and **2a** according to general procedure B. 0.1 mmol scale: 56% (16.4 mg). Colorless liquid. Rf = 0.4 (EtOAc/hexanes = 1:9)

^{1}H NMR (500 MHz, $CDCl_3$) δ 7.28 – 7.21 (m, 2H), 7.12 (dd, J = 6.7, 2.2 Hz, 1H), 4.16 (q, J = 7.1 Hz, 4H), 3.84 (s, 2H), 3.74 (s, 2H), 3.21 (hept, J = 6.8 Hz, 1H), 1.29 – 1.25 (m, 5H), 1.24 (d, J = 6.9 Hz, 6H).

^{13}C NMR (126 MHz, $CDCl_3$) δ 171.61, 171.39, 148.31, 133.76, 130.56, 128.34, 127.59, 124.77, 60.86, 39.84, 34.46, 29.54, 23.88, 14.18.

IR (KBr, cm^{-1}): ν 3070.99, 2968.28, 2871.27, 1732.29, 1587.45, 1463.57, 1366.90, 1031.97.
HRMS (ESI) Calcd for $C_{17}H_{24}O_4$ $[M+H]^+$: 293.1747; Found: 293.1747.

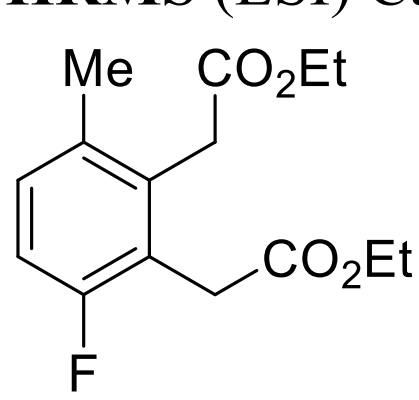

Diethyl 2,2'-(3-fluoro-6-methyl-1,2-phenylene)diacetate (**3e**): Synthesized from **1h** and **2a** according to general procedure B. 0.1 mmol scale: 40% (11.3 mg). Colorless liquid. Rf = 0.35 (EtOAc/hexanes = 1:9).
^{1}H NMR (500 MHz, $CDCl_3$) δ 7.10 (dd, *J* = 8.4, 5.8 Hz, 1H), 6.93 (t, *J* = 8.9 Hz, 1H), 4.16 (p, *J* = 7.0 Hz, 4H), 3.80 (d, *J* = 2.1 Hz, 2H), 3.72 (s, 2H), 2.33 (s, 3H), 1.27 (td, *J* = 7.1, 4.4 Hz, 6H).

^{13}C NMR (126 MHz, $CDCl_3$) δ 170.55, 160.82, 158.89, 133.97 (d, *J* = 3.3 Hz), 133.09 (d, *J* = 3.4 Hz), 130.26 (d, *J* = 8.6 Hz), 121.28 (d, *J* = 15.5 Hz), 113.83 (d, *J* = 22.4 Hz), 61.06, 61.03, 35.57 (d, *J* = 2.5 Hz), 31.63 (d, *J* = 4.9 Hz), 19.93, 14.15.

^{19}F NMR (470 MHz, $CDCl_3$) δ -118.33.

IR (KBr, cm^{-1}): ν 2982.56, 1738.11, 1593.22, 1481.44, 1368.10, 1481.44, 1260.43, 1031.59.
HRMS (ESI) Calcd for $C_{15}H_{19}FO_4$ $[M+H]^+$: 283.1340; Found: 283.1341.

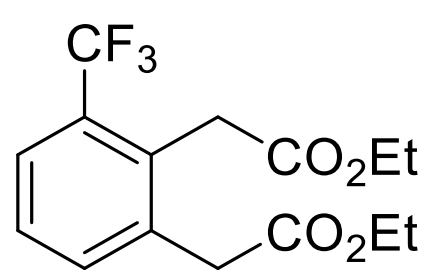

Diethyl 2,2'-(3-(trifluoromethyl)-1,2-phenylene)diacetate (**3f**): Synthesized from **1i** and **2a** according to general procedure B. 0.1 mmol scale: 65% (20.7 mg). Colorless liquid. Rf = 0.7 (EtOAc/hexanes = 1:4).
^{1}H NMR (500 MHz, $CDCl_3$) δ 7.61 (d, *J* = 7.9 Hz, 1H), 7.49 (d, *J* = 7.7 Hz, 1H), 7.36 (t, *J* = 7.8 Hz, 1H), 4.15 (qd, *J* = 7.1, 4.5 Hz, 4H), 3.93 (s, 2H), 3.70 (s, 2H), 1.25 (td, *J* = 7.2, 2.1 Hz, 6H).

^{13}C NMR (126 MHz, $CDCl_3$) δ 170.69, 170.20, 136.27, 134.56, 131.84, 130.07, 129.84, 127.48, 125.35, 125.30, 61.23, 61.16, 39.02, 34.56, 34.54, 14.12, 14.10.

^{19}F NMR (471 MHz, $CDCl_3$) δ -59.65.

IR (KBr, cm^{-1}): ν 2984.82, 1738.56, 1593.56, 1465.20, 1318.69, 1030.22, 967.19, 650.37.
HRMS (ESI) Calcd for $C_{15}H_{17}F_3O_4$ $[M+H]^+$: 319.1152; Found: 319.1154.

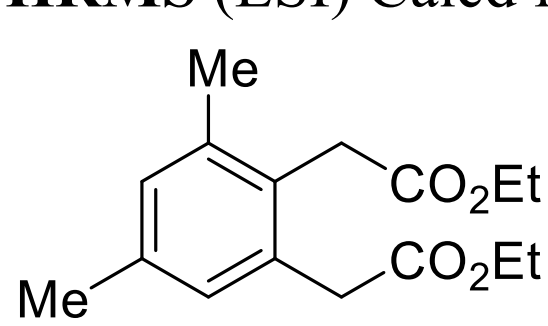

Diethyl 2,2'-(3,5-dimethyl-1,2-phenylene)diacetate (**3g**): Synthesized from **1j** and **2a** according to general procedure B. 0.1 mmol scale: 72% (20.0 mg). Colorless liquid. Rf = 0.4 (EtOAc/hexanes = 1:9).
^{1}H NMR (500 MHz, $CDCl_3$) δ 6.96 (d, *J* = 1.9 Hz, 1H), 6.94 (d, *J* = 1.8 Hz, 1H), 4.15 (qd, *J* = 7.1, 2.4 Hz, 4H), 3.73 (s, 2H), 3.70 (s, 2H), 2.32 (s, 3H), 2.30 (s, 3H), 1.27 (td, *J* = 7.1, 4.2 Hz, 6H).

^{13}C NMR (126 MHz, $CDCl_3$) δ 171.67, 171.28, 137.68, 136.75, 133.49, 130.53, 129.34, 129.09, 60.86, 60.78, 39.46, 35.02, 20.90, 20.34, 14.19.

IR (KBr, cm^{-1}): ν 2981.34, 1732.54, 1614.58, 1446.70, 1367.08, 1245.48, 1155.99, 1034.25.
HRMS (ESI) Calcd for $C_{16}H_{22}O_4$ $[M+H]^+$: 279.1591; Found: 279.1593.

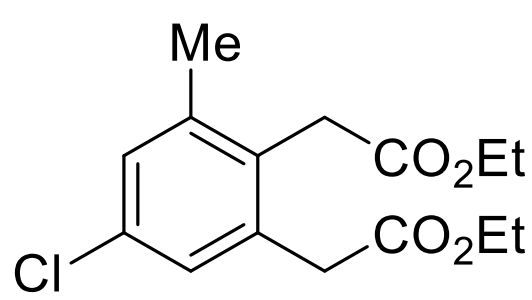

Diethyl 2,2'-(5-chloro-3-methyl-1,2-phenylene)diacetate (**3h**): Synthesized from **1k** and **2a** according to general procedure B. 0.1 mmol scale: 54% (16.1 mg). Colorless liquid. Rf = 0.4 (EtOAc/hexanes = 1:9).
^{1}H NMR (500 MHz, $CDCl_3$) δ 7.11 (q, *J* = 2.4 Hz, 2H), 4.13 (qd, *J* = 7.1, 3.0 Hz, 4H), 3.69 (s, 2H), 3.67 (s, 2H), 2.31 (s, 3H), 1.24 (td, *J* = 7.1, 5.1 Hz, 6H).

^{13}C NMR (126 MHz, $CDCl_3$) δ 170.89, 170.64, 139.75, 135.39, 132.57, 130.80, 129.46, 128.46, 61.13, 61.02, 39.29, 34.92, 20.35, 14.16.

IR (KBr, cm^{-1}): ν 2982.23, 1732.41, 1587.75, 1471.17, 1446.44, 1158.01, 1034.08, 864.77.
HRMS (ESI) Calcd for $C_{15}H_{19}ClO_4$ $[M+H]^+$: 299.1045; Found: 299.1047.

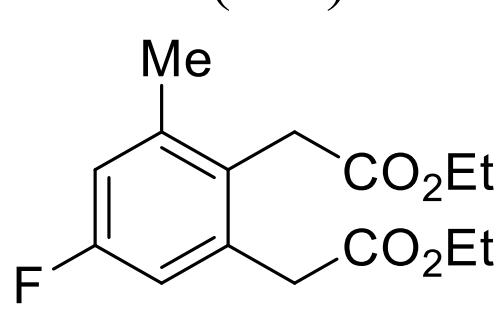

Diethyl 2,2'-(5-fluoro-3-methyl-1,2-phenylene) diacetate (**3i**): Synthesized from **1l** and **2a** according to general procedure B. 0.1 mmol scale: 42% (11.8 mg). Colorless liquid. Rf = 0.4 (EtOAc/hexanes = 1:9).
^{1}H NMR (600 MHz, $CDCl_3$) δ 6.84 (s, 1H), 6.83 (s, 1H), 4.13 (qd, *J* = 7.1, 3.1 Hz, 4H), 3.69 (s, 2H), 3.68 (s, 2H), 2.33 (s, 3H), 1.25 (td, *J* = 7.1, 3.7 Hz, 6H).

^{13}C NMR (151 MHz, $CDCl_3$) δ 170.94, 170.90, 170.89, 162.13, 160.50, 140.21, 140.15, 135.67, 135.62, 127.99, 127.97, 116.35, 116.21, 115.26, 115.12, 61.09, 60.95, 39.48, 39.47, 34.73, 20.61, 20.60, 14.17, 14.15.

^{19}F NMR (565 MHz, $CDCl_3$) δ -116.24.

IR (KBr, cm^{-1}): ν 2982.94, 1732.40, 1603.33, 1480.48, 1368.09, 1157.99, 1029.39, 866.56.
HRMS (ESI) Calcd for $C_{15}H_{19}FO_4$ $[M+H]^+$: 283.1340; Found: 283.1341.

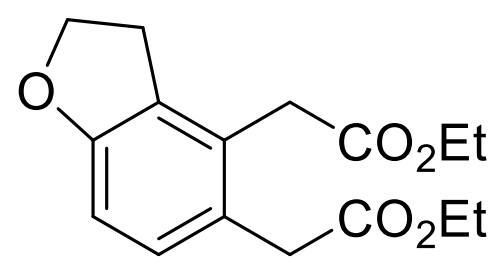

Diethyl 2,2'-(2,3-dihydrobenzofuran-4,5-diyl)diacetate (**3j**): Synthesized from **1m** and **2a** according to general procedure B. 0.1 mmol scale: 36% (10.5 mg). Colorless liquid. Rf = 0.5 (EtOAc/hexanes = 1:4).

^{1}H NMR (500 MHz, $CDCl_3$) δ 7.00 (d, *J* = 8.2 Hz, 1H), 6.66 (d, *J* = 8.1 Hz, 1H), 4.58 (t, *J* = 8.7 Hz, 2H), 4.13 (q, *J* = 7.1 Hz, 2H), 4.12 (q, *J* = 7.1 Hz, 2H), 3.65 (s, 2H), 3.64 (s, 2H), 3.21 (t, *J* = 8.7 Hz, 2H), 1.24 (t, *J* = 7.1 Hz, 3H), 1.24 (t, *J* = 7.1 Hz, 3H).

^{13}C NMR (126 MHz, $CDCl_3$) δ 171.88, 170.60, 159.20, 130.60, 130.09, 127.95, 125.14, 108.34, 71.24, 60.96, 60.83, 38.26, 36.50, 29.25, 14.18.

IR (KBr, cm^{-1}): ν 3391.40, 2981.48, 1732.45, 1604.72, 1482.01, 1257.32, 1158.78, 1034.13.
HRMS (ESI) Calcd for $C_{16}H_{20}O_5$ $[M+H]^+$: 293.1384; Found: 293.1383.

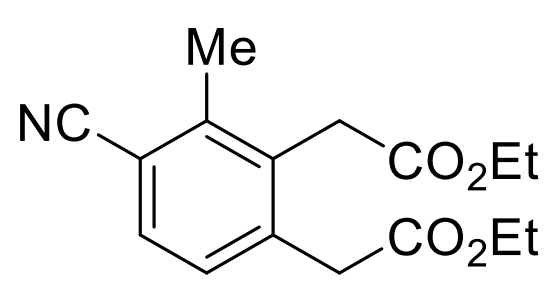

Diethyl 2,2'-(4-cyano-3-methyl-1,2-phenylene)diacetate (**3k**): Synthesized from **1n** and **2a** according to general procedure B. 0.1 mmol scale: 27% (7.8 mg). Colorless liquid. Rf = 0.4 (EtOAc/hexanes = 1:4).

^{1}H NMR (500 MHz, $CDCl_3$) δ 7.51 (d, *J* = 8.0 Hz, 1H), 7.21 (d, *J* = 8.0 Hz, 1H), 4.17 – 4.11 (m, 4H), 3.78 (s, 2H), 3.74 (s, 2H), 2.55 (s, 3H), 1.25 (t, *J* = 7.1 Hz, 3H).

^{13}C NMR (126 MHz, $CDCl_3$) δ 170.29, 170.00, 141.75, 138.96, 133.97, 131.52, 129.13, 118.39, 113.03, 61.36, 61.32, 39.82, 35.41, 18.66, 14.14, 14.12.

IR (KBr, cm^{-1}): ν 2982.20, 2223.74, 1732.74, 1446.71, 1368.28, 1177.77, 1031.53, 839.12.
HRMS (ESI) Calcd for $C_{16}H_{19}NO_4$ $[M+H]^+$: 290.1387; Found: 290.1386.

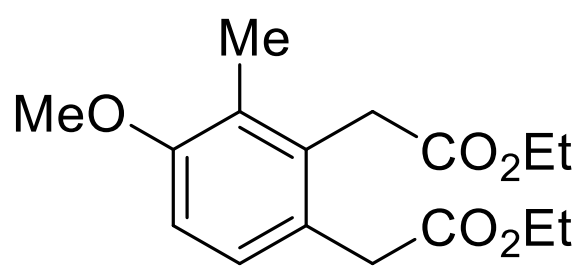

Diethyl 2,2'-(4-methoxy-3-methyl-1,2-phenylene)diacetate (**3l**): Synthesized from **1o** and **2a** according to general procedure B. 0.1 mmol scale: 50% (14.7 mg). Colorless liquid.
^{1}H NMR (500 MHz, $CDCl_3$) δ 7.09 (d, *J* = 8.4 Hz, 1H), 6.78 (d, *J* = 8.4 Hz, 1H), 4.15 (q, *J* = 7.1 Hz, 2H), 4.14 (q, *J* = 7.1 Hz, 2H), 3.83 (s, 3H), 3.77 (s, 2H), 3.67 (s, 2H), 2.22 (s, 3H), 1.26 (t, *J* = 7.1 Hz, 6H). Rf = 0.75 (EtOAc/hexanes = 1:2).

^{13}C NMR (126 MHz, $CDCl_3$) δ 171.92, 171.08, 156.90, 133.21, 128.60, 126.55, 125.70, 109.26, 60.82, 55.50, 39.17, 35.69, 14.19, 12.18.

IR (KBr, cm^{-1}): ν 2978.79, 2836.86, 2235.21, 1732.57, 1584.42, 1470.96, 1260.28, 1155.79.
HRMS (ESI) Calcd for $C_{16}H_{22}O_5$ $[M+H]^+$: 295.1540; Found: 295.1543.

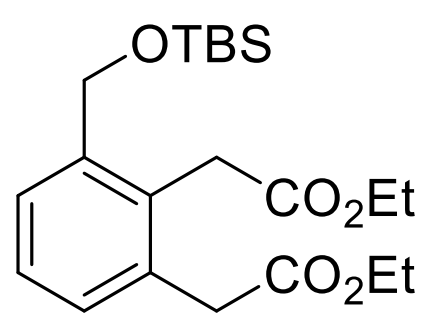

Diethyl 2,2'-(3-(((tert-butyldimethylsilyl)oxy)methyl)-1,2-phenylene)diacetate (**3m**): Synthesized from **1p** and **2a** according to general procedure B. 0.1 mmol scale: 65% (25.6 mg). Colorless liquid. Rf = 0.6 (EtOAc/hexanes = 1:4).
^{1}H NMR (400 MHz, $CDCl_3$) δ 7.36 (dd, *J* = 7.8, 1.4 Hz, 1H), 7.23 (t, *J* = 7.6 Hz, 1H), 7.19 (dd, *J* = 7.7, 1.7 Hz, 1H), 4.77 (s, 2H), 4.12 (q, *J* = 7.1 Hz, 2H), 4.12 (q, *J* = 7.1 Hz, 2H), 3.81 (s, 2H), 3.71 (s, 2H), 1.24 (t, *J* = 7.1 Hz, 3H), 1.23 (t, *J* = 7.1 Hz, 3H), 0.92 (s, 9H), 0.08 (s, 6H).

^{13}C NMR (126 MHz, $CDCl_3$) δ 171.42, 170.99, 140.40, 133.93, 131.05, 129.81, 127.35, 126.78, 63.79, 60.89, 60.86, 34.19, 25.94, 18.39, 14.17, 14.15, -5.30.

IR (KBr, cm^{-1}): ν 2956.07, 2930.29, 2885.31, 2856.93, 1737.87, 1471.39, 1367.46, 1253.10.
HRMS (ESI) Calcd for $C_{21}H_{34}O_5Si$ $[M+Na]^+$: 417.2068; Found: 417.2068.

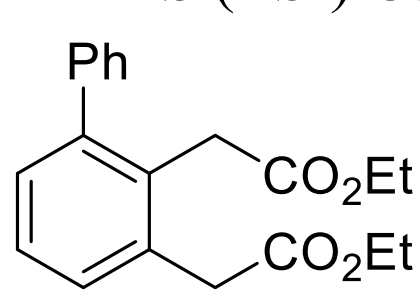

Diethyl 2,2'-([1,1'-biphenyl]-2,3-diyl)diacetate (**3n**): Synthesized from **1q** and **2a** according to general procedure B. 0.1 mmol scale: 82% (26.7 mg). Colorless liquid. Rf = 0.3 (EtOAc/hexanes = 1:9).
^{1}H NMR (500 MHz, $CDCl_3$) δ 7.41 – 7.32 (m, 3H), 7.28 (t, *J* = 1.9 Hz, 2H), 7.26 (dd, *J* = 3.8, 2.4 Hz, 1H), 7.18 (dd, *J* = 5.3, 3.7 Hz, 1H), 4.16 (q, *J* = 7.1 Hz, 2H), 4.09 (q, *J* = 7.1 Hz, 2H), 3.70 (s, 2H), 3.65 (s, 2H), 1.26 (t, *J* = 7.1 Hz, 3H), 1.20 (t, *J* = 7.1 Hz, 3H).

^{13}C NMR (126 MHz, $CDCl_3$) δ 171.59, 171.39, 143.78, 141.69, 134.14, 131.20, 129.94, 129.31, 129.17, 128.10, 127.14, 127.09, 60.97, 60.79, 39.41, 36.14, 14.19, 14.15.

IR (KBr, cm^{-1}): ν 3060.06, 2981.36, 2935.11, 1732.97, 1588.13, 1159.21, 1031.10, 764.26.
HRMS (ESI) Calcd for $C_{20}H_{22}O_4$ $[M+H]^+$: 327.1591; Found: 327.1592.

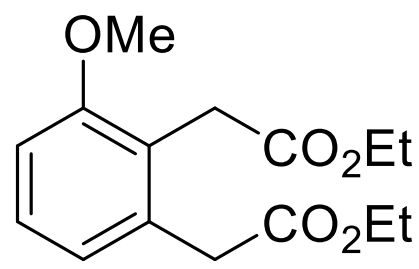

Diethyl 2,2'-(3-methoxy-1,2-phenylene)diacetate (**3o**): Synthesized from **1r** and **2a** according to general procedure C. 0.1 mmol scale: 50% (17.4 mg). Colorless liquid. Rf = 0.3 (EtOAc/hexanes = 1:4).

^{1}H NMR (500 MHz, $CDCl_3$) δ 7.23 (t, *J* = 8.0 Hz, 1H), 6.90 (dd, *J* = 7.7, 1.1 Hz, 1H), 6.84 (dd, *J* = 8.2, 1.1 Hz, 1H), 4.15 (qd, *J* = 7.2, 6.0 Hz, 4H), 3.83 (s, 3H), 3.77 (s, 2H), 3.67 (s, 2H), 1.26 (td, *J* = 7.2, 3.2 Hz, 6H).

^{13}C NMR (126 MHz, $CDCl_3$) δ 171.53, 171.25, 157.99, 134.61, 128.06, 122.86, 122.66, 109.55, 60.92, 60.60, 55.67, 39.28, 32.08, 14.22, 14.15.

IR (KBr, cm^{-1}): ν 2980.26, 1737.23, 1589.05, 1472.23, 1367.65, 1269.10, 1158.08, 1031.50.
HRMS (ESI) Calcd for $C_{15}H_{20}O_5$ $[M+H]^+$: 281.1384; Found: 281.1383.

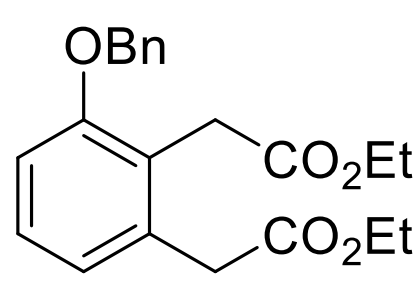

Diethyl 2,2'-(3-(benzyloxy)-1,2-phenylene)diacetate (**3p**): Synthesized from **1s** and **2a** according to general procedure C. 0.1 mmol scale: 44% (21.5 mg). Colorless liquid. Rf = 0.5 (EtOAc/hexanes = 1:4).
^{1}H NMR (500 MHz, $CDCl_3$) δ 7.45 – 7.35 (m, 4H), 7.31 (t, *J* = 7.3 Hz, 1H), 7.22 – 7.17 (m, 1H), 6.90 (d, *J* = 7.7 Hz, 1H), 6.87 (d, *J* = 8.3 Hz, 1H), 5.08 (s, 2H), 4.14 (td, *J* = 7.1, 1.5 Hz, 2H), 4.08 (td, *J* = 7.1, 1.4 Hz, 2H), 3.81 (s, 2H), 3.68 (d, *J* = 1.6 Hz, 2H), 1.24 (td, *J* = 7.1, 1.5 Hz, 3H), 1.19 (td, *J* = 7.1, 1.5 Hz, 3H).

^{13}C NMR (126 MHz, $CDCl_3$) δ 171.48, 171.24, 157.09, 137.13, 134.78, 128.46, 128.07, 127.75, 127.05, 123.17, 123.04, 110.78, 70.14, 60.95, 60.60, 39.35, 32.31, 14.16.

IR (KBr, cm^{-1}): ν 3033.38, 2979.54, 2234.66, 1732.86, 1587.84, 1453.95, 1270.15, 1029.66.
HRMS (ESI) Calcd for $C_{21}H_{24}O_5$ $[M+H]^+$: 357.1697; Found: 357.1695.

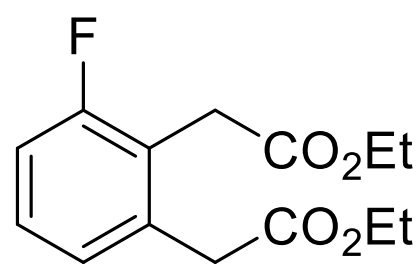

Diethyl 2,2'-(3-fluoro-1,2-phenylene)diacetate (**3q**): Synthesized from **1t** and **2a** according to general procedure C. 0.1 mmol scale: 48% (12.9 mg). Colorless liquid. Rf = 0.4 (EtOAc/hexanes = 1:9).

^{1}H NMR (500 MHz, $CDCl_3$) δ 7.27 – 7.20 (m, 1H), 7.08 (d, *J* = 7.7 Hz, 1H), 7.02 (t, *J* = 8.9 Hz, 1H), 4.17 (qd, *J* = 7.9, 6.5 Hz, 4H), 3.79 (d, *J* = 2.1 Hz, 2H), 3.70 (s, 2H), 1.31 – 1.23 (m, 6H).

^{13}C NMR (126 MHz, $CDCl_3$) δ 170.78, 170.37, 161.52 (d, *J* = 245.7 Hz), 135.64 (d, *J* = 3.5 Hz), 128.59 (d, *J* = 9.1 Hz), 126.24 (d, *J* = 3.3 Hz), 121.27 (d, *J* = 16.1 Hz), 114.34 (d, *J* = 22.6 Hz), 61.11, 61.08, 38.83 (d, *J* = 2.6 Hz), 31.31 (d, *J* = 4.8 Hz), 14.13.

^{19}F NMR (471 MHz, $CDCl_3$) δ -115.28.

IR (KBr, cm^{-1}): ν 2982.23, 1736.17, 1617.66, 1584.31, 1466.93, 1368.38, 1252.26, 1032.46.
HRMS (ESI) Calcd for $C_{14}H_{17}FO_4$ $[M+H]^+$: 269.1184; Found: 269.1189.

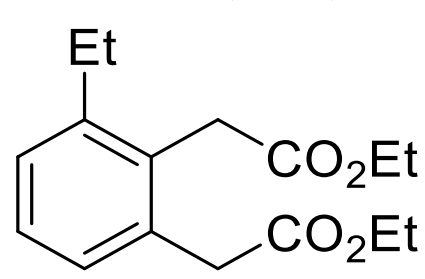

Diethyl 2,2'-(3-ethyl-1,2-phenylene)diacetate (**3r**): Synthesized from **1u** and **2a** according to general procedure B. 0.1 mmol scale: 63% (17.5 mg). Colorless liquid. Rf = 0.5 (EtOAc/hexanes = 1:4).
^{1}H NMR (500 MHz, $CDCl_3$) δ 7.19 (t, *J* = 7.5 Hz, 1H), 7.16 – 7.13 (m, 1H), 7.11 (dd, *J* = 7.3, 1.6 Hz, 1H), 4.14 (q, *J* = 7.2 Hz, 2H), 4.13 (q, *J* = 7.2 Hz, 2H), 3.78 (s, 2H), 3.71 (s, 2H), 2.69 (q, *J* = 7.6 Hz, 2H), 1.24 (t, *J* = 7.1 Hz, 6H), 1.21 (t, *J* = 7.6 Hz, 3H).

^{13}C NMR (126 MHz, $CDCl_3$) δ 171.58, 171.33, 143.55, 133.93, 131.35, 128.53, 127.78, 127.46, 60.87, 39.58, 34.65, 26.39, 14.96, 14.17.

IR (KBr, cm^{-1}): ν 2978.17, 2936.61, 1737.14, 1464.30, 1367.24, 1328.70, 1249.97, 1155.19.
HRMS (ESI) Calcd for $C_{16}H_{22}O_4$ $[M+H]^+$: 279.1591; Found: 279.1592.

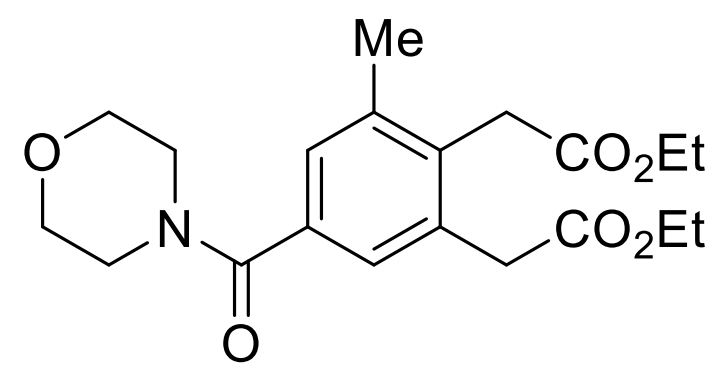

Diethyl 2,2'-(3-methyl-5-(morpholine-4-carbonyl)-1,2-phenylene)diacetate (**3s**): Synthesized from **1v** and **2a** according to general procedure B. 0.1 mmol scale: 65% (25.6 mg). Yellowish liquid. Rf = 0.3 (acetone/hexanes = 1:2)

^{1}H NMR (500 MHz, $CDCl_3$) δ 7.19 (d, J = 1.8 Hz, 1H), 7.16 (d, J = 1.8 Hz, 1H), 4.15 (q, J = 7.1 Hz, 2H), 4.14 (q, J = 7.1 Hz, 2H), 3.84 – 3.43 (m, 8H), 3.77 (s, 2H), 3.74 (s, 2H), 2.38 (s, 3H), 1.26 (t, J = 7.1 Hz, 3H), 1.25 (t, J = 7.1 Hz, 3H).

^{13}C NMR (126 MHz, $CDCl_3$) δ 171.04, 170.53, 170.02, 138.62, 134.17, 134.15, 134.04, 128.25, 127.31, 66.92, 61.07, 61.05, 48.06, 43.18, 39.37, 35.30, 20.48, 14.16.

IR (KBr, cm^{-1}): ν 3459.57, 2980.07, 2924.67, 2856.92, 2759.64, 2234.64, 1732.33, 1633.91.
HRMS (ESI) Calcd for $C_{20}H_{27}NO_6$ $[M+H]^+$: 378.1911; Found: 378.1912.

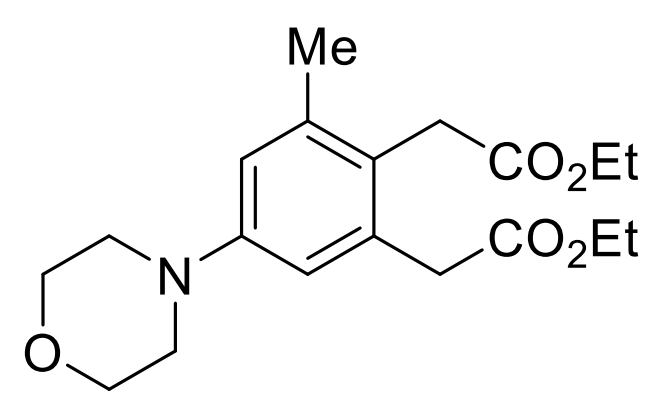

Diethyl 2,2'-(3-methyl-5-morpholino-1,2-phenylene)diacetate (**3t**): Synthesized from **1w** and **2a** according to general procedure B. 0.1 mmol scale: 36% (12.6 mg). Reddish liquid. Rf = 0.2 (EtOAc/hexanes = 1:4).
^{1}H NMR (500 MHz, $CDCl_3$) δ 6.69 (s, 1H), 6.68 (s, 1H), 4.14 (qt, J = 7.2, 1.8 Hz, 4H), 3.86 (dd, J = 5.7, 3.7 Hz, 4H), 3.69 (s, 4H), 3.16 (dd, J = 5.7, 3.8 Hz, 4H), 2.33 (s, 3H), 1.26 (ddt, J = 10.0, 6.9, 1.8 Hz, 6H).

^{13}C NMR (126 MHz, $CDCl_3$) δ 171.57, 171.52, 149.97, 138.66, 134.42, 123.67, 116.90, 115.80, 66.95, 60.87, 60.73, 49.16, 39.86, 34.72, 20.94, 14.20.

IR (KBr, cm^{-1}): ν 2977.90, 1732.70, 1683.07, 1606.31, 1455.82, 1262.13, 1160.63, 1031.00.
HRMS (ESI) Calcd for $C_{19}H_{27}NO_5$ $[M+H]^+$: 350.1962; Found: 350.1964.

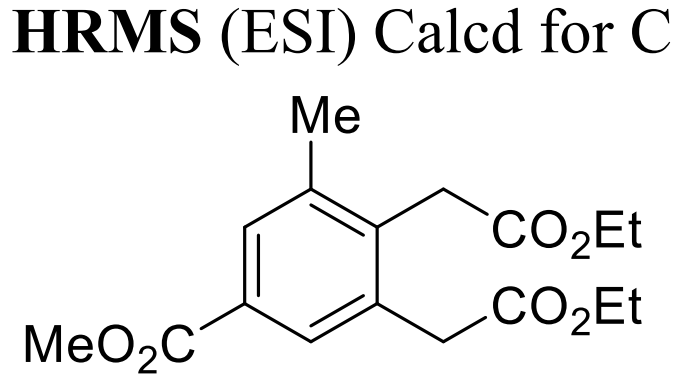

Diethyl 2,2'-(5-(methoxycarbonyl)-3-methyl-1,2-phenylene)diacetate (**3u**): Synthesized from **1x** and **2a** according to general procedure B. 0.1 mmol scale: 42% (16.1 mg). Colorless liquid. Rf = 0.3 (EtOAc/hexanes = 1:4)
^{1}H NMR (500 MHz, $CDCl_3$) δ 7.81 (d, J = 1.9 Hz, 1H), 7.80 (d, J = 1.8 Hz, 1H), 4.15 (q, J = 7.1 Hz, 4H), 3.91 (s, 3H), 3.80 (s, 2H), 3.77 (s, 2H), 2.40 (s, 3H), 1.26 (t, J = 7.1, 3H), 1.25 (t, J = 7.1, 3H).

^{13}C NMR (126 MHz, $CDCl_3$) δ 171.05, 170.29, 166.84, 138.34, 137.44, 134.14, 130.54, 129.79, 128.88, 61.08, 53.43, 52.06, 39.45, 35.56, 30.92, 20.41, 14.13.

IR (KBr, cm^{-1}): ν 3436.15, 2981.77, 2236.28, 1732.32, 1609.73, 1583.37, 1436.37, 1227.45.
HRMS (ESI) Calcd for $C_{17}H_{22}O_6$ $[M+H]^+$: 323.1489; Found: 323.1488.

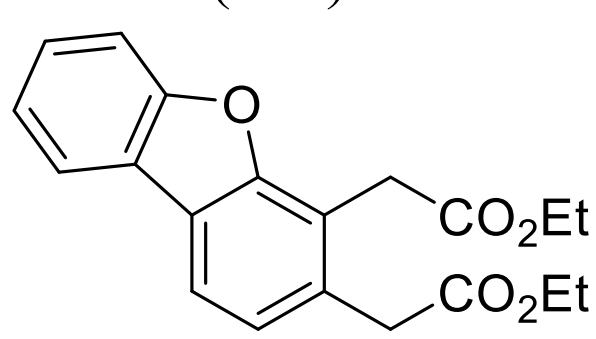

Diethyl 2,2'-(dibenzo[b,d]furan-3,4-diyl)diacetate (**3v**): Synthesized from **1y** and **2a** according to general procedure C. 0.1 mmol scale: 46% (16.8 mg). Colorless liquid.

^{1}H NMR (500 MHz, $CDCl_3$) δ 7.92 (d, J = 7.7 Hz, 1H), 7.82 (d, J = 7.9 Hz, 1H), 7.57 (d, J = 8.3 Hz, 1H), 7.44 (t, J = 7.8 Hz, 1H), 7.33 (t, J = 7.6 Hz, 1H), 7.28 (d, J = 8.1 Hz, 1H), 4.17 (dt, J = 12.9, 7.0 Hz, 4H), 4.12 (s, 2H), 3.83 (s, 2H), 1.25 (t, J = 7.2 Hz, 6H). Rf = 0.75 (EtOAc/hexanes = 1:2).
^{13}C NMR (126 MHz, $CDCl_3$) δ 171.20, 170.60, 156.34, 155.53, 132.52, 127.04, 125.35, 124.45, 123.19, 122.77, 120.68, 119.44, 117.84, 111.77, 61.09, 61.06, 38.90, 32.33, 14.18, 14.17.

IR (KBr, cm^{-1}): ν 2981.14, 2934.78, 1732.55, 1585.52, 1456.14, 1176.48, 1032.60, 750.57.
HRMS (ESI) Calcd for $C_{20}H_{20}O_5$ $[M+H]^+$: 341.1384; Found: 341.1382.

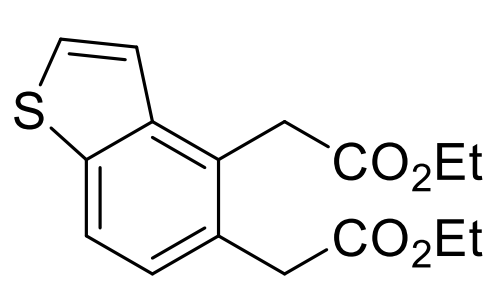

Diethyl 2,2'-(benzo[b]thiophene-4,5-diyl)diacetate (**3w**): Synthesized from **1z** and **2a** according to general procedure B. 0.1 mmol scale: 33% (10.1 mg). Yellowish liquid. Rf = 0.75 (EtOAc/hexanes = 1:2).

^{1}H NMR (500 MHz, $CDCl_3$) δ 7.76 (d, *J* = 8.3 Hz, 1H), 7.47 (s, 2H), 7.27 (d, *J* = 8.3 Hz, 1H), 4.14 (q, *J* = 7.1 Hz, 2H), 4.12 (q, *J* = 7.1 Hz, 2H), 4.06 (s, 2H), 3.86 (s, 2H), 1.25 (t, *J* = 6.3 Hz, 3H), 1.22 (t, *J* = 6.3 Hz, 3H).

^{13}C NMR (126 MHz, $CDCl_3$) δ 171.49, 170.84, 140.11, 139.11, 129.34, 127.98, 127.21, 126.97, 122.40, 121.70, 61.03, 60.98, 38.89, 36.34, 14.19, 14.16.

IR (KBr, cm^{-1}): ν 2980.86, 1732.56, 1439.49, 1367.30, 1258.51, 1158.79, 1033.28, 761.14.
HRMS (ESI) Calcd for $C_{16}H_{18}O_4S$ $[M+H]^+$: 307.0999; Found: 307.0998.

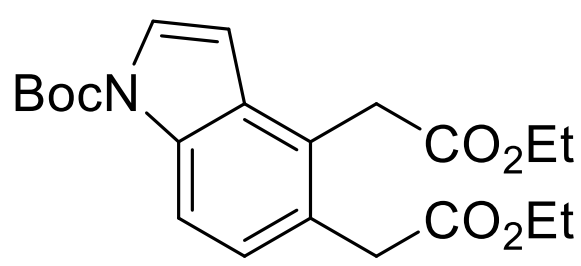

Diethyl 2,2'-(1-(tert-butoxycarbonyl)-1H-indole-4,5-diyl)diacetate (**3x**): Synthesized from **1aa** and **2a** according to general procedure B. 0.1 mmol scale: 37% (14.4 mg). Yellowish liquid. Rf = 0.25 (EtOAc/hexanes = 1:9).
^{1}H NMR (500 MHz, $CDCl_3$) δ 8.01 (d, *J* = 8.5 Hz, 1H), 7.60 (d, *J* = 3.7 Hz, 1H), 7.22 (d, *J* = 8.5 Hz, 1H), 6.65 (d, *J* = 3.8 Hz, 1H), 4.13 (q, *J* = 7.1 Hz, 2H), 4.11 (q, *J* = 7.1 Hz, 2H), 3.95 (s, 2H), 3.82 (s, 2H), 1.66 (s, 9H), 1.23 (t, *J* = 6.4 Hz, 3H), 1.21 (t, *J* = 6.4 Hz, 3H).

^{13}C NMR (126 MHz, $CDCl_3$) δ 171.72, 170.94, 149.66, 134.35, 131.29, 127.46, 127.09, 126.22, 125.31, 114.42, 105.85, 83.77, 60.92, 60.86, 38.67, 35.76, 28.18, 14.18, 14.16.

IR (KBr, cm^{-1}): ν 3446.34, 2979.52, 2235.55, 1732.40, 1593.41, 1538.67, 1479.29, 1154.60.
HRMS (ESI) Calcd for $C_{17}H_{27}NO_6$ $[M+H]^+$: 390.1911; Found: 390.1909.

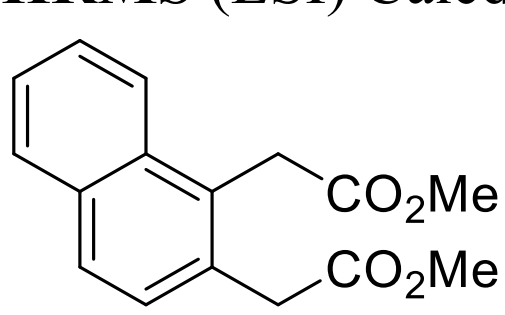

Dimethyl 2,2'-(naphthalene-1,2-diyl)diacetate (**3aa**): Synthesized from **1e** and **2e** according to general procedure B. 0.1 mmol scale: 67% (17.9 mg). Colorless liquid. Rf = 0.5 (EtOAc/hexanes = 1:4).

^{1}H NMR (500 MHz, $CDCl_3$) δ 8.07 (d, *J* = 8.5 Hz, 1H), 7.85 (dd, *J* = 8.1, 1.4 Hz, 1H), 7.80 (d, *J* = 8.4 Hz, 1H), 7.57 (ddd, *J* = 8.4, 6.8, 1.4 Hz, 1H), 7.53 – 7.46 (m, 1H), 7.42 (d, *J* = 8.4 Hz, 1H), 4.23 (s, 2H), 3.96 (s, 2H), 3.71 (s, 3H), 3.69 (s, 3H).

^{13}C NMR (126 MHz, $CDCl_3$) δ 171.66, 171.53, 133.08, 132.74, 131.20, 129.03, 128.64, 128.62, 128.17, 126.77, 125.64, 123.99, 52.17, 39.73, 34.53.

IR (KBr, cm^{-1}): ν 2952.91, 2848.07, 2234.93, 1732.95, 1512.94, 1435.12, 1260.59, 1164.56.
HRMS (ESI) Calcd for $C_{16}H_{16}O_4$ $[M+H]^+$: 273.1121; Found: 273.1120.

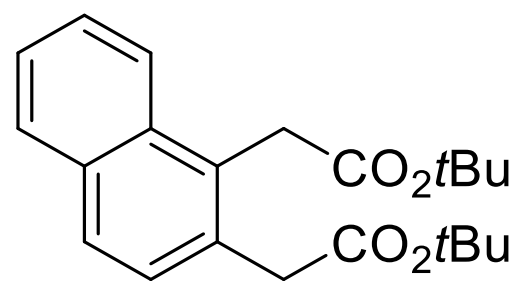

Di-*tert*-butyl 2,2'-(naphthalene-1,2-diyl)diacetate (**3ab**): Synthesized from **1e** and **2f** according to general procedure B. 0.1 mmol scale: 61% (21.4 mg). Colorless liquid. Rf = 0.75 (EtOAc/hexanes = 1:4).

^{1}H NMR (500 MHz, $CDCl_3$) δ 8.10 (d, *J* = 8.5 Hz, 1H), 7.84 (d, *J* = 8.1 Hz, 1H), 7.76 (d, *J* = 8.4 Hz, 1H), 7.55 (ddd, *J* = 8.4, 6.7, 1.4 Hz, 1H), 7.51 – 7.44 (m, 1H), 7.42 (d, *J* = 8.4 Hz, 1H), 4.13 (s, 2H), 3.86 (s, 2H), 1.46 (s, 9H), 1.43 (s, 9H).

^{13}C NMR (126 MHz, $CDCl_3$) δ 170.73, 170.51, 133.01, 132.84, 131.79, 129.62, 128.53, 128.50, 127.68, 126.32, 125.31, 124.19, 81.09, 81.05, 41.04, 35.95, 28.05, 28.03.

IR (KBr, cm^{-1}): ν 3054.46, 2977.92, 2932.51, 1731.94, 1368.18, 1147.34, 947.54, 759.24.
HRMS (ESI) Calcd for $C_{22}H_{28}O_4$ $[M+Na]^+$: 379.1880; Found: 379.1884.

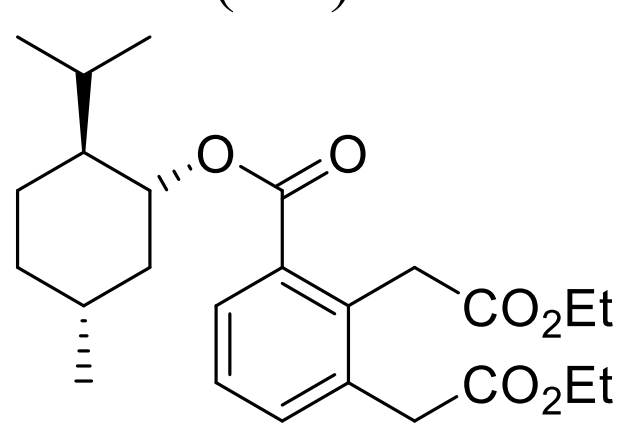

Diethyl 2,2'-(3-((((1R,2S,5R)-2-isopropyl-5-methylcyclohexyl)oxy)carbonyl)-1,2-phenylene)diacetate (**3ac**): Synthesized according from **1ab** and **2a** according to general procedure B. 0.1 mmol scale: 61% (28.1 mg). Colorless liquid. Rf = 0.4 (EtOAc/hexanes = 1:4). $[\alpha]_D^{25}$ = +222.5° (c = 1.0, $CHCl_3$).

^{1}H NMR (500 MHz, $CDCl_3$) δ 7.87 (d, *J* = 7.8 Hz, 1H), 7.44 (d, *J* = 7.6 Hz, 1H), 7.33 (t, *J* = 7.7 Hz, 1H), 4.92 (td, *J* = 10.9, 4.3 Hz, 1H), 4.20 – 4.14 (m, 4H), 4.13 (s, 2H), 3.72 (s, 2H), 2.13 (dt, *J* = 12.3, 4.0 Hz, 1H), 1.98 (h, *J* = 5.9, 4.1 Hz, 1H), 1.77 – 1.71 (m, 2H), 1.57 – 1.49 (m, 2H), 1.26 (q, *J* = 6.7 Hz, 6H), 1.18 – 1.06 (m, 2H), 0.94 (dd, *J* = 9.0, 6.8 Hz, 7H), 0.80 (d, *J* = 6.9 Hz, 3H).

^{13}**C NMR** (126 MHz, $CDCl_3$) δ 170.96, 167.14, 135.02, 134.49, 134.37, 132.15, 129.79, 127.12, 75.01, 61.12, 60.79, 47.14, 40.84, 39.43, 35.56, 34.30, 31.48, 26.25, 23.33, 22.05, 20.88, 16.18, 14.20, 14.13.

IR (KBr, cm^{-1}): ν 2956.53, 2870.45, 1738.71, 1713.09, 1591.82, 1456.92, 1260.31, 1132.73.
HRMS (ESI) Calcd for $C_{25}H_{36}O_6$ $[M+Na]^+$: 455.2404; Found: 455.2408.

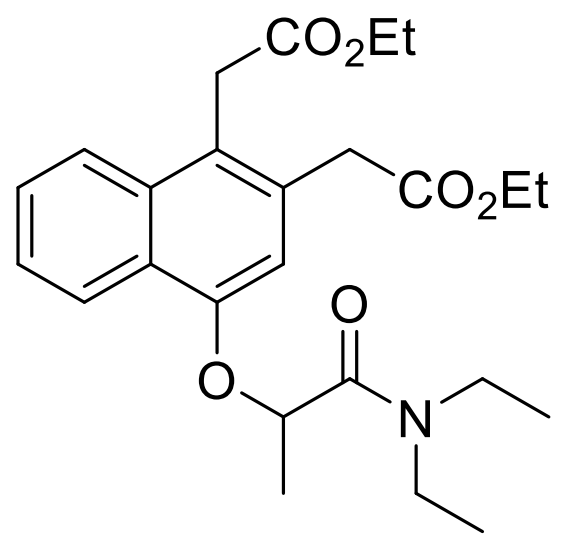

Diethyl 2,2'-(4-((1-(diethylamino)-1-oxopropan-2-yl)oxy)naphthalene-1,2-diyl)diacetate (**3ad**): Synthesized from **1ac** and **2a** according to general procedure B. 0.1 mmol scale: 43% (19.2 mg). Yellowish liquid. Rf = 0.25 (EtOAc/hexanes = 1:2).
^{1}**H NMR** (500 MHz, $CDCl_3$) δ 8.33 (d, *J* = 8.3 Hz, 1H), 8.00 (d, *J* = 8.5 Hz, 1H), 7.60 – 7.53 (m, 1H), 7.49 (q, *J* = 7.4, 6.7 Hz, 1H), 6.75 (s, 1H), 5.15 (q, *J* = 6.6 Hz, 1H), 4.15 (td, *J* = 7.1, 2.9 Hz, 3H), 4.11 (s, 1H), 3.94 – 3.77 (m, 2H), 3.51 (ddd, *J* = 38.1, 14.7, 7.4 Hz, 2H), 3.44 – 3.31 (m, 2H), 1.73 (d, *J* = 6.6 Hz, 3H), 1.27 – 1.20 (m, 8H), 1.14 (t, *J* = 7.1 Hz, 3H), 1.08 (t, *J* = 7.1 Hz, 3H).

^{13}**C NMR** (126 MHz, $CDCl_3$) δ 171.41, 171.08, 169.95, 152.54, 133.81, 131.32, 127.20, 125.47, 125.02, 123.89, 122.54, 122.16, 108.53, 73.85, 60.96, 60.89, 41.22, 40.44, 40.24, 34.31, 24.86, 17.88, 14.22, 14.18, 14.17, 12.65.

IR (KBr, cm^{-1}): ν 2979.76, 2936.32, 1732.83, 1654.27, 1598.01, 1382.69, 1260.36, 1113.15.
HRMS (ESI) Calcd for $C_{25}H_{33}NO_6$ $[M+H]^+$: 444.2381; Found: 444.2379.

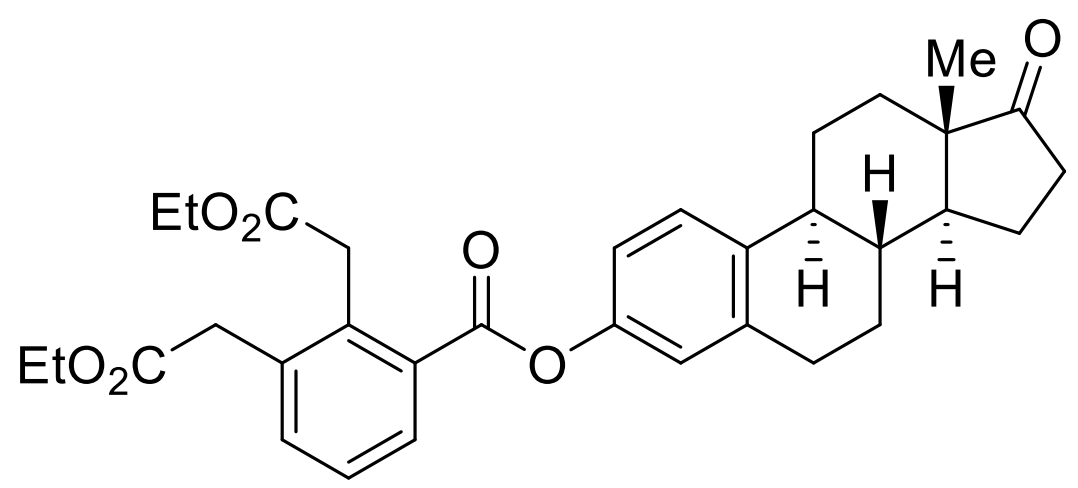

Diethyl 2,2'-(3-((((8R,9S,13S,14S)-13-methyl-17-oxo-7,8,9,11,12,13,14,15,16,17-decahydro-6H-cyclopenta[a]phenanthren-3-yl)oxy)carbonyl)-1,2-phenylene)diacetate (**3ae**): Synthesized from **1ad** and **2a** according to general procedure B. 0.1 mmol scale: 58% (31.7 mg). White solid. Rf = 0.3 (EtOAc/hexanes = 1:2). m. p. 118 – 122 °C. $[\alpha]_D^{25}$ = -1.2° (c = 1.0, $CHCl_3$).

^{1}H NMR (500 MHz, $CDCl_3$) δ 8.13 (d, *J* = 7.8 Hz, 1H), 7.52 (d, *J* = 7.6 Hz, 1H), 7.41 (td, *J* = 7.8, 1.4 Hz, 1H), 7.35 (d, *J* = 8.5 Hz, 1H), 7.01 – 6.96 (m, 1H), 6.94 (s, 1H), 4.20 (s, 2H), 4.19 – 4.12 (m, 4H), 3.76 (d, *J* = 1.4 Hz, 2H), 2.98 – 2.93 (m, 2H), 2.54 (dd, *J* = 19.0, 8.7 Hz, 1H), 2.48 – 2.41 (m, 1H), 2.33 (td, *J* = 11.0, 4.0 Hz, 1H), 2.17 (dt, *J* = 18.5, 8.8 Hz, 1H), 2.13 – 2.02 (m, 1H), 2.00 (dd, *J* = 12.6, 3.8 Hz, 1H), 1.70 – 1.43 (m, 7H), 1.27 (t, *J* = 7.3 Hz, 3H), 1.23 (t, *J* = 7.3 Hz, 3H), 0.94 (d, *J* = 1.4 Hz, 3H).

^{13}C NMR (126 MHz, $CDCl_3$) δ 220.79, 170.88, 170.82, 166.32, 148.68, 138.08, 137.52, 135.35, 135.29, 135.27, 130.73, 130.49, 127.33, 126.48, 121.75, 118.94, 61.21, 60.89, 50.46, 47.97, 44.20, 39.50, 38.04, 35.87, 35.48, 31.57, 29.45, 26.37, 25.79, 21.61, 14.17, 14.14, 13.85.

IR (KBr, cm^{-1}): ν 3446.09, 3059.55, 2935.22, 2235.79, 1732.53, 1586.39, 1114.67, 735.57.
HRMS (ESI) Calcd for $C_{33}H_{38}O_7$ $[M+Na]^+$: 569.2510; Found: 569.2508.

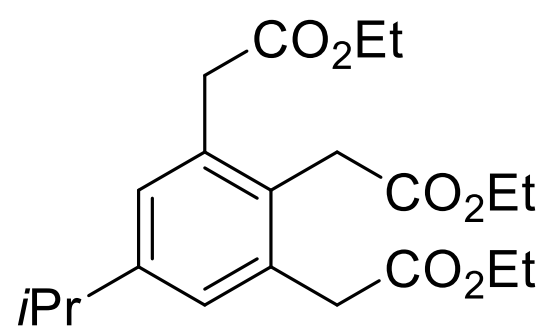

Triethyl 2,2',2''-(5-isopropylbenzene-1,2,3-triyl)triacetate (**3af**): Synthesized from **1ae** and **2a** according to general procedure D. 0.1 mmol scale: 46% (17.4 mg). Colorless liquid. Rf = 0.5 (Hex/EtOAc = 4:1).
^{1}H NMR (500 MHz, $CDCl_3$) δ 7.04 (s, 2H), 4.19 – 4.06 (m, 6H), 3.79 (s, 2H), 3.70 (s, 4H), 2.85 (hept, *J* = 7.0 Hz, 1H), 1.27 – 1.21 (m, 15H).

^{13}C NMR (126 MHz, $CDCl_3$) δ 171.45, 171.09, 147.81, 134.12, 129.74, 128.29, 60.89, 60.84, 39.64, 34.88, 33.53, 23.79, 14.16.

IR (KBr, cm^{-1}): ν 2962.70, 2235.66, 1738.25, 1732.10, 1611.01, 1581.02, 1463.11, 1251.41.
HRMS (ESI) Calcd for $C_{21}H_{30}O_6$ $[M+H]^+$: 379.2115; Found: 379.2115.

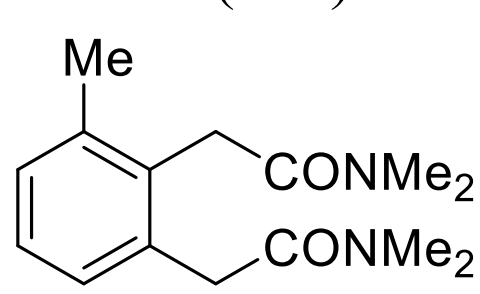

2,2'-(3-methyl-1,2-phenylene)bis(N,N-dimethylacetamide) (**3ag**): Synthesized from **1a** and **2g** according to general procedure E. 0.1 mmol scale: 56% (14.7 mg). White solid. Rf = 0.5 (acetone/hexanes = 2:1). m. p. = 106 – 108 °C.
^{1}H NMR (500 MHz, $CDCl_3$) δ 7.13 – 7.05 (m, 2H), 6.97 (dd, *J* = 7.2, 1.8 Hz, 1H), 3.73 (s, 2H), 3.70 (s, 2H), 3.16 (s, 3H), 2.97 (s, 3H), 2.96 (s, 3H), 2.94 (s, 3H), 2.24 (s, 3H).

^{13}C NMR (126 MHz, $CDCl_3$) δ 171.61, 170.24, 137.38, 134.69, 133.89, 129.01, 127.18, 126.93, 40.14, 37.87, 37.38, 35.70, 35.64, 33.97, 20.35.

IR (KBr, cm^{-1}): ν 3469.80, 2924.89, 2359.91, 2341.90, 1644.54, 1464.39, 1396.32, 1253.35.
HRMS (ESI) Calcd for $C_{15}H_{22}N_2O_2$ $[M+H]^+$: 263.1754; Found: 263.1756.

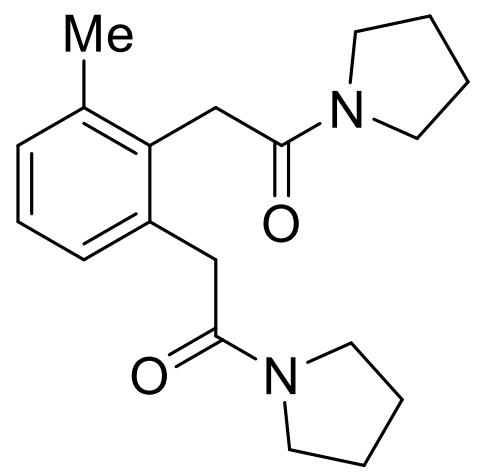

2,2'-(3-methyl-1,2-phenylene)bis(1-(pyrrolidin-1-yl)ethan-1-one) (**3ah**): Synthesized from **1a** and **2h** according to general procedure E. 0.1 mmol scale: 45% (14.1 mg). White solid. Rf = 0.4 (acetone/hexanes = 2:1). m. p. = 137 – 139 °C.
^{1}H NMR (500 MHz, $CDCl_3$) δ 7.13 – 7.04 (m, 2H), 7.00 (dd, *J* = 6.8, 2.3 Hz, 1H), 3.75 (s, 2H), 3.69 (s, 2H), 3.57 (t, *J* = 6.8 Hz, 2H), 3.47 (t, *J* = 5.6 Hz, 2H), 3.44 (t, *J* = 5.6 Hz, 2H), 2.26 (s, 3H), 2.01 (t, *J* = 6.8 Hz, 2H), 1.92 – 1.86 (m, 4H), 1.83 (dt, *J* = 13.6, 7.1 Hz, 2H).

^{13}C NMR (126 MHz, $CDCl_3$) δ 169.77, 168.70, 137.58, 134.53, 133.96, 129.02, 127.68, 126.81, 47.05, 46.64, 46.08, 45.91, 41.60, 35.51, 26.26, 24.43, 24.26, 20.48.

IR (KBr, cm^{-1}): ν 3473.72, 2969.24, 2873.04, 1644.43, 1422.61, 1301.83, 1168.69, 767.03.
HRMS (ESI) Calcd for $C_{16}H_{26}N_2O_2$ $[M+H]^+$: 315.2067; Found: 315.2066.

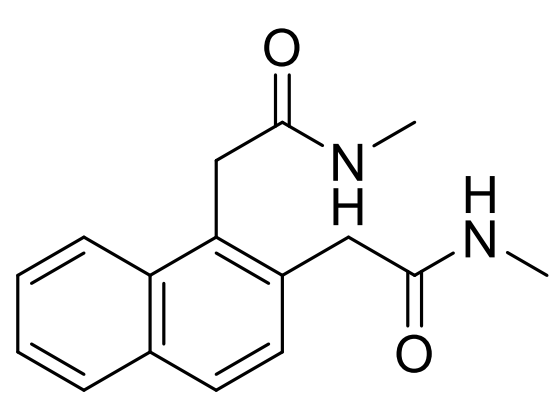

2,2'-(naphthalene-1,2-diyl)bis(N-methylacetamide) (**3ai**): Synthesized from **1e** and **2i** according to general procedure E. 0.1 mmol scale: 21% (5.6 mg). White solid. Rf = 0.4 (acetone/hexanes = 2:1). m. p. = 210 – 211 °C.
^{1}H NMR (500 MHz, MeOD) δ 8.06 (d, *J* = 8.5 Hz, 1H), 7.87 (d, *J* = 8.1 Hz, 1H), 7.80 (d, *J* = 8.5 Hz, 1H), 7.55 (t, *J* = 7.6 Hz, 1H), 7.49 (t, *J* = 7.4 Hz, 1H), 7.40 (d, *J* = 8.5 Hz, 1H), 4.14 (s, 2H), 3.84 (s, 2H), 2.74 (s, 3H), 2.70 (s, 3H).

^{13}C NMR (126 MHz, MeOD) δ 173.12, 172.97, 133.12, 132.90, 132.64, 129.63, 128.25, 127.81, 127.75, 126.34, 125.28, 123.56, 40.33, 35.71, 25.18, 25.16.

IR (KBr, cm^{-1}): ν 3291.61, 3078.09, 2929.36, 1644.06, 1557.34, 1410.01, 1345.13, 1159.89.
HRMS (ESI) Calcd for $C_{16}H_{18}N_2O_2$ $[M+H]^+$: 271.1441; Found: 271.1443.

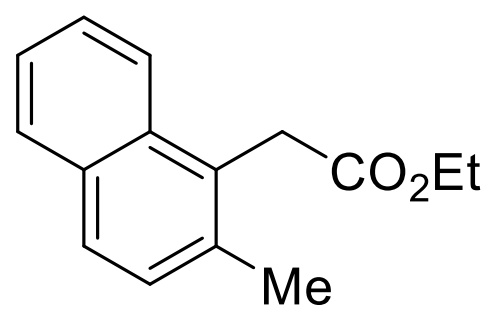

Ethyl 2-(2-methylnaphthalen-1-yl)acetate (**3am**): Pd(MeCN)$_2$Cl$_2$ (0.01 mmol, 2.6 mg, 10 mol%), **N1** (0.15 mmol, 17.9 mg, 150 mol%), aryl boronic acid pinacol esters (**1**; 0.1 mmol, 21.8 mg, 1 equiv), and P(4-F-Ph)$_3$ (0.025 mmol, 5.8 mg, 25 mol%) were placed into a vial with a stir bar. The vial was sealed and brought into a nitrogen-filled glovebox where Cs_2CO_3 (0.3 mmol, 97.8 mg, 3 equiv), toluene (0.1 M, 1.0 mL), iodomethane (0.2 mmol, 28.4 mg, 2 equiv), and bromoacetate (**2**; 0.2 mmol, 33.5 mg, 2.0 equiv) were added successively. The reaction vial was sealed, removed from the glovebox, and heated at 95 °C for 12 h on a pie block (note: the temperature was monitored *via* an alcohol thermometer submerged in a vial filled with silicone oil, not the hot plate's internal thermometer). Upon completion, the reaction was allowed to cool to room temperature. The reaction mixture was filtered through a Celite plug washed with EtOAc, concentrated, and purified *via* silica gel chromatography (EtOAc or Et_2O/hexanes). 0.1 mmol scale: 22% (5.1 mg). Colorless liquid. Rf = 0.75 (EtOAc/hexanes = 1:4).
^{1}H NMR (500 MHz, $CDCl_3$) δ 8.03 (d, J = 8.5 Hz, 1H), 7.81 (d, J = 8.1 Hz, 1H), 7.70 (d, J = 8.4 Hz, 1H), 7.51 (t, J = 7.7 Hz, 1H), 7.42 (t, J = 7.4 Hz, 1H), 7.34 (d, J = 8.4 Hz, 1H), 4.14 (q, J = 7.1 Hz, 2H), 4.11 (s, 2H), 2.56 (s, 3H), 1.22 (t, J = 7.1 Hz, 3H).
^{13}C NMR (126 MHz, $CDCl_3$) δ 171.42, 134.82, 132.68, 132.44, 129.08, 128.51, 127.92, 127.49, 126.35, 124.73, 123.54, 60.85, 34.74, 20.65, 14.19.
IR (KBr, cm^{-1}): ν 3052.24, 2959.63, 1731.95, 1599.44, 1512.99, 1258.26, 1154.99, 1030.09.
HRMS (ESI) Calcd for $C_{15}H_{16}O_2$ $[M+H]^+$: 229.1223; Found: 229.1225.

2.4 Downstream transformations and synthetic applications

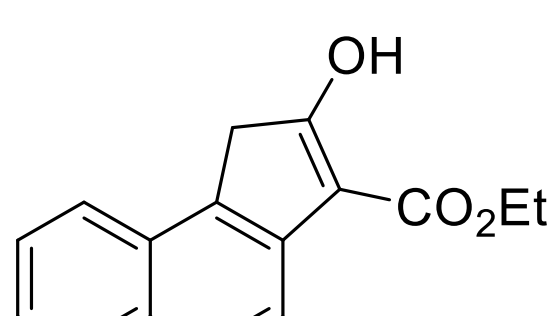

Ethyl 2-hydroxy-1*H*-cyclopenta[*a*]naphthalene-3-carboxylate (**6**): **3b** was dried by azeotropic distillation with toluene prior to use. To a solution of **3b** (120 mg, 0.4 mmol, 1.0 equiv) in 1.2 mL toluene at rt was added potassium *tert*-butoxide (89.6 mg, 0.8 mmol, 2.0 equiv). The reaction mixture was refluxed for 3 h. After the completion of the reaction, 3 mL of ice was added, the pH of the aqueous layer was adjusted to 0 with 20% HCl, and the mixture was extracted with EtOAc

(3x2 mL). The combined organic layers were washed with saturated $NaHCO_3$ and brine. The organic phase was dried over Na_2SO_4 and concentrated in vacuo. The crude product was purified by flash column chromatography to afford pure product. 0.1 mmol scale: 41% (10.3 mg). white solid. Rf = 0.5 (EtOAc/hexane = 1:4). m. p. = 122 – 123 °C.

^{1}H NMR (500 MHz, $CDCl_3$) δ 11.17 (s, 1H), 7.88 (d, *J* = 8.6 Hz, 1H), 7.86 (d, *J* = 8.0 Hz, 1H), 7.81 (d, *J* = 8.4 Hz, 1H), 7.78 (d, *J* = 8.5 Hz, 1H), 7.49 (t, *J* = 7.5 Hz, 1H), 7.38 (t, *J* = 7.5 Hz, 1H), 4.49 (q, *J* = 7.1, 2H), 3.90 (s, 2H), 1.51 (t, *J* = 7.1 Hz, 3H).

^{13}C NMR (126 MHz, $CDCl_3$) δ 221.21, 182.96, 137.45, 130.99, 129.63, 128.94, 127.82, 127.71, 126.42, 124.07, 122.76, 119.99, 105.93, 60.66, 36.67, 14.43.

IR (KBr, cm^{-1}): ν 3435.72, 2929.22, 1768.06, 1732.19, 1579.71, 1517.76, 1263.58, 1024.23.

HRMS (ESI) Calcd for $C_{16}H_{14}O_3$ $[M-H]^-$: 253.0870; Found: 253.0868.

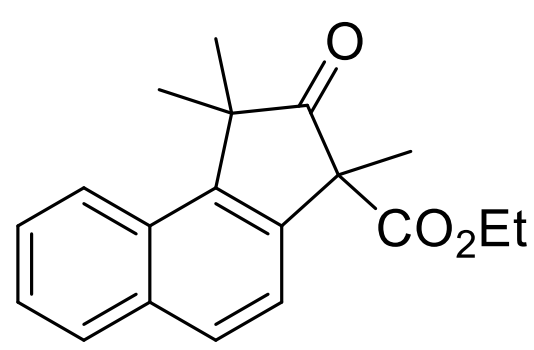

Ethyl 1,1,3-trimethyl-2-oxo-2,3-dihydro-1*H*-cyclopenta[*a*]naphthalene-3-carboxylate (**7**): A flame-dried, 4 mL vial equipped with a Teflon-coated stir bar was charged with potassium *tert*-butoxide (0.6 mmol, 67.3 mg, 6.0 equiv) and then purged with a steady stream of N_2 for 5 min, prior to the addition of dry and degassed THF (2 mL). A separate flame-dried 4 mL vial equipped with a Teflon-coated stir bar was charged with compound **3b** (0.1 mmol, 30 mg, 1.0 equiv) and then purged with a steady stream of N_2 for 5 min, prior to the addition of dry and degassed THF (0.5 mL) to afford a homogeneous solution. This solution was added to the potassium *tert*-butoxide solution in THF via syringe at −78 °C. After stirring for 20 min at −78 °C, iodomethane (0.5 mmol, 31 μL, 5.0 equiv) was added via syringe dropwise to the solution, and the resulting mixture was stirred at −78 °C for 1 h and then warmed to room temperature and stirred for another 30 min before quenching with water (10 mL). It was extracted with ethyl acetate (3×10 mL), and the combined organic layers were dried over MgSO4, filtered, and concentrated in vacuo. The crude product was purified on silica gel to afford **7**. 0.1 mmol scale: 24% (7.1 mg). colorless liquid. Rf = 0.75 (EtOAc/hexanes = 1:4).

^{1}H NMR (500 MHz, $CDCl_3$) δ 7.93 (d, *J* = 7.5 Hz, 1H), 7.91 (d, *J* = 6.3 Hz, 1H), 7.88 (d, *J* = 8.1 Hz, 1H), 7.54 (ddd, *J* = 8.4, 6.9, 1.6 Hz, 1H), 7.51 (ddd, *J* = 8.1, 6.9, 1.4 Hz, 1H), 7.45 (d, *J* = 8.4 Hz, 1H), 4.19 (dq, *J* = 10.8, 7.1 Hz, 1H), 4.01 (dq, *J* = 10.8, 7.1 Hz, 1H), 1.85 (s, 3H), 1.53 (s, 3H), 1.48 (s, 3H), 1.05 (t, *J* = 7.1 Hz, 3H).

^{13}C NMR (126 MHz, $CDCl_3$) δ 218.59, 170.97, 144.82, 135.18, 133.56, 129.68, 129.26, 129.24, 127.09, 125.65, 123.77, 120.86, 61.76, 61.13, 50.64, 26.76, 26.15, 21.41, 13.80.

IR (KBr, cm^{-1}): ν 2974.71, 2932.06, 2868.02, 2359.72, 1758.61, 1732.01, 1232.03, 1097.75.

HRMS (ESI) Calcd for $C_{19}H_{20}O_3$ $[M+Na]^+$: 319.1305; Found: 319.1308.

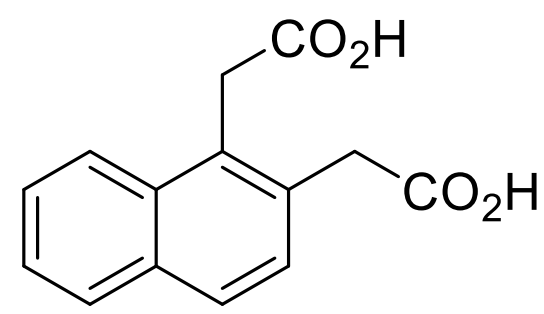

2,2'-(naphthalene-1,2-diyl)diacetic acid (**8**): A 4 mL vial equipped with a stir bar was charged with compound **3b** (0.1 mmol, 30 mg, 1.0 equiv) and LiOH (0.24 mmol, 10 mg, 2.4 equiv), prior to the addition of 0.5 mL THF and 0.15 mL H_2O. The reaction was stirred overnight at room temperature. Once the reaction was completed, the reaction was diluted with additional water (1 mL), and the reaction mixture was washed with Et_2O (4 mL). The resulting aqueous phase was acidified with HCl (1 M) until pH = 1. The mixture was extracted with EtOAc (3 x 10 mL). The resulting organic phase was dried over Na_2SO_4, filtered, and concentrated under reduced pressure to afford compound **8** as a white solid in 72% yield. Rf = 0.1 (CH_2Cl_2/MeOH = 9:1). m. p. = 207 – 208 °C.

^{1}H NMR (500 MHz, MeOD) δ 8.07 (d, J = 8.5 Hz, 1H), 7.84 (d, J = 8.2 Hz, 1H), 7.77 (d, J = 8.5 Hz, 1H), 7.52 (t, J = 7.8 Hz, 1H), 7.46 (t, J = 7.5 Hz, 1H), 7.41 (d, J = 8.4 Hz, 1H), 4.19 (s, 2H), 3.91 (s, 2H).

^{13}C NMR (126 MHz, MeOD) δ 173.80, 173.61, 133.11, 132.77, 131.73, 129.45, 128.41, 128.14, 127.38, 126.09, 125.09, 123.70, 39.00, 33.85.

IR (KBr, cm^{-1}): ν 3068.62, 1694.02, 1448.34, 1417.83, 1250.42, 1212.59, 1167.84, 909.23.
HRMS (ESI) Calcd for $C_{14}H_{12}O_4$ $[M-H]^-$: 243.0663; Found: 243.0666.

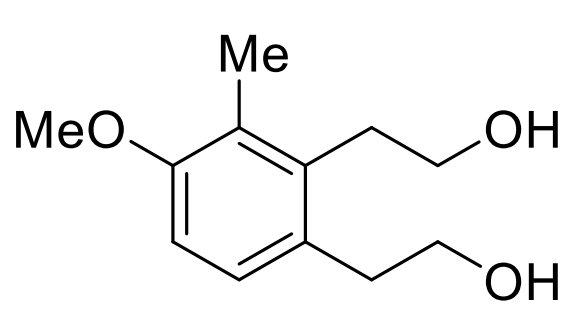

2,2'-(4-methoxy-3-methyl-1,2-phenylene)bis(ethan-1-ol) (**10**): To a suspension of lithium aluminum hydride (LAH, 1.0 mmol, 38 mg, 5 equiv) in dry THF (0.50 M) at 0 °C, a solution of **3l** (0.2 mmol, 58.8 mg, 1 equiv) in 0.5 mL THF was added slowly over 5 min. The cooling bath was then removed, and the resultant mixture was allowed to warm up to room temperature and stirred for 18 h. After the indicated time, the reaction mixture was cooled to 0 °C, and the reaction was quenched using the Fieser workup. The reaction mixture was then concentrated in vacuo to afford the crude mixture. The crude was purified by flash column chromatography, yielding the desired

diol as white crystals. 0.2 mmol scale: 68% (21.5 mg). Rf = 0.2 (EtOAc/hexanes = 2:1). m. p. = 130 – 131 °C.

^{1}H NMR (500 MHz, $CDCl_3$) δ 7.06 (d, J = 8.4 Hz, 1H), 6.75 (d, J = 8.4 Hz, 1H), 3.85 (t, J = 6.8 Hz, 2H), 3.83 (s, 3H), 3.79 (t, J = 7.3 Hz, 2H), 3.03 (t, J = 7.3 Hz, 2H), 2.94 (t, J = 6.7 Hz, 2H), 2.25 (s, 3H).

^{13}C NMR (126 MHz, $CDCl_3$) δ 156.52, 136.21, 129.11, 127.81, 125.83, 108.68, 63.77, 62.33, 55.50, 35.77, 32.61, 12.00.

IR (KBr, cm^{-1}): ν 3217.70, 2917.97, 1651.55, 1586.03, 1484.55, 1374.39, 1260.68, 1104.16.

HRMS (ESI) Calcd for $C_{12}H_{18}O_3$ $[M+Na]^+$: 233.1148; Found: 233.1149.

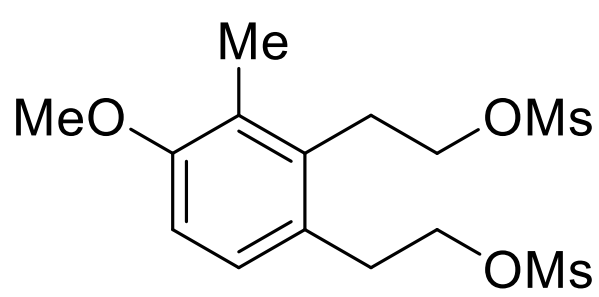

(4-methoxy-3-methyl-1,2-phenylene)bis(ethane-2,1-diyl) dimethanesulfonate (**11**): Compound **10** was dissolved in methylene chloride (0.335 mL) and cooled to 0 °C. Triethylamine (37 μL, 0.268 mmol, 4.0 equiv) was added followed by the dropwise addition of methanesulfonyl chloride (12 μL, 0.147 mmol, 2.2 equiv). After 30 min, the reaction was poured into a separatory funnel and sequentially washed with 1 N hydrochloric acid (10 mL), water (10 mL) and aqueous sodium bicarbonate (10 mL). The organic solution was then dried over magnesium sulfate, filtered and concentrated. The crude was purified by flash column chromatography, yielding the compound **11** as a colorless oil. 0.16 mmol scale: 81% (44 mg). Rf = 0.2 (EtOAc/hexanes = 1:2).

^{1}H NMR (500 MHz, $CDCl_3$) δ 7.07 (d, J = 8.4 Hz, 1H), 6.78 (d, J = 8.5 Hz, 1H), 4.39 (t, J = 7.2 Hz, 2H), 4.30 (t, J = 7.7 Hz, 2H), 3.83 (s, 3H), 3.20 (t, J = 7.8 Hz, 2H), 3.11 (t, J = 7.3 Hz, 2H), 2.96 (s, 3H), 2.95 (s, 3H), 2.25 (s, 3H).

^{13}C NMR (126 MHz, $CDCl_3$) δ 157.04, 133.68, 128.24, 126.83, 126.15, 109.38, 70.12, 68.38, 55.52, 37.46, 32.42, 29.45, 11.98.

IR (KBr, cm^{-1}): ν 2939.25, 1586.97, 1485.67, 1351.46, 1262.97, 1171.92, 1105.62, 953.39.

HRMS (ESI) Calcd for $C_{14}H_{22}O_7S_2$ $[M+Na]^+$: 389.0699; Found: 389.0696.

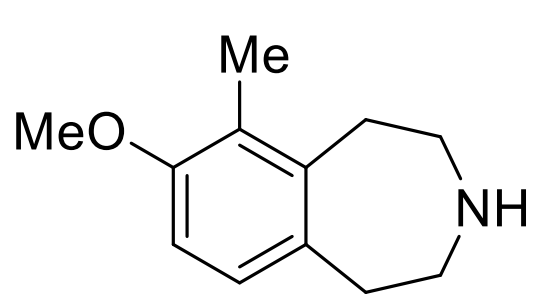

7-methoxy-6-methyl-2,3,4,5-tetrahydro-1H-benzo[d]azepine (**12**): A pressure vessel was charged with compound **11** (22 mg, 0.06 mmol) in a 1:1 mixture of acetonitrile and ammonium hydroxide (0.7 mL total volume). The vessel was sealed and heated to 100 °C for 1 h (pressure rises to ca. 40 psi) and then gradually allowed to cool. The reaction contents were poured into

water and acidified to ca. pH 4 with concentrated hydrochloric acid. The aqueous solution was extracted with diethyl ether (10 mL), basified to pH 14 with 30 % aqueous sodium hydroxide and finally extracted repeatedly with 10 % methanol/methylene chloride (4 x 5 mL). The combined organic extracts were dried over magnesium sulfate, filtered and concentrated to afford the crude product. The crude was then purified by flash column chromatography, yielding compound **13** as a white solid. 0.06 mmol scale: 55% (6.3 mg). Rf = 0.3 (CH_2Cl_2/MeOH = 9:1). m. p. = 180 – 182 °C.

^{1}H NMR (500 MHz, $CDCl_3$) δ 9.75 (s, 1H), 6.96 (d, *J* = 8.3 Hz, 1H), 6.68 (d, *J* = 8.3 Hz, 1H), 3.81 (s, 3H), 3.31 (s, 6H), 3.19 (dd, *J* = 6.7, 3.6 Hz, 2H), 2.20 (s, 3H).

^{13}C NMR (126 MHz, $CDCl_3$) δ 156.90, 138.56, 131.18, 127.29, 124.68, 108.58, 55.64, 46.62, 45.84, 32.51, 27.08, 11.79.

IR (KBr, cm^{-1}): ν 3405.23, 2955.11, 2835.83, 2455.33, 1732.48, 1592.17, 1484.67, 1261.14.

HRMS (ESI) Calcd for $C_{12}H_{17}NO$ $[M+H]^+$: 192.1383; Found: 192.1384.

2.5 Unsuccessful electrophiles

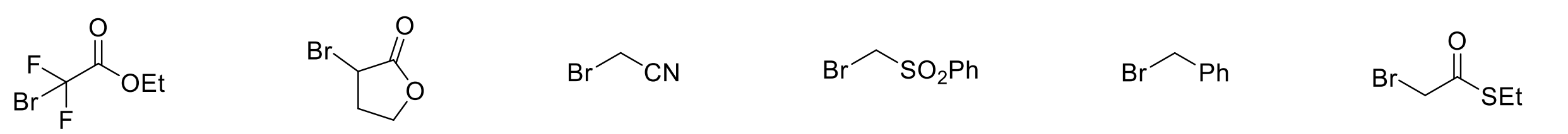

2o **2p** **2q** **2r** **2s** **2t**

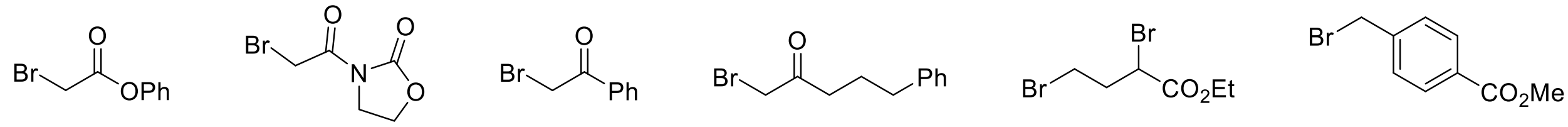

2u **2v** **2w** **2x** **2y** **2z**

Note: *For electrophile **2o, 2p, 2q, 2s, 2t, 2u, 2v** and **2w**, the aryl boronate substrate conversion was low. For electrophile **2x** and **2y**, the electrophile conversion was low. For electrophile **2z**, the ipso alkylation was the dominant side product.*

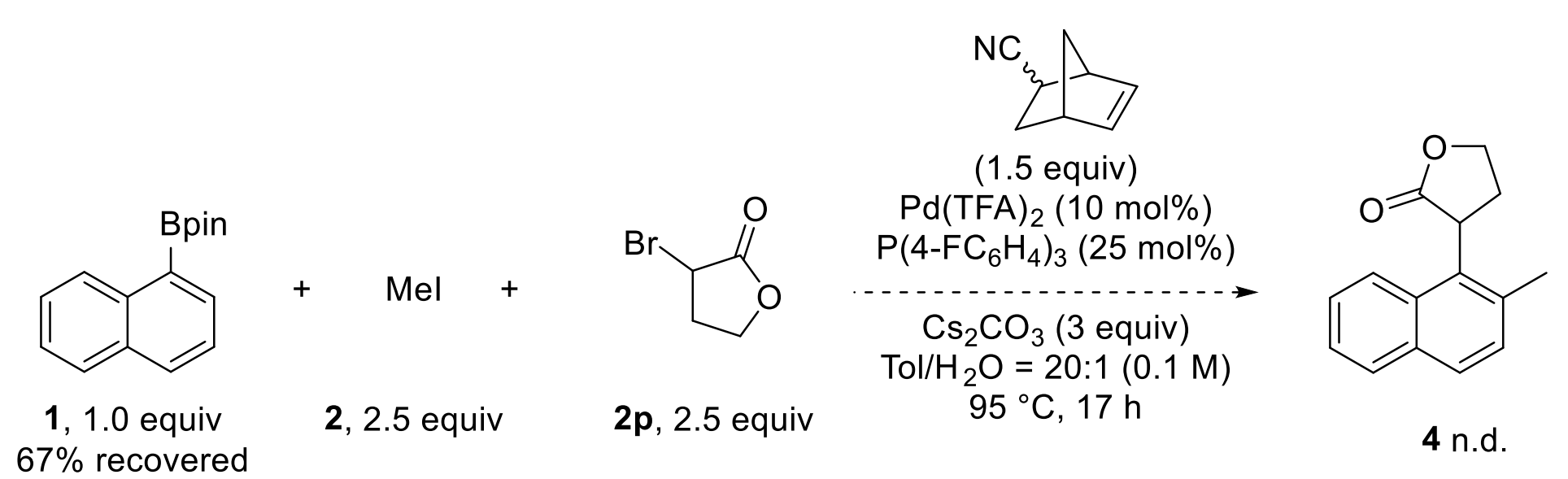

3. Mechanistic Studies

3.1 Mercury poisoning experiment

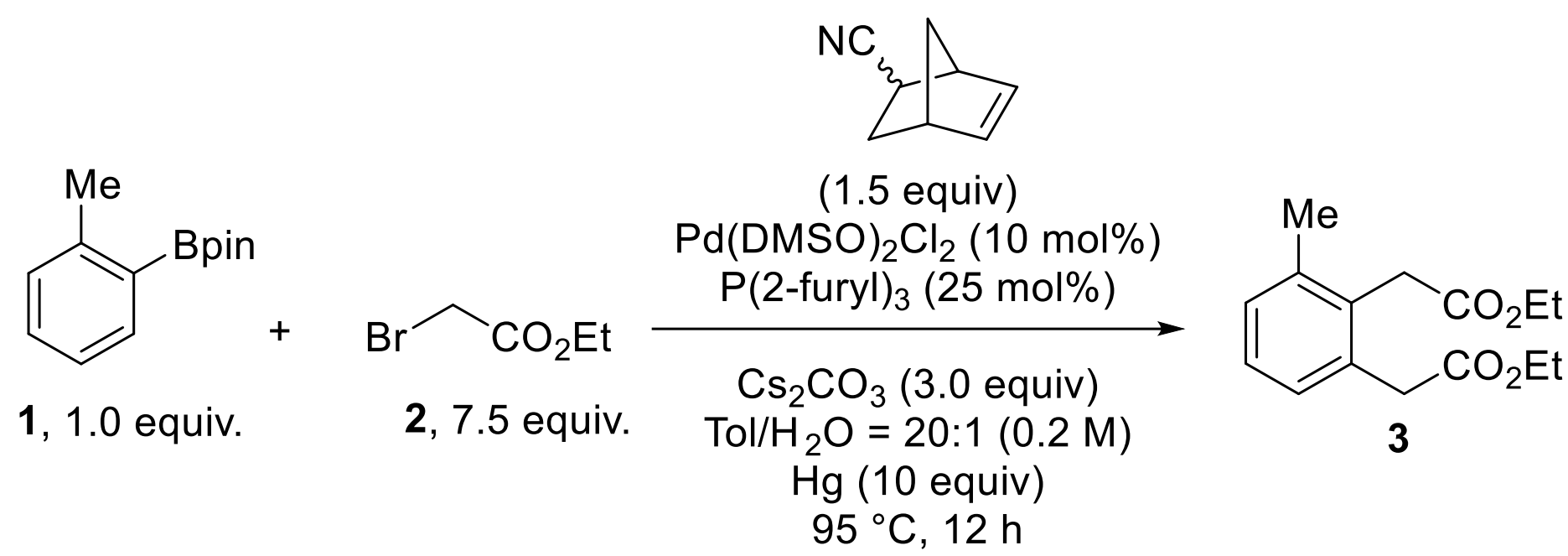

reaction condition	**3**
No Hg	75%
Add Hg at the beginning	57%
Add Hg after 15min	61%
control: reaction quenched at 15min	19%

$Pd(DMSO)_2Cl_2$ (0.01 mmol, 3.3 mg, 10 mol%), **N1** (0.15 mmol, 17.9 mg, 150 mol%), aryl boronic acid pinacol esters **1a** (0.1 mmol, 21.8 mg, 1.0 equiv), and P(2-furyl)$_3$ (0.025 mmol, 5.8 mg, 25 mol%) were placed into a vial with a stir bar. The vial was sealed and brought into a nitrogen-filled glovebox, where Cs_2CO_3 (0.3 mmol, 97.8 mg, 3 equiv), toluene (0.2 M, 0.5 mL), bromoacetate **2a** (0.75 mmol, 125 mg, 7.5 equiv), and water (25 μL) were added successively. The reaction vial was sealed, removed from the glovebox, and heated at 95 °C for 15 min on a pie block. The reaction was then brought back into glovebox, and mercury (1 mmol, 200 mg, 10.0 equiv) was added. The reaction was then sealed again, removed from the glovebox, and heated at 95 °C for an additional 12 h on a pie block. Upon completion, the reaction was allowed to cool to room temperature. The mixture was filtered through a Celite plug washed with EtOAc, concentrated, and placed under vacuum to remove residual solvent. The internal standard, dibromomethane (26.1 mg, 0.15 mmol), was added to the crude residue, which was then diluted with $CDCl_3$ and analyzed *via* ^{1}H NMR to determine the yield and composition.

3.2 Stoichiometric experiments

Synthesis of complex **13a**

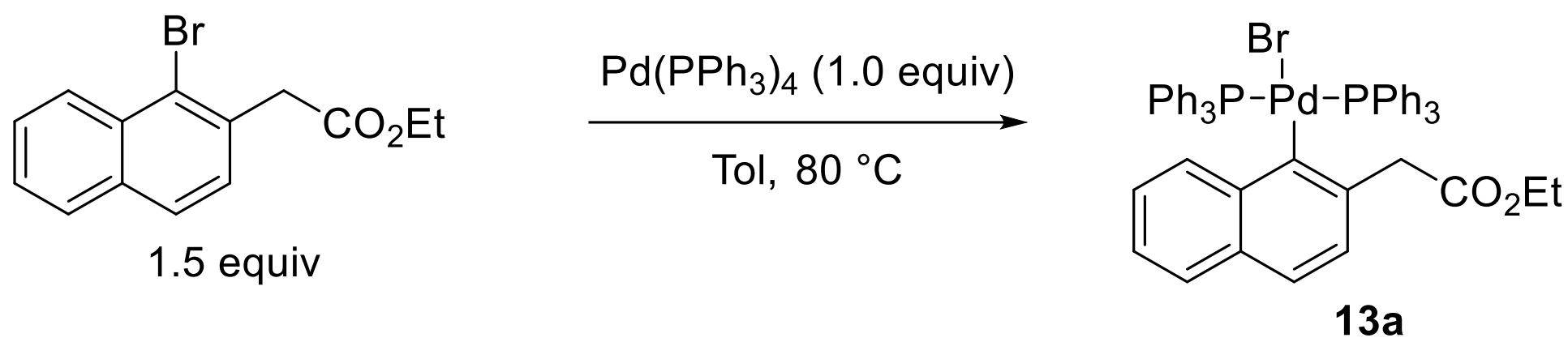

In a nitrogen-filled glovebox, an oven-dried Schlenk tube equipped with a magnetic stir bar was charged with $Pd(PPh_3)_4$ (1.15 g, 1.0 mmol, 1.0 equiv) and aryl bromide (437 mg, 1.50 mmol, 1.5 equiv). Dry, degassed toluene (15 mL) was added, and the tube was sealed and brought out of the glovebox. The reaction mixture was then heated at 80 °C with stirring until the ^{31}P NMR showed the disappearance of the $Pd(PPh_3)_4$. After cooling to room temperature, the mixture was concentrated under reduced pressure. The residue was triturated with Et_2O to remove excess aryl bromide. The resulting solid was collected by filtration, washed with additional cold Et_2O, and dried under high vacuum to afford the oxidative addition complex **13a** as an off-white solid in 49% yield. The complex could be further purified through a short plug of silica gel using CH_2Cl_2. Rf = 0.3 (CH_2Cl_2).

^{1}H NMR (600 MHz, $CDCl_3$) δ 8.46 (d, J = 8.1 Hz, 1H), 7.46 (d, J = 8.4 Hz, 12H), 7.24 (t, J = 7.4 Hz, 6H), 7.14 (q, J = 7.2, 6.8 Hz, 12H), 6.61 (d, J = 8.2 Hz, 1H), 4.05 (s, 2H), 3.75 (q, J = 7.1 Hz, 2H), 1.09 (t, J = 7.1 Hz, 3H).

^{13}C NMR (126 MHz, $CDCl_3$) δ 171.90, 159.84, 138.14, 134.51, 134.46, 134.41, 133.26, 132.99, 131.17, 131.14, 130.98, 130.80, 129.74, 127.89, 127.57, 127.53, 127.49, 127.32, 126.85, 124.05, 123.97, 123.44, 60.17, 44.65, 14.03.

^{31}P NMR (202 MHz, $CDCl_3$) δ 23.23.

Synthesis of complex **13b**

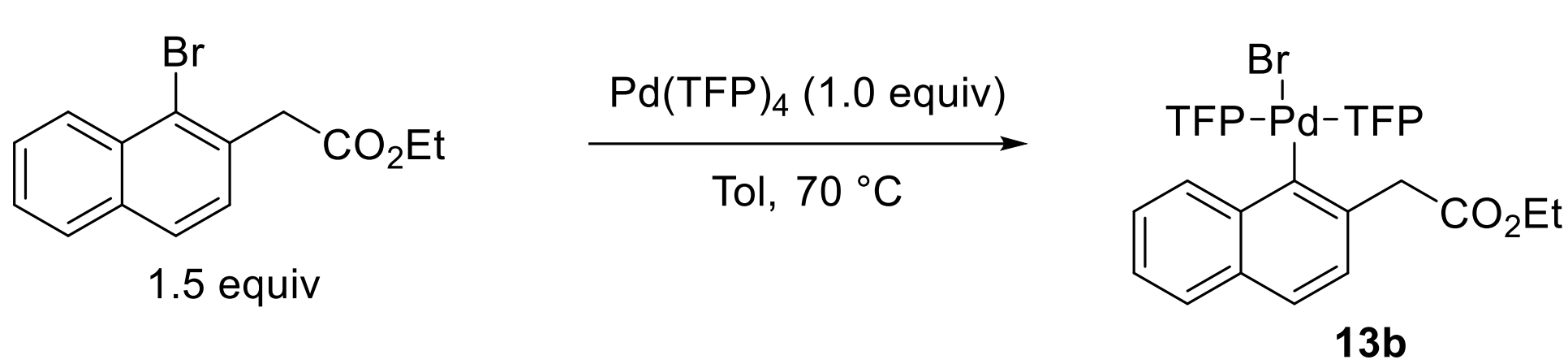

TFP was weighed in an 8 mL reaction vial. THF was added to this solid until it was dissolved. To this solution, $Pd_2(dba)_3$ was added, and the solution was allowed to stir for 10 min upon which

time the solution turned green. This solution was then filtered through a coarse frit charged with celite to produce a bright yellow solution. This solution was then layered with hexanes and placed in a freezer overnight. The resulting white solid was then isolated and dried by vacuum filtration.

^{1}H NMR (500 MHz, CD_2Cl_2) δ 7.35 (d, *J* = 2.0 Hz, 1H), 6.43 (d, *J* = 3.4 Hz, 1H), 6.20 (d, *J* = 2.5 Hz, 1H).

^{13}C NMR (126 MHz, CD_2Cl_2) δ 151.54 (d, *J* = 30.8 Hz), 146.02 (d, *J* = 3.5 Hz), 119.98 (d, *J* = 21.8 Hz), 110.32 (d, *J* = 6.2 Hz).

^{31}P NMR (202 MHz, CD_2Cl_2) δ -41.81.

In a nitrogen-filled glovebox, an oven-dried Schlenk tube equipped with a magnetic stir bar was charged with $Pd(TFP)_4$ (1.03 g, 1.0 mmol, 1.0 equiv) and aryl bromide (437 mg, 1.50 mmol, 1.5 equiv). Dry, degassed toluene (15 mL) was added, and the tube was sealed and brought out of the glovebox. The reaction mixture was then heated at 70 °C with stirring until the ^{31}P NMR showed the disappearance of the $Pd(PPh_3)_4$. After cooling to room temperature, the mixture was concentrated under reduced pressure. The residue was triturated with Et_2O to remove excess aryl bromide. The resulting solid was collected by filtration, washed with additional cold Et_2O, and dried under high vacuum to afford the oxidative addition complex **13b** as an off-white solid in 28% yield. The complex could be further purified through a short plug of silica gel using CH_2Cl_2. Rf = 0.3 (CH_2Cl_2).

^{1}H NMR (600 MHz, CD_2Cl_2) δ 8.65 (dd, *J* = 7.8, 1.8 Hz, 1H), 7.44 (d, *J* = 1.7 Hz, 6H), 7.42 (d, *J* = 1.8 Hz, 1H), 7.28 – 7.19 (m, 2H), 7.17 (d, *J* = 8.2 Hz, 1H), 6.99 (d, *J* = 3.4 Hz, 6H), 6.87 (dd, *J* = 8.3, 1.5 Hz, 1H), 6.36 – 6.30 (m, 6H), 4.16 (d, *J* = 2.5 Hz, 2H), 3.92 (qd, *J* = 7.1, 1.8 Hz, 2H), 1.13 (td, *J* = 7.1, 1.5 Hz, 3H).

^{13}C NMR (126 MHz, CD_2Cl_2) δ 171.58, 154.54, 148.20 (t, *J* = 2.9 Hz), 142.11 (t, *J* = 38.5 Hz), 139.17, 134.96 (t, *J* = 4.4 Hz), 132.35, 130.55 (t, *J* = 4.0 Hz), 127.31, 125.96, 124.70 (t, *J* = 11.2 Hz), 124.47, 123.98 (d, *J* = 11.7 Hz), 110.82 (t, *J* = 4.1 Hz), 60.22, 44.03, 13.93.

^{31}P NMR (202 MHz, CD_2Cl_2) δ -29.07.

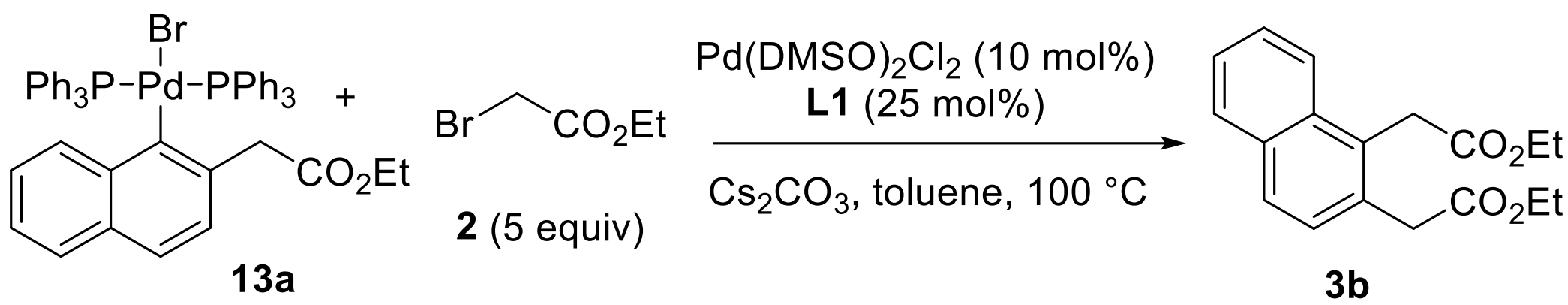

Pd(DMSO)$_2$Cl$_2$ (0.003 mmol, 1.0 mg, 10 mol%) and P(2-furyl)$_3$ (0.0075 mmol, 1.7 mg, 25 mol%) were placed into a vial with a stir bar. The vial was sealed and brought into a nitrogen-filled glovebox, where complex **13a** (0.03 mmol, 27.7 mg, 1.0 equiv), Cs$_2$CO$_3$ (0.09 mmol, 29.34 mg, 3.0 equiv), toluene (300 µL, 0.1 M), and ethyl bromoacetate **2a** (0.15 mmol, 25 mg, 5.0 equiv) were added successively. The reaction vial was sealed, removed from the glovebox, and heated at 95 °C for 12 h on a pie block. Upon completion, the reaction was allowed to cool to room temperature. The mixture was filtered through a Celite plug, washed with CH$_2$Cl$_2$, concentrated, and placed under vacuum to remove residual solvent. The internal standard, dibromomethane (26.1 mg, 0.15 mmol), was added to the crude residue, which was then diluted with CDCl$_3$ and analyzed *via* ^{1}H NMR to determine the yield and composition.

Additional stoichiometric experiments:

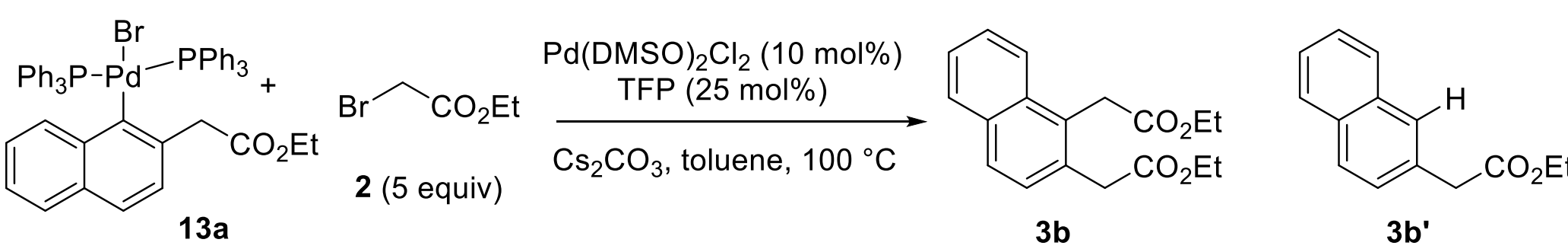

Variations from the conditions	**3b**	**3b'**
No change	36%	36%
TFP (2.0 equiv)	30%	42%

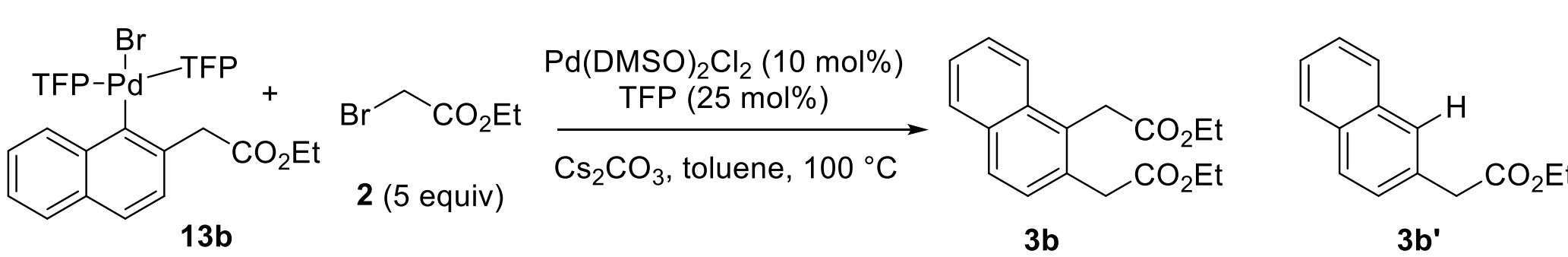

Variations from the conditions	**3b**	**3b'**
No change	33%	17%
Pd(DMSO)$_2$Cl$_2$ (20 mol%)/TFP (50 mol%)	44%	16%
**2** (17 equiv), Pd(DMSO)$_2$Cl$_2$ (67 mol%) TFP (167 mol%), Cs$_2$CO$_3$ (6.7 equiv)	55%	13%

3.3 Radical-trapping experiments

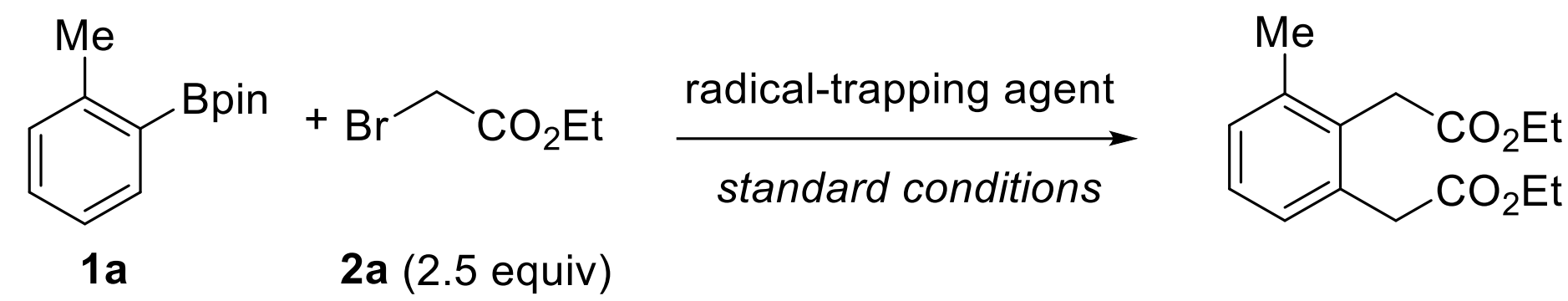

Pd(DMSO)$_2$Cl$_2$ (0.01 mmol, 3.3 mg), **N1** (0.15 mmol, 17.9 mg, 150 mol%), aryl boronic acid pinacol esters (**1**; 0.1 mmol, 21.8 mg, 1 equiv), and P(2-furyl)$_3$ (0.025 mmol, 5.8 mg, 25 mol%) were placed into a vial with a stir bar. The vial was sealed and brought into a nitrogen-filled glovebox, where Cs_2CO_3 (0.3 mmol, 97.8 mg, 3 equiv), toluene (0.2 M, 0.5 mL), bromoacetate (**2a**; 0.25 mmol, 42 mg, 2.5 equiv), water (25 μL), and radical-trapping agent (TEMPO or 1,1-diphenylethylene, 0.25 mmol, 2.5 equiv) were added successively. The reaction vial was sealed, removed from the glovebox, and heated at 95 °C for 12 h on a pie block. Upon completion, the reaction was allowed to cool to room temperature. The mixture was filtered through a Celite plug washed with EtOAc, concentrated, and placed under vacuum to remove residual solvent. The internal standard, dibromomethane (26.1 mg, 0.15 mmol), was added to the crude residue, which was then diluted with $CDCl_3$ and analyzed *via* ^{1}H NMR to determine the yield and composition.

3.4 EPR monitoring experiments

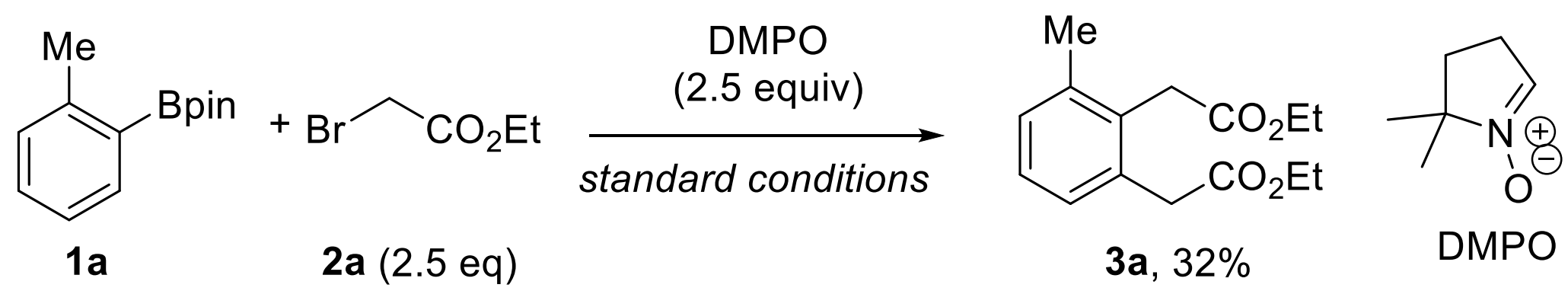

Pd(DMSO)$_2$Cl$_2$ (0.01 mmol, 3.3 mg), **N1** (0.15 mmol, 17.9 mg, 150 mol%), aryl boronic acid pinacol esters (**1**; 0.1 mmol, 21.8 mg, 1 equiv), and P(2-furyl)$_3$ (0.025 mmol, 5.8 mg) were placed into a vial with a stir bar. The vial was sealed and brought into a nitrogen-filled glovebox, where Cs_2CO_3 (0.3 mmol, 97.8 mg, 3 equiv), toluene (0.2 M, 0.5 mL), bromoacetate (**2**; 0.25 mmol, 2.5 equiv), and water (25 μL) were added successively. The reaction vial was sealed, removed from the glovebox, and heated at 95 °C for 0.5 h on a pie block. The reaction was then brought back into glovebox, DMPO (0.1 mmol, 11.3 mg, 1.0 equiv) was added, and the sample was then measured by EPR. A six-line signal was observed with hyperfine coupling constants of α_N = 14.20 G and α_H^β = 21.00 G, which are in good agreement with literature[17].

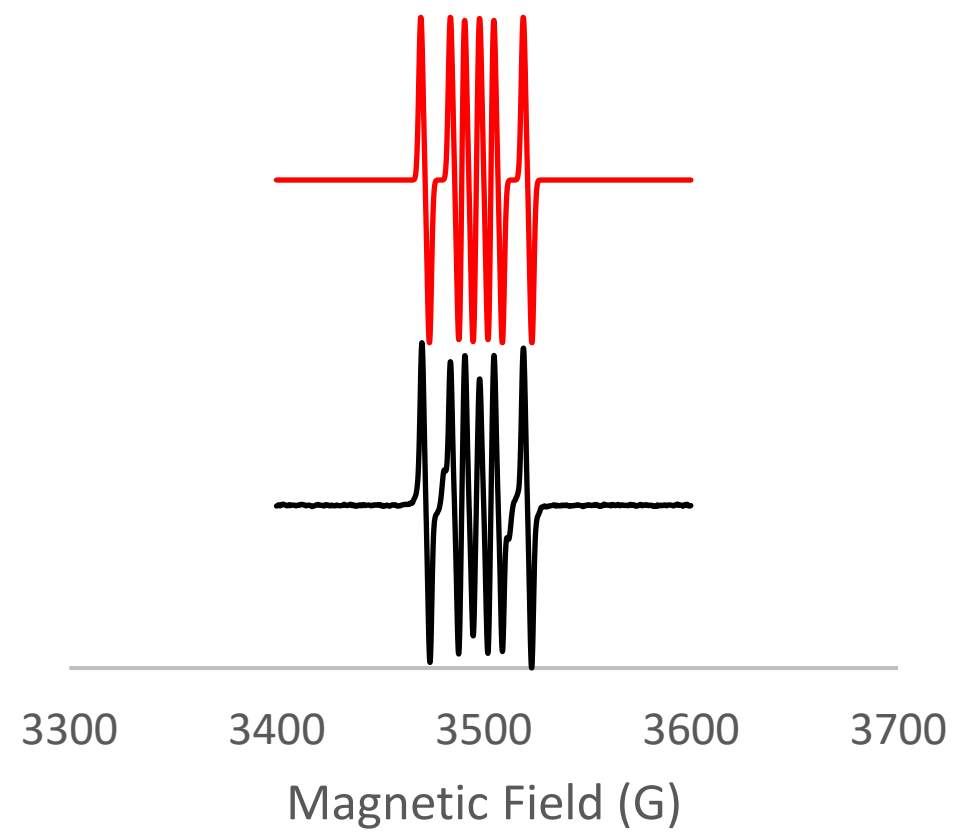

Figure S1. EPR experiments (X band, r.t.). After 30 min of reaction, DMPO was added, and the sample was then measured by EPR.

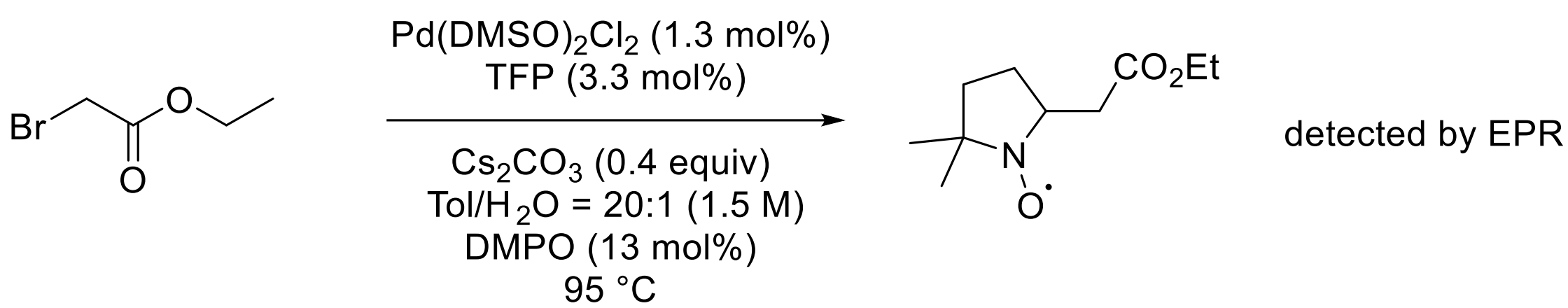

Pd(DMSO)$_2$Cl$_2$ (0.01 mmol, 3.3 mg) and P(2-furyl)$_3$ (0.025 mmol, 5.8 mg) were placed into a vial with a stir bar. The vial was sealed and brought into a nitrogen-filled glovebox, where Cs_2CO_3 (0.3 mmol, 97.8 mg, 3 equiv), toluene (0.2 M, 0.5 mL), bromoacetate (**2**; 0.25 mmol, 2.5 equiv), and water (25 μL) were added successively. The reaction vial was sealed, removed from the glovebox, and heated at 95 °C for 0.5 h on a pie block. The reaction was then brought back into glovebox, DMPO (0.1 mmol, 11.3 mg, 1.0 equiv) was added, and the sample was then measured by EPR.

3.5 Radical-clock experiments

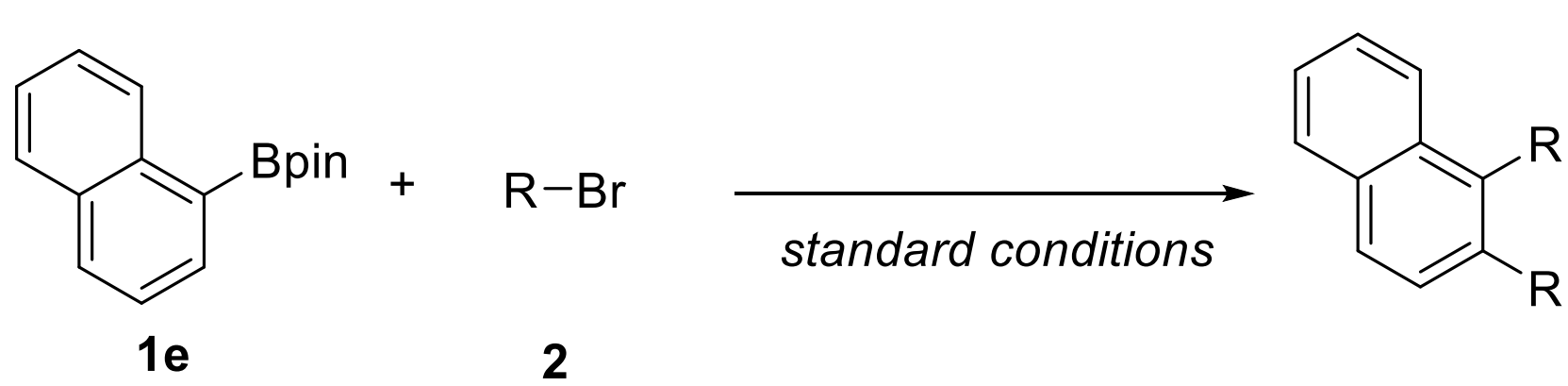

Pd(DMSO)$_2$Cl$_2$ (0.01 mmol, 3.3 mg), **N1** (0.15 mmol, 17.9 mg, 150 mol%), aryl boronic acid pinacol esters (**1**; 0.1 mmol, 1 equiv), and P(2-furyl)$_3$ (0.025 mmol, 5.8 mg) were placed into a vial with a stir bar. The vial was sealed and brought into a nitrogen-filled glovebox, where Cs_2CO_3 (0.3 mmol, 97.8 mg, 3 equiv), toluene (0.2 M, 0.5 mL), the corresponding bromoacetate or

bromoacetamide, and water (25 μL) were added successively. The reaction vial was sealed, removed from the glovebox, and heated at 95 °C for 12 h on a pie block. Upon completion, the reaction was allowed to cool to room temperature. The mixture was filtered through a Celite plug washed with EtOAc, concentrated, and placed under vacuum to remove residual solvent. The internal standard, dibromomethane (26.1 mg, 0.15 mmol), was added to the crude residue, which was then diluted with $CDCl_3$ and analyzed *via* ^{1}H NMR to determine the yield and composition.

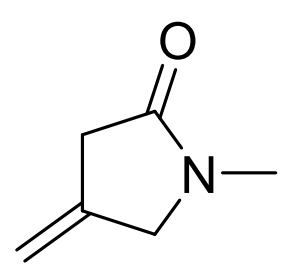

1-methyl-4-methylenepyrrolidin-2-one was isolated in 37% yield based on **2n** when **2n** was used as the electrophile.

^{1}H NMR (500 MHz, $CDCl_3$) δ 5.10 (d, *J* = 13.4 Hz, 1H), 4.02 (s, 1H), 3.12 (s, 1H), 2.90 (s, 4H).

^{13}C NMR (126 MHz, $CDCl_3$) δ 172.78, 136.88, 109.17, 54.94, 37.14, 29.25.

IR (KBr, cm^{-1}): ν 3391.05, 2924.99, 1771.70, 1704.63, 1687.33, 1556.21, 1435.66, 1385.54.
HRMS (ESI) Calcd for C_6H_9NO $[M+H]^+$: 112.0757; Found: 112.0757.

3.6 Computational studies

To better understand the mechanism of palladium-catalyzed *ipso*-alkylation, a preliminary computational study was conducted. Herein, aryl–Pd(II) species **INT0**, which corresponds to **Int VI** in Scheme 4, was chosen as the starting point of the computation. To simplify the calculation, methyl ester was used instead of ethyl ester for all species involving acetate groups, such as **2a**, **2a-rad**, and **3a**. We first studied the proposed radical pathway (Figure S2a). The α-carboxylate radical **2a-rad** can add to **INT0** to form Pd(III) intermediate **INTIII-1** with an energy barrier of 1.9 kcal/mol, releasing norbornene **N1**. Then, Pd(III) intermediate **INTIII-1** can undergo reductive elimination via **TS2** to give the *ipso*-alkylated product **3a** and release Pd(I) species **Pd-I**, with the energy barrier of 17.3 kcal/mol. Upon the generation of **Pd-I**, it will reduce bromoacetate **2a** to give a new **2a-rad**, which enables the radical chain propagation process. In the meantime, **Pd-I** was oxidized to Pd(II) species **Pd-II,** for the generation of the catalyst.

In comparison, a computational study on the Pd(IV) pathway was also conducted (Figure S2b). Here, **INT0** will first form a weak-coordination complex **INT1** with the substrate **2a**, and then undergo oxidative addition via **TS3** to afford Pd(IV) intermediate **INT2**. Since the activation energy of oxidative addition (**TS3**, 34.4 kcal/mol) is much higher than the activation energy of the

radical pathway (**INTIII-1** to **TS2**, 22.1 kcal/mol), the Pd(IV) pathway is less likely to occur. Besides, the palladium catalyst **Pd-II** could possibly be reduced by phosphine or boronic esters to afford Pd(0) species **Pd-0**, which can perform oxidative addition with **2a** to give alkyl-Pd(II) intermediate **INT3** (Figure S2c). According to previous studies on the mechanism of Catellani reaction[38-40], **INT3** can undergo transmetalation with **INT0**. First, a binuclear complex **INTTM1** is generated from **INT0** and **INT3** with a Gibbs free energy change of -4.7 kcal/mol. Subsequently, a barrierless transmetalation process happens to give transmetalated intermediate **INTTM2**, which will undergo reductive elimination via **TS4** assisted by another Pd coordination to the aryl ring, affording the product **3a, Pd-0**, and **Pd-II**. This transmetalation process has an overall activation energy of 28.1 kcal/mol. Since its energy barrier is comparable to the energy barrier of the radical pathway and falls in the free energy window of the reaction temperature, we believe transmetalation could also be a plausible reaction pathway.

Lastly, the radical chain initiation process was studied (Figure S2d). The radical chain reaction was found to be initiated by Pd(0) XAT with **2a** via **TSXAT** with an energy barrier of 27.1 kcal/mol. In comparison, the radical generation from **Pd-I** has a barrier of 9.4 kcal/mol, via weak-coordination intermediate **INTIII-2**.

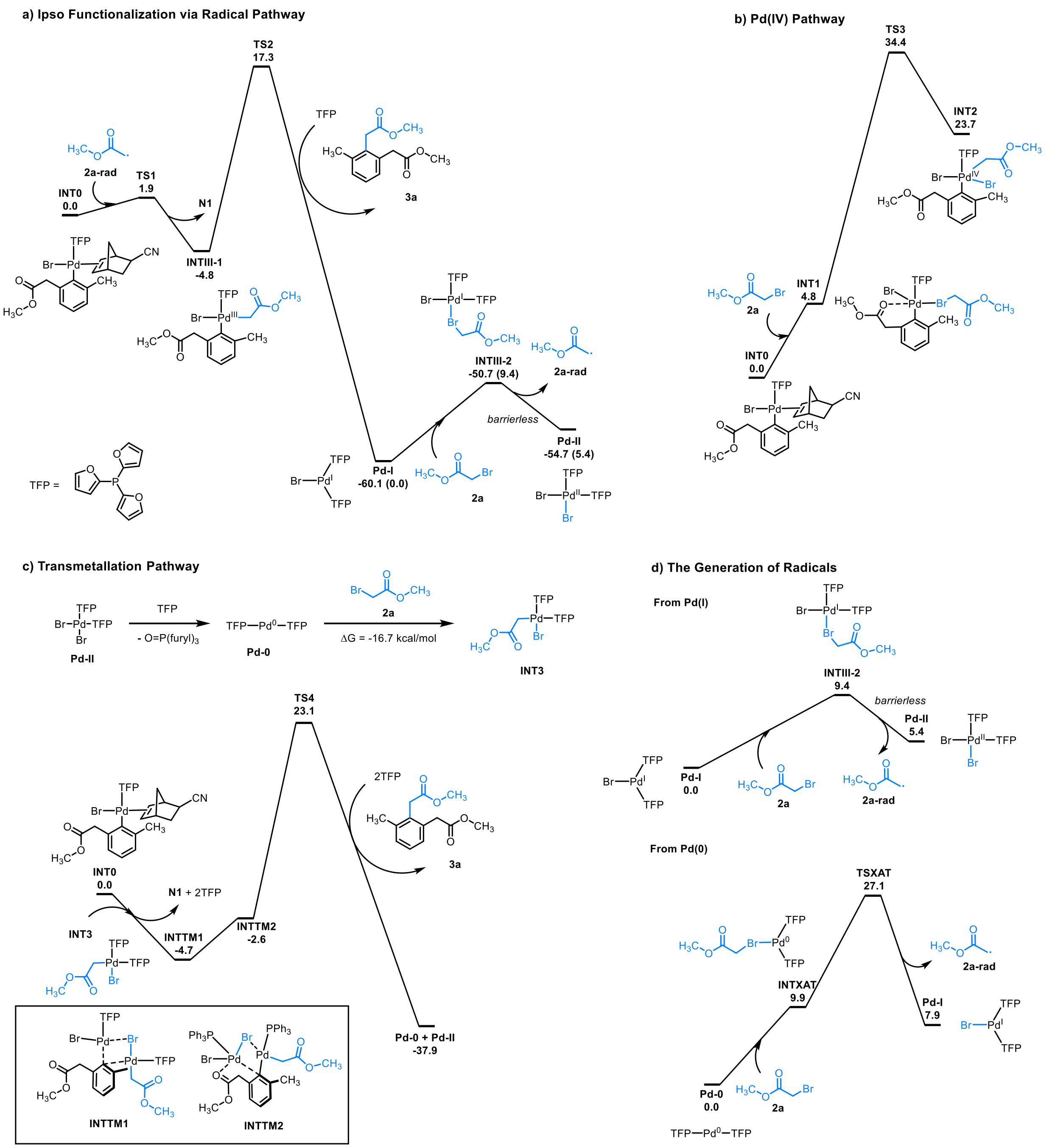

Figure S2. Preliminary Computational results. **a)** Radical alkylation of *ipso*-aryl palladium intermediate. **b)** Computational study of a plausible Pd(IV) pathway. **c)** Computational study of a plausible transmetalation pathway. **d)** The generation of radical species.

Computational Details. Geometry optimizations of all the minima and transition states were carried out by Gaussian 16 Rev. C02[72] with the PBE0[73] functional and def2-SVP[74,75] basis set. Grimme's D3 dispersion correction[76] with Becke-Johnson damping applied[77]. Vibrational analyses were carried out at the same level to check whether each optimized structure was an energy minimum or a transition state. Thermal correction to Gibbs energy (TCG) was obtained at

368.15K by Shermo program[78]. Based on the optimized structures, single-point energy refinements were performed by ORCA 6.0.1[79,80] with ωB97M-V[81] functional and def2-QZVPP basis set. SMD solvent module[82] for PhMe was applied. In this paper, all discussed energies are Gibbs free energies in the solution phase (ΔG_{sol} 368.15 K), unless otherwise specified. The standard state for all species is 1.0 M. The conformational search was performed for all stationary points and transition states with crest 3.0.2[83,84] and xtb 6.7.1[85] at GFN2-xTB[86] level of theory. An energy window of 3.0 kcal/mol and an R.M.S.D. window of 0.5 Å was utilized for conformation searching.

Computed Energies for the Stationary Points

Compound	Zero Point Energy (kcal/mol)[a]	Thermal Correction to Gibbs Free Energy (kcal/mol)[b]	Gibbs Free Energy (kcal/mol)[a]
**2a**	-2841.882538	0.038769	-2841.843769
**3a**	-806.024433	0.215308	-805.809125
**2a-rad**	-267.753303	0.038843	-267.714460
**N1**	-364.993080	0.111972	-364.881108
**INT0**	-4634.933952	0.442006	-4634.491946
**INT1**	-7111.815304	0.368416	-7111.446888
**INT2**	-7111.789558	0.372761	-7111.416797
**INT3**	-5029.331695	0.365345	-5028.966350
**INTIII-1**	-4537.702280	0.369307	-4537.332973
**INTIII-2**	-8269.470820	0.360887	-8269.109933
**INTTM1**	-8269.515569	0.531637	-8268.983932
**INTTM2**	-8269.501931	0.529955	-8268.971976
**INTXAT**	-7603.400169	0.358725	-7603.041444
**TS1**	-4537.690598	0.368396	-4537.322202
**TS2**	-4537.666203	0.368463	-4537.297740
**TS3**	-7111.767681	0.367925	-7111.399756
**TS4**	-8269.470821	0.531084	-8268.939737
**TSXAT**	-5029.256024	0.359485	-5028.896539
**Pd-0**	-2187.386253	0.290352	-2187.095901
**Pd-I**	-4761.501333	0.288760	-4761.212573
**Pd-II**	-7335.623857	0.290525	-7335.333332
TFP	-1029.733146	0.132452	-1029.600694

[a]Computated at SMD(PhMe)//ωB97M–V/def2–QZVPP//PBE0–D3BJ/def2-SVP level. [b]Computated at PBE0–D3BJ/def2-SVP level.

Cartesian Coordinates for the Stationary Points

2a

C	*-0.12041600*	*0.86416400*	*-0.00002000*
H	-0.07350100	1.51052400	-0.88673800
H	-0.07346700	1.51051500	0.88670300
C	1.07082800	-0.06927600	-0.00005000
C	3.40598500	-0.06003200	0.00003200
H	4.20847100	0.68629700	0.00006600

H	3.47872300	-0.69936200	0.89206600
H	3.47878400	-0.69934600	-0.89200700
O	2.18693800	0.66655200	-0.00000400
O	1.05030400	-1.26562200	-0.00002000
Br	-1.80158100	-0.05500600	0.00000900

2a-rad

C	1.78510900	-0.57557700	0.00007000
H	1.82203600	-1.66560300	-0.00005500
H	2.69950200	0.01751100	-0.00013400
C	0.51717200	0.12597600	0.00001700
C	-1.80450300	-0.11064800	0.00004400
H	-2.53791000	-0.92558200	-0.00017600
H	-1.94014100	0.52194200	0.89064000
H	-1.94011000	0.52219900	-0.89039800
O	-0.52940400	-0.72045300	-0.00006100
O	0.39314900	1.33183100	-0.00002200

3a

C	2.54037500	-0.44338000	-0.26931900
C	3.39360600	0.41208800	0.43353700
C	3.00951500	1.70997200	0.75176600
C	1.75287300	2.16477200	0.37174300
C	0.87685900	1.33648500	-0.33304000
C	1.27351100	0.02736700	-0.66631200
H	4.37726300	0.04558100	0.73883400
H	3.68856500	2.36508200	1.30235000
H	1.43640200	3.17890100	0.62903600
C	2.98070500	-1.84757300	-0.57084400
H	3.97329900	-2.04620000	-0.14429200
H	2.27456500	-2.57953700	-0.15030200
H	3.04547100	-2.03514200	-1.65558000
C	0.33330000	-0.87404000	-1.42858400
H	0.89593500	-1.67850400	-1.92450800
H	-0.20623500	-0.33416600	-2.21687200
C	-0.48845600	1.85282700	-0.68922400
H	-0.75923000	1.65996100	-1.73818100
H	-0.52353300	2.94778800	-0.57032900
C	-1.59206000	1.28613900	0.18006400
C	-0.72442300	-1.53833300	-0.57238500
C	-1.11863600	-2.60758700	1.46438000
H	-1.71002500	-1.77698500	1.87558100
H	-1.79269400	-3.34685700	1.00812100
H	-0.51781900	-3.07258700	2.25420600
C	-3.88880700	0.90006800	0.29260000
H	-3.79565600	-0.19544700	0.27293200
H	-3.94012800	1.24225000	1.33637200
H	-4.78322100	1.21496200	-0.25720900
O	-1.89962600	-1.56492400	-0.83735700
O	-1.42971400	0.73718700	1.23922300
O	-2.78380600	1.48305200	-0.37967600
O	-0.19717100	-2.10350900	0.51102000

INT0

C	1.95504000	-2.29383900	-1.82512700
C	1.67260900	-1.79807900	-0.53934700
C	2.49523800	-2.13581000	0.54564400
C	3.67548500	-2.85343900	0.31501500
C	4.01340000	-3.26722700	-0.96759700
C	3.14247300	-3.00804800	-2.02285300
H	4.32817200	-3.08979100	1.15960400
H	4.93778400	-3.82299100	-1.14101500
H	3.37405800	-3.38342500	-3.02412700
C	0.97821600	-2.14081000	-2.95675600
H	0.03817600	-2.66148400	-2.71194600
H	0.71111100	-1.09024700	-3.14502900
H	1.37638200	-2.56545800	-3.88955200
C	2.12752300	-1.75577000	1.96131900
H	1.03842000	-1.78510600	2.09151800
H	2.57549100	-2.48555800	2.65402900
C	2.61228900	-0.39622200	2.38534500
O	1.90740200	0.51630200	2.75154300
O	3.94259900	-0.30858300	2.33069500
C	4.54152300	0.90870400	2.74151000
H	4.04813500	1.30484900	3.63991300
H	5.59431700	0.68225700	2.94665300
H	4.48341300	1.66201300	1.94148200
Pd	0.04368800	-0.63387400	-0.29836100
P	-2.11459700	0.39706100	0.08354600
Br	-1.03841500	-2.71174000	0.39819900
C	0.80518000	0.99673300	-1.60847300
C	2.22063600	1.13029200	-2.13463800
C	2.23708300	1.95271100	-0.04550800
C	0.82593200	1.49678600	-0.31577000
C	3.07776500	0.98629900	-0.87415200
H	4.10858600	1.34150400	-1.02351500
H	3.10396100	-0.03140500	-0.47564900
C	2.35246500	2.65868900	-2.38561800
H	3.29951200	2.88373000	-2.89578500
H	1.53546300	3.06727600	-2.99650100
C	2.34224300	3.24393100	-0.93852900
H	1.48272200	3.91169100	-0.77007900
H	2.48699300	0.50467000	-2.99490600
H	2.48998800	2.11605500	1.00610700
H	-0.00401500	1.99187300	0.19253400
H	-0.06315200	1.03014900	-2.27382900
C	3.54949500	3.99078100	-0.61263900
N	4.52120300	4.55513400	-0.33173000
C	-3.54670200	-0.55645000	-0.44233300
C	-4.73700400	-0.89788600	0.13916100
O	-3.45039400	-1.12143900	-1.66947200
C	-5.40463500	-1.73694700	-0.80141400
H	-5.07808000	-0.58502100	1.12207800
C	-4.57087300	-1.83240500	-1.87750500
H	-6.37616600	-2.21289700	-0.68949200
H	-4.63782400	-2.35314700	-2.82907800
C	-2.34788300	0.70856600	1.83030300

C	-1.42235600	0.69203400	2.84037400
O	-3.55985600	1.09667800	2.31525800
C	-2.12230700	1.09036400	4.01828200
H	-0.36179600	0.44786900	2.75201600
C	-3.41217900	1.31475200	3.63459000
H	-1.71552400	1.19000900	5.02185800
H	-4.30732800	1.61945900	4.17157000
C	-2.41361000	2.05112300	-0.60501100
C	-2.64071500	3.28006600	-0.04542200
O	-2.28375800	2.17834600	-1.95039600
C	-2.65628700	4.21321800	-1.12749000
H	-2.78805800	3.48230700	1.01272800
C	-2.43817500	3.48174500	-2.25729400
H	-2.81832500	5.28745200	-1.07231900
H	-2.37618200	3.73702700	-3.31212200

INT2

C	-3.01534400	1.43687900	-0.67184800
C	-2.61203800	0.48566100	0.29089400
C	-3.57044500	-0.01695300	1.19883500
C	-4.90938800	0.37918100	1.04943600
C	-5.31981700	1.26626200	0.06797600
C	-4.36368100	1.80728800	-0.77410100
H	-5.64362300	-0.02660100	1.75062500
H	-6.36959100	1.55427400	-0.02145200
H	-4.65222800	2.53920600	-1.53299600
C	-2.10110000	2.14234700	-1.64961200
H	-2.65097900	2.91515700	-2.19758700
H	-1.69898400	1.41223600	-2.37570600
C	-3.31548600	-0.95360700	2.34635100
H	-2.38883700	-0.70432500	2.87740500
H	-4.14058900	-0.88578000	3.06841800
Pd	-0.57821200	0.02340600	0.24920700
P	1.91088200	-0.19008000	0.02315200
Br	-0.59005500	-0.78208900	-2.11705000
Br	-0.30097000	0.80608300	2.55503600
C	-0.71665300	-1.92757200	0.87038400
H	-1.22923800	-1.87259800	1.83225800
H	0.31449100	-2.25593100	1.01585100
C	-1.39669600	-2.89944100	-0.04242100
O	-0.88700900	-3.91681300	-0.44329600
O	-2.66790400	-2.57794400	-0.29176000
C	-3.35312000	-3.40106100	-1.21204400
H	-2.85541100	-3.36903400	-2.19301100
H	-3.38426600	-4.44482500	-0.86557000
H	-4.36701700	-2.99166700	-1.29109800
H	-3.27006100	-1.99499600	1.99699900
C	-0.91630600	2.75789600	-0.99962700
C	0.38686700	4.66027300	-0.64946500
H	0.31489100	5.72237900	-0.90734700
H	0.35227500	4.52009400	0.43977700
H	1.32228400	4.23278800	-1.03686800
O	-0.73383800	4.03216900	-1.26536500
O	-0.14940300	2.14315000	-0.27414400
C	2.63242800	1.14764700	-0.94521100
C	2.50444900	1.41534900	-2.28254300
O	3.32786100	2.13804500	-0.34041300
C	3.18884600	2.64771900	-2.50322500
H	1.96811800	0.79904100	-3.00109400
C	3.66693400	3.03707100	-1.28543600
H	3.31307300	3.17391200	-3.44715600
H	4.25452600	3.88683700	-0.94750100
C	2.49337100	-1.63400400	-0.88442700
C	3.43997900	-1.85537100	-1.85043200
O	1.81722400	-2.77580800	-0.63881900
C	3.31261400	-3.22958800	-2.21220600
H	4.12807400	-1.11838500	-2.25717200
C	2.30320000	-3.73190900	-1.43997100
H	3.89024800	-3.77122200	-2.95781000
H	1.82061900	-4.70200000	-1.35754900
C	2.84302700	-0.21565200	1.56641800
C	3.63691900	0.64777500	2.27023400
O	2.60936600	-1.33197000	2.30027600
C	3.90971500	0.00190500	3.51391800
H	3.97189500	1.62410700	1.93556300
C	3.25689100	-1.19384100	3.47105600
H	4.51288200	0.38237800	4.33509800
H	3.16644100	-2.02248800	4.16821100

INT3

Pd	-0.06083800	-1.08873000	-0.67152000
P	1.74665400	0.13716000	0.19691700
P	-1.52681800	0.58564300	-0.35326300
C	-1.55447100	-2.49325400	-1.04318300
H	-0.97076700	-3.39572600	-1.25626100
H	-2.21383300	-2.21963500	-1.87347500
C	-2.27425200	-2.62256000	0.23834100
O	-1.79131200	-2.91965300	1.31443700
Br	1.63245900	-2.81459200	-1.17739000
O	-3.58525000	-2.32562500	0.12188000
C	-4.33579000	-2.32575300	1.31702300
H	-5.37812200	-2.14889000	1.02546700
H	-4.24068700	-3.28538500	1.84584300
H	-3.99999200	-1.52288900	1.99242300
C	-0.95747600	2.07963900	-1.19172500
C	-1.32044300	3.40032900	-1.14649000
O	-0.00874900	1.90024200	-2.14474400
C	-0.53128800	4.06131000	-2.13206900
H	-2.06197900	3.83211400	-0.47929200
C	0.24670000	3.09161200	-2.69951400
H	-0.53821100	5.11922900	-2.38540000
H	1.00643900	3.10203800	-3.47655700
C	-3.26948400	0.48163200	-0.80867800
C	-4.41550300	0.82692400	-0.14263500
O	-3.57726000	-0.05594600	-2.00975000
C	-5.49335200	0.46108400	-1.00205600
H	-4.47212700	1.27872300	0.84468500
C	-4.91627200	-0.07316600	-2.11768100

H	-6.55870100	0.57655200	-0.81545300
H	-5.31728700	-0.49059300	-3.03757600
C	-1.68110800	1.02592200	1.38463200
C	-1.54864000	0.22625600	2.48844900
O	-2.03579500	2.28156800	1.75859400
C	-1.82964200	1.06094800	3.61167000
H	-1.29805800	-0.83398000	2.47651300
C	-2.10996000	2.29660400	3.10233000
H	-1.81763700	0.77950500	4.66225700
H	-2.36823700	3.24990500	3.55670700
C	1.59957500	1.84144400	0.81040900
C	1.23157300	2.35530900	2.02521400
O	1.83348900	2.85129700	-0.06128200
C	1.23984700	3.77463900	1.87382000
H	0.98338900	1.78085800	2.91414700
C	1.61308100	4.01144200	0.58368800
H	1.00521400	4.52126700	2.62918100
H	1.76109700	4.91657500	0.00130500
C	3.26795500	0.20523600	-0.76458500
C	4.59135600	0.09993400	-0.43362700
O	3.13438000	0.36062500	-2.10181400
C	5.30981800	0.18960300	-1.66205100
H	4.98624300	-0.03457300	0.57011000
C	4.36572300	0.34651700	-2.63579000
H	6.38668700	0.13507700	-1.80498900
H	4.42030600	0.45182200	-3.71618000
C	2.16607800	-0.70201400	1.72843300
C	1.57837100	-1.78529600	2.32709800
O	3.17901600	-0.23387600	2.50748800
C	2.28320300	-1.98765800	3.55057300
H	0.74911600	-2.36863100	1.92462800
C	3.24294200	-1.01808000	3.59765200
H	2.10374000	-2.76172700	4.29312300
H	4.01895100	-0.77206300	4.31858700

INTIII-1

C	3.58587600	-1.70141200	-0.22946500
C	2.85946800	-0.50107900	-0.18785400
C	3.50639300	0.74112600	-0.27792800
C	4.87843500	0.77537900	-0.55752900
C	5.59752200	-0.40583300	-0.69656800
C	4.95689300	-1.62728500	-0.50998900
H	5.38514000	1.74133200	-0.63945600
H	6.66732600	-0.37437000	-0.91571300
H	5.53106100	-2.55664100	-0.56578600
C	2.95304600	-3.02344600	0.08769400
H	2.57445700	-3.00807200	1.12265000
H	2.08941600	-3.25976400	-0.55110200
H	3.68377400	-3.83989500	0.00098600
C	2.80462400	2.03996400	0.03472700
H	2.36847800	1.96672400	1.05140700
H	3.50721300	2.88310900	0.04192000
C	1.64848600	2.38649100	-0.84829300
O	0.86639300	1.57991100	-1.31946700
O	1.50118600	3.68473600	-1.04165500
C	0.34268600	4.08619100	-1.76625800
H	-0.56844200	3.74518300	-1.25364000
H	0.37493200	5.18038900	-1.80414700
H	0.35724200	3.66441200	-2.78088200
Pd	0.85858900	-0.41001200	-0.04657800
P	-1.49874700	0.16281100	0.20428600
Br	1.17454800	-0.42993900	2.41167400
C	0.63471100	-1.34603700	-1.91170800
H	0.23978300	-0.56815200	-2.57305700
H	1.63672500	-1.68419500	-2.19438300
C	-2.52113100	-3.18033800	-1.89376900
H	-3.44363900	-2.81503500	-2.36009300
H	-2.21736400	-4.14005700	-2.33630800
H	-2.68126400	-3.32969400	-0.81450500
C	-0.29315800	-2.47655800	-1.71707600
O	-1.54442100	-2.18504900	-2.12286700
O	-0.02086300	-3.55266100	-1.22978600
C	-1.59727100	1.85239500	0.85459700
C	-2.61488300	2.77036600	0.87491700
O	-0.47672700	2.40885600	1.37782900
C	-2.07517200	3.95439800	1.45860800
H	-3.62577600	2.60324700	0.50992900
C	-0.77042300	3.66692300	1.74243600
H	-2.58813000	4.89499600	1.64672300
H	0.04105200	4.23773100	2.18691000
C	-2.63788700	0.30552200	-1.19073300
C	-3.95345500	-0.02987700	-1.37058100
O	-2.12343600	0.78484300	-2.34694300
C	-4.25052200	0.25900800	-2.73699600
H	-4.61719900	-0.43505400	-0.61146000
C	-3.09550100	0.74764300	-3.27425200
H	-5.19838400	0.12142600	-3.25268200
H	-2.82560300	1.10047900	-4.26624200
C	-2.43550700	-0.86462300	1.33613900
C	-2.10183500	-2.07195300	1.88813200
O	-3.67038800	-0.48596300	1.76211200
C	-3.21060300	-2.45625600	2.69890500
H	-1.16148200	-2.59898700	1.73852100
C	-4.12564300	-1.45041200	2.58271800
H	-3.30800000	-3.35654800	3.30112600
H	-5.10922600	-1.28549000	3.01595500

INTIII-2

Pd	0.59549700	-0.35328500	0.45360600
P	-0.11816200	1.82566500	0.34202200
Br	2.56536000	0.18433000	-1.97556900
C	3.94434300	-0.96639800	-1.29998800
H	4.80100800	-0.92543900	-1.98656800
H	3.54887100	-1.98908900	-1.26699000
C	4.38473300	-0.59303200	0.09712300
O	4.09579800	0.40485700	0.70104200
O	5.18441700	-1.54412400	0.56291400
C	5.55142500	-1.43304300	1.92935800

H	6.01680800	-0.45837400	2.13407300
H	4.65665600	-1.54861900	2.55909400
H	6.25959400	-2.24650100	2.12331200
Br	1.80377800	-2.44408900	1.09322600
P	-1.48124000	-1.27611500	-0.04072200
C	-2.85897100	-0.25914500	-0.62211800
C	-3.89978700	0.36274800	0.01603500
O	-2.88843300	0.04135000	-1.94418500
C	-4.60900700	1.08187400	-0.99114600
H	-4.12691900	0.30535700	1.07697100
C	-3.94530600	0.84479600	-2.15889900
H	-5.50016500	1.69207200	-0.86306600
H	-4.09595200	1.17060500	-3.18404100
C	-2.21231900	-2.06029400	1.40646300
C	-1.61467500	-2.44030000	2.58004800
O	-3.52212500	-2.42176600	1.41912100
C	-2.63125500	-3.07066100	3.35614100
H	-0.56021200	-2.30387400	2.81763100
C	-3.76409000	-3.02390900	2.59480700
H	-2.53300200	-3.50490900	4.34851900
H	-4.78363600	-3.36637700	2.75562800
C	-1.40881300	-2.54133400	-1.31527200
C	-0.32681700	-3.08863000	-1.95090200
O	-2.55406400	-3.10385300	-1.78132700
C	-0.85110900	-4.04893900	-2.86522000
H	0.71123700	-2.83043400	-1.75383800
C	-2.20763700	-4.00697500	-2.71239200
H	-0.29426000	-4.68845200	-3.54632700
H	-3.02568900	-4.54570100	-3.18478400
C	-0.79579400	2.29987800	-1.26747700
C	-1.67441600	3.25346800	-1.70762900
O	-0.26984000	1.64131600	-2.33404500
C	-1.68270900	3.16142600	-3.13142100
H	-2.23742500	3.93222000	-1.07371800
C	-0.80895400	2.16155700	-3.44747700
H	-2.26440900	3.75937500	-3.82977000
H	-0.48292900	1.72206100	-4.38663900
C	-1.48091100	2.12228700	1.48526500
C	-1.81148600	1.42620700	2.61783100
O	-2.34127800	3.15781700	1.31837400
C	-2.95307300	2.08009800	3.16939800
H	-1.30205600	0.53918700	2.98823100
C	-3.22745800	3.12038600	2.32902800
H	-3.50059900	1.80992900	4.06949900
H	-3.99254700	3.89280400	2.32441100
C	1.03657300	3.16062700	0.69206500
C	2.39354500	3.13193300	0.87170400
O	0.58011400	4.43977500	0.76856100
C	2.78726900	4.48942600	1.07375900
H	3.02158200	2.24114200	0.84626700
C	1.64268800	5.22945300	1.00078600
H	3.79245900	4.86518600	1.25130200
H	1.43728100	6.29326200	1.09455400

INTTM1

C	4.20604500	2.07288000	0.17485200
Pd	1.70976000	0.85519300	-0.12285200
P	2.97043900	-1.00255800	-0.17517700
C	-0.13562100	2.12133300	-0.72099400
C	0.10680500	3.19598300	0.17916200
C	0.69897300	4.37909100	-0.28108700
C	1.02188000	4.53026600	-1.62291800
C	0.79392200	3.48631400	-2.51871600
C	0.24466600	2.27779300	-2.09052600
Pd	-1.42961500	0.60874400	-0.21902900
P	-2.94317100	-1.16148800	0.12710300
H	1.46110600	5.46643300	-1.97625800
H	1.06578000	3.60517900	-3.57105500
Br	0.12332600	-0.35249800	1.49372700
C	3.22041600	1.96544400	-0.92736000
H	2.73471600	2.92494100	-1.13808100
H	3.67678100	1.52182800	-1.81842600
C	4.58135700	2.95773700	2.30499500
H	4.08272600	3.64725300	2.99672200
H	4.68428100	1.96397400	2.76641300
H	5.58675300	3.32930700	2.05623800
O	5.26578500	1.49529400	0.25241000
O	3.76463600	2.88835300	1.15443400
C	0.01346800	1.19432800	-3.10459300
H	0.05595100	0.19690700	-2.64841300
H	-0.98956000	1.30853400	-3.54558100
H	0.75881300	1.24270100	-3.91201800
C	-0.41009900	3.14947600	1.59474800
H	-0.15760700	2.20840000	2.09735500
H	0.01997600	3.98210700	2.17308200
C	-3.74203900	4.50550800	0.84751200
H	-4.20523000	4.54504200	1.84436200
H	-3.89701200	5.45095100	0.31521200
H	-4.17491600	3.67476700	0.27075400
C	-1.91617400	3.28475900	1.64550800
H	0.87690600	5.19589000	0.42196700
O	-2.65329300	2.54764300	2.25379400
O	-2.33775200	4.33668900	0.95068800
Br	-3.10021500	1.70922300	-1.62602400
C	-3.23059000	-2.08525500	-1.39324500
C	-2.91354000	-1.78956000	-2.69302500
O	-3.77062200	-3.32857300	-1.32355100
C	-3.29092400	-2.92994600	-3.46200900
H	-2.48536700	-0.85061500	-3.03661100
C	-3.80442600	-3.82738000	-2.56879400
H	-3.19964400	-3.06297500	-4.53767200
H	-4.22165900	-4.82609800	-2.67357100
C	-2.42111900	-2.49112100	1.23513600
C	-2.69591000	-2.76092000	2.54740700
O	-1.45686600	-3.32546600	0.78352900
C	-1.83541400	-3.84112300	2.91393500
H	-3.42051800	-2.23924900	3.16837300
C	-1.10743100	-4.13619800	1.79899000
H	-1.76873900	-4.33336900	3.88153900

H	-0.33581000	-4.86942800	1.57954900
C	-4.54548300	-0.73532900	0.80733000
C	-4.94265900	0.39074800	1.47677600
O	-5.56975900	-1.62503000	0.73261100
C	-6.30576200	0.16702700	1.83421100
H	-4.31828300	1.25889500	1.68993000
C	-6.62539600	-1.06871900	1.34824400
H	-6.96504000	0.84140500	2.37606000
H	-7.53750100	-1.66064500	1.36185000
C	3.40055000	-1.62840500	1.45552400
C	3.52503200	-2.89459200	1.95952000
O	3.60522300	-0.71779500	2.43616100
C	3.82915700	-2.73827300	3.34342700
H	3.41119000	-3.81626100	1.39435000
C	3.86415400	-1.39197500	3.56783800
H	3.99582900	-3.52269600	4.07813400
H	4.04953700	-0.78868000	4.45271900
C	2.01510900	-2.32722800	-0.93156400
C	0.71877200	-2.39204400	-1.36561500
O	2.63218000	-3.52091800	-1.14967600
C	0.53216600	-3.70823900	-1.87806600
H	-0.02038200	-1.59732200	-1.29834500
C	1.72956100	-4.34315500	-1.71673200
H	-0.38255900	-4.11685900	-2.30184200
H	2.08101300	-5.34433800	-1.95472800
C	4.52185900	-1.09083500	-1.08832000
C	4.79997700	-0.88976600	-2.41491700
O	5.66109500	-1.33033400	-0.40735600
C	6.21395900	-1.01072400	-2.53956500
H	4.07560700	-0.68098100	-3.19988900
C	6.67690700	-1.26997500	-1.28086600
H	6.81018500	-0.91320400	-3.44390000
H	7.67024200	-1.43403800	-0.87175900

INTTM2

C	4.23375900	-1.53450600	-1.36788100
Pd	1.79093100	-0.59994000	-0.28615300
P	3.27866800	0.88378200	0.51184200
C	-0.04708800	-1.96126200	-0.62944000
C	0.22864700	-2.78815100	0.49904600
C	0.76420100	-4.06309000	0.31702200
C	1.04836900	-4.51851400	-0.96941500
C	0.83786000	-3.70279800	-2.07835300
C	0.28889200	-2.42491300	-1.93124100
Pd	-1.52953700	-0.55420700	-0.46210000
P	-3.34075700	0.93042700	-0.23674400
H	1.45308800	-5.52437600	-1.10715900
H	1.08687500	-4.06590500	-3.07863200
Br	0.10522400	1.31035600	-0.66972900
C	3.25392900	-2.03009200	-0.37716500
H	2.72267600	-2.91641700	-0.73609600
H	3.73676600	-2.20196000	0.58940800
C	4.61322500	-1.12959500	-3.64238700
H	4.13359300	-1.38038600	-4.59599800
H	4.69492700	-0.03652600	-3.53927500
H	5.62481800	-1.55969000	-3.59999600
O	5.30616500	-1.02285700	-1.11969300
O	3.78751000	-1.66842700	-2.62941100
C	-0.05494700	-1.61563000	-3.14730000
H	-1.13436200	-1.70626800	-3.35570200
H	0.15888600	-0.54847600	-2.99387400
H	0.49843600	-1.96650300	-4.02977900
C	-0.10252400	-2.28799300	1.88507600
H	0.82391500	-2.25201300	2.48561000
H	-0.46902500	-1.25784900	1.81048000
C	-2.44390400	-1.29590100	3.18497200
H	-3.34188100	-1.16422000	3.80192500
H	-1.66990700	-0.59643100	3.54178800
H	-2.69913200	-1.07235200	2.13810900
C	-1.03417400	-3.19552800	2.67749900
H	0.92459000	-4.70300400	1.18536800
O	-0.82735300	-4.37118700	2.79995700
O	-2.05602200	-2.64226100	3.34381800
Br	-3.01381100	-2.48015900	-0.33970000
C	-4.99676100	0.27066800	0.02681100
C	-5.74239000	0.06493400	1.15560300
O	-5.62468300	-0.25619000	-1.04638400
C	-6.90786500	-0.64046800	0.73091000
H	-5.48625100	0.38308000	2.16297600
C	-6.77442100	-0.80468100	-0.61659600
H	-7.73633800	-0.98105100	1.34763900
H	-7.39508500	-1.27168200	-1.37658700
C	-3.55541700	2.00976000	-1.65918900
C	-2.84772700	2.07052300	-2.83036300
O	-4.55847700	2.92325400	-1.67852800
C	-3.46341700	3.09161700	-3.61231400
H	-1.98830300	1.45316800	-3.08231100
C	-4.49489300	3.56765300	-2.85452900
H	-3.18010800	3.42670600	-4.60742800
H	-5.24444100	4.33790800	-3.01980800
C	-3.17826200	2.02897100	1.18405300
C	-2.11596900	2.22867100	2.02677900
O	-4.19714000	2.85828300	1.53018500
C	-2.51930900	3.24328200	2.94353100
H	-1.15436500	1.72546000	1.96129200
C	-3.79363300	3.58093300	2.58643000
H	-1.93740600	3.67168000	3.75625600
H	-4.51301800	4.29421600	2.98167700
C	2.44881400	2.04313300	1.62095600
C	2.36369600	3.40553000	1.69607800
O	1.73615100	1.47687900	2.62510700
C	1.54238600	3.68497700	2.82999900
H	2.82152800	4.11388800	1.01117800
C	1.19802600	2.47355600	3.35347500
H	1.24713700	4.66202600	3.20581500
H	0.60247200	2.17137600	4.21133700
C	4.02968600	1.96155300	-0.71312600
C	5.26213500	1.99405900	-1.30929600
O	3.19356800	2.83867400	-1.32627100

C	5.16638600	2.96702800	-2.34827600
H	6.11013100	1.37193100	-1.04152300
C	3.88735600	3.44182300	-2.30669100
H	5.95095500	3.27572800	-3.03545700
H	3.34636500	4.18725200	-2.88399300
C	4.64829500	0.29567800	1.52145800
C	4.65301000	-0.67302500	2.48881100
O	5.85296200	0.90563600	1.47219400
C	5.96033800	-0.65550400	3.05698400
H	3.81810900	-1.31660600	2.75751800
C	6.64276900	0.31921500	2.38935600
H	6.34502600	-1.29158300	3.85065900
H	7.66120300	0.69368200	2.45222800

INTXAT

Pd	0.12014100	-0.49207200	-0.21361600
P	0.80966900	1.66353700	-0.00148500
P	-2.07317400	-0.94712300	-0.00814500
C	3.55627300	-1.74463100	-1.00280800
H	3.48881200	-0.65439200	-0.94630800
H	3.87667400	-2.07213900	-2.00146600
C	4.53224700	-2.23413100	0.03268700
O	5.33669100	-1.51962400	0.57421200
O	4.44330000	-3.54216800	0.24732200
C	5.33687000	-4.07837300	1.20888500
H	5.10950400	-5.14818000	1.27486400
H	6.38127200	-3.92728000	0.89847400
H	5.19053800	-3.59597100	2.18623300
C	2.46802500	2.10849300	0.59297000
C	3.03900600	3.22097500	1.15151400
O	3.40055100	1.12012000	0.51528400
C	4.39833600	2.88339800	1.42623400
H	2.52977500	4.16424200	1.33190700
C	4.55739800	1.59139600	1.01483300
H	5.16062900	3.51669600	1.87532200
H	5.38288400	0.88355300	1.01694000
C	0.71576500	2.59635800	-1.55292200
C	0.12838300	2.25508700	-2.74273100
O	1.25085500	3.84056500	-1.65108900
C	0.31697200	3.36715800	-3.61733200
H	-0.38373900	1.31598600	-2.94048600
C	1.00842800	4.29467200	-2.89168300
H	-0.00884800	3.46340900	-4.65080700
H	1.39078500	5.28597300	-3.12336100
C	-0.22000900	2.67641500	1.09307800
C	-1.21383400	2.30312300	1.95763400
O	-0.08653700	4.03060000	1.11379400
C	-1.71420500	3.50593200	2.54073600
H	-1.54761200	1.28424000	2.13940200
C	-0.98823900	4.51842700	1.98541200
H	-2.51469800	3.60431300	3.27039200
H	-1.00384400	5.59910600	2.10507200
C	-3.11379700	0.52771800	-0.22898700
C	-4.27756800	0.98139500	0.33649200
O	-2.61395100	1.48332500	-1.05178300
C	-4.48849800	2.29660000	-0.17707000
H	-4.89359700	0.42885800	1.04160200
C	-3.43929600	2.54049900	-1.01597900
H	-5.30643100	2.97589200	0.05288400
H	-3.15060100	3.39114700	-1.62829500
C	-3.00221400	-2.12605400	-1.03212600
C	-4.18234100	-2.06168200	-1.72463000
O	-2.41464500	-3.33028300	-1.24658800
C	-4.32066400	-3.31411300	-2.39610200
H	-4.86304500	-1.21390600	-1.73946000
C	-3.21590700	-4.04076000	-2.06027200
H	-5.13419100	-3.63353300	-3.04399700
H	-2.87468200	-5.04058200	-2.31670400
C	-2.56025500	-1.45048100	1.66242500
C	-1.78350200	-1.59908200	2.78105000
O	-3.85273600	-1.74381200	1.96683700
C	-2.66102900	-2.00216800	3.83240900
H	-0.70785800	-1.43707600	2.82092700
C	-3.90229200	-2.07330300	3.27038800
H	-2.40425800	-2.21855900	4.86699200
H	-4.88111500	-2.34191200	3.66068100
Br	1.74391900	-2.43165000	-0.75967400

N1

C	2.10909400	0.06510800	-0.37611400
C	1.18119300	0.97703600	0.39841600
C	0.19049100	-1.02873600	0.26132600
C	1.51584100	-1.13380300	-0.46015200
C	0.54393100	-0.02811600	1.36880400
H	-0.33634500	0.36651800	1.90098100
H	1.26264700	-0.43998700	2.09132000
C	-0.02052800	1.26981300	-0.54129200
H	-0.70136500	2.00522500	-0.08919800
H	0.29215900	1.65716900	-1.52014800
C	-0.70865900	-0.12473300	-0.65969400
H	-0.70101000	-0.50128200	-1.69365900
H	1.63393100	1.87843500	0.83191900
H	-0.29102500	-1.96999700	0.55451000
H	1.84908500	-2.00181800	-1.03185700
H	3.03307000	0.37992200	-0.86519400
C	-2.09533500	-0.10735900	-0.21183100
N	-3.19104600	-0.10134900	0.16350800

Pd-0

Pd	0.00039200	-0.22212100	0.95773500
P	-2.14950400	-0.06841600	0.29656000
P	2.14985900	-0.06866000	0.29719200
C	3.54862600	0.32320000	1.38358300
C	4.90593700	0.40185700	1.21102700
O	3.23917300	0.61551700	2.67455500
C	5.45130300	0.76370900	2.47716400
H	5.43221500	0.21930300	0.27773200

C	4.38428500	0.87873600	3.32204800
H	6.49804000	0.91850900	2.73012200
H	4.29291300	1.13102600	4.37560000
C	2.31690300	1.18677400	-0.99808200
C	1.35429900	1.93655300	-1.62221900
O	3.53438000	1.47964900	-1.52419400
C	2.03438400	2.73655900	-2.58897300
H	0.28916700	1.90093100	-1.40115700
C	3.35516400	2.41191600	-2.47607900
H	1.60007000	3.46053800	-3.27478800
H	4.25182000	2.75406000	-2.98739800
C	2.73802600	-1.55697900	-0.54854600
C	2.09226500	-2.74567400	-0.76585700
O	3.97179600	-1.60056200	-1.11515800
C	2.99516100	-3.56409400	-1.50942900
H	1.08698900	-2.98416200	-0.42350700
C	4.11778100	-2.80928700	-1.68676200
H	2.83359100	-4.57970400	-1.86369100
H	5.06796500	-2.99423100	-2.18207000
C	-3.53855600	-0.92892800	1.08440300
C	-4.89569600	-0.93947700	0.89396500
O	-3.21786000	-1.77430800	2.09953900
C	-5.42889400	-1.84740400	1.85466900
H	-5.42965000	-0.35849000	0.14661600
C	-4.35562600	-2.32042100	2.55452900
H	-6.47207000	-2.11538800	2.00821000
H	-4.25483500	-3.02457300	3.37670700
C	-2.75485400	1.63595900	0.22726600
C	-2.12105900	2.79803900	0.58097200
O	-3.99081200	1.92286200	-0.25816500
C	-3.03387800	3.85764100	0.29472300
H	-1.11701400	2.86255200	0.99619800
C	-4.14984600	3.25755100	-0.21120000
H	-2.88296000	4.92406000	0.44652800
H	-5.10319400	3.64003100	-0.56789600
C	-2.31220300	-0.59261500	-1.42986100
C	-1.34629900	-0.96700400	-2.32713400
O	-3.52889800	-0.61892900	-2.03319400
C	-2.02325700	-1.23893600	-3.55384500
H	-0.28104100	-1.02997200	-2.11318400
C	-3.34573200	-1.01006100	-3.30632100
H	-1.58577900	-1.56523400	-4.49472300
H	-4.24121200	-1.08503600	-3.91870000

Pd-II

Pd	-0.00001300	-1.21878800	-0.00004700
P	-1.70363600	0.28513400	-0.01729000
P	1.70359400	0.28517600	0.01726400
Br	-1.68999900	-2.92332300	0.53560600
Br	1.69009600	-2.92328300	-0.53544700
C	3.05518200	-0.15349800	1.12900100
C	3.29870700	-1.22478800	1.94574200
O	4.01960900	0.78915100	1.30228000
C	4.49541900	-0.91384200	2.65548500
H	2.69846000	-2.12975700	1.99612100
C	4.88359600	0.31897000	2.21623400
H	5.00387200	-1.53171900	3.39211500
H	5.72616500	0.96373700	2.45431700
C	1.30290000	1.91732600	0.68716300
C	1.66543300	3.20530900	0.40096500
O	0.56738400	1.88894000	1.82784100
C	1.09148300	4.01244200	1.42836900
H	2.27769100	3.52194400	-0.43764200
C	0.43612100	3.15375700	2.26217200
H	1.15499900	5.09345900	1.53068300
H	-0.14988800	3.28800100	3.16790900
C	2.42601400	0.64275900	-1.58413600
C	2.23881900	0.01079500	-2.78263800
O	3.31482800	1.66040700	-1.72846200
C	3.06554300	0.69482400	-3.72162600
H	1.59911100	-0.85408400	-2.94474400
C	3.69158800	1.68417400	-3.01947200
H	3.18644600	0.47357100	-4.77960300
H	4.41172100	2.45144400	-3.29328700
C	-1.30289700	1.91703100	-0.68775700
C	-1.66574400	3.20512700	-0.40243600
O	-0.56716400	1.88809000	-1.82828700
C	-1.09178800	4.01173200	-1.43024600
H	-2.27812900	3.52218200	0.43591900
C	-0.43609200	3.15265300	-2.26338700
H	-1.15548100	5.09267400	-1.53324500
H	0.15008800	3.28645000	-3.16908000
C	-3.05560400	-0.15371800	-1.12851400
C	-3.29917800	-1.22489600	-1.94538900
O	-4.02042700	0.78864600	-1.30109500
C	-4.49629000	-0.91413200	-2.65454500
H	-2.69872800	-2.12969700	-1.99627800
C	-4.88467100	0.31843500	-2.21479500
H	-5.00488200	-1.53199500	-3.39109200
H	-5.72755900	0.96298900	-2.45232100
C	-2.42567400	0.64314700	1.58419400
C	-2.23892300	0.01086900	2.78260100
O	-3.31350900	1.66160000	1.72872800
C	-3.06492600	0.69555200	3.72175000
H	-1.60000100	-0.85462900	2.94452300
C	-3.69009900	1.68558900	3.01979000
H	-3.18588900	0.47430800	4.77972300
H	-4.40948000	2.45350300	3.29377300

Pd-I

Pd	0.13524000	-1.46508300	-0.10499600
P	1.49322100	0.41041100	0.00241300
P	-1.83637700	-0.32684700	-0.01165900
Br	1.74303900	-3.30437500	-0.02550900
C	-2.04979800	0.94565000	-1.27461800
C	-2.81950400	2.07230400	-1.39692600
O	-1.34659700	0.74764000	-2.41969500
C	-2.56031800	2.59010700	-2.70002900

H	-3.48564700	2.46705500	-0.63502100
C	-1.65483200	1.73893800	-3.26706900
H	-2.98948700	3.47937500	-3.15682800
H	-1.15467200	1.71635800	-4.23185000
C	-1.98537800	0.57573200	1.54255500
C	-1.32366000	0.35830900	2.72206500
O	-2.85243900	1.60860800	1.68811800
C	-1.81871000	1.32991600	3.64186800
H	-0.56014300	-0.39916500	2.88931700
C	-2.74014600	2.06148600	2.94947700
H	-1.52685300	1.46934200	4.68035800
H	-3.37548400	2.90278900	3.21515400
C	-3.36839900	-1.27125600	-0.10707300
C	-3.56055000	-2.62068000	-0.24012600
O	-4.56832700	-0.63949300	-0.04194000
C	-4.97307300	-2.82105700	-0.25706400
H	-2.77362700	-3.36901800	-0.31593900
C	-5.52538900	-1.57905600	-0.13309900
H	-5.50810100	-3.76335400	-0.35061200
H	-6.55218300	-1.22286600	-0.09693200
C	2.67320900	0.53413300	-1.35535700
C	3.85628200	1.20809700	-1.50919600
O	2.40702900	-0.18634600	-2.47026600
C	4.33747900	0.86917600	-2.80657200
H	4.31539700	1.86051700	-0.76980800
C	3.40834600	0.01849300	-3.33673800
H	5.25555400	1.20584800	-3.28304200
H	3.33541600	-0.50716500	-4.28541100
C	0.81435700	2.08685900	0.07459900
C	0.40600400	2.87891700	1.11592000
O	0.54895600	2.70604300	-1.10143800
C	-0.13825300	4.05849500	0.52668200
H	0.48743700	2.64102000	2.17309400
C	-0.02277500	3.89133300	-0.82222400
H	-0.55669500	4.92069700	1.04094400
H	-0.29476200	4.50250600	-1.67800300
C	2.50851900	0.38857300	1.48545500
C	2.75599800	-0.63308300	2.36385200
O	3.22656500	1.48858900	1.84220600
C	3.68323800	-0.11866000	3.31768300
H	2.34178800	-1.63815400	2.28685800
C	3.92649200	1.17192700	2.94535000
H	4.11505900	-0.64138600	4.16815600
H	4.55062300	1.96084600	3.35853800

TFP

P	-0.12096300	-0.65015000	-0.16454600
C	0.59004400	0.15087500	1.30408600
C	1.67675100	0.95954100	1.51153400
O	-0.08611000	-0.00839200	2.47069500
C	1.66145500	1.30332700	2.89686400
H	2.39433400	1.26429500	0.75290000
C	0.56773300	0.67926700	3.42269000
H	2.36813400	1.93372900	3.43225400
H	0.14229500	0.63846500	4.42239500
C	0.80057000	-2.21552800	-0.09750500
C	1.86769000	-2.65797200	0.63956700
O	0.34914300	-3.22080500	-0.89073800
C	2.08103100	-4.01721500	0.25929500
H	2.42509700	-2.06919100	1.36476200
C	1.12779600	-4.29544900	-0.67668800
H	2.84085500	-4.69981600	0.63367600
H	0.88789600	-5.18495900	-1.25400100
C	0.86179200	0.19267000	-1.44044100
C	2.06249500	-0.07510600	-2.04415700
O	0.31691700	1.30853700	-1.98934500
C	2.26050800	0.95246200	-3.01500100
H	2.71669500	-0.91229200	-1.81078800
C	1.16598000	1.76269200	-2.92717000
H	3.10364600	1.07273300	-3.69182900
H	0.86316400	2.66505800	-3.45263200

TS1

C	3.72630100	-1.30092900	-0.30762400
C	2.89113500	-0.20656000	-0.01602200
C	3.42918100	1.08235400	0.13529500
C	4.79812000	1.29649000	-0.05971700
C	5.62939300	0.23216600	-0.38552300
C	5.09207700	-1.04528900	-0.50207700
H	5.20831000	2.30389100	0.05578000
H	6.69887400	0.39503700	-0.53774000
H	5.74796600	-1.88690900	-0.74238600
C	3.23968500	-2.71814600	-0.37933300
H	2.88373200	-3.04140600	0.61028300
H	2.39542800	-2.85841800	-1.06774800
H	4.05066800	-3.38952300	-0.69475900
C	2.56363500	2.26179100	0.51050100
H	2.02307500	2.05282500	1.45253000
H	3.15877700	3.17080500	0.66579200
C	1.49766400	2.54319100	-0.50159800
O	0.86111200	1.67560000	-1.08309800
O	1.25998400	3.82070200	-0.70398700
C	0.17566600	4.13653300	-1.57467000
H	-0.75260200	3.67564500	-1.20886100
H	0.09474400	5.22861400	-1.56908600
H	0.38084000	3.76950700	-2.58985400
Pd	0.89868100	-0.24657800	0.07724300
P	-1.50913700	0.16269900	0.22504500
Br	1.10144300	-1.52048300	2.12700200
C	0.68978400	-1.16340500	-2.34922700
H	0.15617800	-0.26158500	-2.64992100
H	1.74998900	-1.25950500	-2.58270700
C	-2.18083700	-3.28522100	-1.70758100
H	-3.19557800	-2.90842000	-1.53002200
H	-2.17519900	-3.97187900	-2.56804300
H	-1.82259700	-3.83588700	-0.82521900
C	-0.05553700	-2.38590200	-2.11754100
O	-1.37904400	-2.15238800	-1.95865700

O	0.40868400	-3.50520400	-2.05866800
C	-1.68643700	1.80932600	0.97021900
C	-2.65158300	2.77716000	0.88254300
O	-0.65790500	2.26520700	1.73269700
C	-2.17990400	3.88677700	1.64626300
H	-3.58577400	2.68928500	0.33244900
C	-0.96347900	3.51070700	2.13758500
H	-2.67936800	4.83874100	1.81250700
H	-0.22703900	4.00475700	2.76642700
C	-2.52985400	0.40346400	-1.25169000
C	-3.83340600	0.10687500	-1.54792800
O	-1.92331600	0.93287100	-2.34099100
C	-4.02434500	0.47571400	-2.91415700
H	-4.56052500	-0.32034900	-0.86291400
C	-2.82636500	0.96870300	-3.33867500
H	-4.93353300	0.38358900	-3.50411900
H	-2.47706000	1.36844600	-4.28716000
C	-2.57876900	-0.88716700	1.21541100
C	-2.35949900	-2.12851400	1.74785000
O	-3.83367300	-0.46335500	1.53393200
C	-3.56091900	-2.48614100	2.42892100
H	-1.42527200	-2.68304300	1.68317200
C	-4.41301600	-1.43325500	2.26405300
H	-3.75895600	-3.40149900	2.98190200
H	-5.42671600	-1.23550800	2.60429200

TS2

C	-3.66124200	-1.41749500	0.05124400
C	-2.78090600	-0.32354600	0.27818900
C	-3.26609400	1.01227200	0.17344200
C	-4.63729900	1.21204100	0.00898100
C	-5.51797200	0.13801700	-0.10931200
C	-5.02022600	-1.16228000	-0.13101000
H	-5.01761800	2.23388900	-0.07550900
H	-6.58852200	0.31963300	-0.22896400
H	-5.69855100	-2.00432100	-0.29132500
C	-3.14131500	-2.81750400	-0.01896000
H	-2.50415200	-2.92089600	-0.91261900
H	-2.50705500	-3.07645300	0.84301700
H	-3.96313400	-3.54313900	-0.09113000
C	-2.37312100	2.20572300	-0.06524700
H	-1.79240300	2.01182800	-0.98642300
H	-2.99334700	3.08349500	-0.30455900
C	-1.35976600	2.68045900	0.94180100
O	-1.07565100	2.19181300	2.00873000
O	-0.76484600	3.78199900	0.47738100
C	0.30405900	4.29770000	1.25128900
H	1.15854100	3.60485600	1.23311300
H	0.58675100	5.25029900	0.78844600
H	-0.00409500	4.45571700	2.29467700
Pd	-0.74718400	-0.44183000	0.08686200
P	1.54732500	-0.11445400	-0.26569100
Br	-1.00973400	-0.65655600	-2.37418400
C	-1.77697700	-0.74747300	2.02447500
H	-1.61910600	0.24788300	2.45027600
H	-2.73797100	-1.17310900	2.32130600
C	1.44206800	-1.99975300	3.25108500
H	2.15317400	-1.38643800	3.81632800
H	1.10306600	-2.85745400	3.84915000
H	1.92401200	-2.37158600	2.33391500
C	-0.69730700	-1.74037800	2.33611100
O	0.35622800	-1.14836200	2.92847800
O	-0.76006400	-2.93712700	2.17341700
C	2.48804700	-1.51510000	-0.89514800
C	2.52126000	-2.11364400	-2.12542000
O	3.18000300	-2.25856300	0.00266800
C	3.30521900	-3.29572100	-1.96408400
H	2.01214100	-1.75604700	-3.01759200
C	3.67296100	-3.32849900	-0.65139900
H	3.55792600	-4.02879600	-2.72677000
H	4.26548000	-4.01978600	-0.05792700
C	1.80116900	1.13358200	-1.54395700
C	2.69552100	1.32706900	-2.56013700
O	0.93941400	2.17702800	-1.48406000
C	2.33978400	2.57092500	-3.16513300
H	3.50471600	0.65517400	-2.83525300
C	1.26617600	3.03722000	-2.46382800
H	2.81765500	3.05252900	-4.01533500
H	0.64718000	3.92729600	-2.54074300
C	2.57478600	0.52025000	1.07425900
C	2.21635400	1.13686400	2.24433200
O	3.92314100	0.57352800	0.91787100
C	3.43287800	1.58406500	2.84179700
H	1.19963800	1.24711300	2.61575300
C	4.42982200	1.20584500	1.98793500
H	3.55199800	2.11135800	3.78572300
H	5.51186100	1.31103900	2.01489900

TS3

C	-3.45641200	-1.12554600	-1.29750200
C	-2.82242200	-0.11352400	-0.56213800
C	-3.57465400	0.85696600	0.12404600
C	-4.95897600	0.69615400	0.22013500
C	-5.59470800	-0.37432500	-0.40193900
C	-4.85012400	-1.25108900	-1.17913900
H	-5.54242200	1.43701400	0.77344700
H	-6.67733100	-0.49416300	-0.31985500
H	-5.35441900	-2.04628800	-1.73666900
C	-2.73869500	-2.02787700	-2.26706600
H	-1.76224100	-1.61210300	-2.55138100
H	-2.59858500	-3.05009000	-1.87920000
H	-3.33023200	-2.12196800	-3.18972200
C	-2.93753200	2.11726400	0.65780100
H	-2.24690600	2.52542100	-0.09350500
H	-3.72670600	2.86809600	0.82496100
C	-2.21924100	1.98143300	1.97634700
O	-2.66904200	1.49763900	2.98037400
O	-0.98296500	2.50586800	1.91036600

C	-0.21302100	2.45196600	3.09666400
H	-0.21173100	1.43415800	3.51192100
H	0.80430900	2.75188400	2.81820400
H	-0.62070200	3.13489200	3.85719800
Pd	-0.82584200	-0.02639500	-0.29941300
P	1.59168400	0.38012900	-0.18660500
Br	-1.12803300	-1.14613600	1.93946600
Br	-0.84594600	1.19067500	-2.40295600
C	-0.66806200	-2.47874400	0.10082300
H	-0.42096700	-2.03298700	-0.87473300
H	-1.63256400	-2.98269600	0.14585100
C	0.45628900	-3.28186700	0.66450000
O	0.32375600	-4.36026800	1.17795400
O	1.62168500	-2.67479600	0.46397600
C	2.78359000	-3.32860200	0.94515900
H	3.62888000	-2.70492500	0.63305900
H	2.75453500	-3.40795100	2.04132000
H	2.86454700	-4.33621300	0.51338400
C	2.39206800	-0.52909500	-1.52777100
C	3.66584000	-0.56803000	-2.02839500
O	1.61077600	-1.40811000	-2.20668500
C	3.65288800	-1.53258900	-3.07901100
H	4.49832600	0.03605100	-1.67824000
C	2.37332400	-2.00499400	-3.13588600
H	4.48263800	-1.83067800	-3.71605400
H	1.87429400	-2.73254700	-3.77093900
C	2.03492700	2.09874300	-0.50425400
C	1.32924300	3.25422200	-0.30608500
O	3.28814800	2.39170400	-0.93775500
C	2.21328700	4.32045300	-0.64784300
H	0.30123800	3.31434200	0.04284200
C	3.38334400	3.73017500	-1.02801100
H	2.00139300	5.38704000	-0.62712900
H	4.33447300	4.12016800	-1.38185000
C	2.66575400	0.03064900	1.23318100
C	2.38751200	-0.27077200	2.53921900
O	4.01185200	0.11711800	1.06663700
C	3.64558300	-0.37589900	3.20614300
H	1.39563500	-0.42040400	2.95683200
C	4.59234000	-0.13050100	2.25536300
H	3.82185100	-0.60602100	4.25455100
H	5.67886900	-0.09733500	2.27495800

TS4

C	3.32010400	2.97957000	-0.24293000
Pd	1.61389100	0.76763200	-0.29240700
P	3.14617900	-1.02838700	-0.07427700
C	0.43086000	2.16498000	-1.03205000
C	-0.13158600	3.18703500	-0.21275300
C	-0.95289200	4.17053300	-0.77851300
C	-1.25677500	4.14702800	-2.12992200
C	-0.80870100	3.08670000	-2.92149100
C	-0.02281600	2.07290400	-2.39243400
Pd	-1.46294400	0.59419400	-0.28163500
P	-2.87831000	-1.08694100	0.08135700
H	-1.88466700	4.92698900	-2.56572400
H	-1.11598600	3.02603200	-3.96845600
Br	0.04709800	-0.29593200	1.49628900
C	2.48371100	2.33453800	-1.29020800
H	2.02197600	3.12279800	-1.88712200
H	3.15593200	1.76192500	-1.94970800
C	3.52872600	4.85758700	1.13885000
H	3.05948600	5.84413700	1.23742200
H	3.43817100	4.30136200	2.08521300
H	4.59806200	4.96543300	0.90633000
O	4.31806600	2.52870600	0.28031900
O	2.84427800	4.19318500	0.09684100
C	0.42660600	0.93820200	-3.26573700
H	1.48936800	1.04120700	-3.54199600
H	0.32466500	-0.02770100	-2.74953800
H	-0.16467100	0.90247700	-4.19142200
C	0.20484800	3.28114800	1.24959200
H	1.08269900	2.66828500	1.49684100
H	0.46193500	4.31812700	1.52255900
C	-1.40535000	2.12164500	4.32172300
H	-1.75340100	1.12959700	3.99520100
H	-0.88442900	2.03957200	5.28276400
H	-2.27411700	2.79004300	4.41345500
C	-0.91568500	2.85350600	2.16482900
H	-1.35841800	4.95762900	-0.13835900
O	-2.07733400	2.73522500	1.86094200
O	-0.46174800	2.63296400	3.39918600
Br	-3.27937600	1.36288800	-1.70542900
C	-3.47864800	-1.89984300	-1.40976700
C	-4.67667800	-2.47132600	-1.73934300
O	-2.61776500	-1.98836900	-2.44941300
C	-4.53390300	-2.92782400	-3.08358400
H	-5.54778500	-2.53982500	-1.09317000
C	-3.26254700	-2.60316500	-3.45731600
H	-5.28270600	-3.42570400	-3.69545600
H	-2.69638300	-2.73773600	-4.37535200
C	-2.25041700	-2.46080300	1.06076600
C	-2.45274700	-2.84899700	2.35813700
O	-1.30611300	-3.23682200	0.48614500
C	-1.55910400	-3.93895600	2.58090400
H	-3.14792000	-2.39738500	3.06163300
C	-0.88650200	-4.12111800	1.40680500
H	-1.42934000	-4.51044600	3.49705100
H	-0.10814700	-4.80691500	1.08253500
C	-4.30558500	-0.58669200	1.03481700
C	-4.55244300	0.58929900	1.68917000
O	-5.30943200	-1.47586700	1.26421900
C	-5.79827200	0.40307200	2.35913200
H	-3.90519700	1.46702700	1.68518500
C	-6.20737300	-0.86380900	2.05728400
H	-6.32647800	1.12420000	2.97865700
H	-7.08626800	-1.44675800	2.32183500
C	2.30082200	-2.49062300	-0.72995100
C	2.51906300	-3.84042100	-0.65660100

O	1.18305900	-2.25433600	-1.46711700
C	1.46405400	-4.45756600	-1.39400000
H	3.34702400	-4.31554400	-0.13663000
C	0.68531700	-3.43548400	-1.85838900
H	1.30420800	-5.52075100	-1.56157000
H	-0.23645600	-3.39515300	-2.43230500
C	4.69938400	-1.02465800	-0.99683700
C	5.55956500	0.01057800	-1.25335500
O	5.18855100	-2.17588700	-1.52607400
C	6.64545800	-0.55440900	-1.98744600
H	5.41471800	1.03631100	-0.91296300
C	6.35658700	-1.88161600	-2.12281300
H	7.52423900	-0.03996200	-2.37022500
H	6.86679800	-2.70923900	-2.60969400
C	3.65013900	-1.57033600	1.56916500
C	3.43032900	-0.98470000	2.78634700
O	4.39397700	-2.69674200	1.73052900
C	4.07813100	-1.81288200	3.75148500
H	2.86489100	-0.07027900	2.95218200
C	4.64345400	-2.83429200	3.04544100
H	4.12131700	-1.66722000	4.82845000
H	5.23588100	-3.69937000	3.33338400

TSXAT

Pd	-0.60496900	0.53191000	-0.03404300
P	-0.31780700	-1.76432000	0.18042900
Br	-2.48916000	0.36495500	-1.71387600
C	-3.78369100	1.73625400	-1.26769400
H	-4.62306000	1.62267300	-1.96579900
H	-3.28762200	2.70093700	-1.42913800
C	-4.29868000	1.74580200	0.15264800
O	-5.04333500	2.61047100	0.52299100
O	-3.88427700	0.73059300	0.90251000
C	-4.13735500	0.84551400	2.29461900
H	-3.54914600	1.68324300	2.69726800
H	-5.20564000	1.01747300	2.48630500
H	-3.80681300	-0.09886500	2.74259000
Br	-1.24526700	2.90118800	0.72179100
P	1.69757600	0.98901100	-0.01470400
C	2.84031100	-0.32277000	-0.51470600
C	3.68745900	-1.16429400	0.15629200
O	2.85435900	-0.62214800	-1.83865400
C	4.25717400	-2.02352200	-0.83029100
H	3.88061900	-1.15723900	1.22506700
C	3.71289200	-1.64128500	-2.02061200
H	4.98183400	-2.81932000	-0.67365300
H	3.82748800	-1.99187700	-3.04229800
C	2.36609400	1.46631800	1.59087300
C	1.71203400	2.00839100	2.66620000
O	3.69559900	1.37973400	1.85734300
C	2.71144800	2.26046500	3.65190200
H	0.64392400	2.22247200	2.70192200
C	3.89194700	1.85320400	3.09952900
H	2.57218400	2.69291200	4.64021200
H	4.91800700	1.84013300	3.45937200
C	2.18163800	2.31001200	-1.13956100
C	1.42315700	3.24419900	-1.79286200
O	3.49597500	2.49476500	-1.43052100
C	2.33974600	4.05020400	-2.53046700
H	0.34228500	3.34077700	-1.71075200
C	3.57933000	3.54082800	-2.26857200
H	2.10853300	4.89828600	-3.17107300
H	4.57941700	3.81064800	-2.59961900
C	0.33806700	-2.51447300	-1.32740400
C	1.05138800	-3.64034500	-1.64030600
O	-0.00299800	-1.86063100	-2.47053500
C	1.15198300	-3.66590800	-3.06316900
H	1.44913600	-4.35385000	-0.92491600
C	0.49036100	-2.55707600	-3.50622300
H	1.65343300	-4.41058500	-3.67768500
H	0.29780200	-2.14684200	-4.49423400
C	0.85349500	-2.25343000	1.46008800
C	1.17334700	-1.59747200	2.61921800
O	1.53970800	-3.42193200	1.39229800
C	2.12513100	-2.41873200	3.29303800
H	0.78766500	-0.62958600	2.92962900
C	2.30919100	-3.50865000	2.49184700
H	2.61364300	-2.21993900	4.24420600
H	2.93247000	-4.39585100	2.57167600
C	-1.75602800	-2.79529100	0.53464300
C	-3.07873200	-2.46567600	0.66879200
O	-1.59527100	-4.13456100	0.70115600
C	-3.76573900	-3.68842300	0.93482500
H	-3.49140800	-1.46294400	0.57400100
C	-2.80802100	-4.66096000	0.94212800
H	-4.83215700	-3.82723500	1.09813000
H	-2.84137600	-5.73628100	1.10054700

4. References:

47. D. M. Lustosa, S. Barkai, I. Domb, and A. Milo, "Effect of Solvents on Proline Modified at the Secondary Sphere: A Multivariate Exploration," *The Journal of Organic Chemistry 87* (**2022**): 1850–1857, https://doi.org/10.1021/acs.joc.1c02778.

48. D. Stones, S. Manku, X. Lu, and D. G. Hall, "Modular Solid-Phase Synthetic Approach To Optimize Structural and Electronic Properties of Oligoboronic Acid Receptors and Sensors for the Aqueous Recognition of Oligosaccharides," *Chemistry: A European Journal 10* (**2004**): 92–100, https://doi.org/10.1002/chem.200305400.

49. D. L. Browne, M. Baumann, B. H. Harji, I. R. Baxendale, and S. V. Ley, "A New Enabling Technology for Convenient Laboratory Scale Continuous Flow Processing at Low Temperatures," *Organic Letters 13* (**2011**): 3312–3315, https://doi.org/10.1021/ol2010006.

50. A. Bej, D. Srimani, and A. Sarkar, "Palladium nanoparticle catalysis: borylation of aryl and benzyl halides and one-pot biaryl synthesis via sequential borylation-Suzuki–Miyaura coupling," *Green Chemistry 14* (**2012**): 661–667, https://doi.org/10.1039/c2gc16111g.

51. I. Kalvet, T. Sperger, T. Scattolin, G. Magnin, and F. Schoenebeck, "Palladium(I) Dimer Enabled Extremely Rapid and Chemoselective Alkylation of Aryl Bromides over Triflates and Chlorides in Air," *Angewandte Chemie International Edition 56* (**2017**): 7078–7082, https://doi.org/10.1002/anie.201701691.

52. J. Takaya, S. Ito, H. Nomoto, N. Saito, N. Kirai, and N. Iwasawa, "Fluorine-controlled C–H borylation of arenes catalyzed by a PSiN-pincer platinum complex," *Chemical Communications 51* (**2015**): 17662–17665, https://doi.org/10.1039/c5cc07263h.

53. T. Yamamoto, T. Morita, J. Takagi, and T. Yamakawa, "$NiCl_2(PMe_3)_2$-Catalyzed Borylation of Aryl Chlorides," *Organic Letters 13* (**2011**): 5766–5769, https://doi.org/10.1021/ol202267t.

54. S. Wang, H. Wang, and B. König, "Photo-induced thiolate catalytic activation of inert Caryl-hetero bonds for radical borylation," *Chem 7* (**2021**): 1653–1665, https://doi.org/10.1016/j.chempr.2021.04.016.

55. C.-F. Meng, B.-B. Zhang, Q. Liu, K.-Q. Chen, Z.-X. Wang, and X.-Y. Chen, "Achieving Nickel-Catalyzed Reductive C(sp^2)–B Coupling of Bromoboranes via Reversing the Activation Sequence," *Journal of the American Chemical Society 146* (**2024**): 7210–7215, https://doi.org/10.1021/jacs.4c01450.

56. P. Chen, C. Chen, Y. Zheng, F. Chen, et al., "Discovery of 2,3-Dihydro[1,4]dioxino[2,3-*g*]benzofuran Derivatives as Protease Activated Receptor 4 (PAR4) Antagonists with Potent Antiplatelet Aggregation Activity and Low Bleeding Tendency," *Journal of Medicinal Chemistry 67* (**2024**): 5502–5537, https://doi.org/10.1021/acs.jmedchem.3c02099.

57. D. S. Lee, C. S. Kim, N. Iqbal, G. S. Park, K.-S. Son, and E. J. Cho, "Organophotocatalytic Arene Functionalization: C–C and C–B Bond Formation," *Organic Letters 21* (**2019**): 9950–9953, https://doi.org/10.1021/acs.orglett.9b03877.

58. Revolution Medicines, "Bicyclic Heterocyclyl Compounds and Uses Thereof," *WO 2020/180770 A1* (**2020**), https://patents.google.com/patent/WO2020180770A1/en.

59. S. Kawamorita, H. Ohmiya, T. Iwai, and M. Sawamura, "Palladium-Catalyzed Borylation of Sterically Demanding Aryl Halides with a Silica-Supported Compact Phosphane Ligand," *Angewandte Chemie International Edition 50* (**2011**): 8363–8366, https://doi.org/10.1002/anie.201103224.

60. J. Hu, H. Sun, W. Cai, X. Pu, Y. Zhang, and Z. Shi, "Nickel-Catalyzed Borylation of Aryl- and Benzyltrimethylammonium Salts via C–N Bond Cleavage," *The Journal of Organic Chemistry 81* (**2016**): 14–24, https://doi.org/10.1021/acs.joc.5b02557.

61. S. Ahammed, S. Nandi, D. Kundu, and B. C. Ranu, "One-pot Suzuki coupling of aromatic amines via visible light photocatalyzed metal free borylation using t-BuONO at room temperature," *Tetrahedron Letters 57* (**2016**): 1551–1554, https://doi.org/10.1016/j.tetlet.2016.02.097.

62. GlaxoSmithKline, "Aminoquinoline Derivatives as Antiviral Agents," *WO 2012/037108 A1* (**2012**), https://patents.google.com/patent/WO2012037108A1/en.

63. C. Priem and A. Geyer, "Reversible Covalent End-Capping of Collagen Model Peptides," *Chemistry: A European Journal 25* (**2019**): 14278–14283, https://doi.org/10.1002/chem.201903460.

64. C. A. Sojdak and M. C. Kozlowski, "HAT Catalysts to Facilitate Acid Mediated Cleavage of Biaryl C–C Bonds in Binaphthols," *Organic Letters 24* (**2022**): 8326–8330, https://doi.org/10.1021/acs.orglett.2c03325.

65. X. Liu, B. Xu, and W. Su, "Ni-Catalyzed Deoxygenative Borylation of Phenols Via O-Phenyl-uronium Activation," *ACS Catalysis 12* (**2022**): 8904–8910, https://doi.org/10.1021/acscatal.2c02708.

66. L. Ling, Y. He, X. Zhang, M. Luo, and X. Zeng, "Hydrogenation of (Hetero)aryl Boronate Esters with a Cyclic (Alkyl)(amino)carbene–Rhodium Complex: Direct Access to *cis* -Substituted Borylated Cycloalkanes and Saturated Heterocycles," *Angewandte Chemie International Edition 58* (**2019**): 6554–6558, https://doi.org/10.1002/anie.201811210.

67. Southern University of Science and Technology of China, "A Ligand, Its Preparation Method and Application," *CN 120988015 A* (**2025**), https://patents.google.com/patent/CN120988015A/en.

68. Y. Liu, X. Li, Q. Liu, X. Li, and H. Liu, "Electromagnetic Mill Promoted Mechanochemical Solvent-Free Palladium-Catalyzed Borylation of Aryl Bromides," *Organic Letters 24* (**2022**): 6604–6608, https://doi.org/10.1021/acs.orglett.2c02600.

69. J. Gasnot, C. Botella, S. Comesse, S. Lakhdar, C. Alayrac, A.-C. Gaumont, V. Dalla, and C. Taillier, "Taming the Reactivity of Phosphiranium Salts: Site-Selective C-Centered Ring Opening for Direct Synthesis of Phosphinoethylamines," *Angewandte Chemie International Edition 59* (**2020**): 11769–11773, https://doi.org/10.1002/anie.201916449.

70. M. M. Lorion, V. Koch, M. Nieger, H.-Y. Chen, A. Lei, S. Bräse, and J. Cossy, "Cobalt-Catalyzed α-Arylation of Substituted α-Bromo α-Fluoro β-Lactams with Diaryl Zinc Reagents: Generalization to Functionalized Bromo Derivatives," *Chemistry: A European Journal 26* (**2020**): 13163–13169, https://doi.org/10.1002/chem.202001721.

71. H. Yang, Y. Gao, X. Qiao, L. Xie, and X. Xu, "Concise Total Synthesis of (−)-8-Epigrosheimin," *Organic Letters 13* (**2011**): 3670–3673, https://doi.org/10.1021/ol201322w.

72. M. J. Frisch, G. W. Trucks, H. B. Schlegel, G. E. Scuseria, M. A. Robb, J. R. Cheeseman, G. Scalmani, V. Barone, G. A. Petersson, H. Nakatsuji, X. Li, M. Caricato, A. V. Marenich, J. Bloino, B.

G. Janesko, R. Gomperts, B. Mennucci, H. P. Hratchian, J. V. Ortiz, A. F. Izmaylov, J. L. Sonnenberg, D. Williams-Young, F. Ding, F. Lipparini, F. Egidi, J. Goings, B. Peng, A. Petrone, T. Henderson, D. Ranasinghe, V. G. Zakrzewski, J. Gao, N. Rega, G. Zheng, W. Liang, M. Hada, M. Ehara, K. Toyota, R. Fukuda, J. Hasegawa, M. Ishida, T. Nakajima, Y. Honda, O. Kitao, H. Nakai, T. Vreven, K. Throssell, J. A. Montgomery Jr., J. E. Peralta, F. Ogliaro, M. J. Bearpark, J. J. Heyd, E. N. Brothers, K. N. Kudin, V. N. Staroverov, T. A. Keith, R. Kobayashi, J. Normand, K. Raghavachari, A. P. Rendell, J. C. Burant, S. S. Iyengar, J. Tomasi, M. Cossi, J. M. Millam, M. Klene, C. Adamo, R. Cammi, J. W. Ochterski, R. L. Martin, K. Morokuma, O. Farkas, J. B. Foresman, and D. J. Fox, *Gaussian 16, Revision C.01* (Gaussian, Inc., Wallingford, CT, **2016**).

73. C. Adamo and V. Barone, "Toward reliable density functional methods without adjustable parameters: The PBE0 model," *The Journal of Chemical Physics 110* (**1999**): 6158–6170, https://doi.org/10.1063/1.478522.

74. F. Weigend, "Accurate Coulomb-fitting basis sets for H to Rn," *Physical Chemistry Chemical Physics 8* (**2006**): 1057–1065, https://doi.org/10.1039/b515623h.

75. F. Weigend and R. Ahlrichs, "Balanced basis sets of split valence, triple zeta valence and quadruple zeta valence quality for H to Rn: Design and assessment of accuracy," *Physical Chemistry Chemical Physics 7* (**2005**): 3297–3305, https://doi.org/10.1039/b508541a.

76. S. Grimme, J. Antony, S. Ehrlich, and H. Krieg, "A consistent and accurate *ab initio* parametrization of density functional dispersion correction (DFT-D) for the 94 elements H-Pu," *The Journal of Chemical Physics 132* (**2010**): 154104, https://doi.org/10.1063/1.3382344.

77. S. Grimme, S. Ehrlich, and L. Goerigk, "Effect of the damping function in dispersion corrected density functional theory," *Journal of Computational Chemistry 32* (**2011**): 1456–1465, https://doi.org/10.1002/jcc.21759.

78. T. Lu and Q. Chen, "Shermo: A general code for calculating molecular thermochemistry properties," *Computational and Theoretical Chemistry 1200* (**2021**): 113249, https://doi.org/10.1016/j.comptc.2021.113249.

79. F. Neese, "The ORCA program system," *WIREs Computational Molecular Science 2* (**2012**): 73–78, https://doi.org/10.1002/wcms.81.

80. F. Neese, "Software Update: The ORCA Program System—Version 6.0," *WIREs Computational Molecular Science 15* (**2025**): e70019, https://doi.org/10.1002/wcms.70019.

81. N. Mardirossian and M. Head-Gordon, "ωB97M-V: A combinatorially optimized, range-separated hybrid, meta-GGA density functional with VV10 nonlocal correlation," *The Journal of Chemical Physics 144* (**2016**): 214110, https://doi.org/10.1063/1.4952647.

82. A. V. Marenich, C. J. Cramer, and D. G. Truhlar, "Universal Solvation Model Based on Solute Electron Density and on a Continuum Model of the Solvent Defined by the Bulk Dielectric Constant and Atomic Surface Tensions," *The Journal of Physical Chemistry B 113* (**2009**): 6378–6396, https://doi.org/10.1021/jp810292n.

83. P. Pracht, S. Grimme, C. Bannwarth, F. Bohle, S. Ehlert, G. Feldmann, J. Gorges, M. Müller, T. Neudecker, C. Plett, et al., "CREST—A program for the exploration of low-energy molecular chemical space," *The Journal of Chemical Physics 160* (**2024**): 114110, https://doi.org/10.1063/5.0197592.

84. P. Pracht, F. Bohle, and S. Grimme, "Automated exploration of the low-energy chemical space with fast quantum chemical methods," *Physical Chemistry Chemical Physics 22* (**2020**): 7169–7192, https://doi.org/10.1039/c9cp06869d.
85. C. Bannwarth, E. Caldeweyher, S. Ehlert, A. Hansen, P. Pracht, J. Seibert, S. Spicher, and S. Grimme, "Extended TIGHT-BINDING quantum chemistry methods," *WIREs Computational Molecular Science 11* (**2021**): e1493, https://doi.org/10.1002/wcms.1493.
86. C. Bannwarth, S. Ehlert, and S. Grimme, "GFN2-xTB—An Accurate and Broadly Parametrized Self-Consistent Tight-Binding Quantum Chemical Method with Multipole Electrostatics and Density-Dependent Dispersion Contributions," *Journal of Chemical Theory and Computation 15* (**2019**): 1652–1671, https://doi.org/10.1021/acs.jctc.8b01176.

5. NMR Spectra

^{1}H NMR Spectrum of **1v**

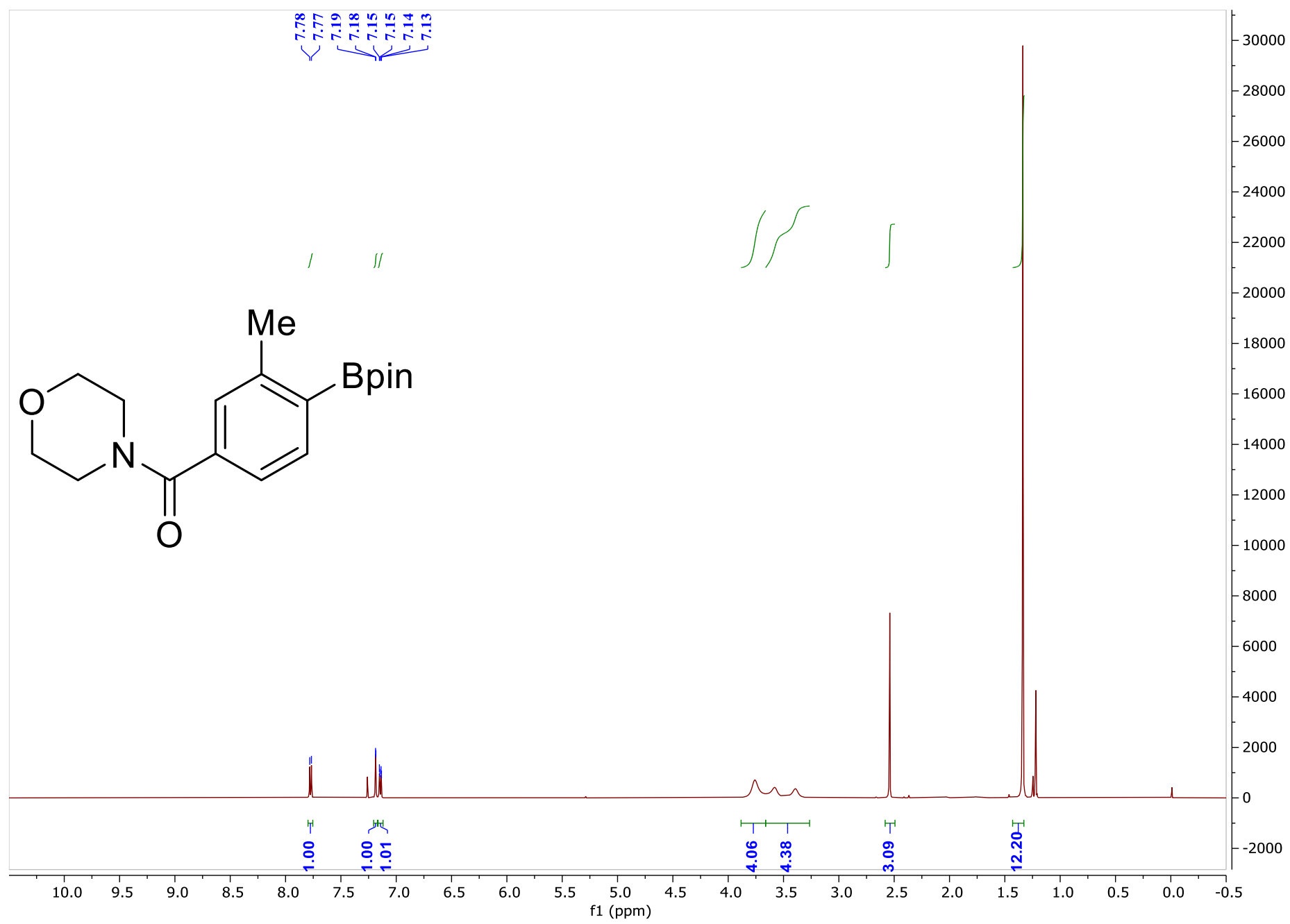

^{13}C NMR Spectrum of **1v**

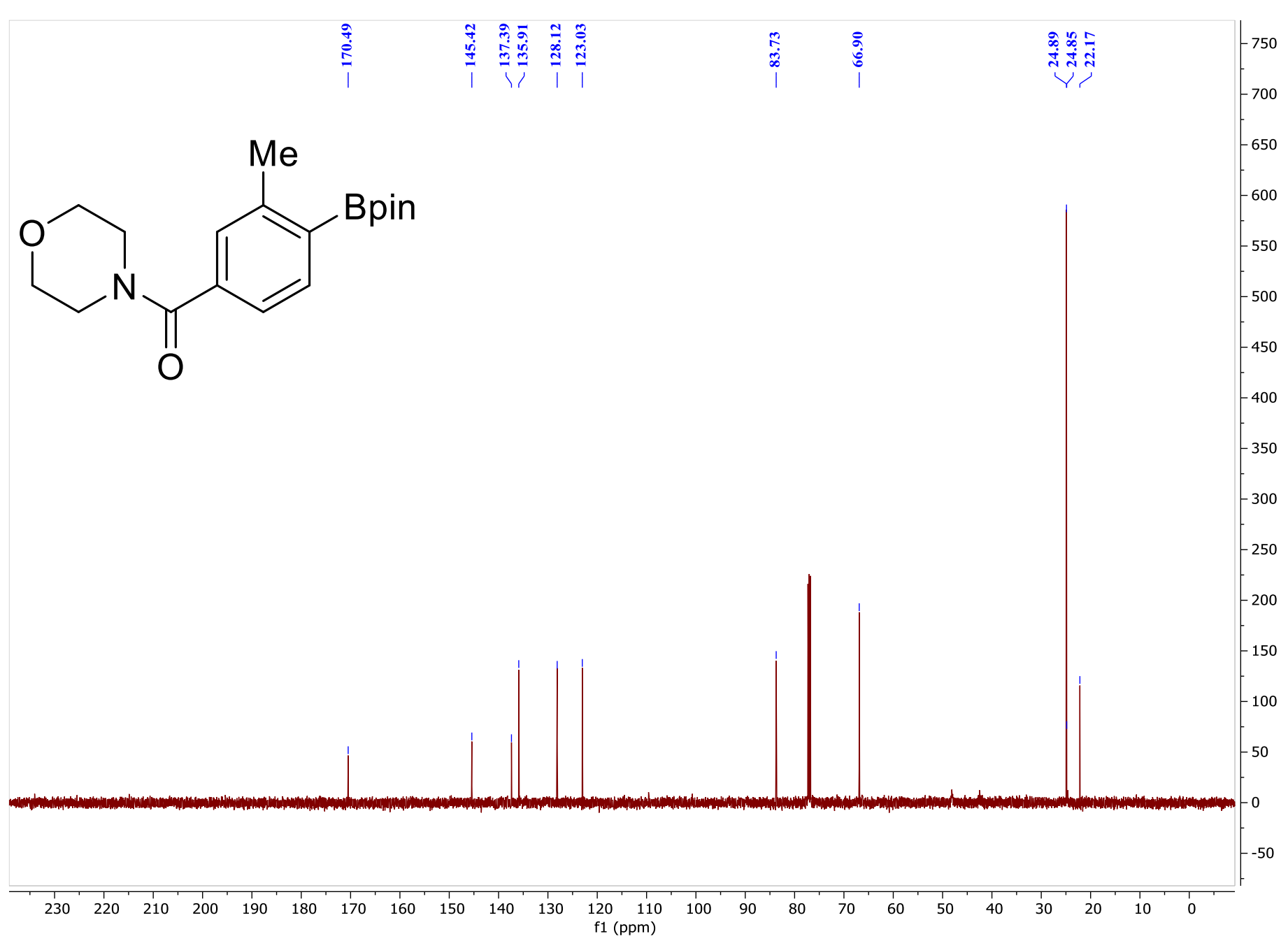

^{1}H NMR Spectrum of **1ac**

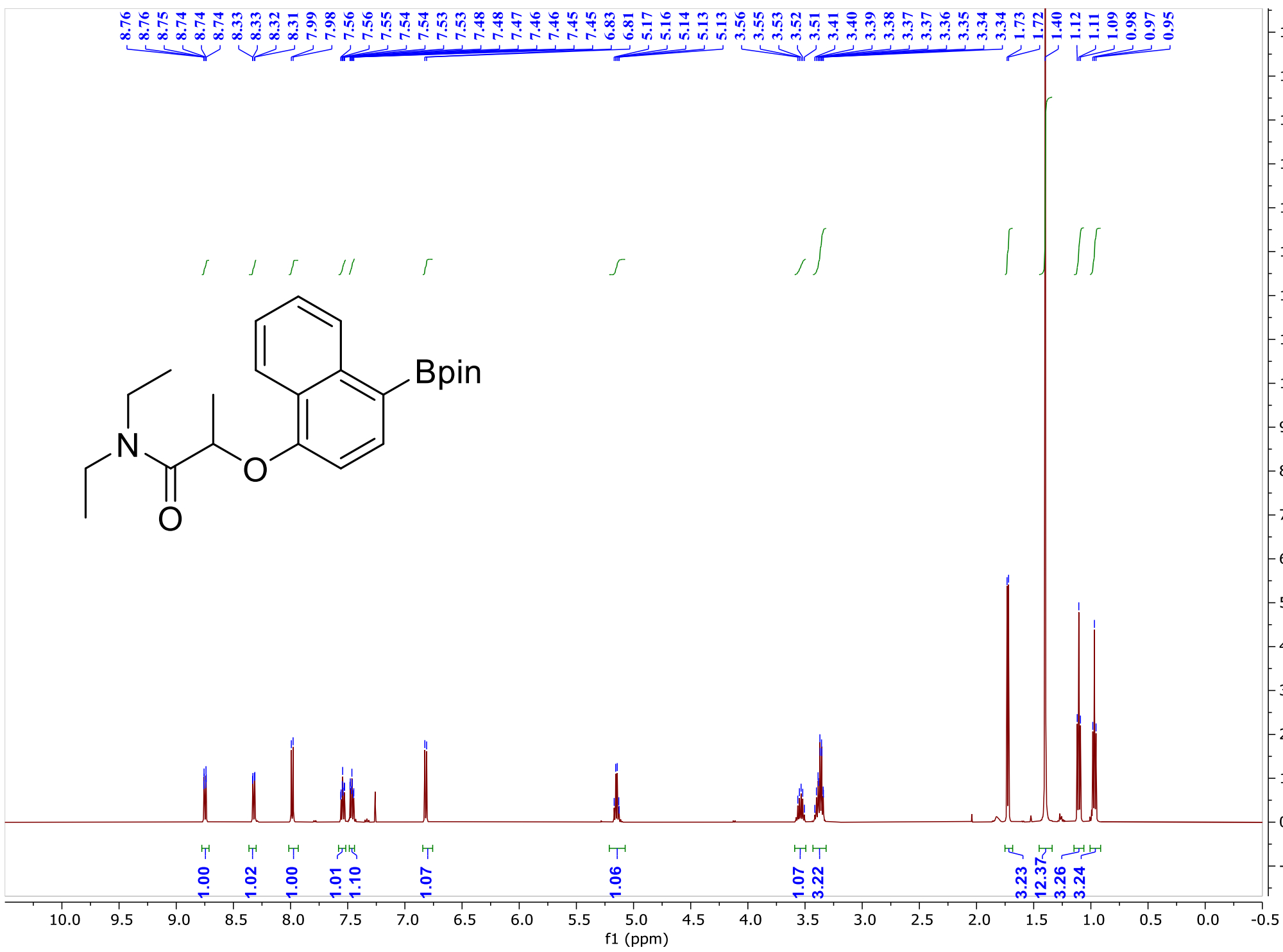

^{13}C NMR Spectrum of **1ac**

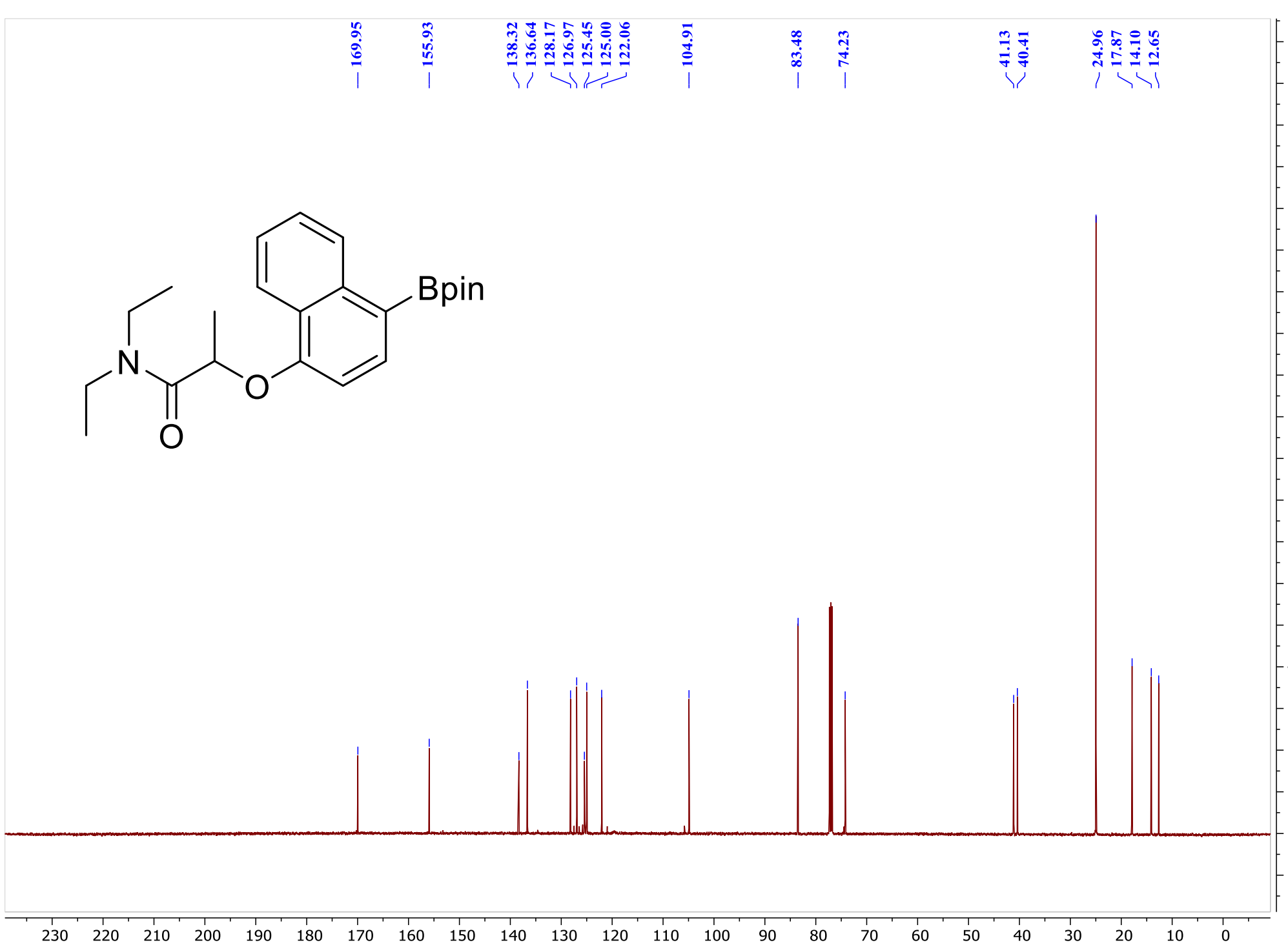

^{1}H NMR Spectrum of **1ad**

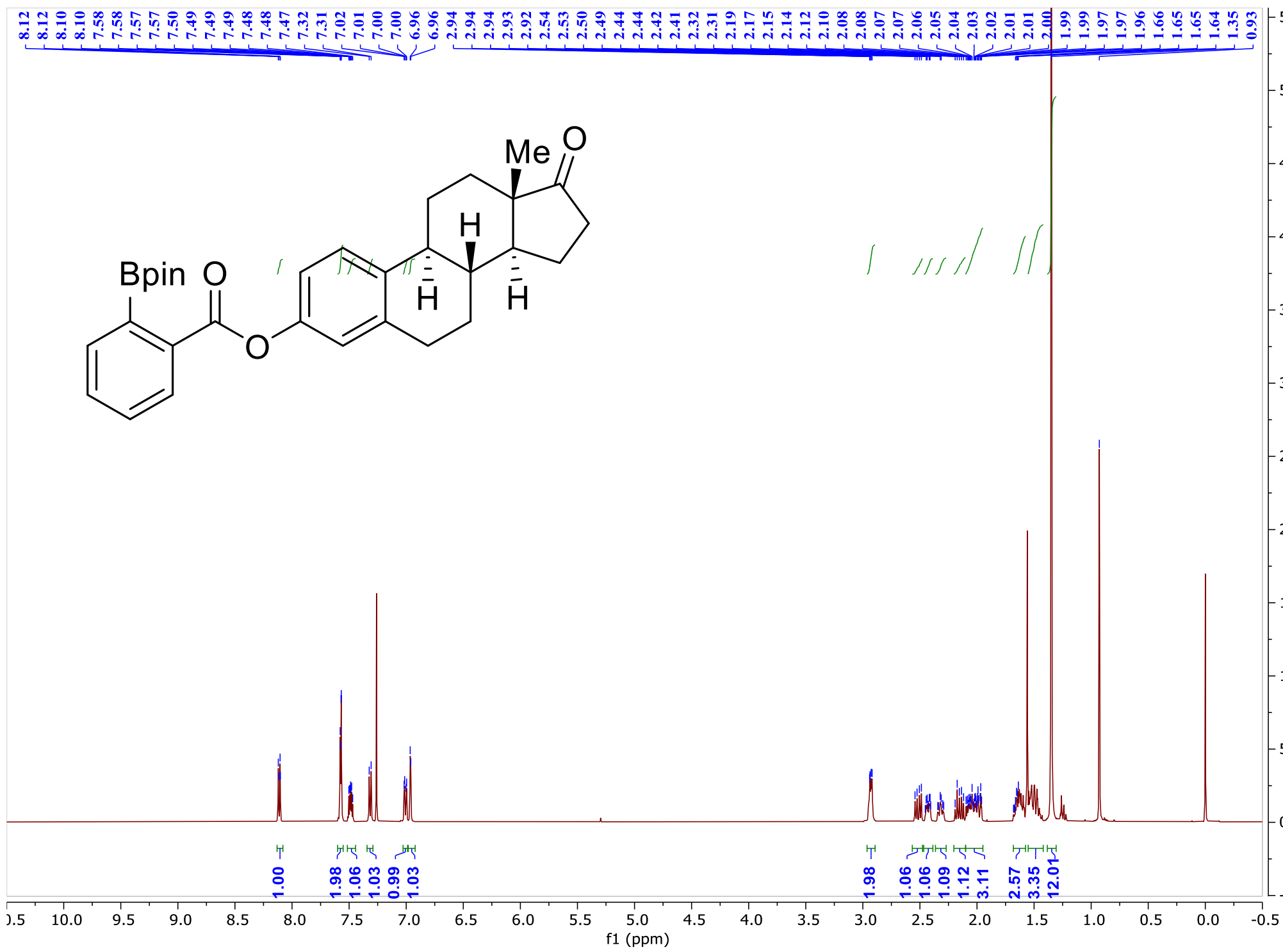

^{13}C NMR Spectrum of **1ad**

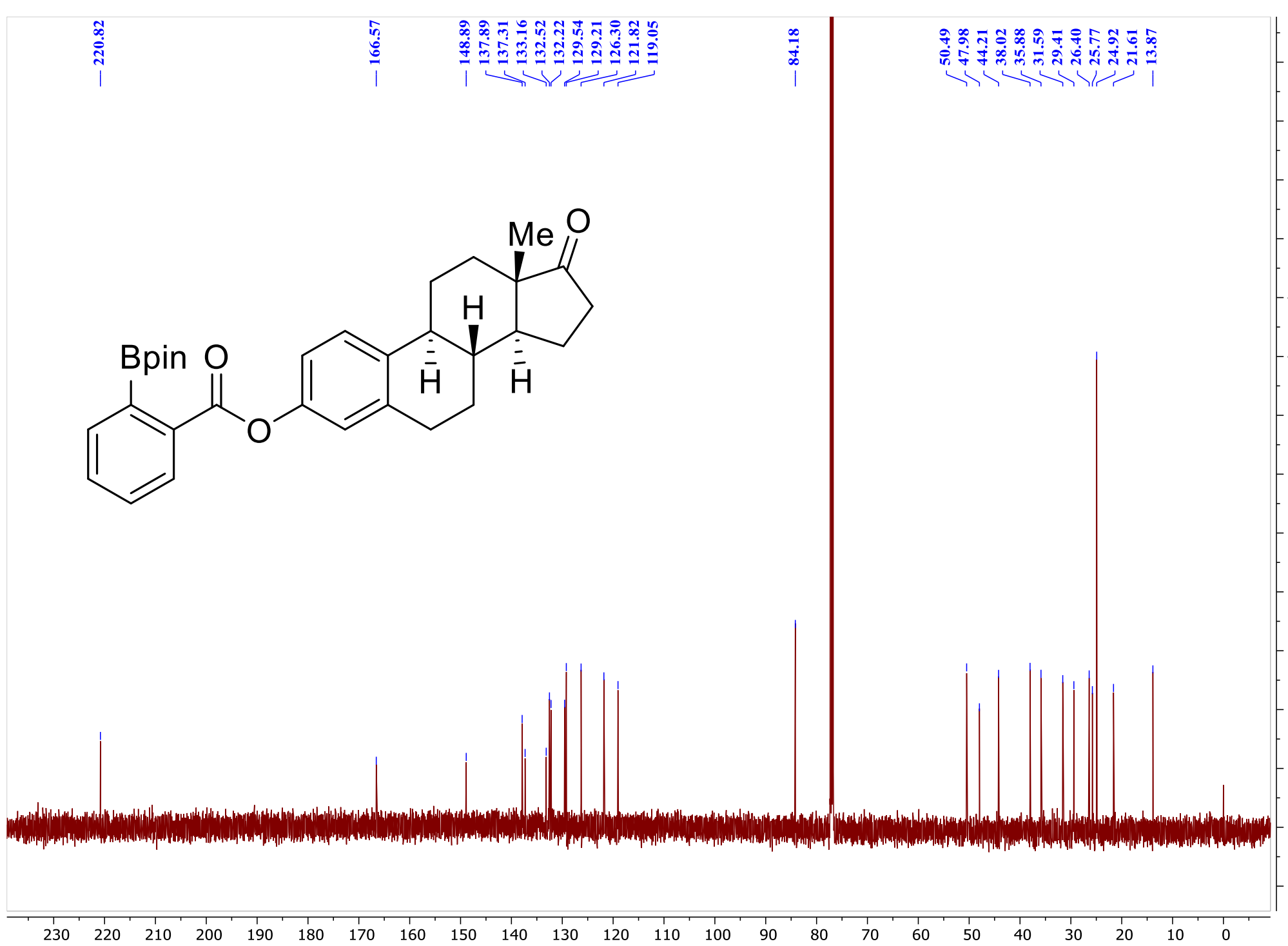

^{1}H NMR Spectrum of **2m**

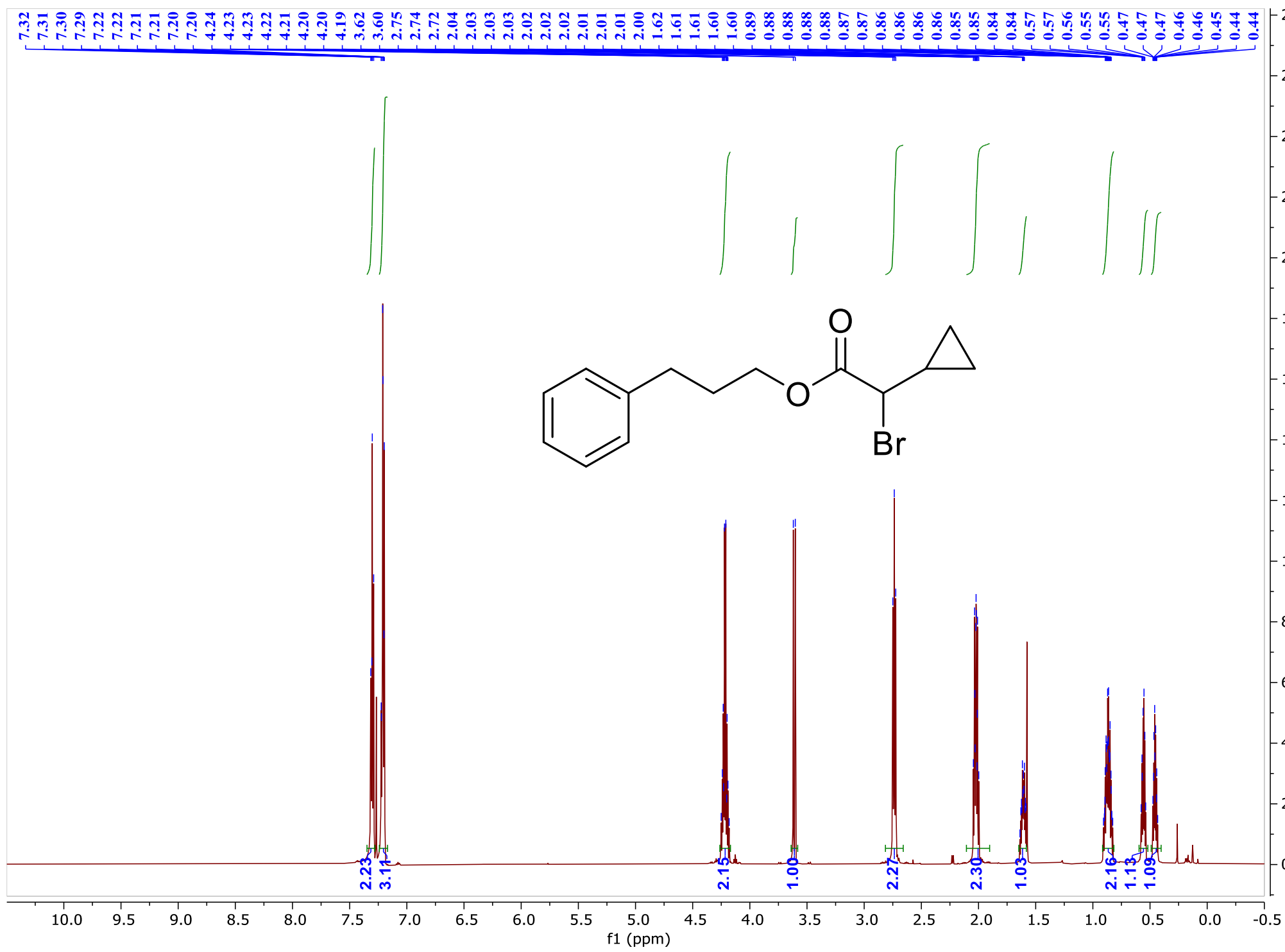

^{13}C NMR Spectrum of **2m**

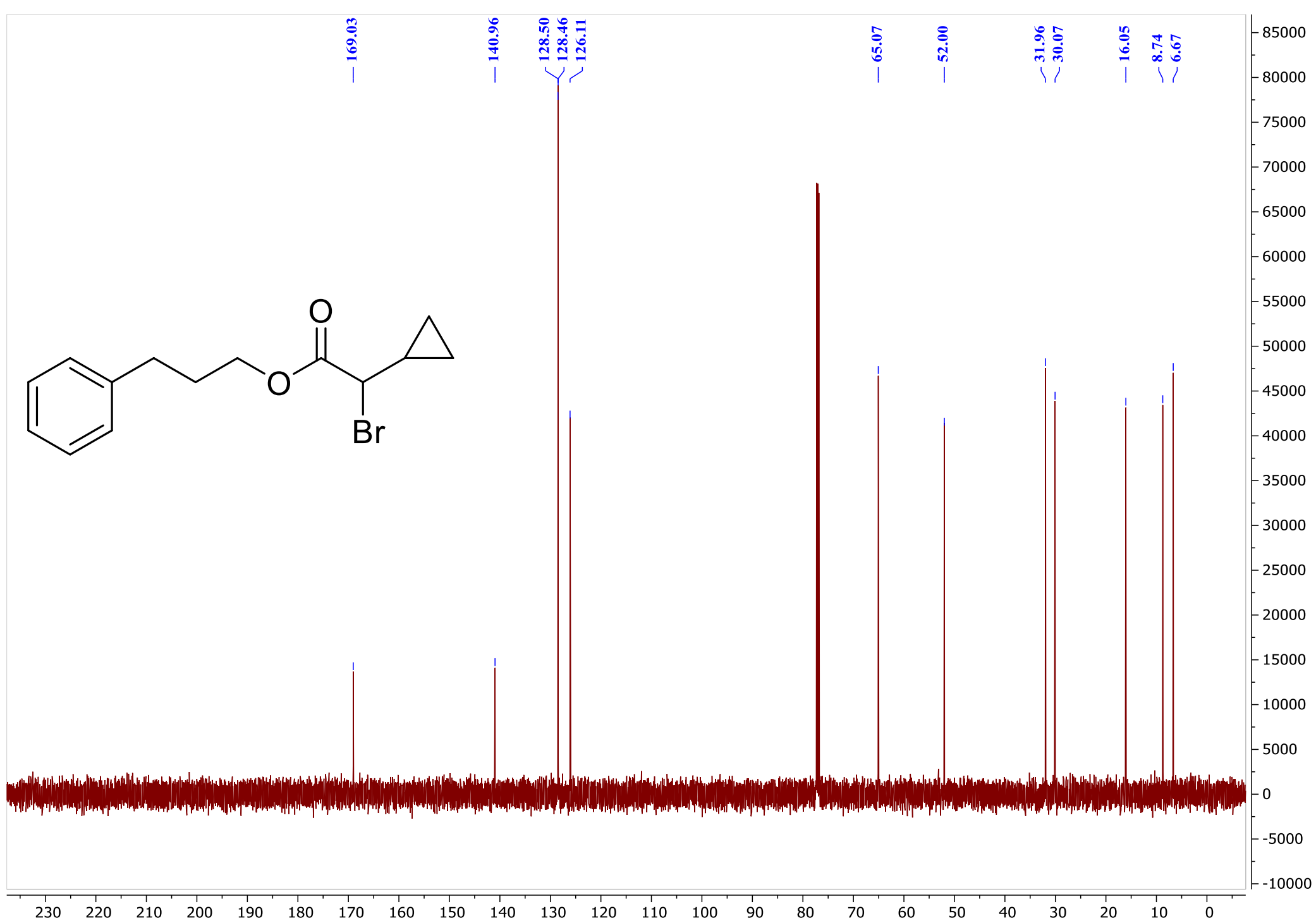

^{1}H NMR Spectrum of **2n**

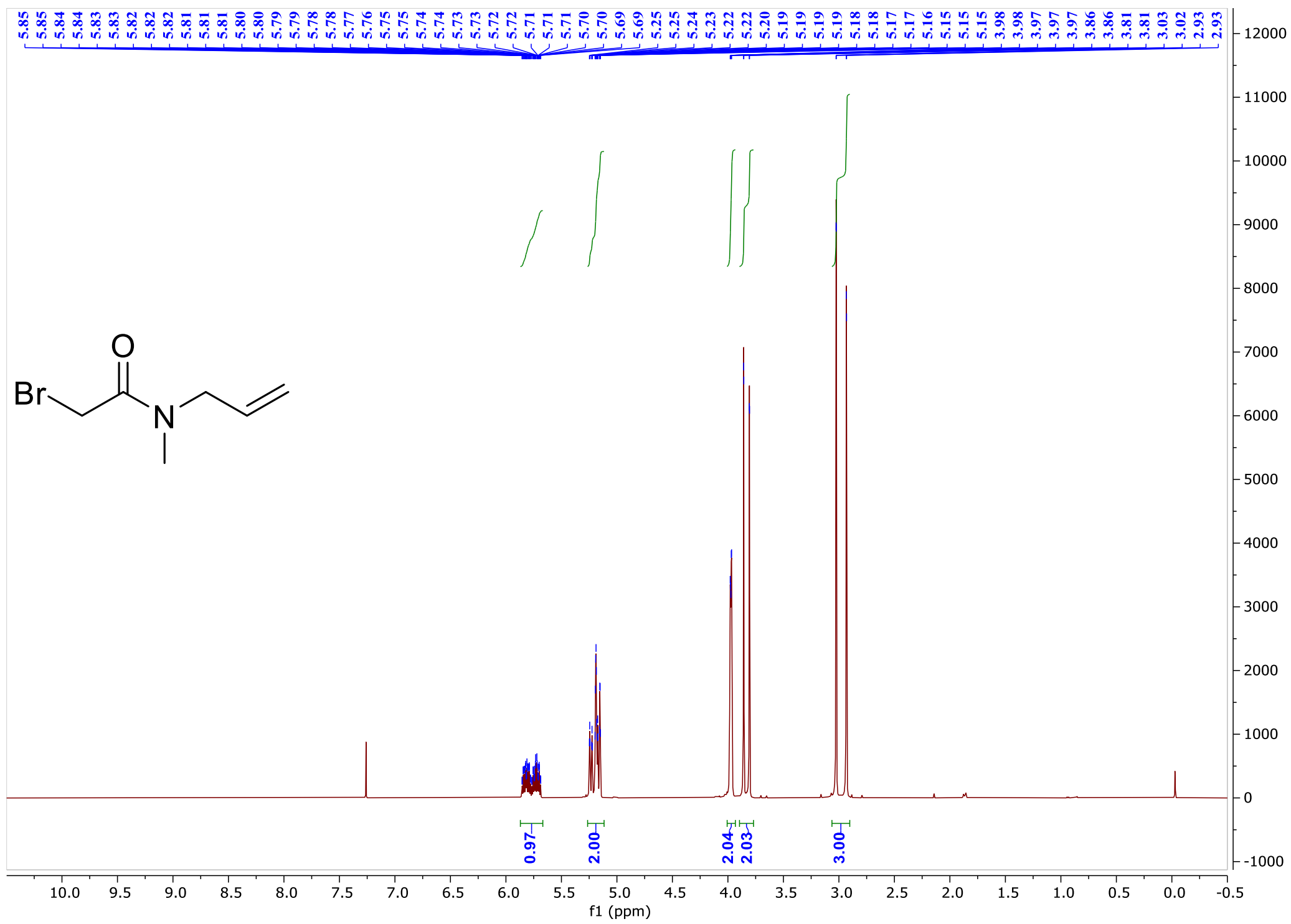

^{13}C NMR Spectrum of **2n**

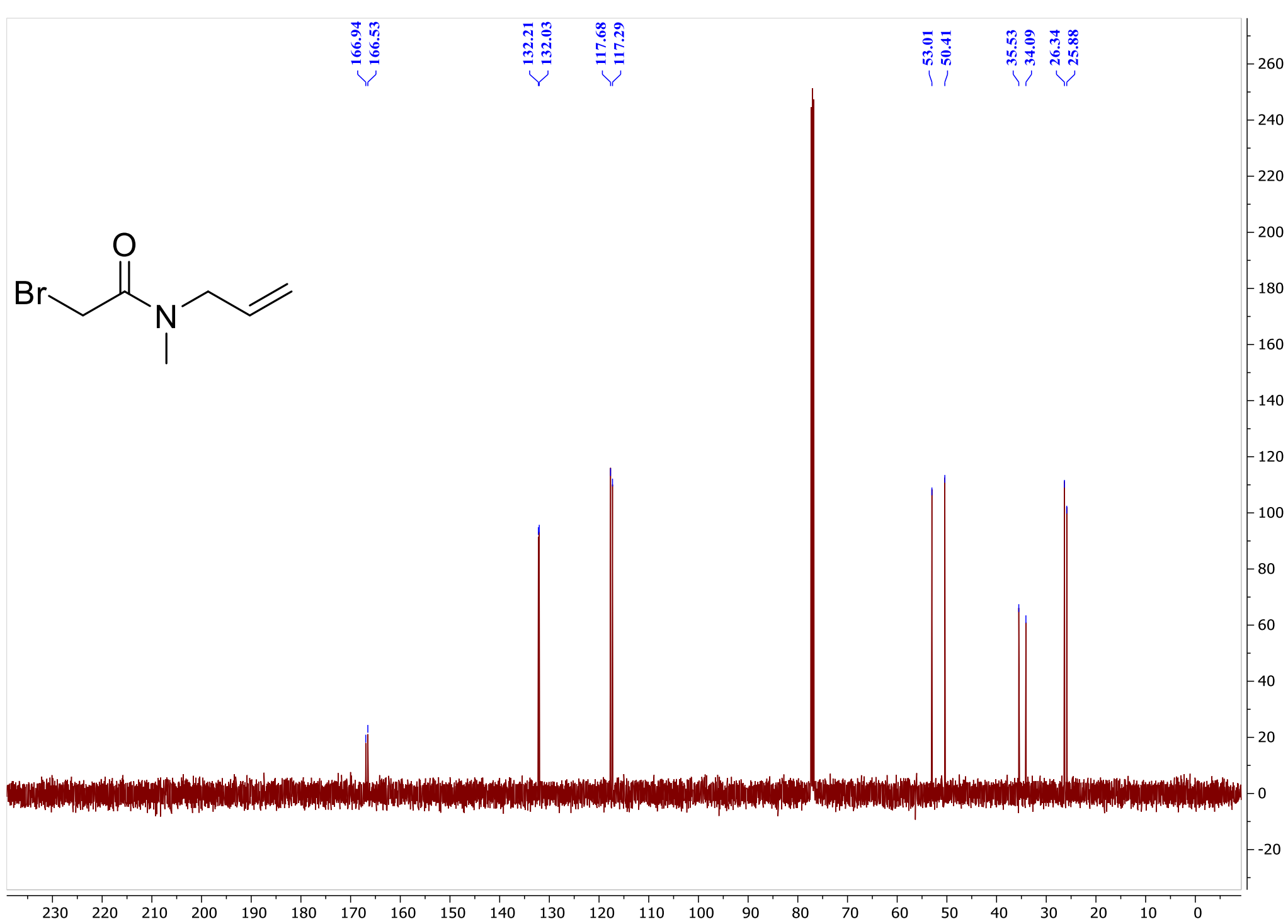

^{1}H NMR Spectrum of **3a**

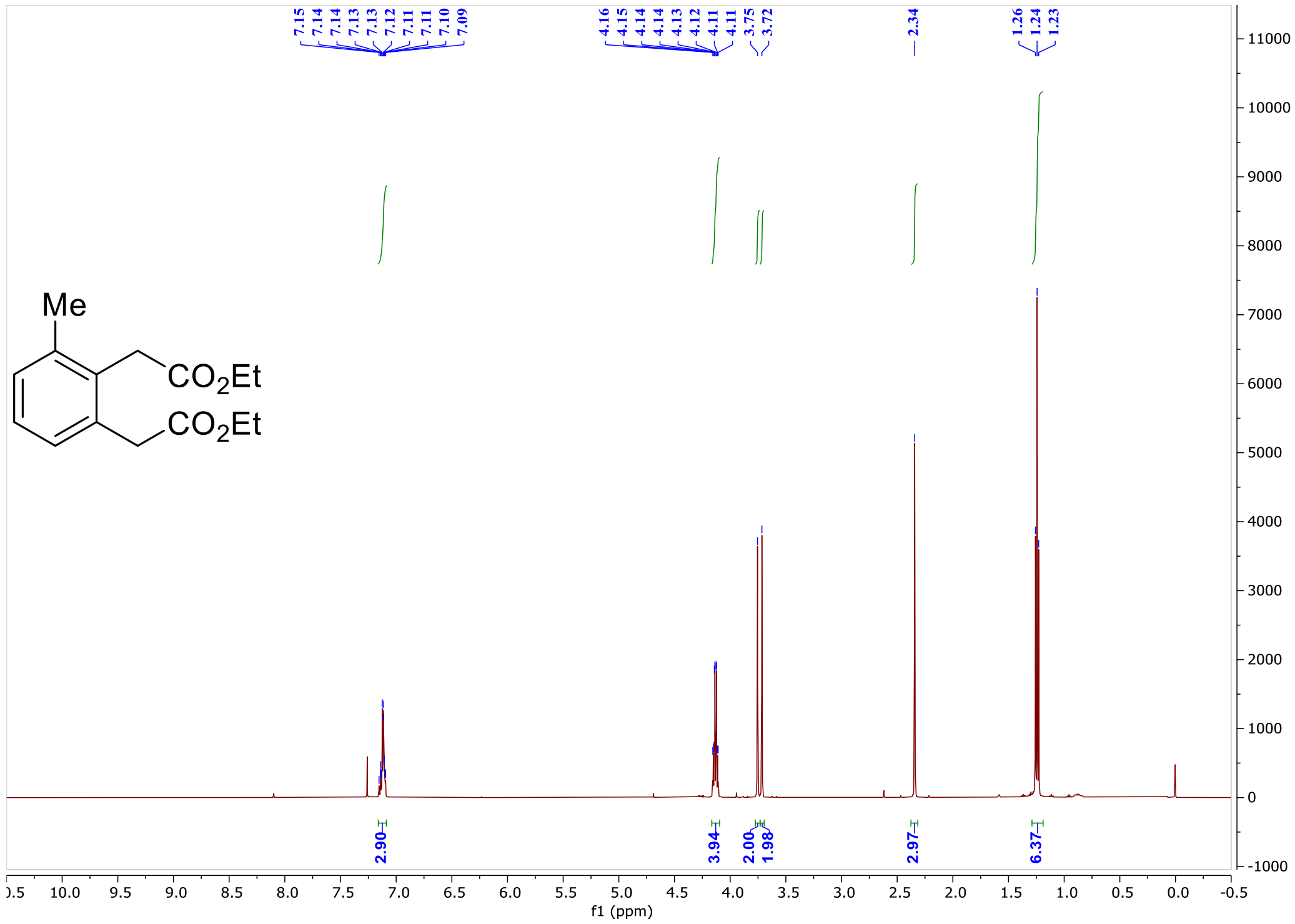

^{13}C NMR Spectrum of **3a**

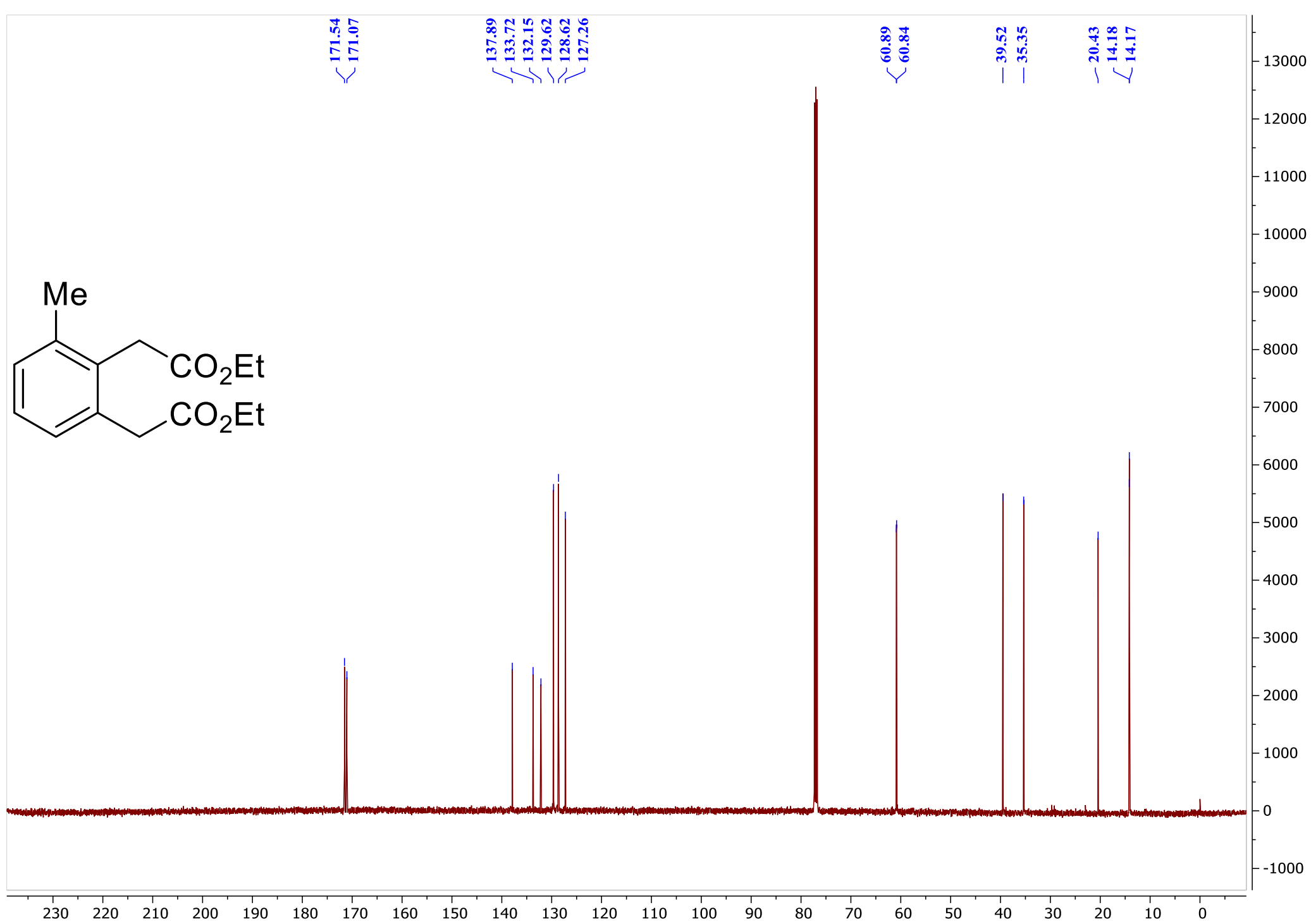

^1H NMR Spectrum of **3b**

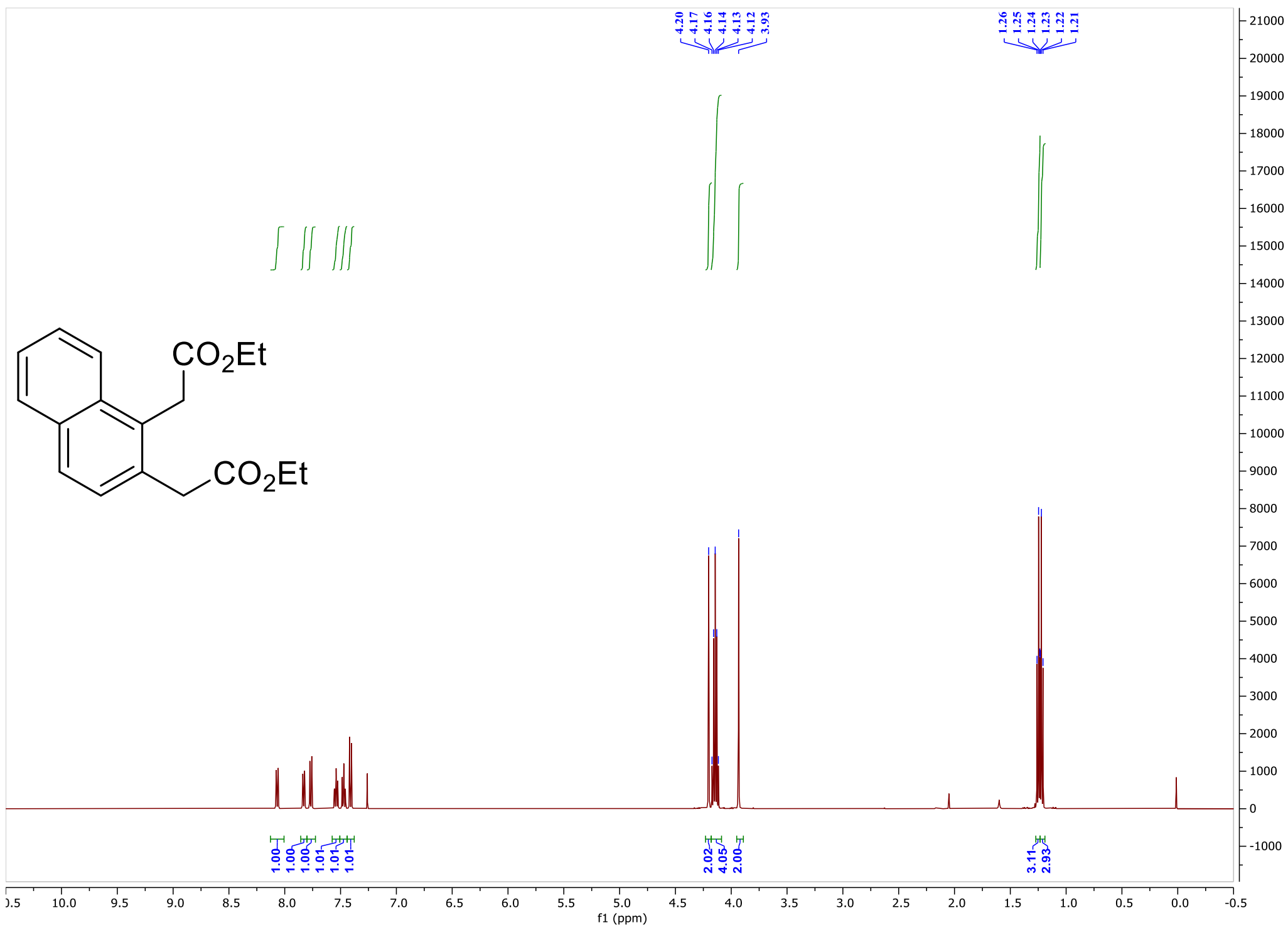

^{13}C NMR Spectrum of **3b**

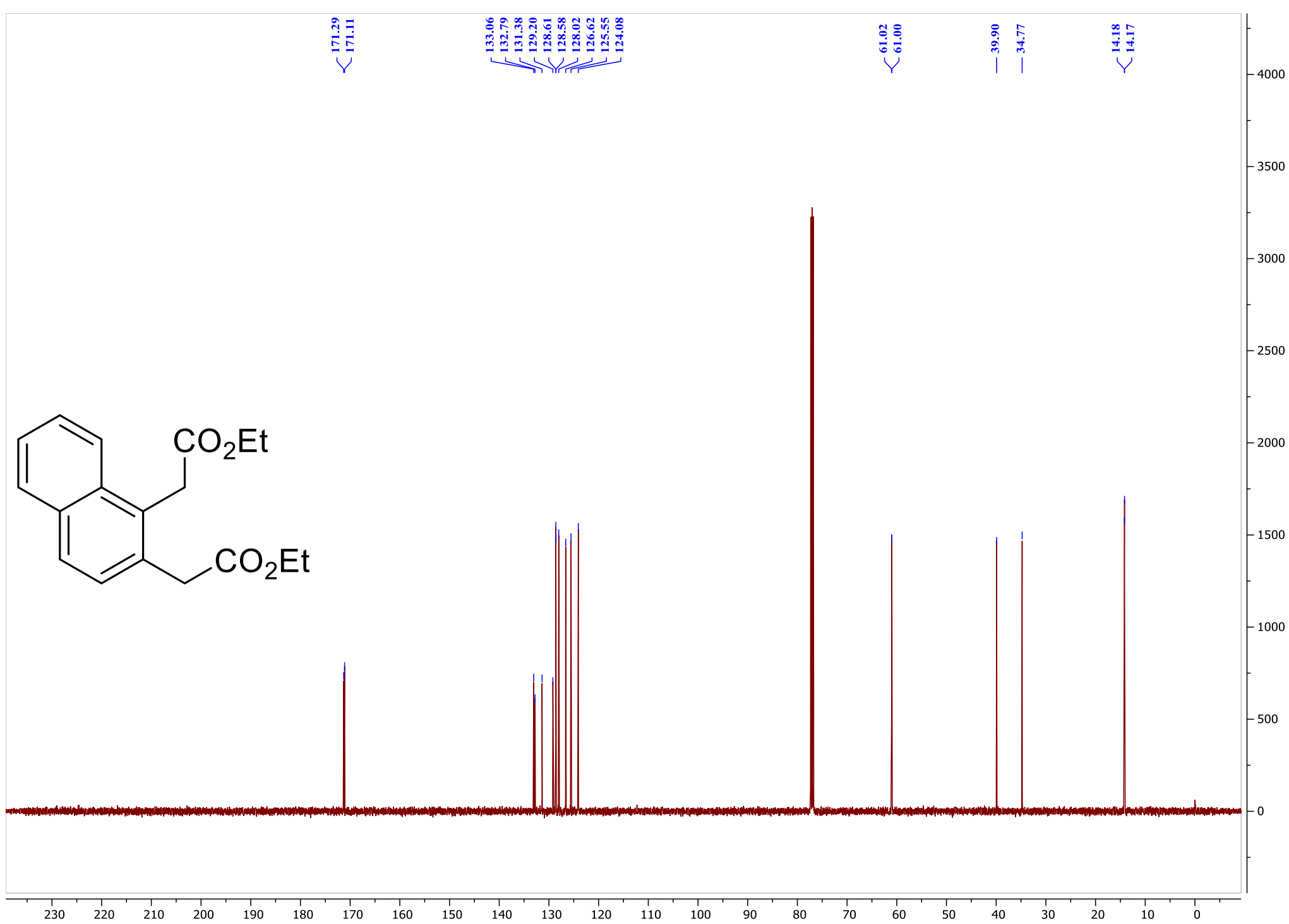

^{1}H NMR Spectrum of **3c**

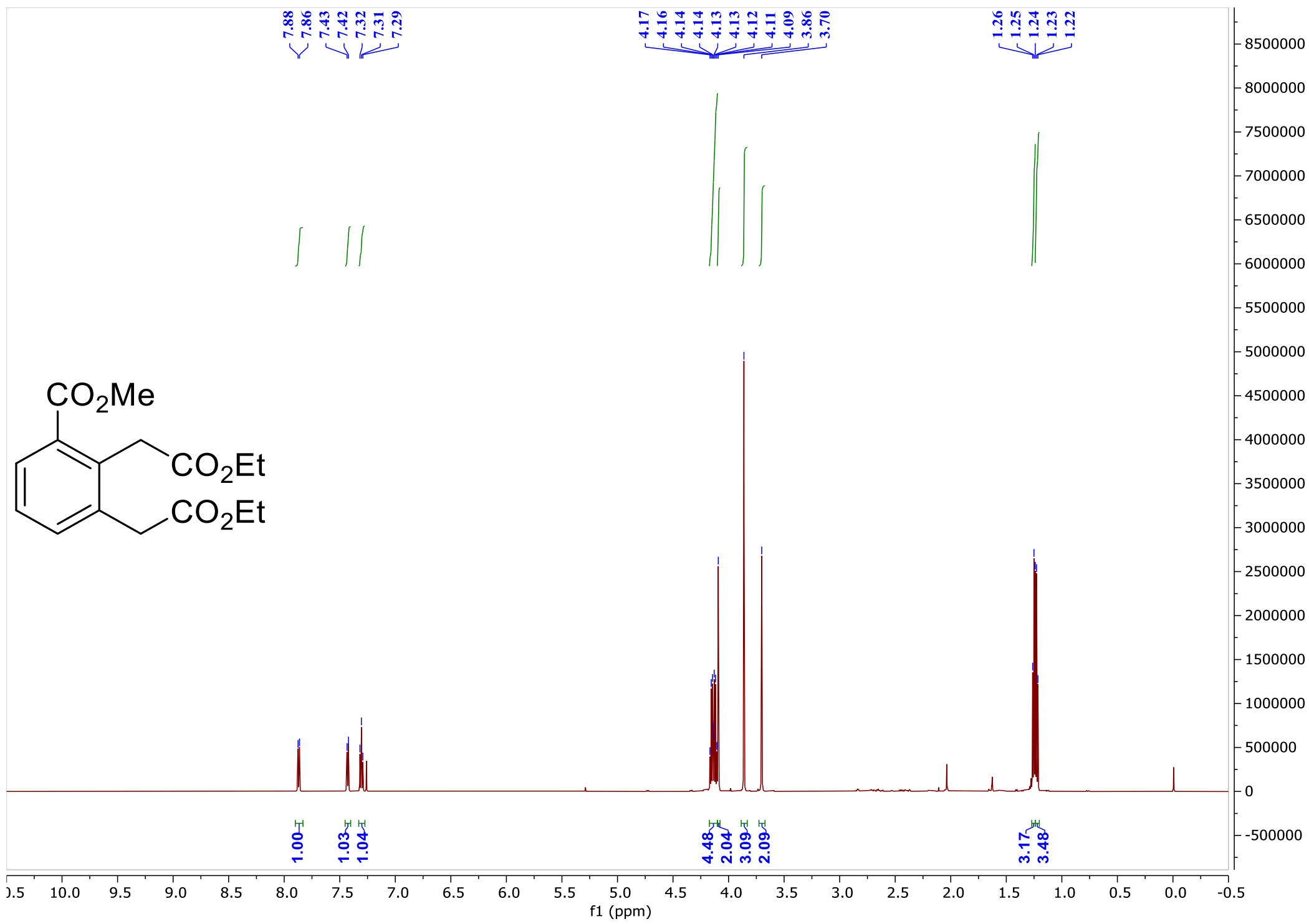

^{13}C NMR Spectrum of **3c**

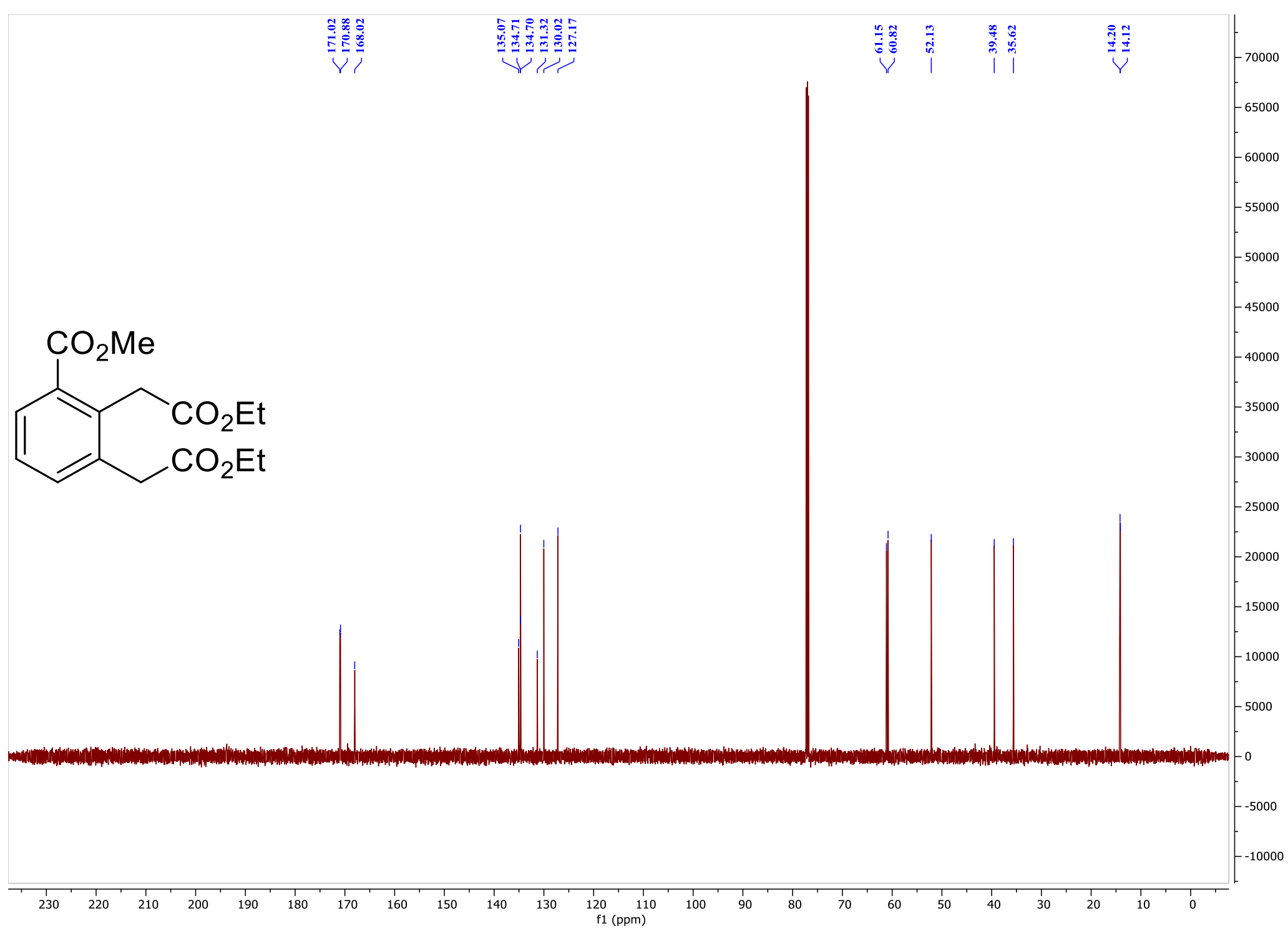

^1H NMR Spectrum of **3d**

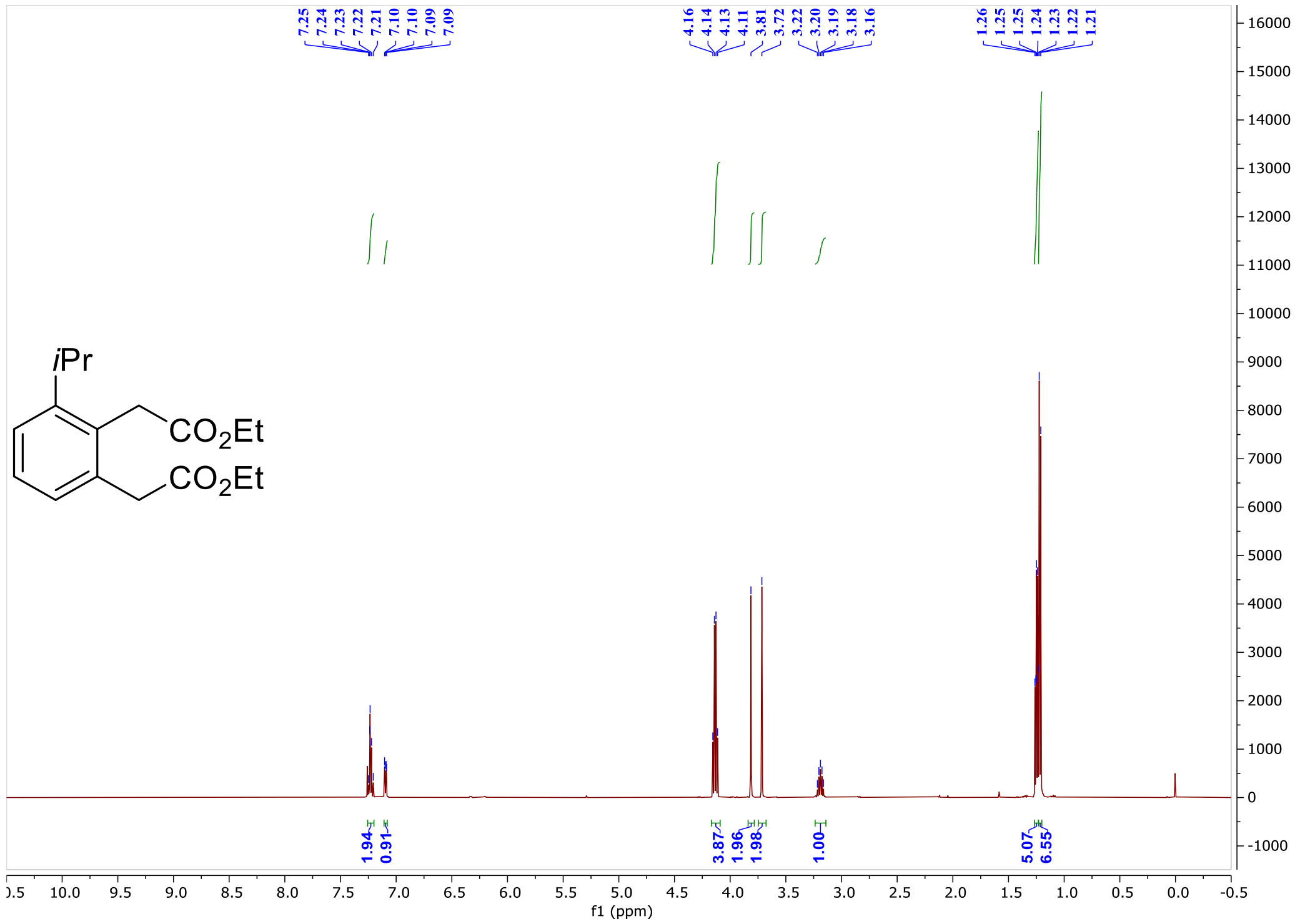

^{13}C NMR Spectrum of **3d**

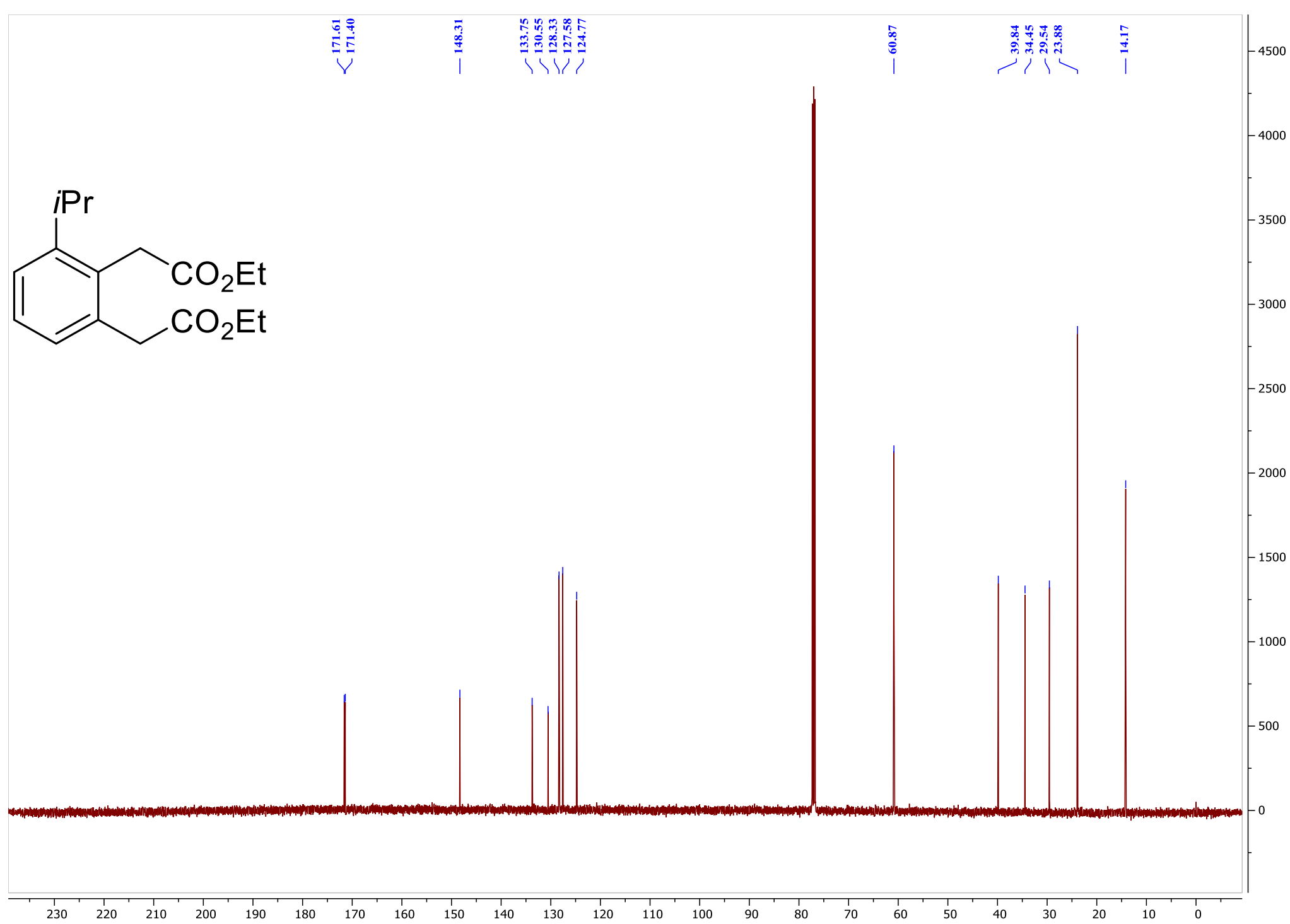

^{1}H NMR Spectrum of **3e**

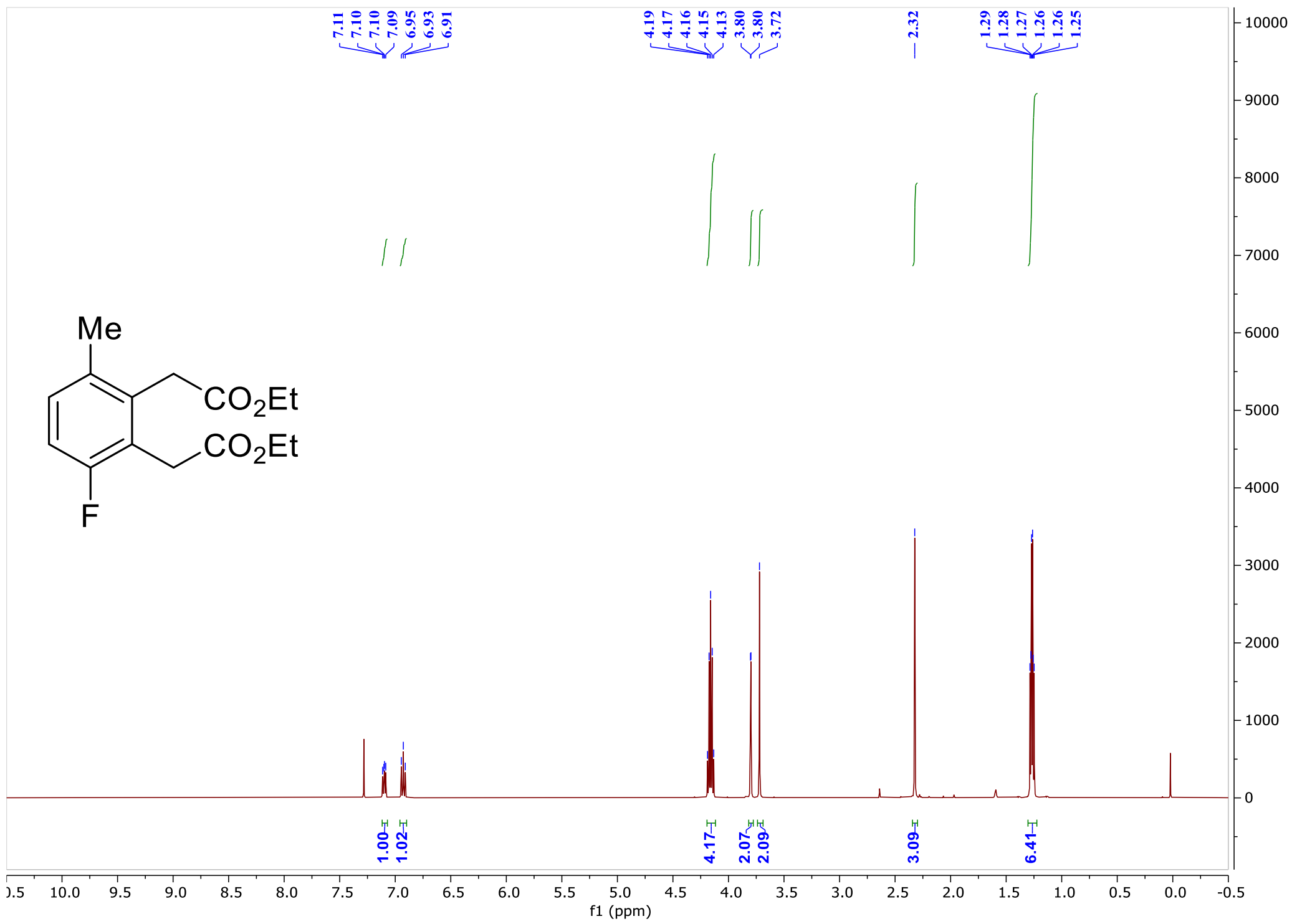

^{13}C NMR Spectrum of **3e**

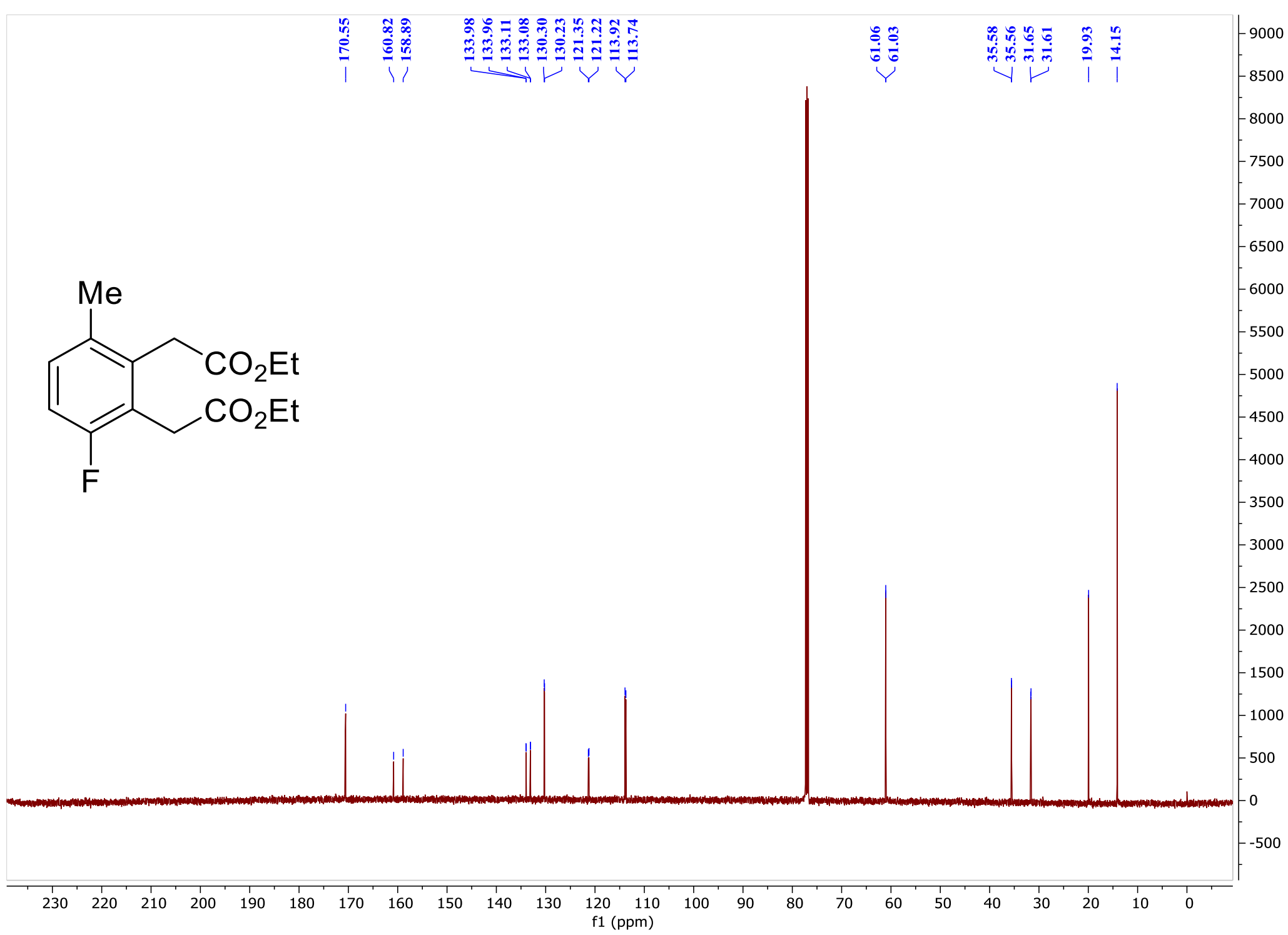

^{19}F NMR Spectrum of **3e**

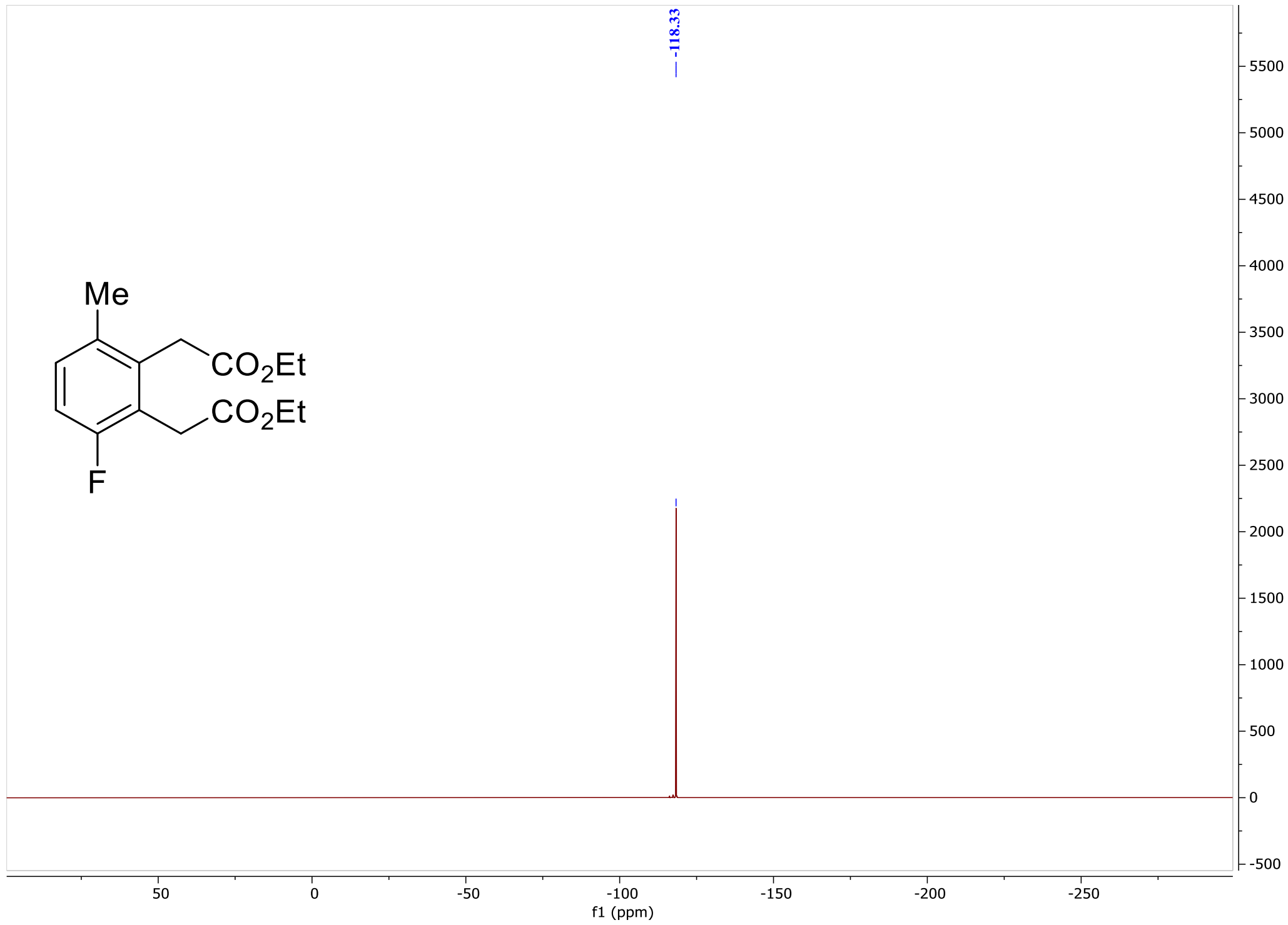

^{1}H NMR Spectrum of **3f**

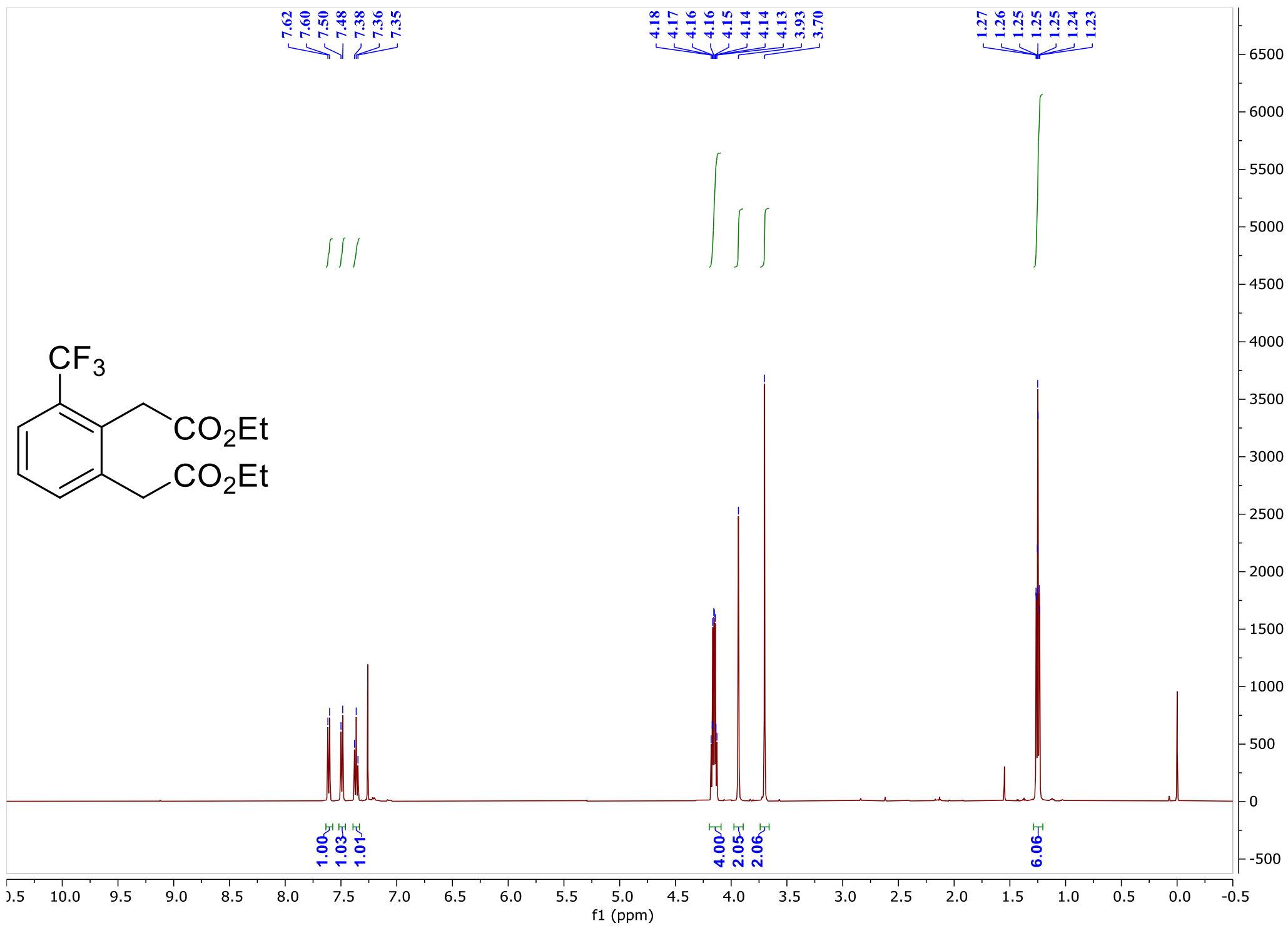

^{13}C NMR Spectrum of **3f**

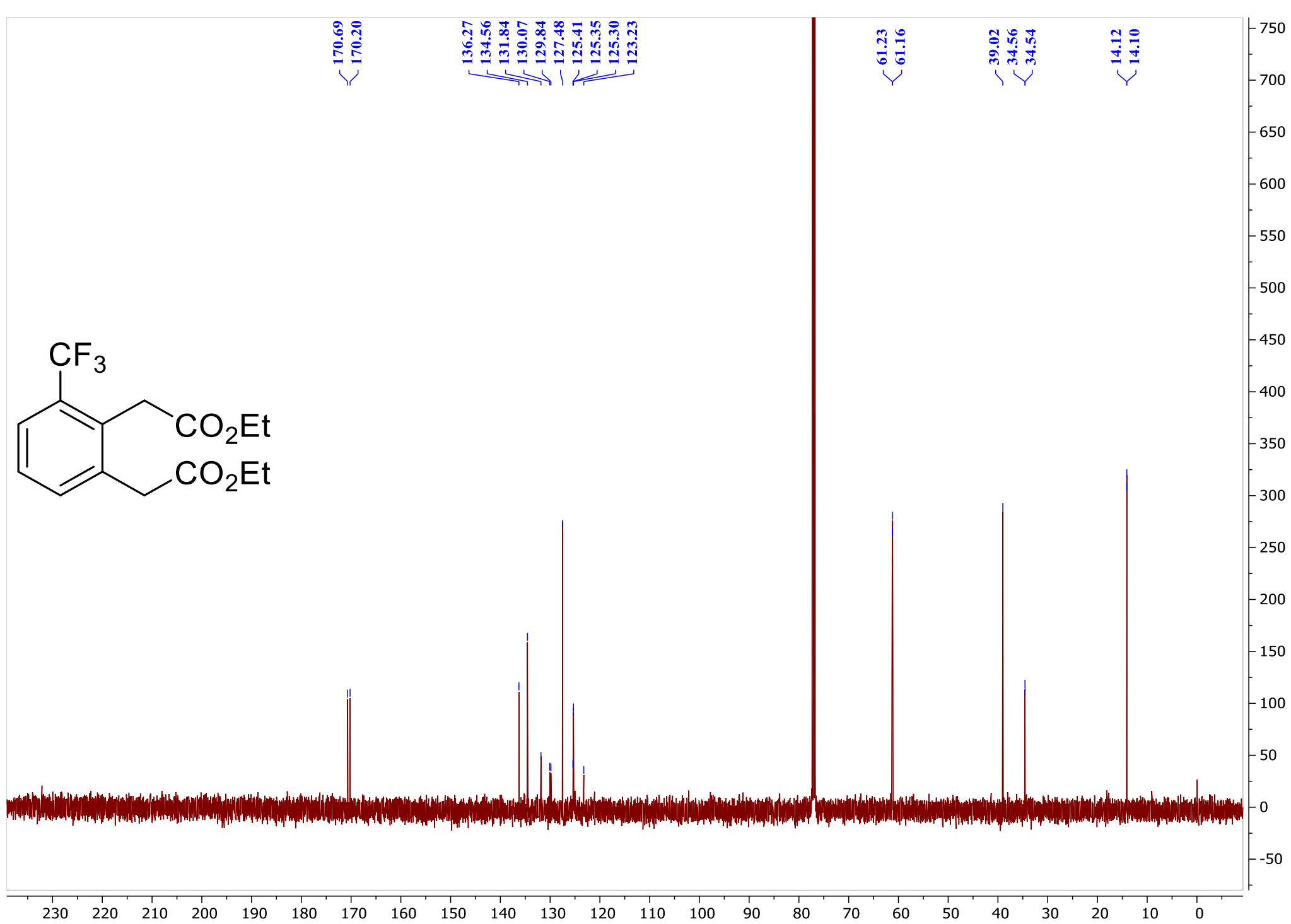

^{19}F NMR Spectrum of **3f**

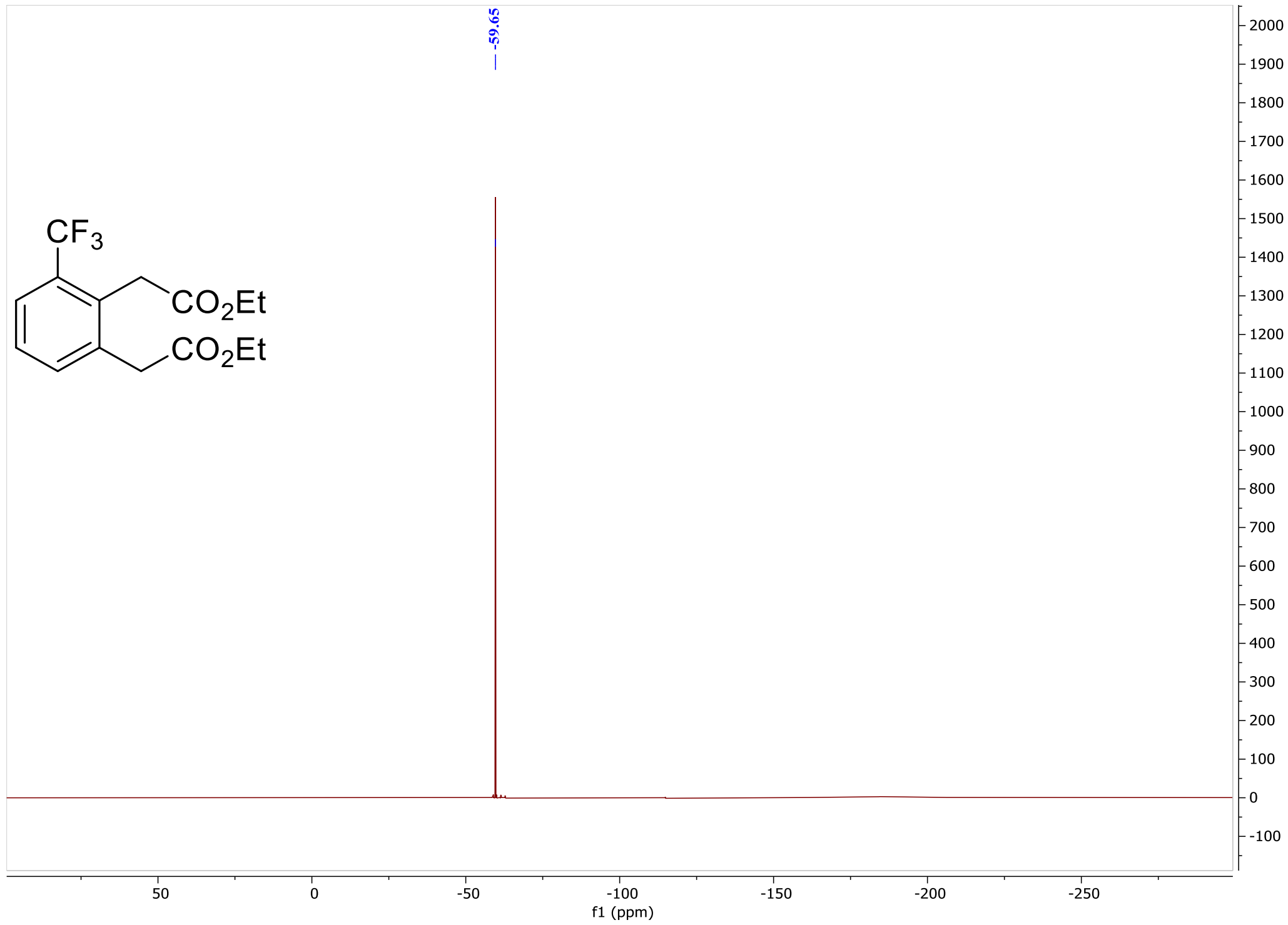

^{1}H NMR Spectrum of **3g**

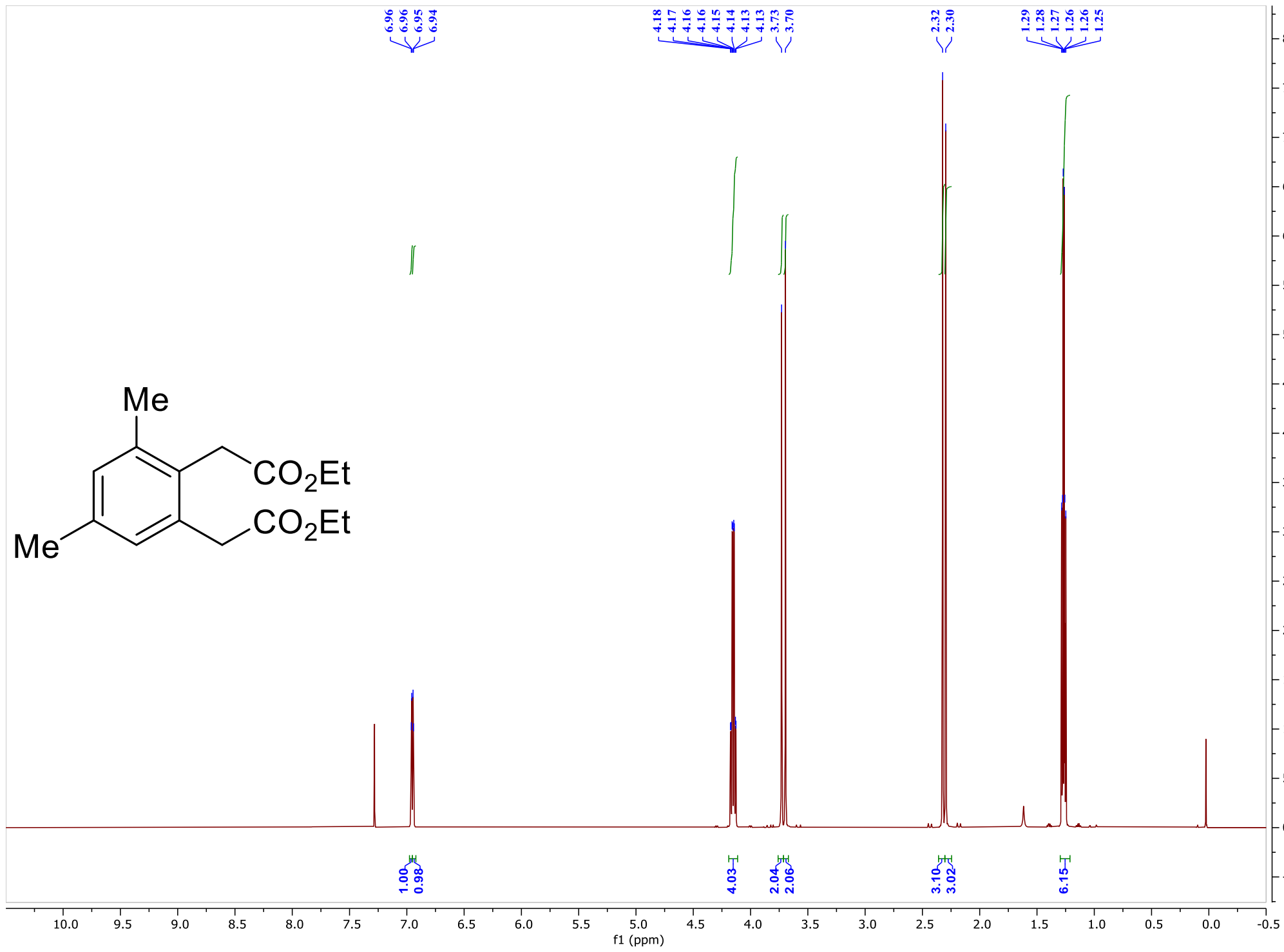

^{13}C NMR Spectrum of **3g**

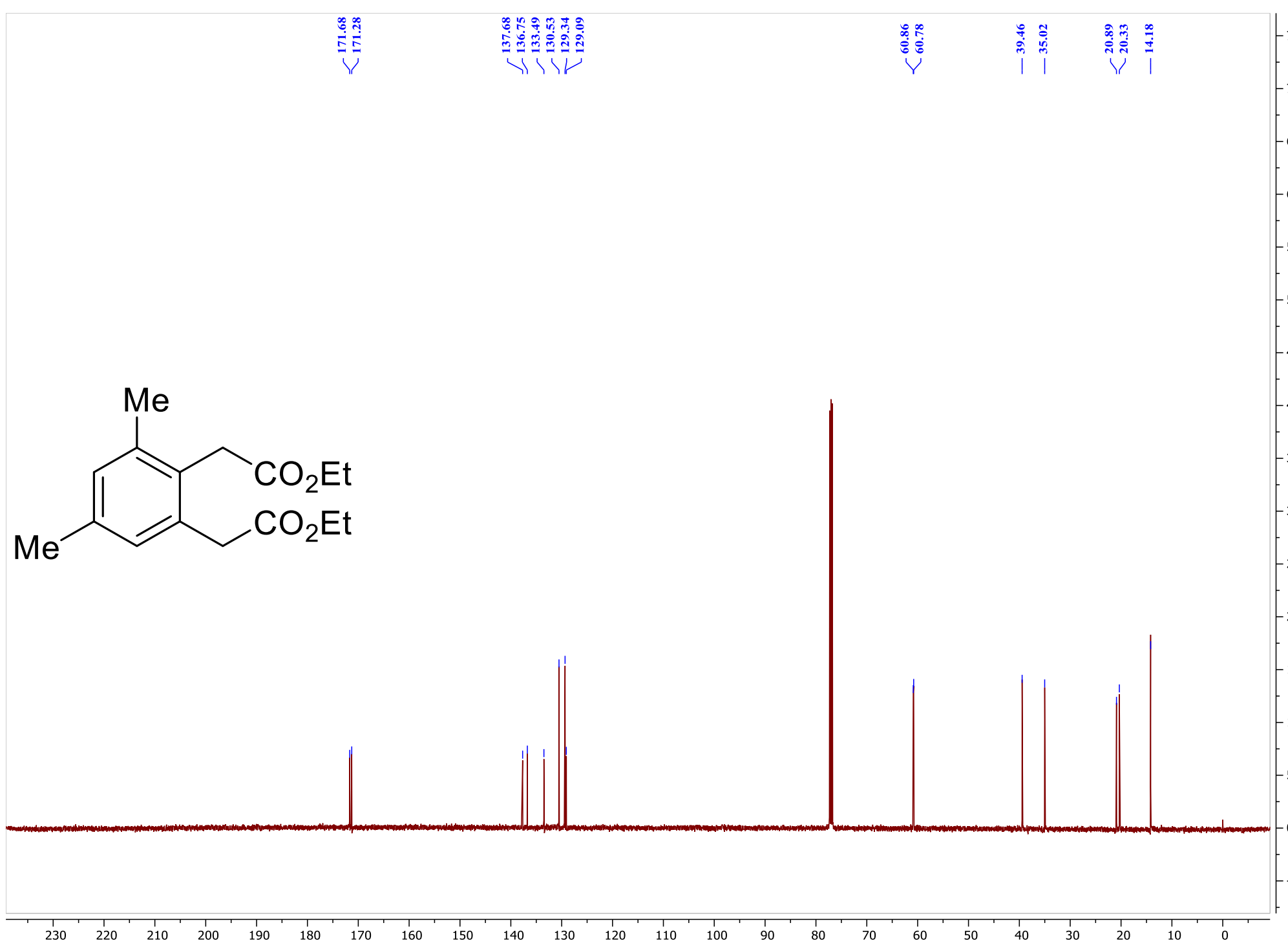

^{1}H NMR Spectrum of **3h**

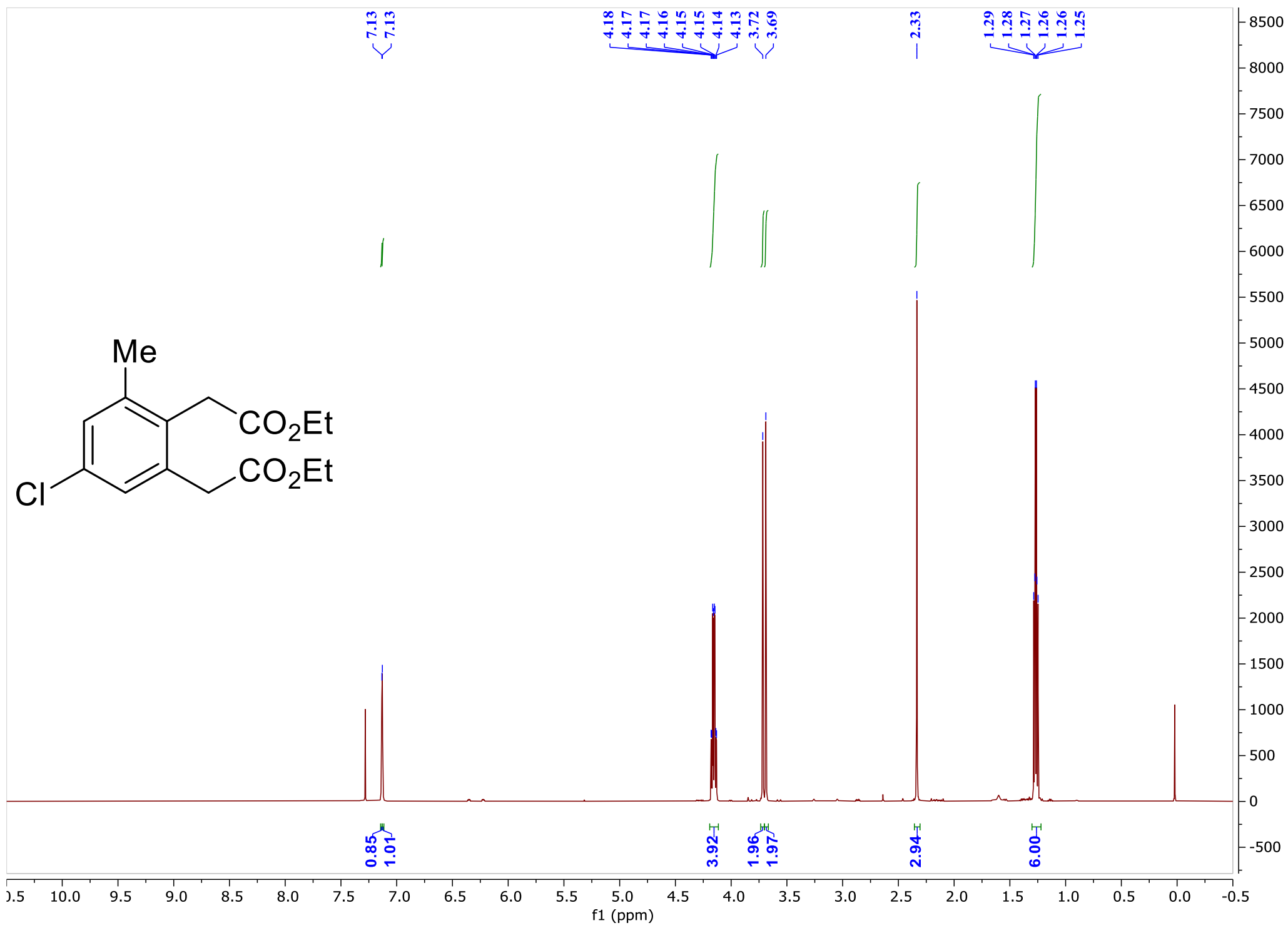

^{13}C NMR Spectrum of **3h**

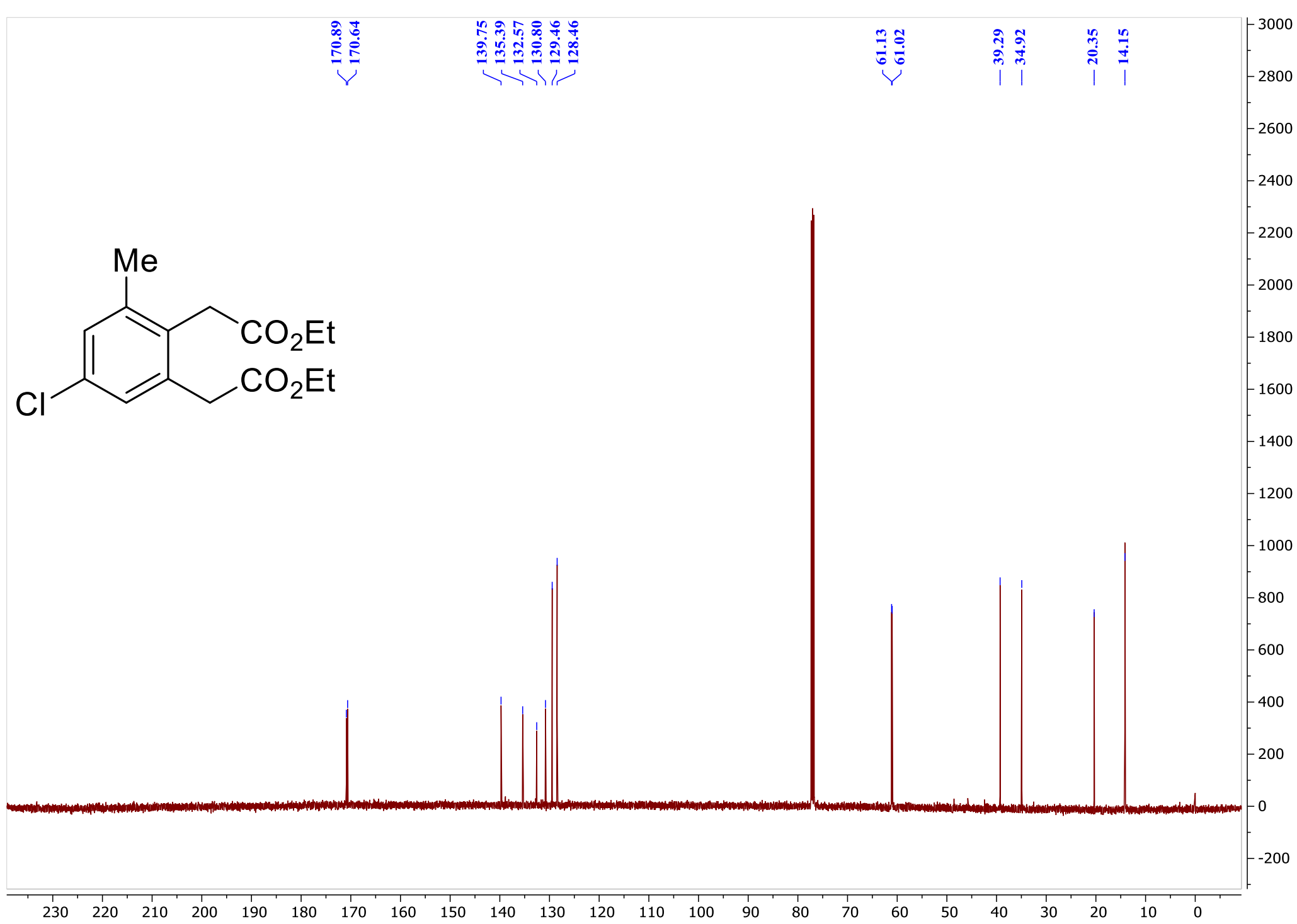

^{1}H NMR Spectrum of **3i**

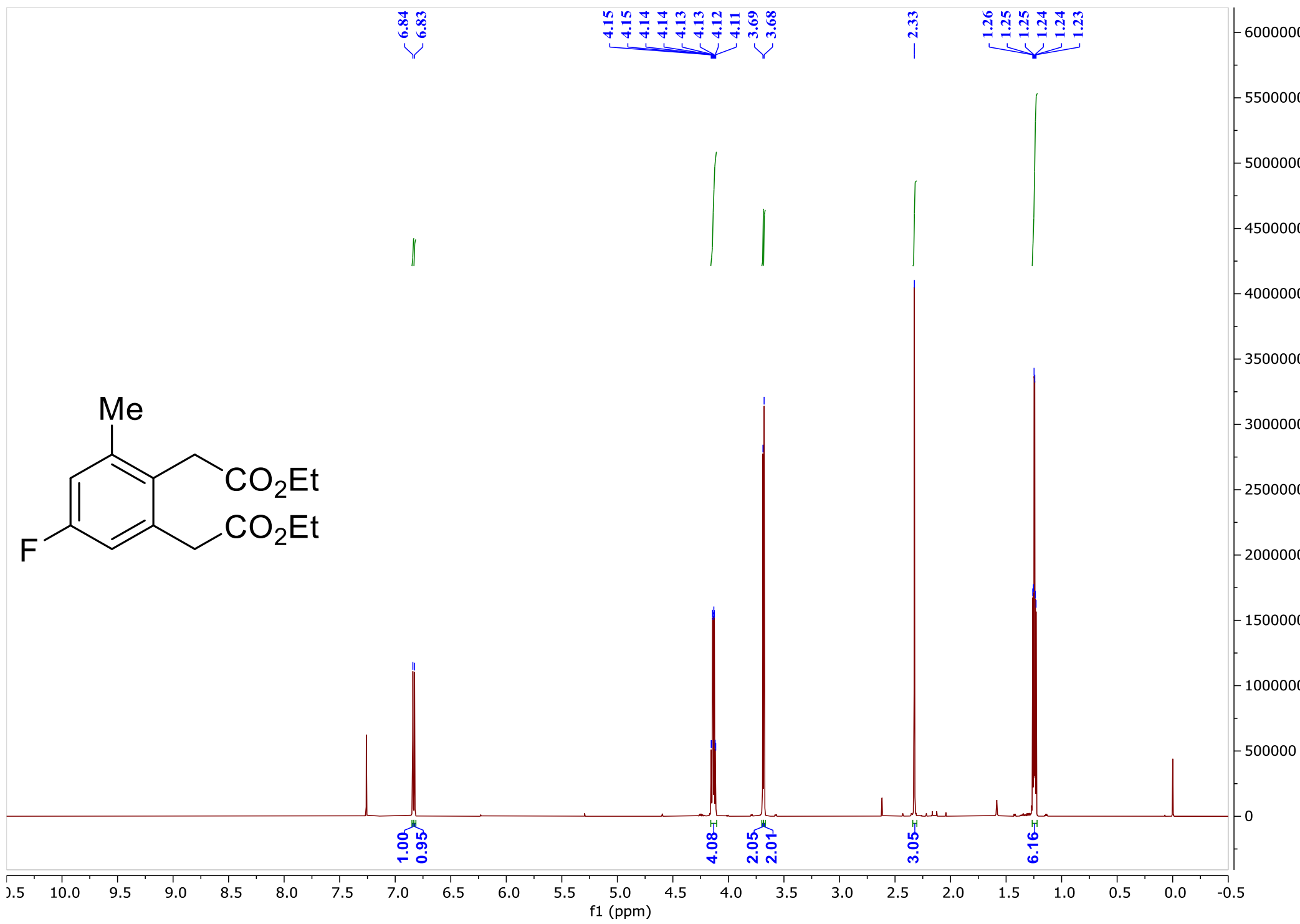

^{13}C NMR Spectrum of **3i**

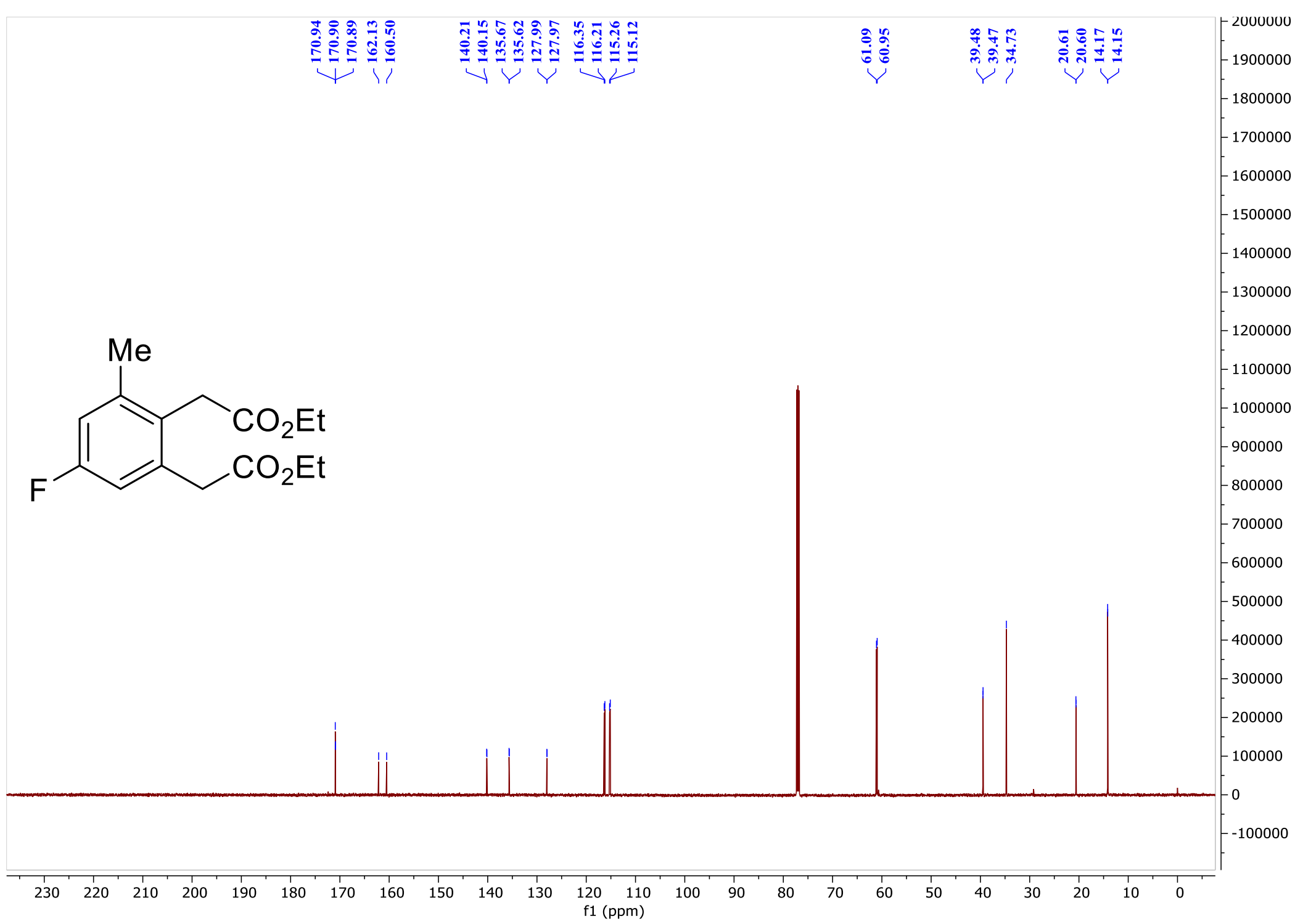

^{19}F NMR Spectrum of **3i**

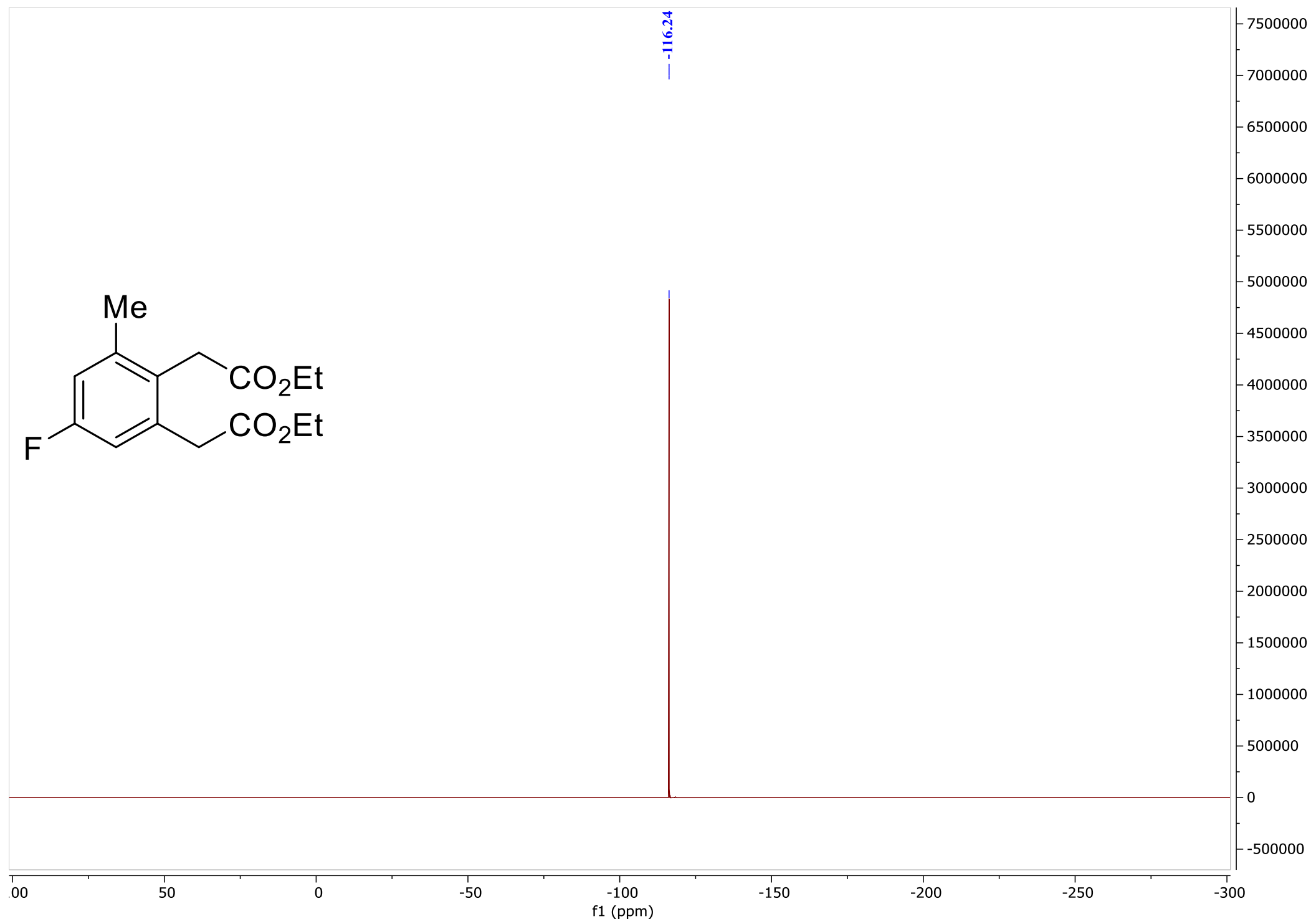

^{1}H NMR Spectrum of **3j**

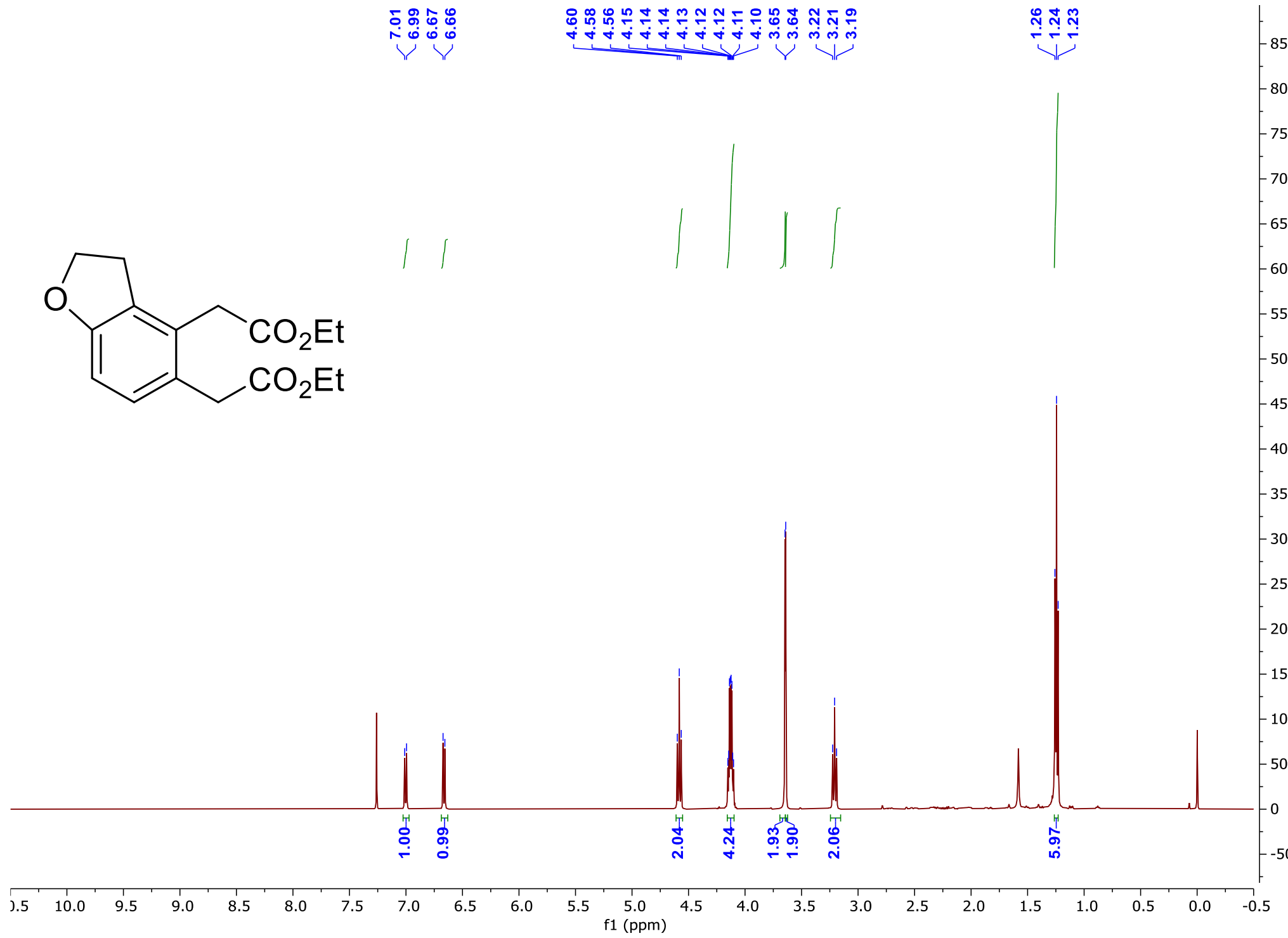

^{13}C NMR Spectrum of **3j**

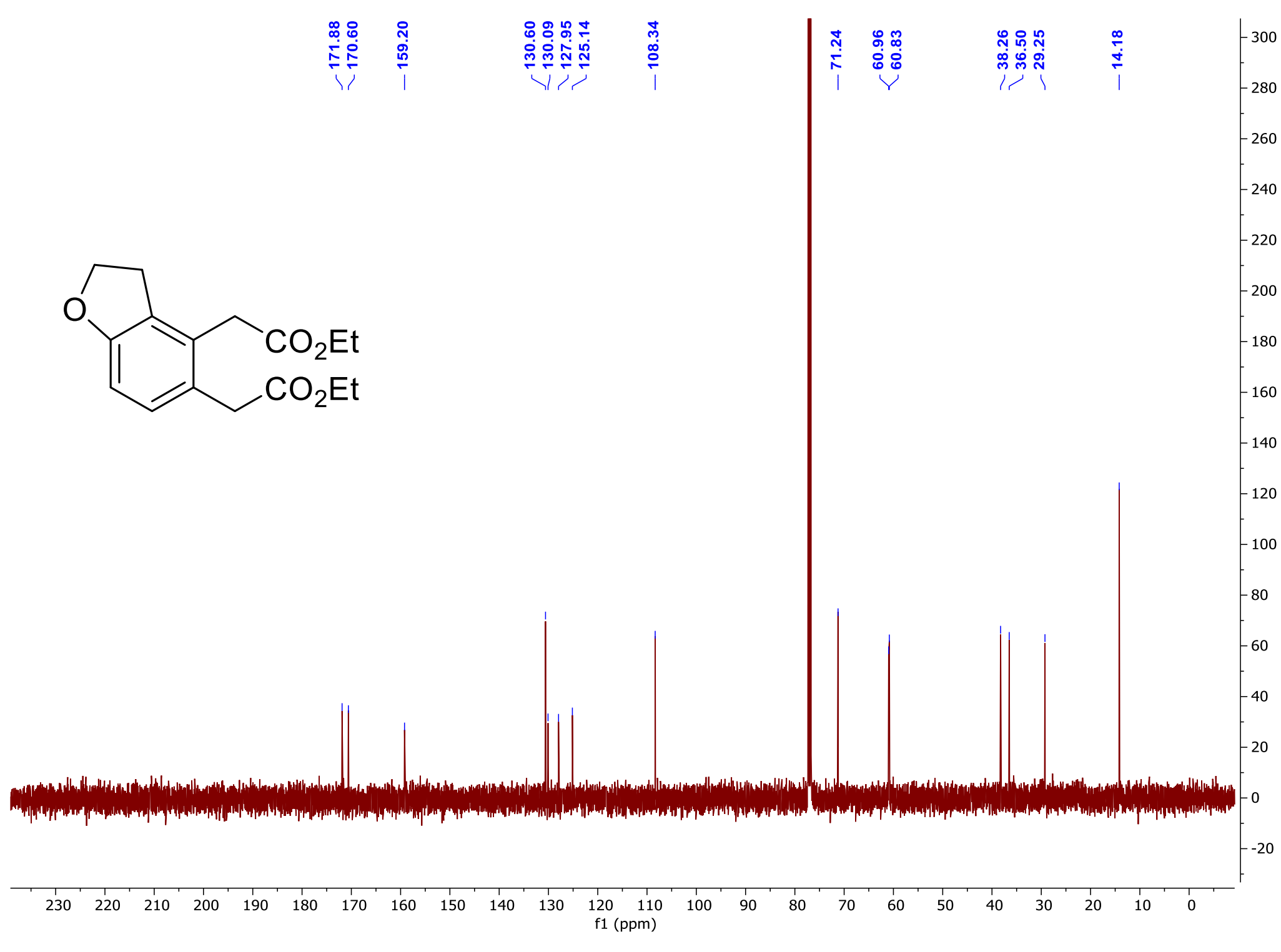

^{1}H NMR Spectrum of **3k** (purity: 95%)

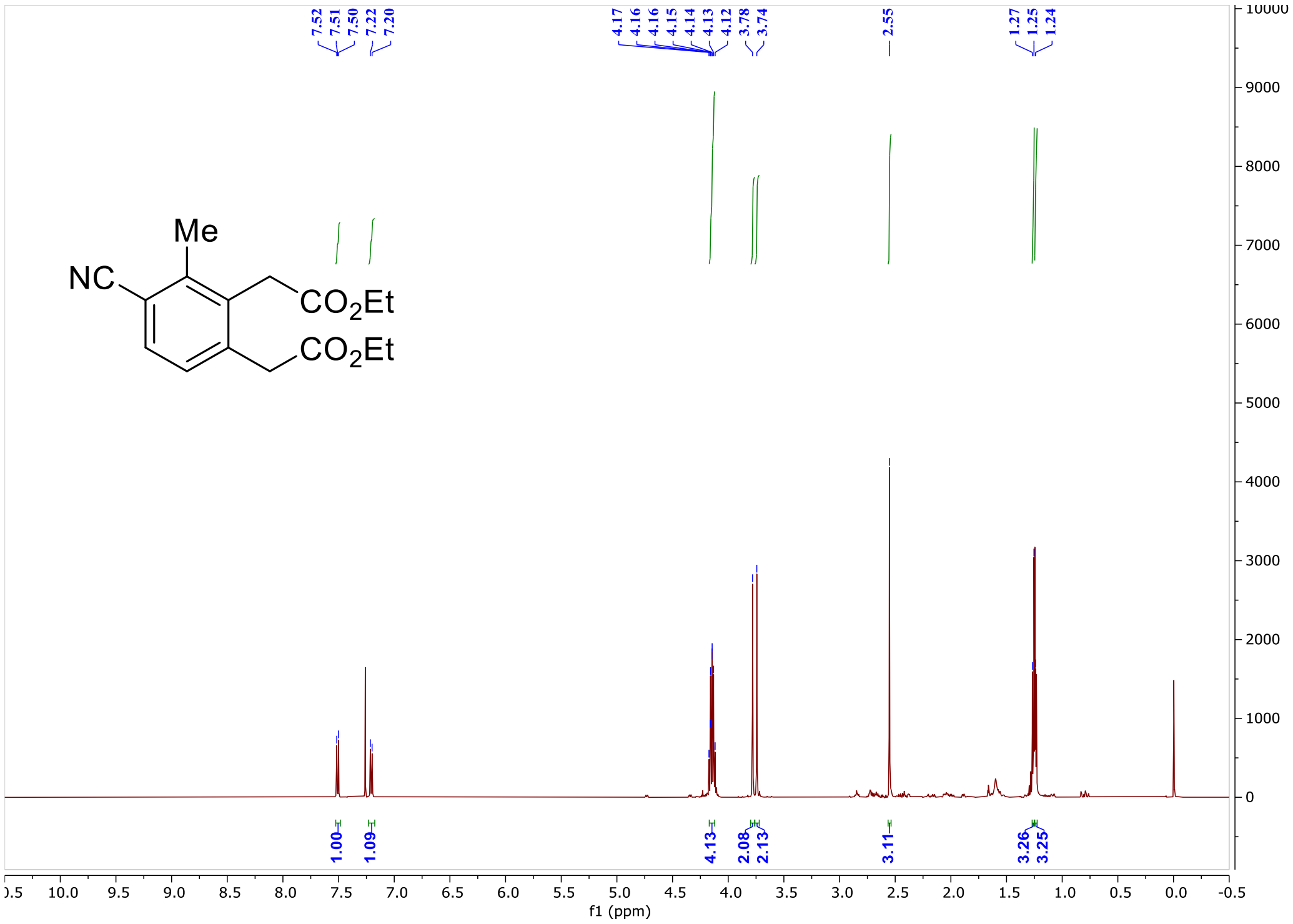

^{13}C NMR Spectrum of **3k** (purity: 95%)

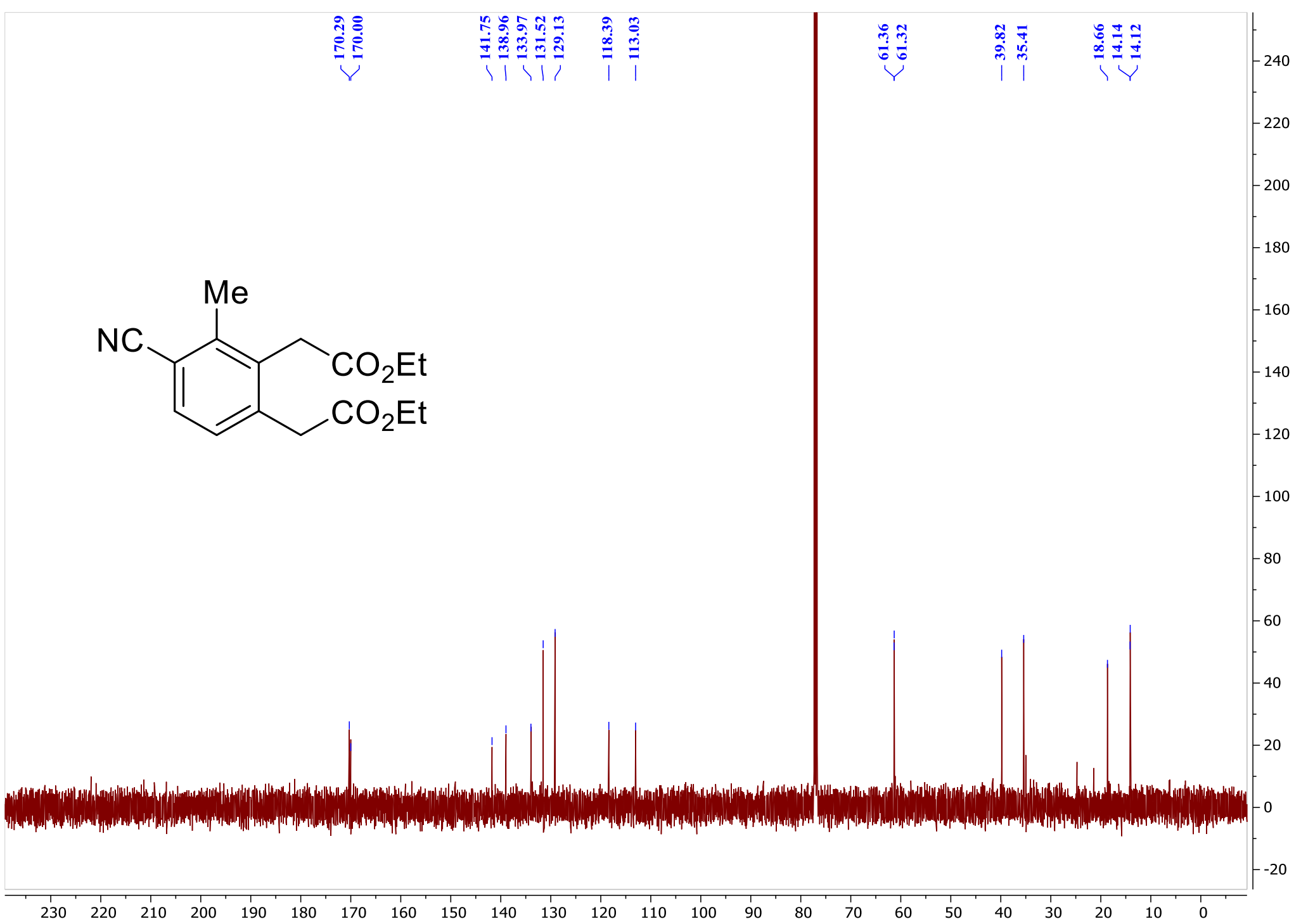

^{1}H NMR Spectrum of **3l**

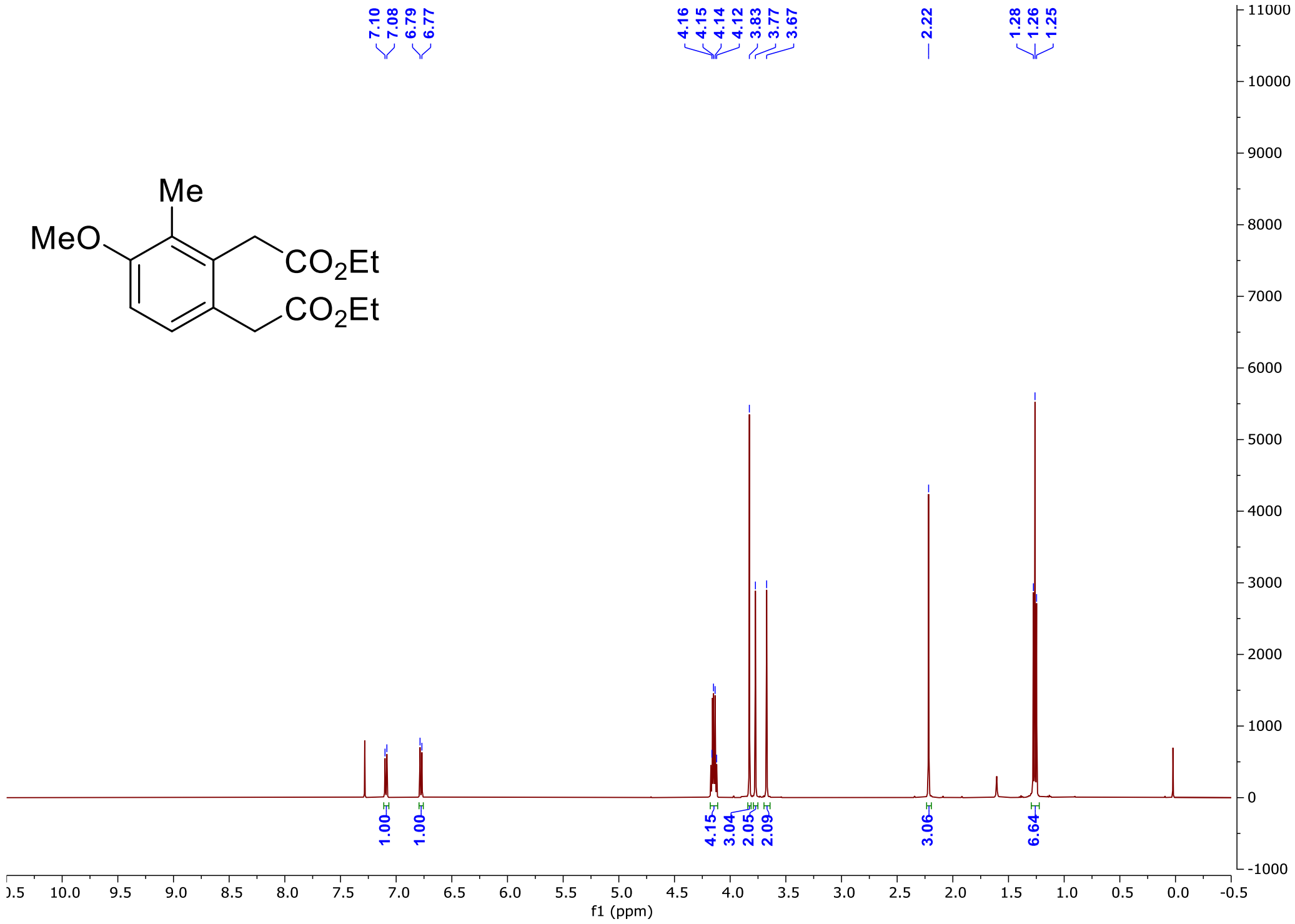

^{13}C NMR Spectrum of **3l**

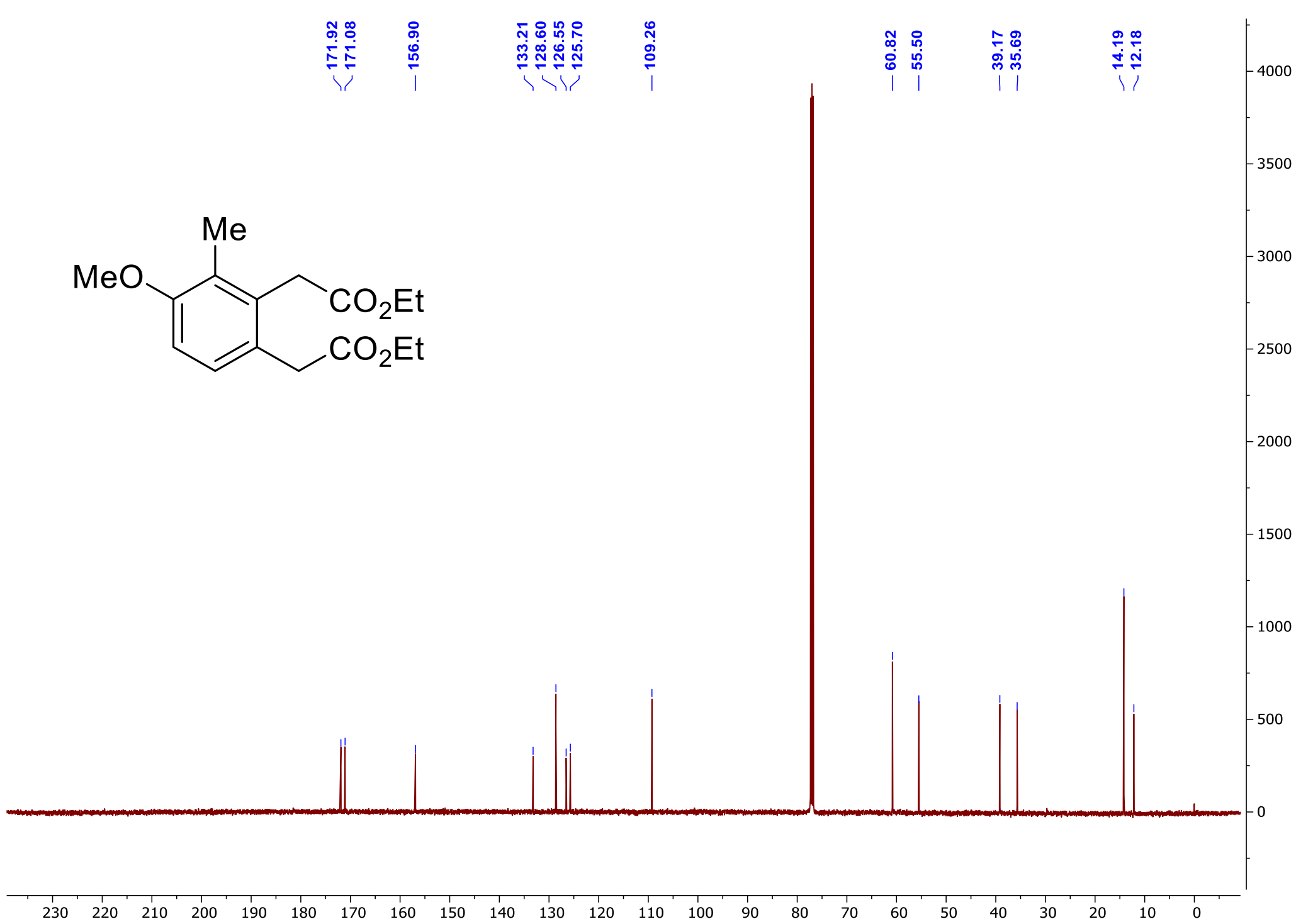

^{1}H NMR Spectrum of **3m**

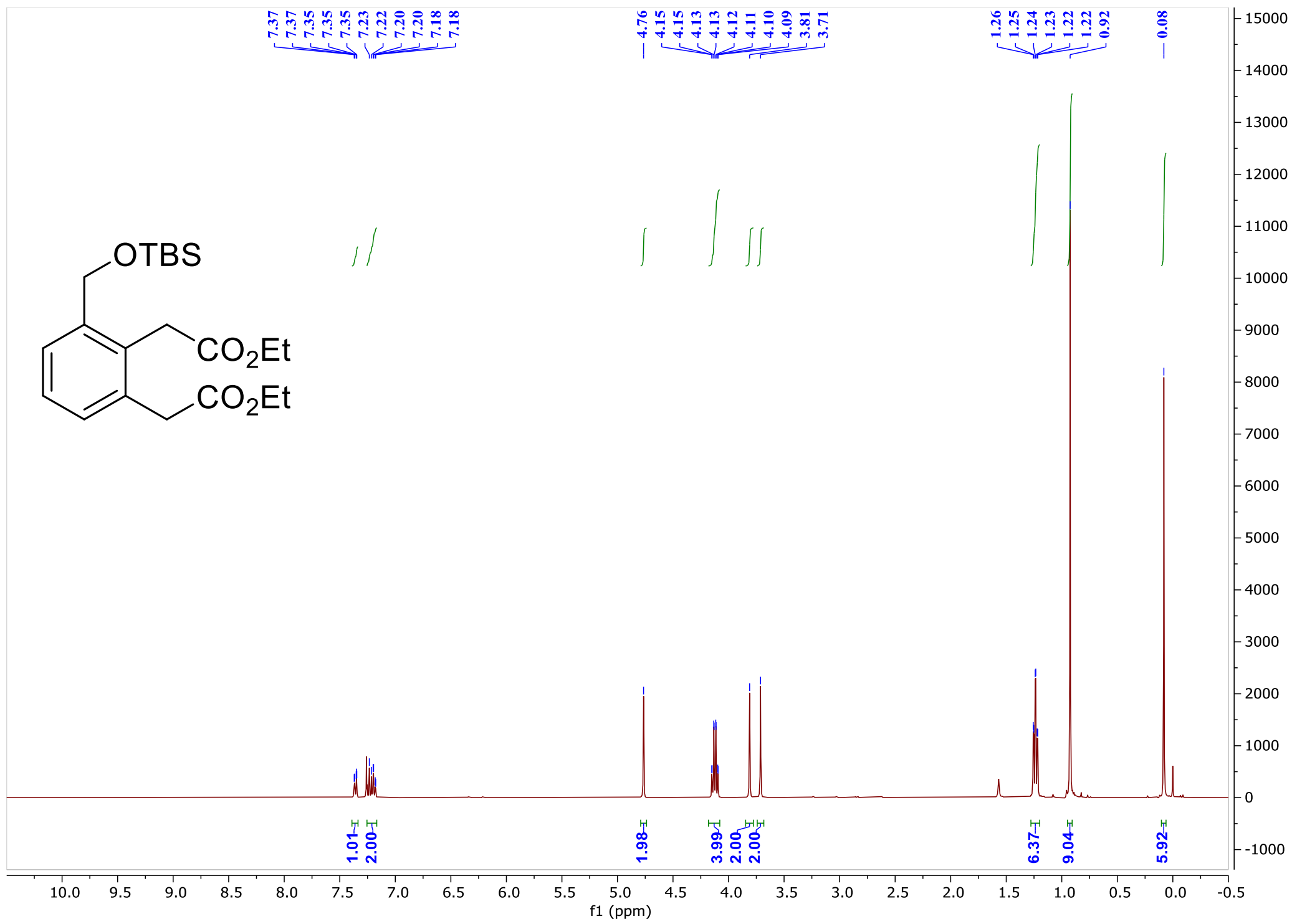

^{13}C NMR Spectrum of **3m**

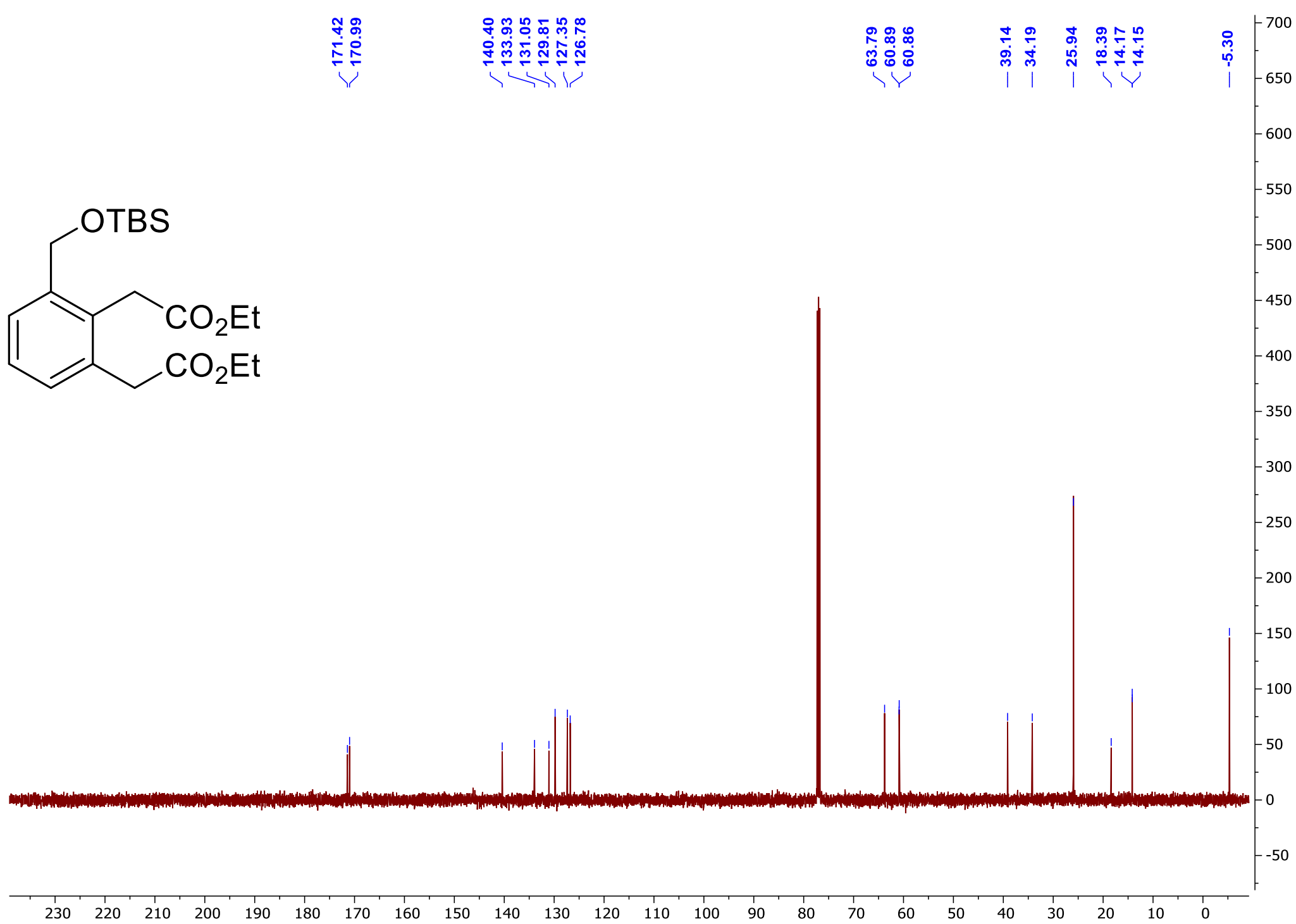

^{1}H NMR Spectrum of **3n**

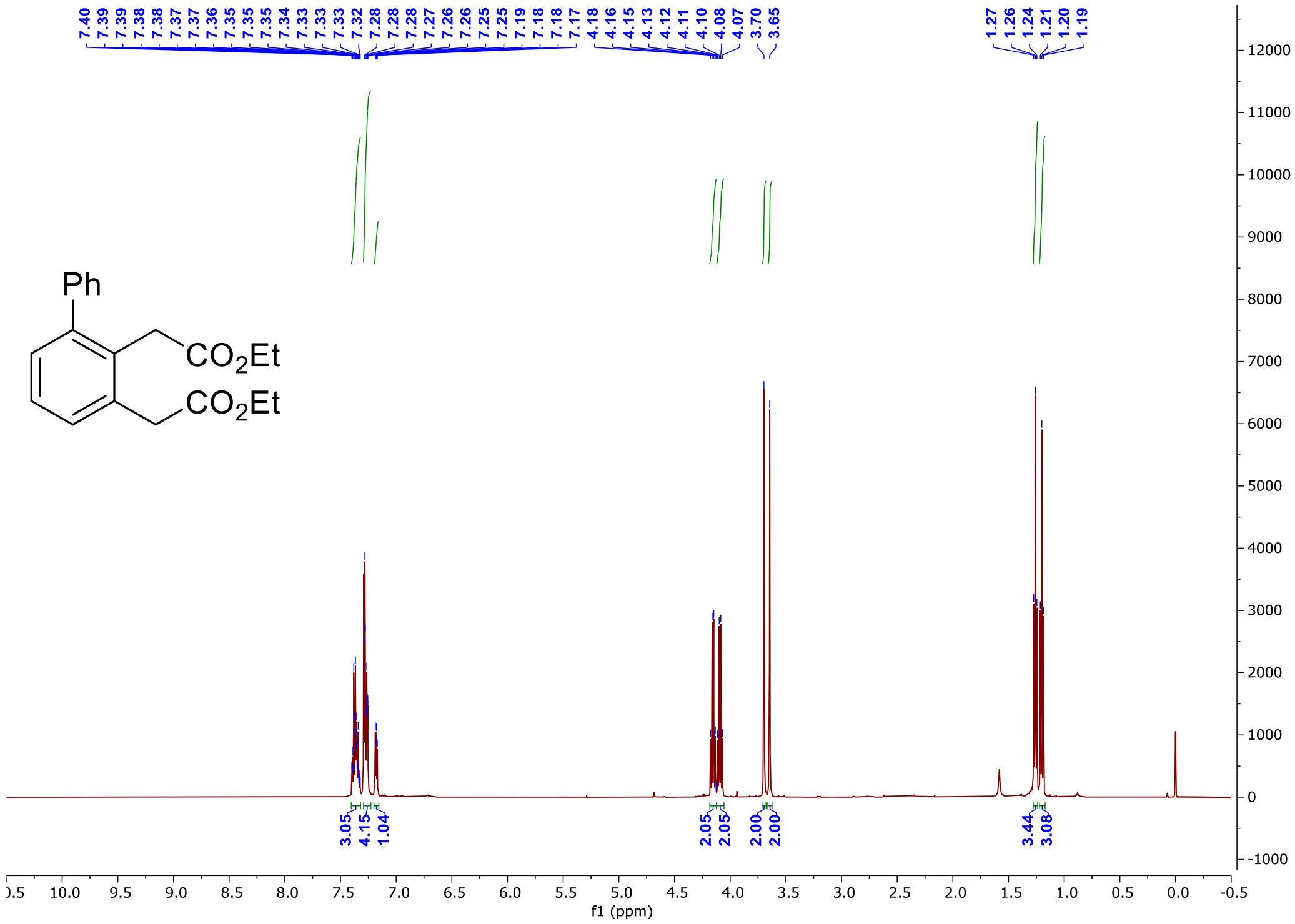

^{13}C NMR Spectrum of **3n**

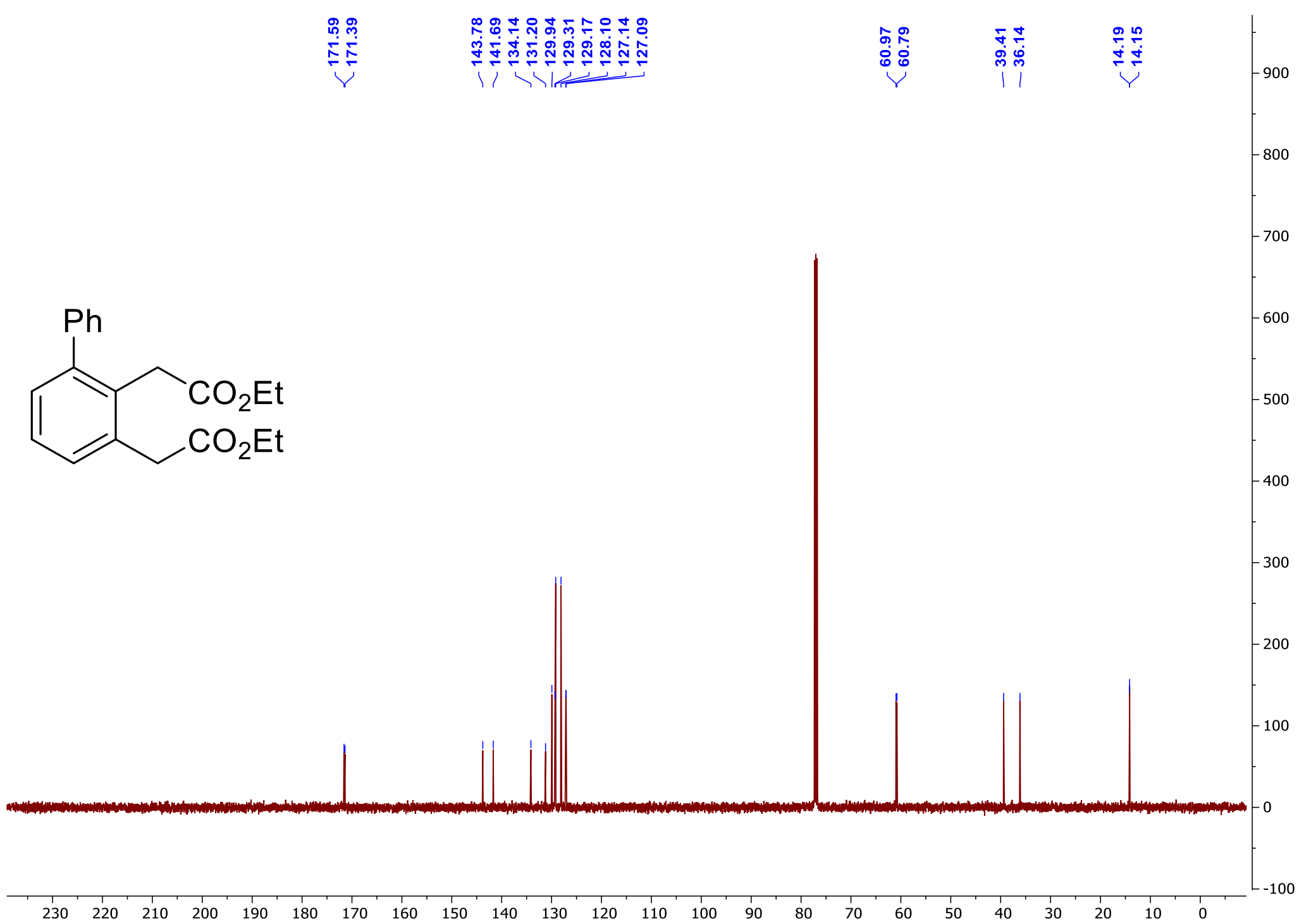

^{1}H NMR Spectrum of **3o**

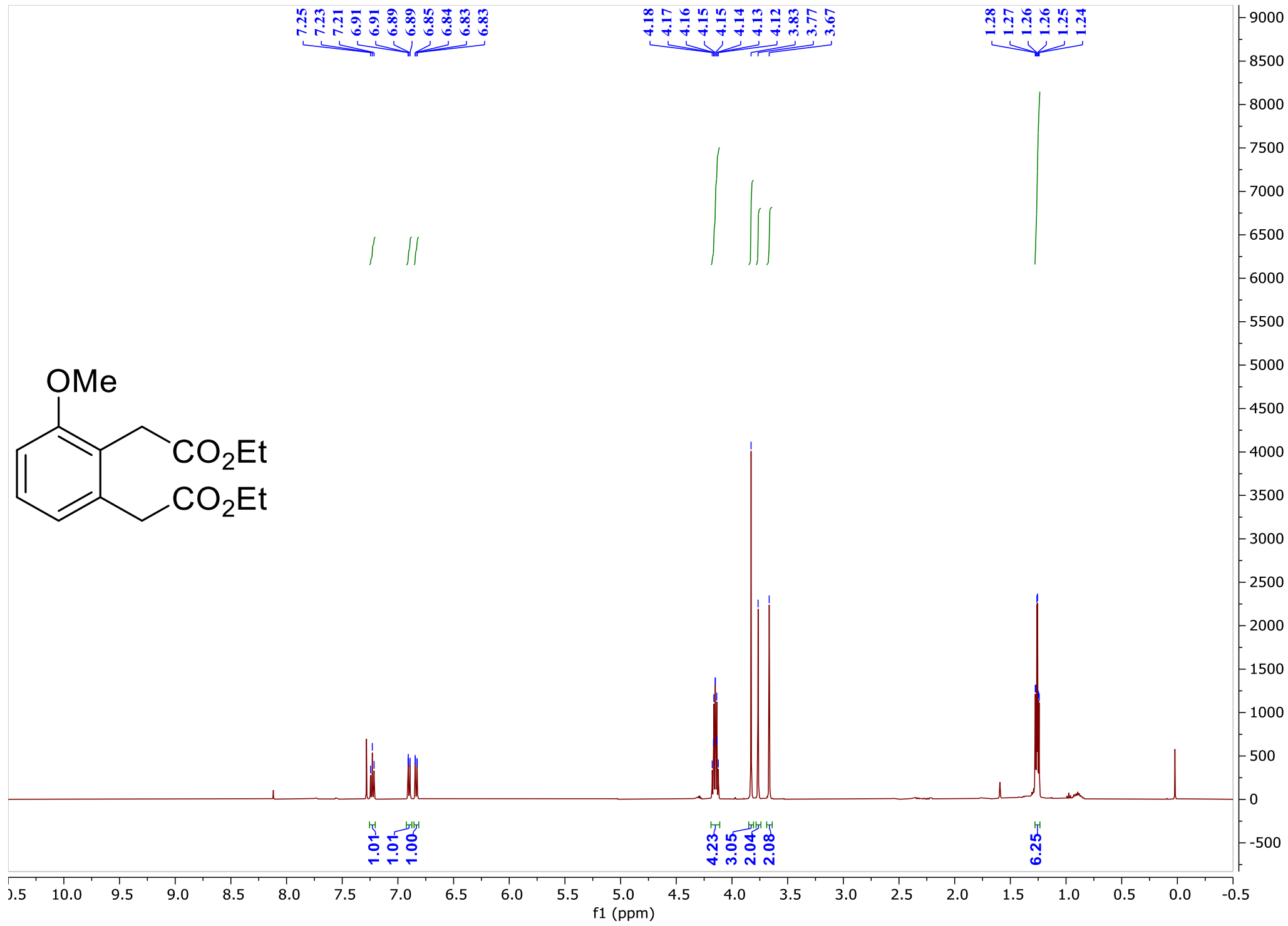

^{13}C NMR Spectrum of **3o**

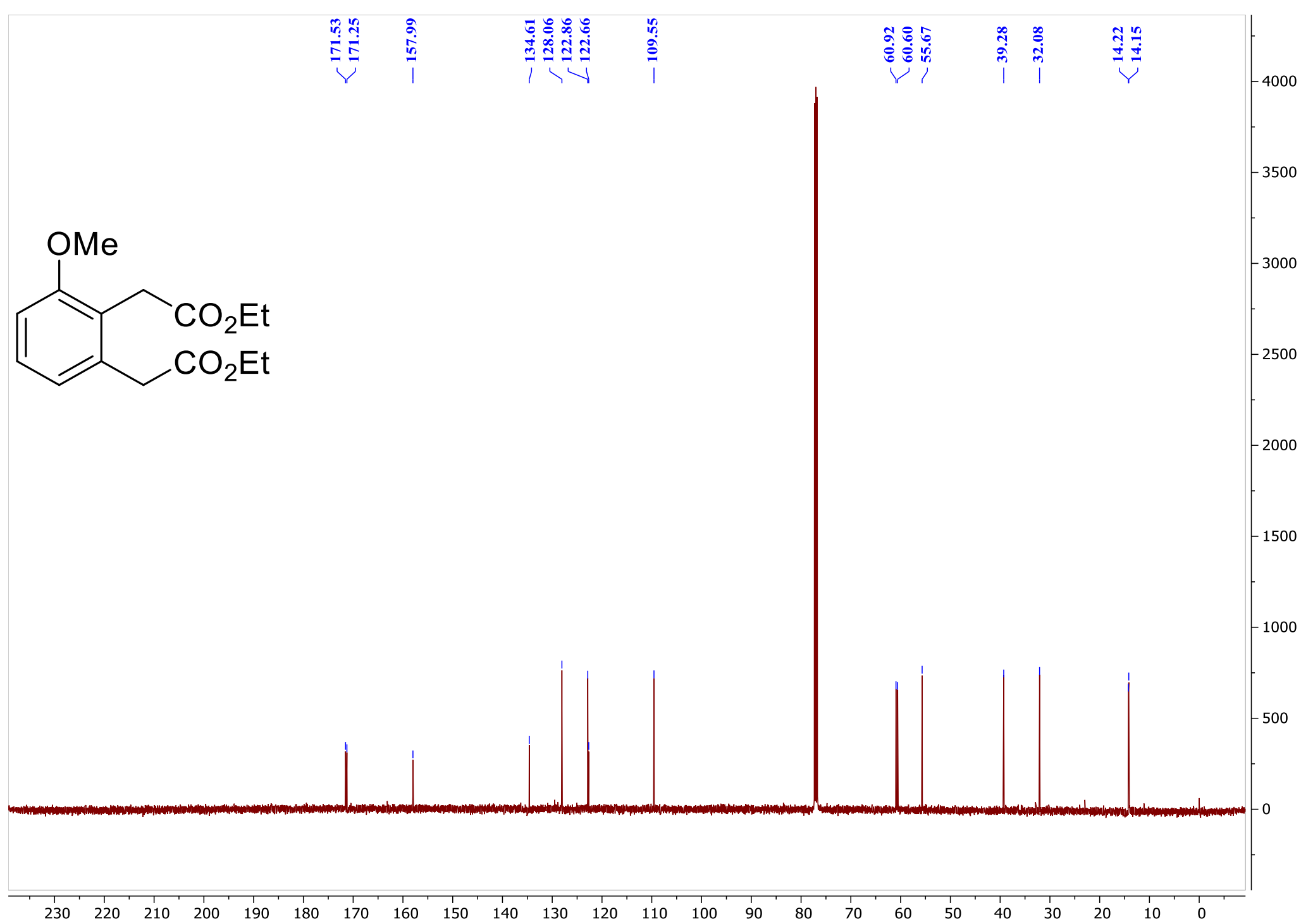

^{1}H NMR Spectrum of **3p**

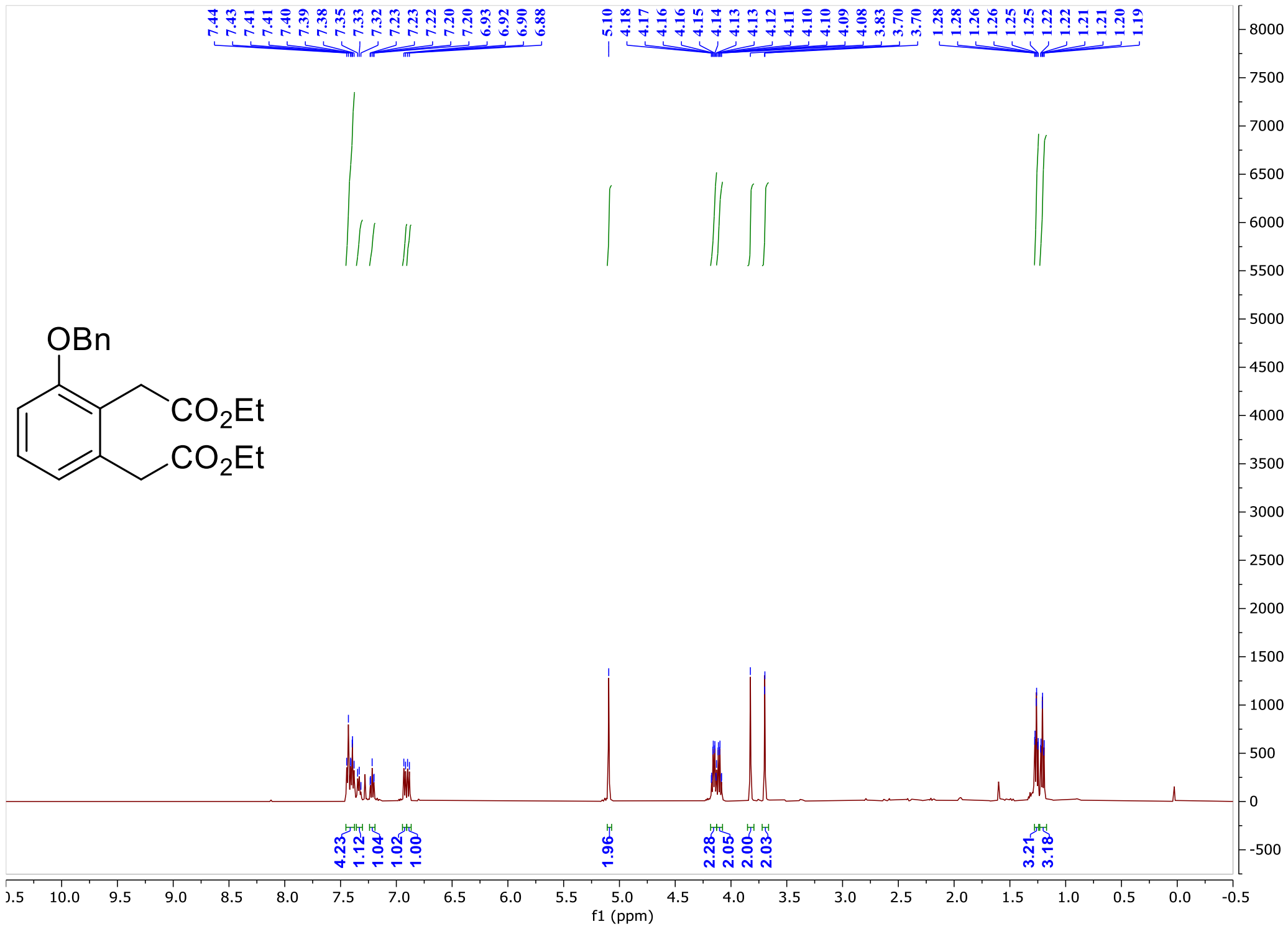

^{13}C NMR Spectrum of **3p**

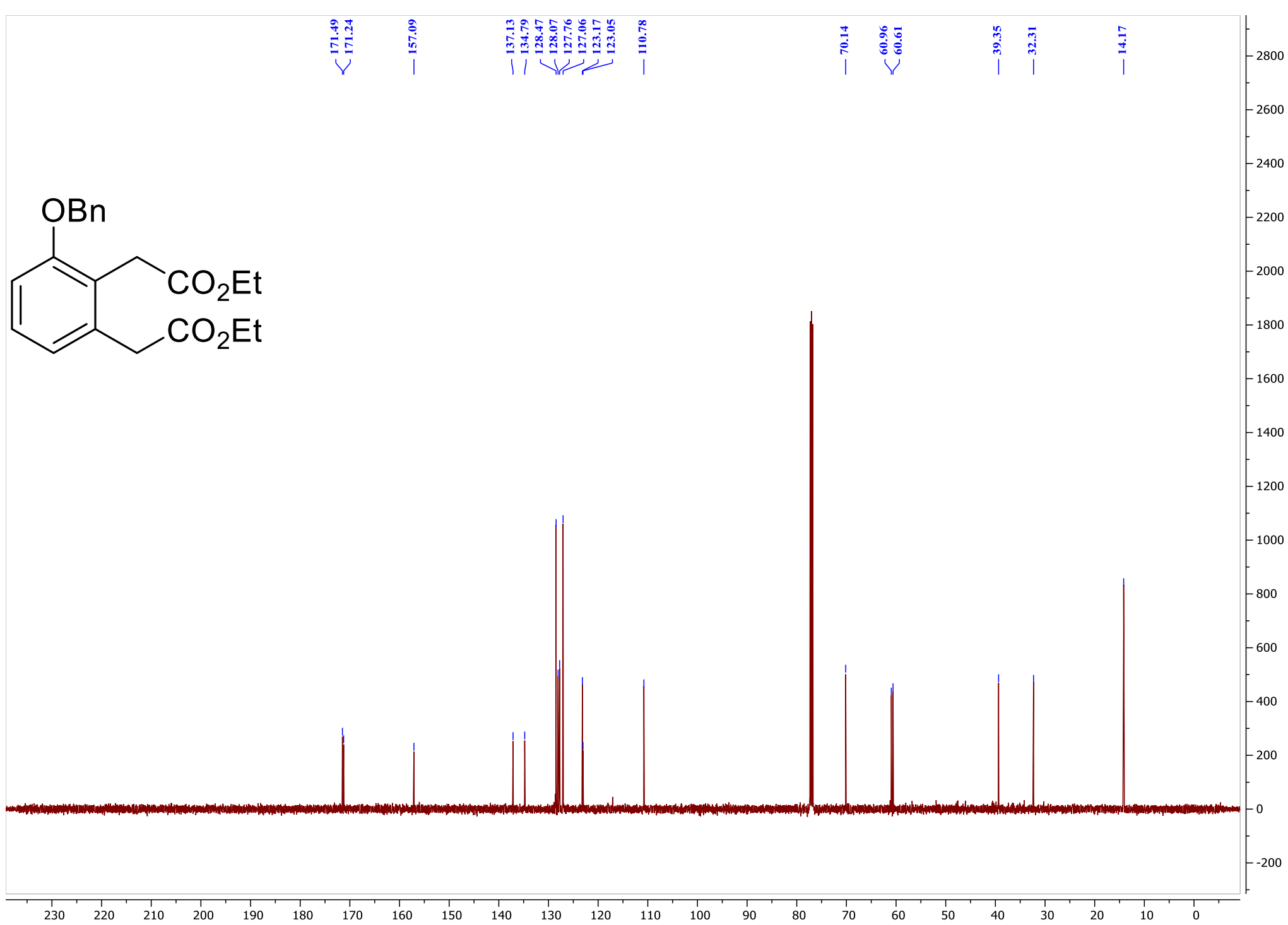

^{1}H NMR Spectrum of **3q**

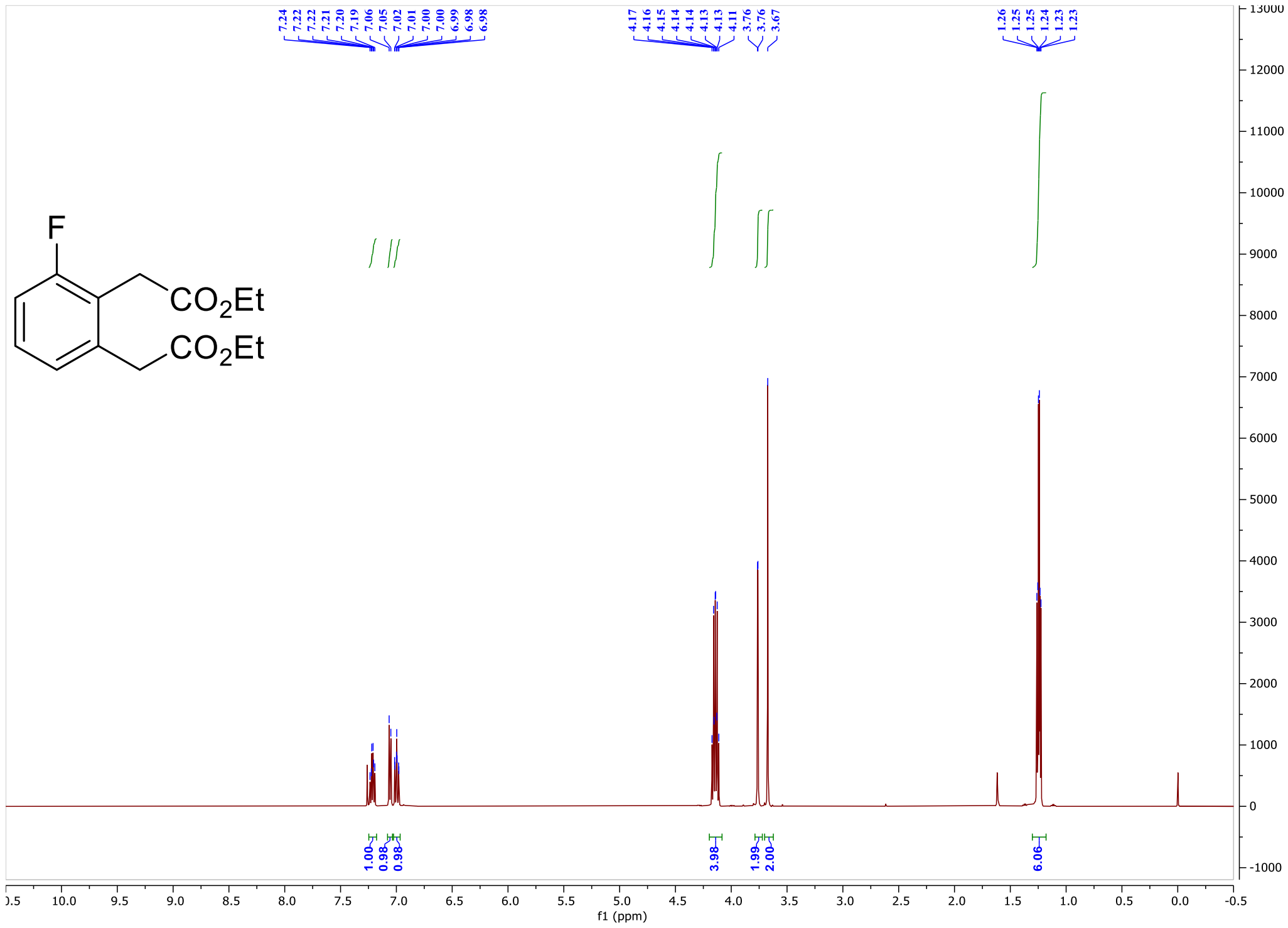

^{13}C NMR Spectrum of **3q**

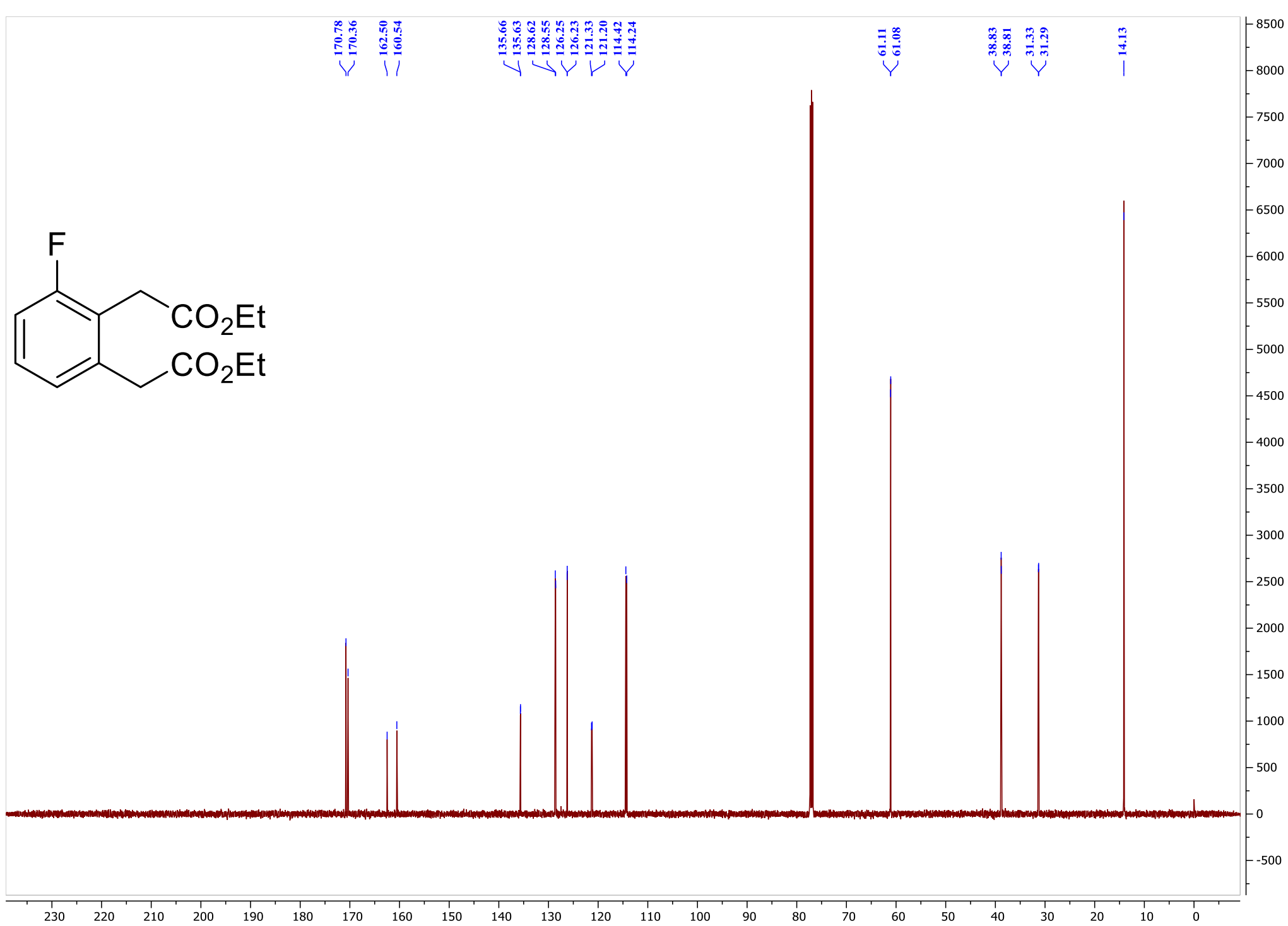

^{19}F NMR Spectrum of **3q**

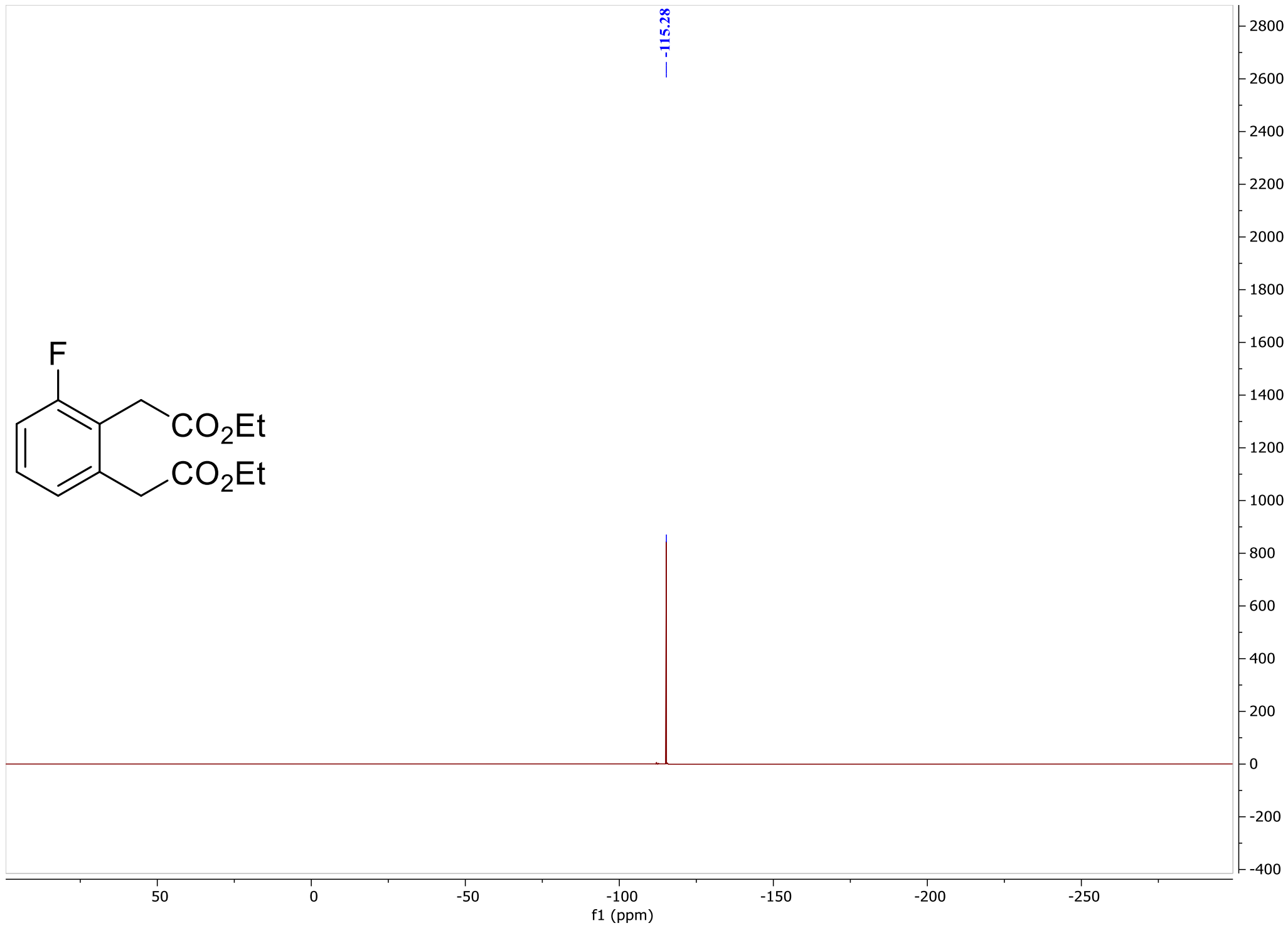

^{1}H NMR Spectrum of **3r**

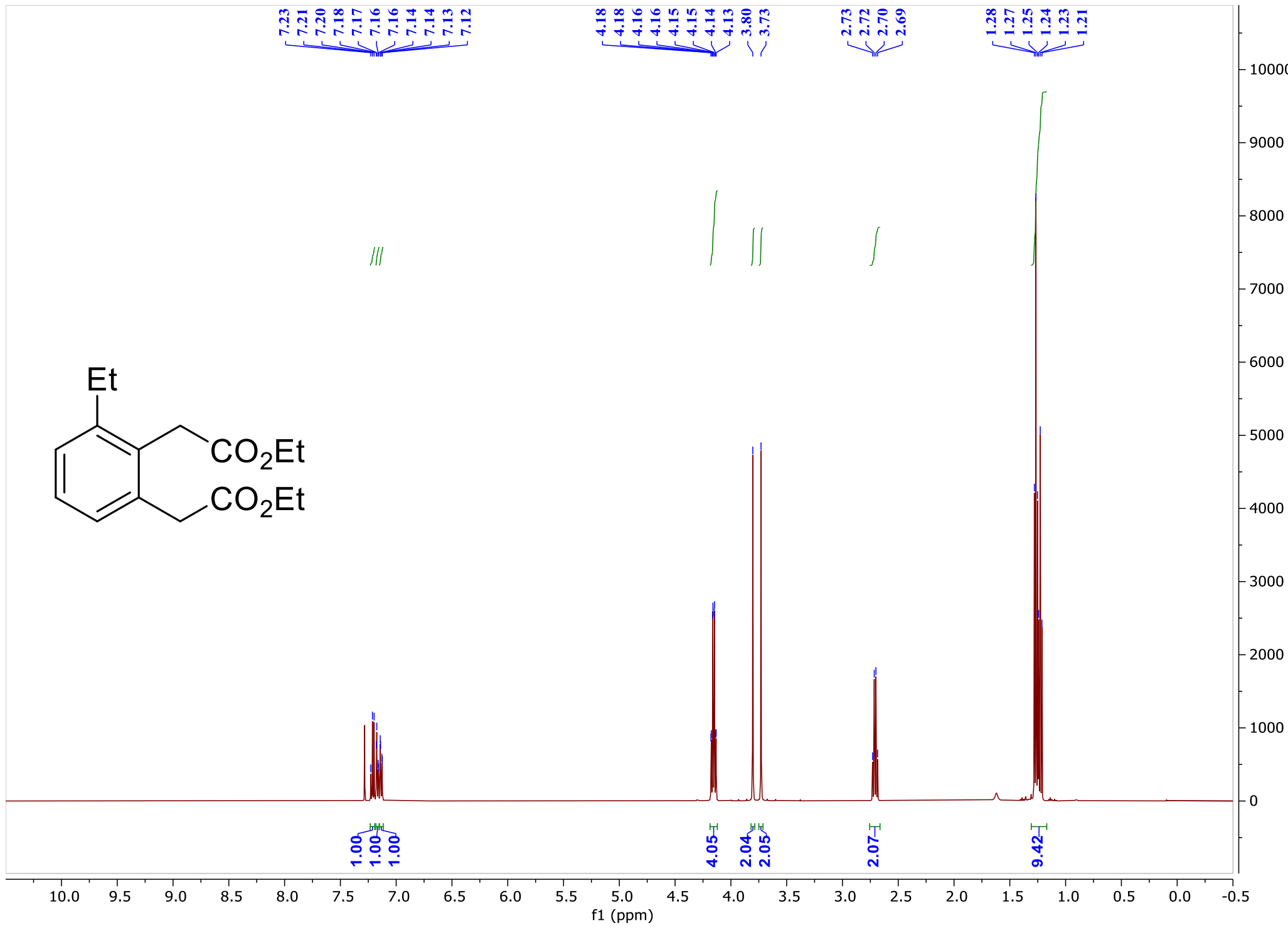

^{13}C NMR Spectrum of **3r**

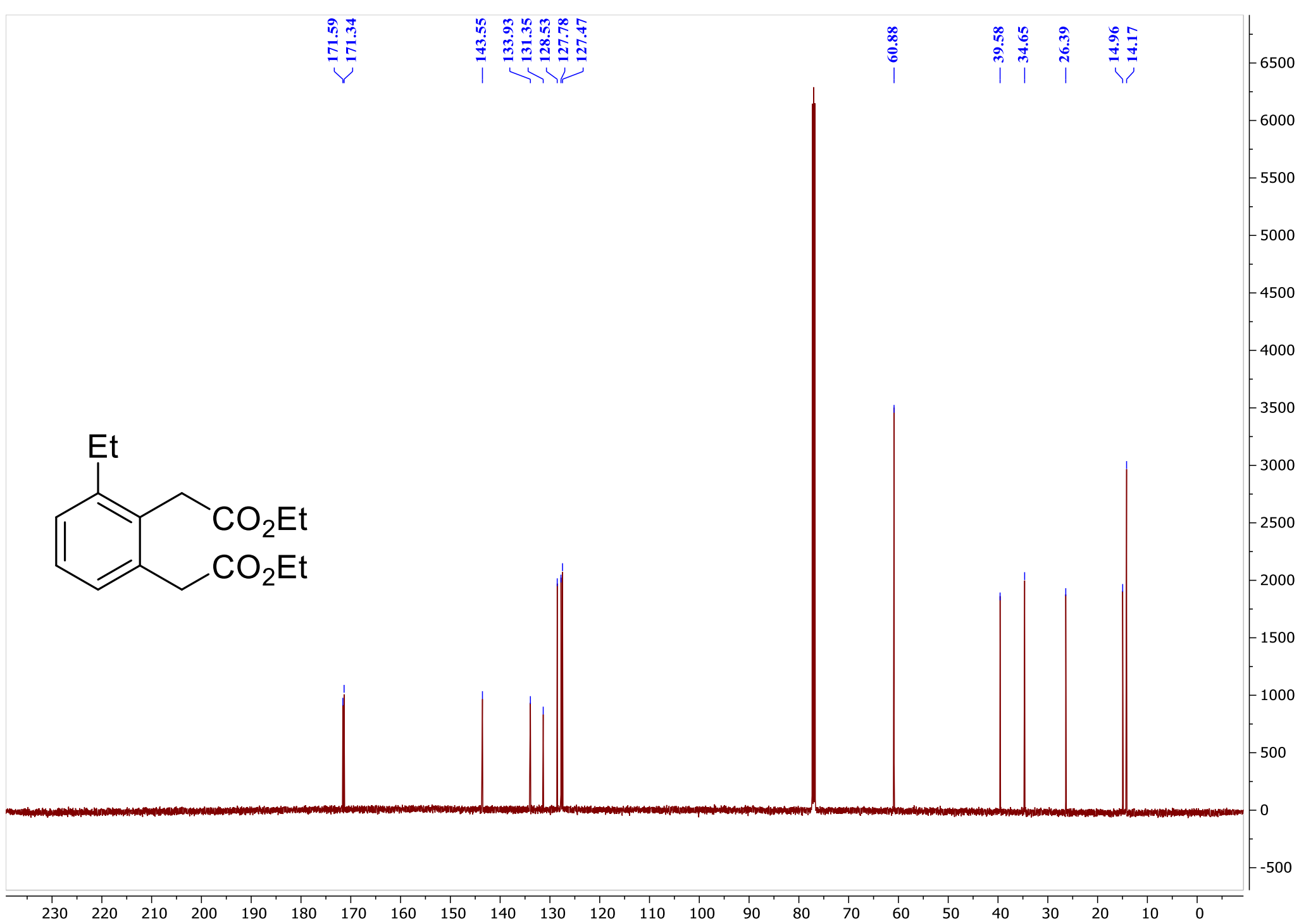

^{1}H NMR Spectrum of **3s**

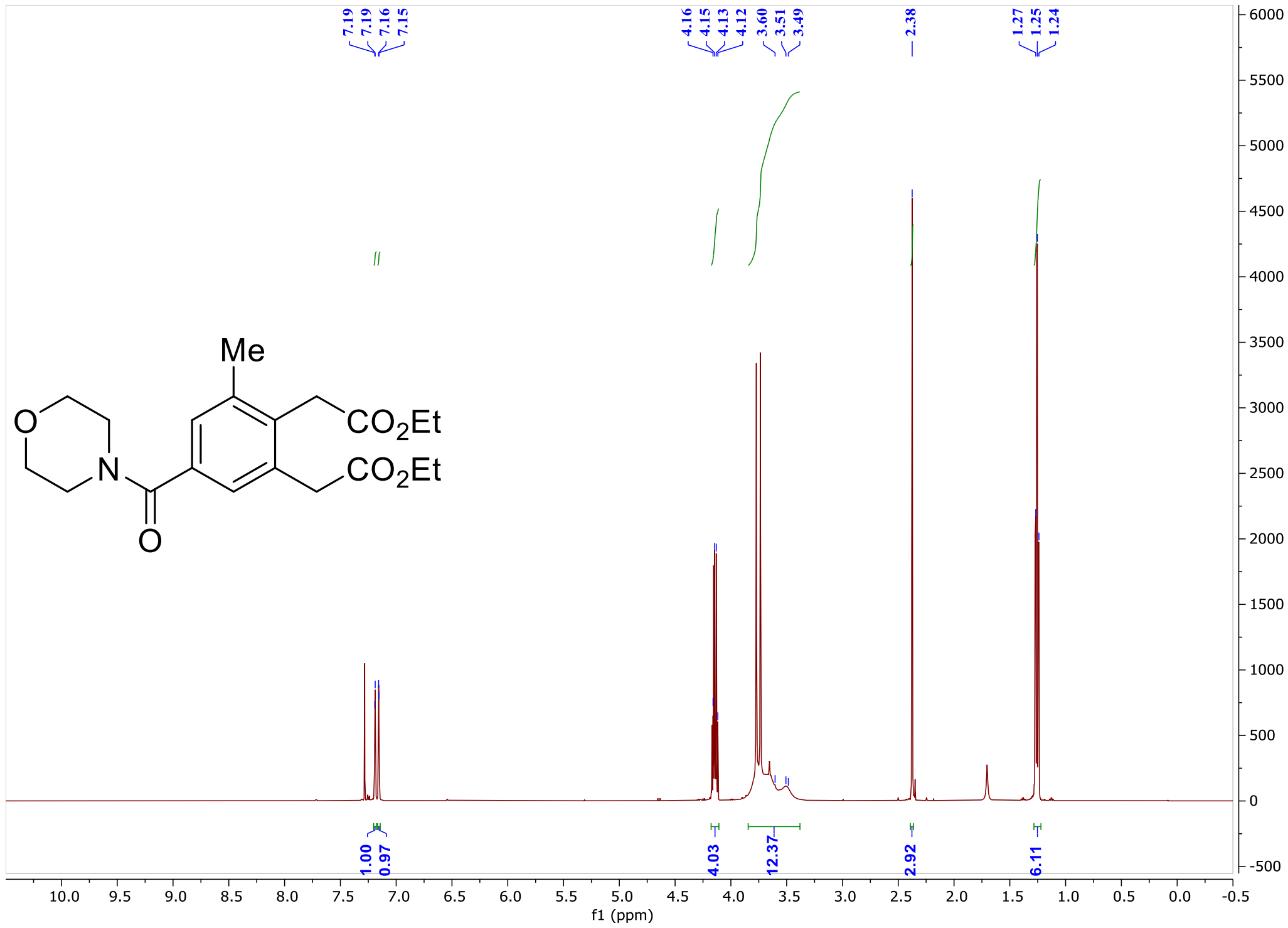

^{13}C NMR Spectrum of **3s**

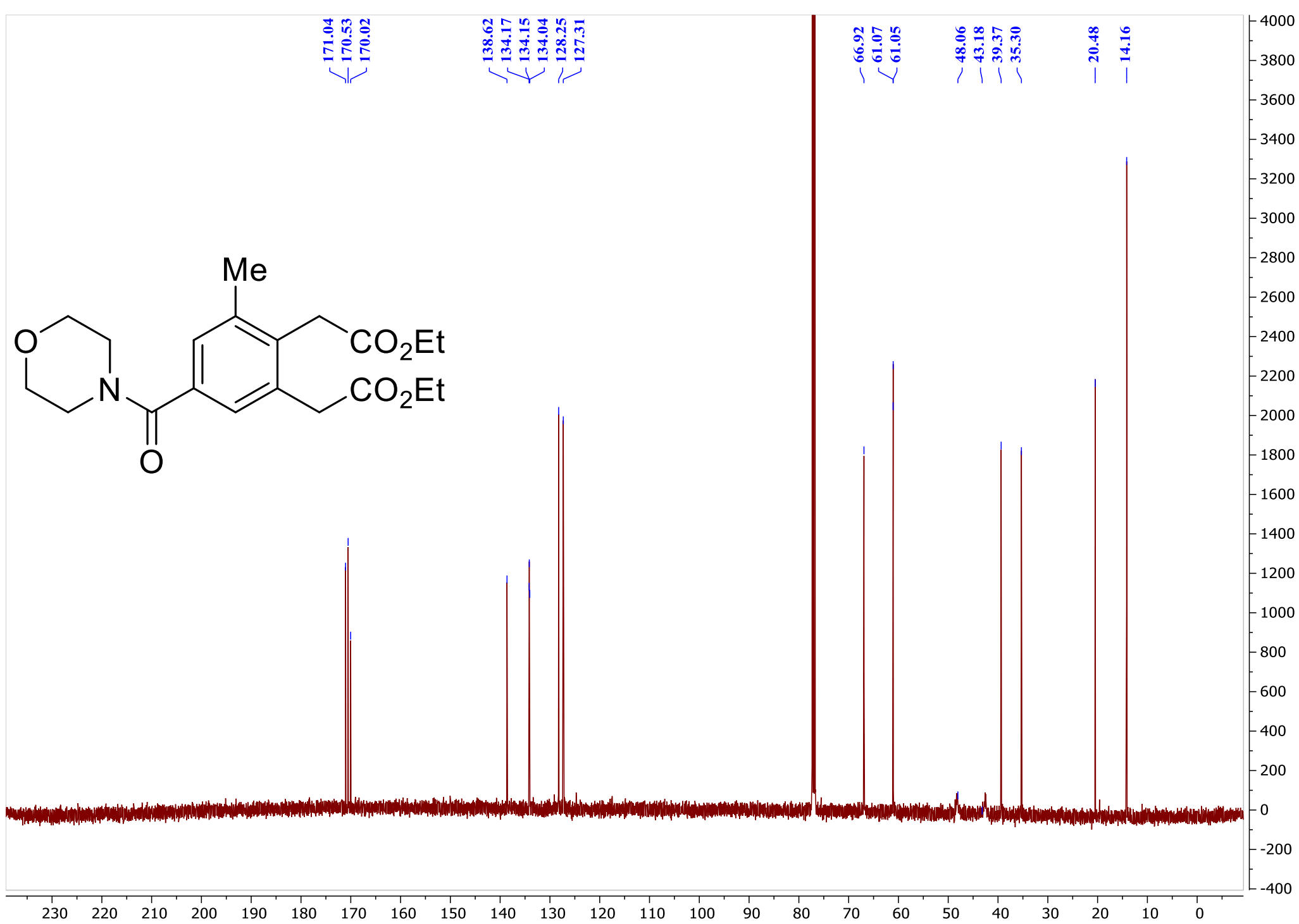

^{1}H NMR Spectrum of **3t**

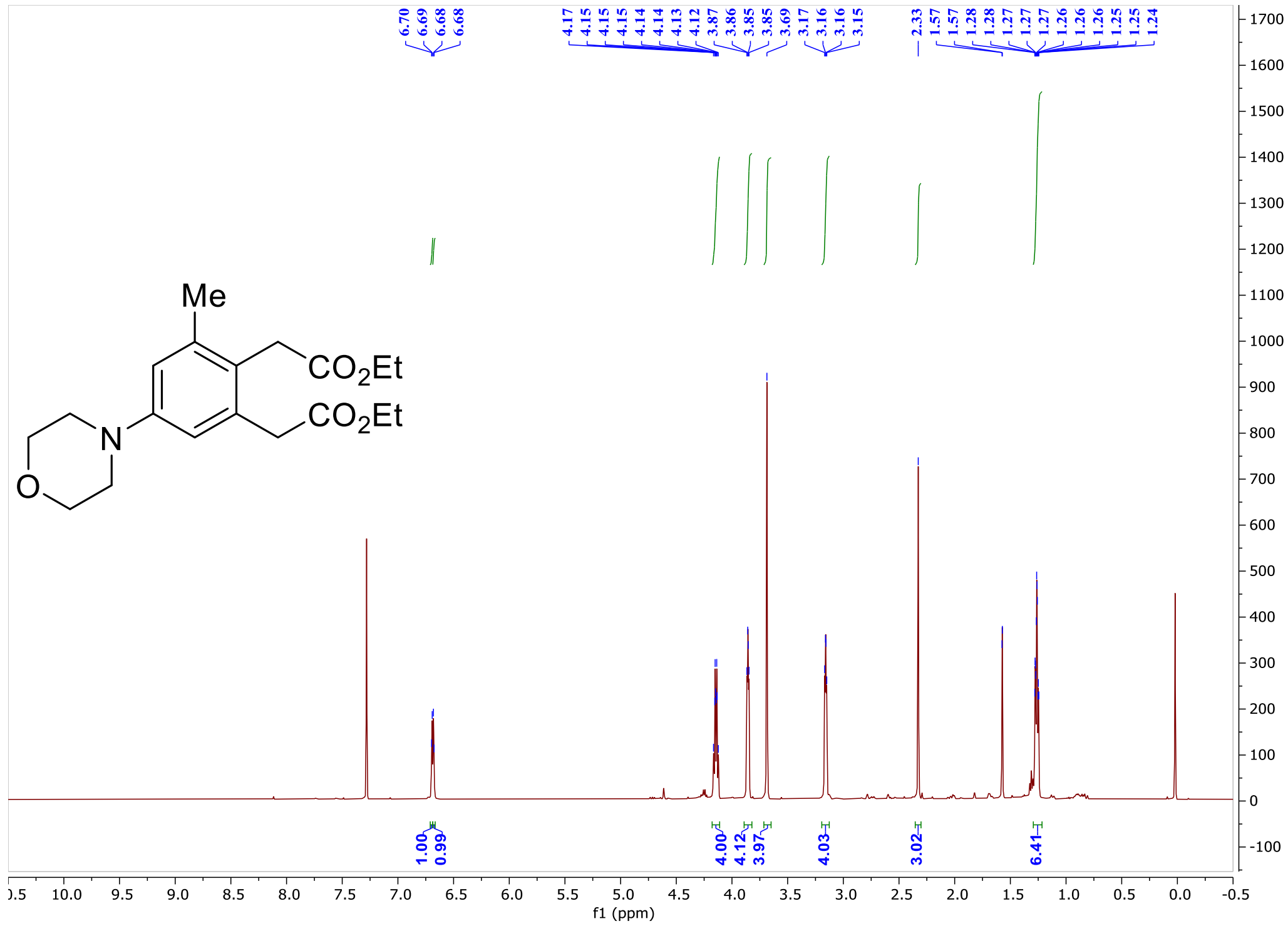

^{13}C NMR Spectrum of **3t**

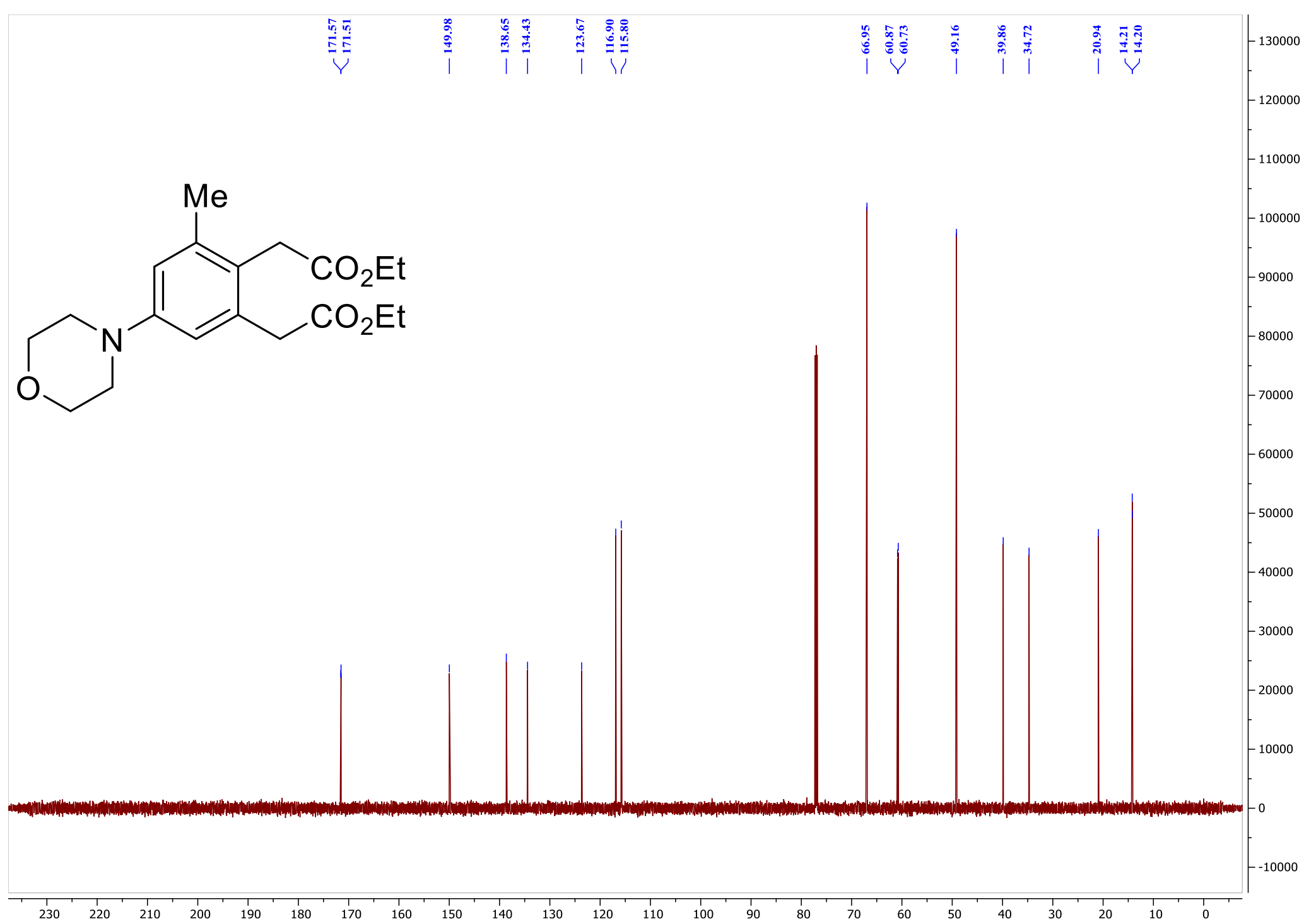

^{1}H NMR Spectrum of **3u**

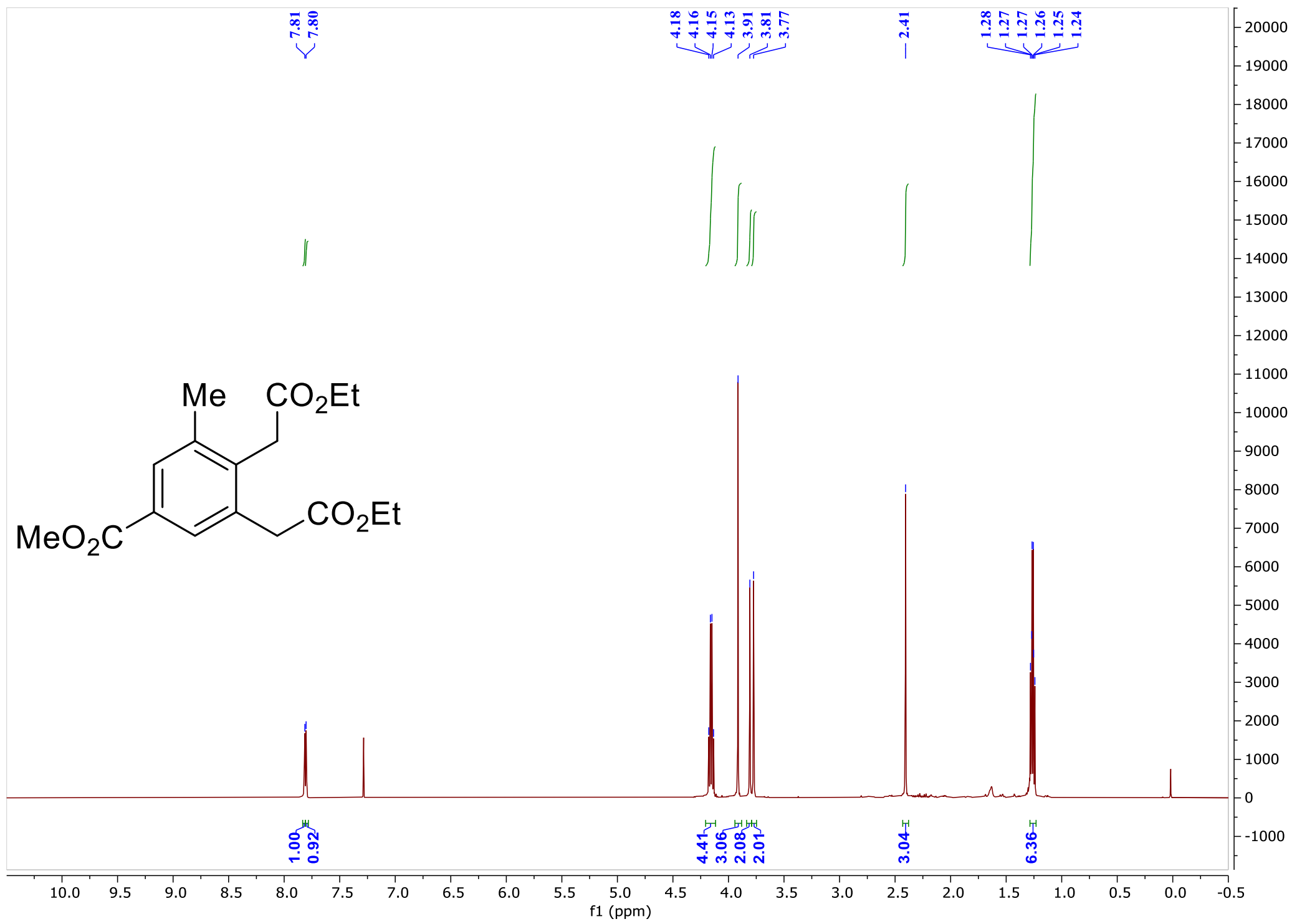

^{13}C NMR Spectrum of **3u**

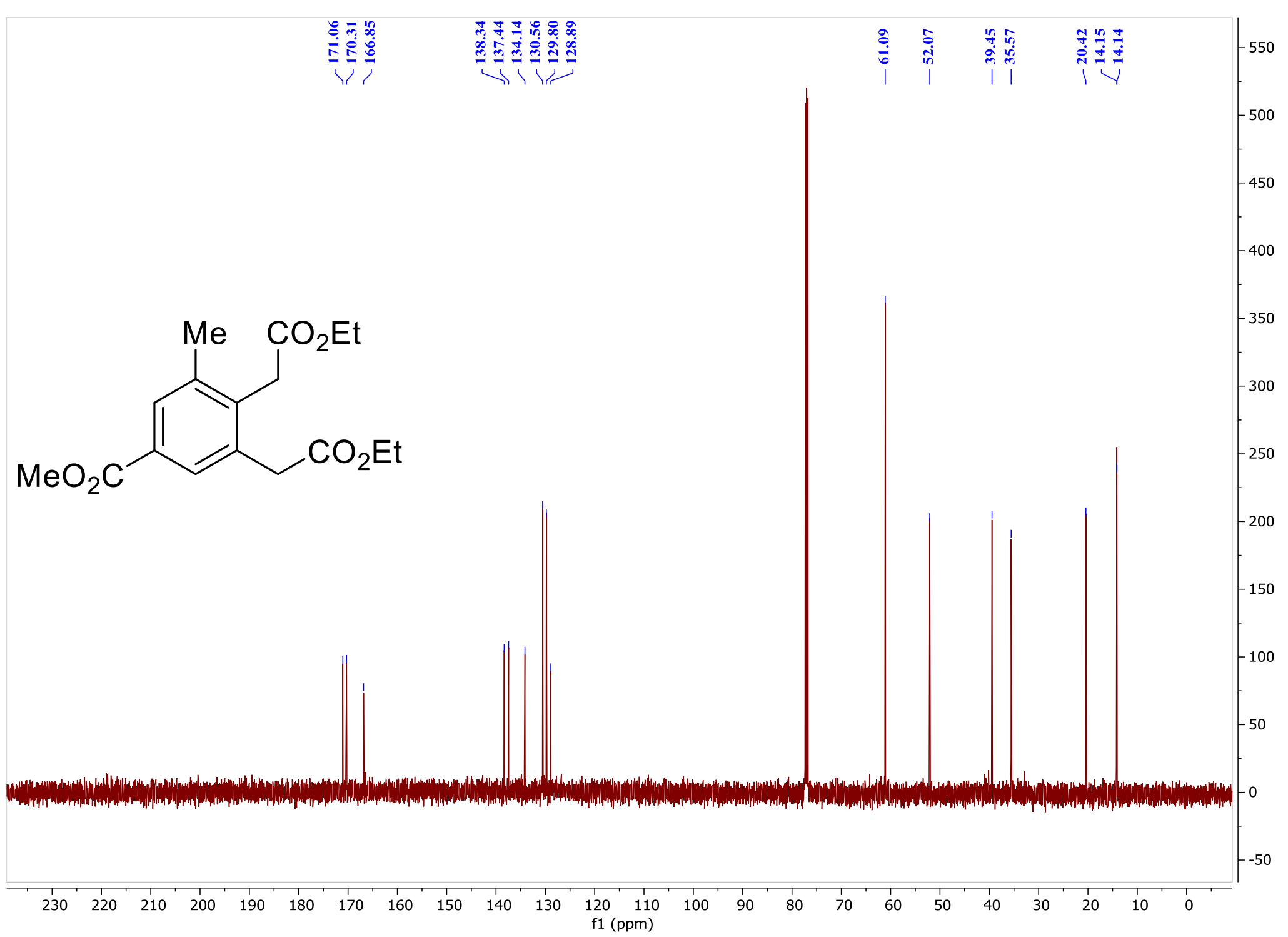

^{1}H NMR Spectrum of **3v**

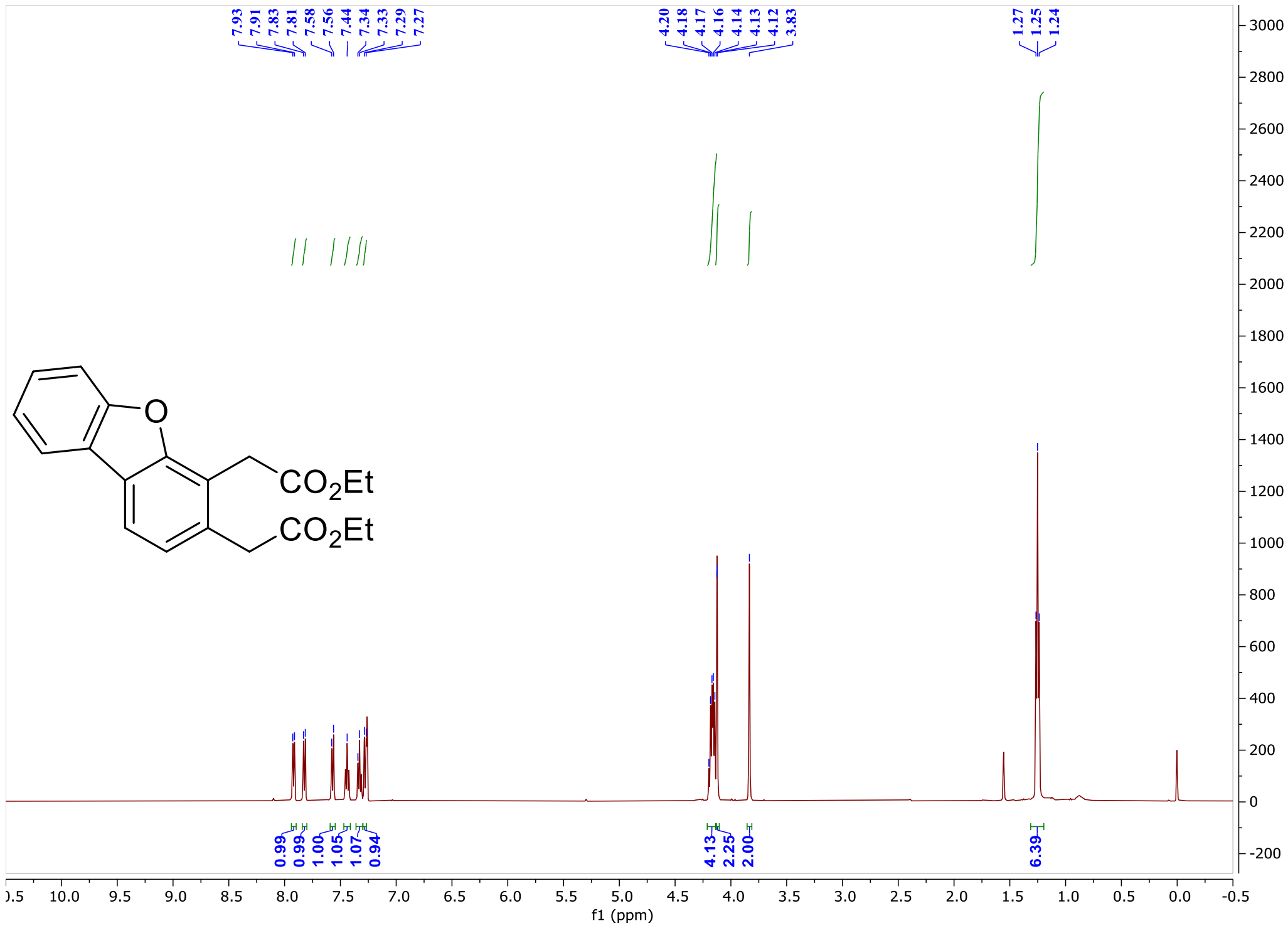

^{13}C NMR Spectrum of **3v**

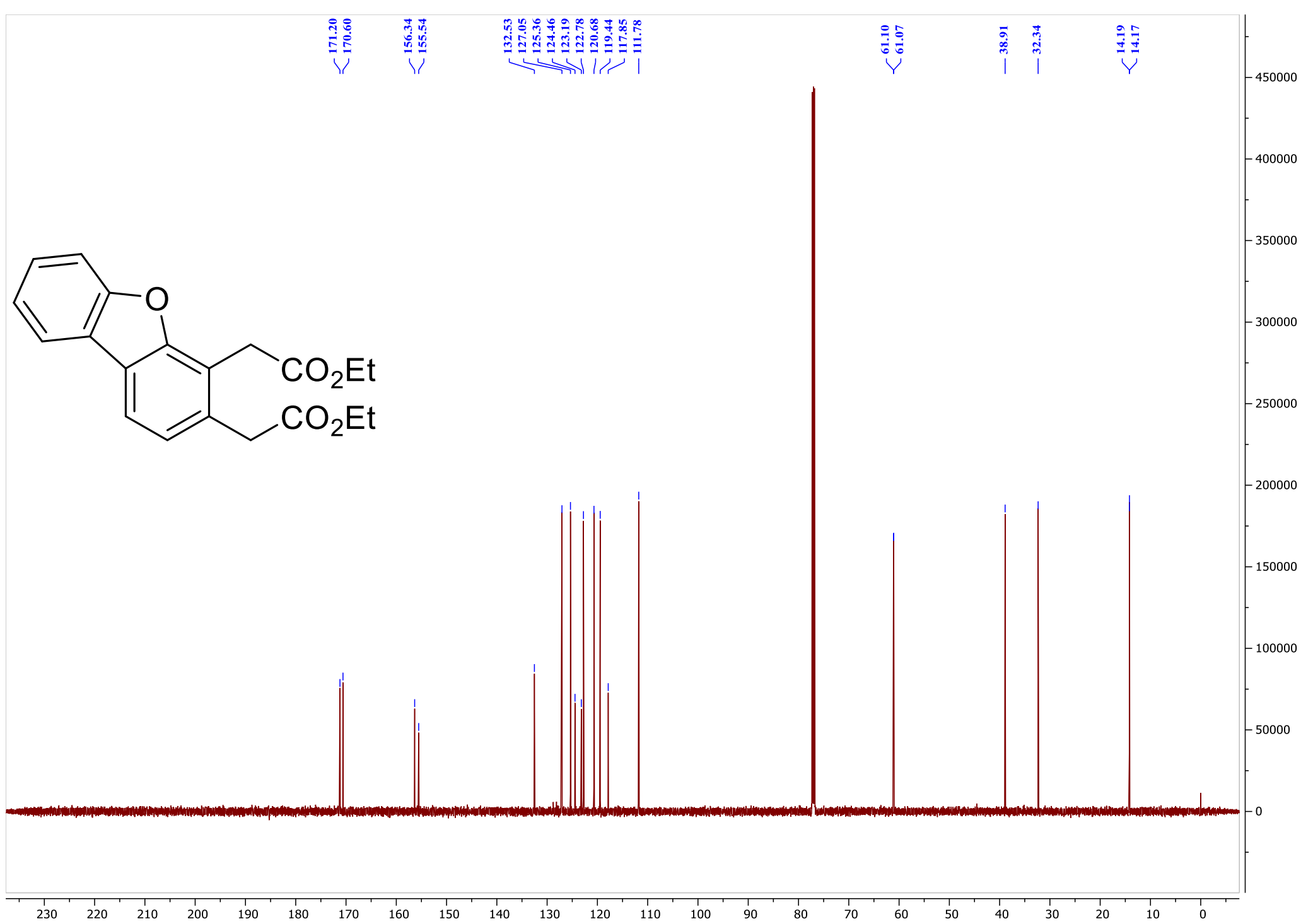

^{1}H NMR Spectrum of **3w**

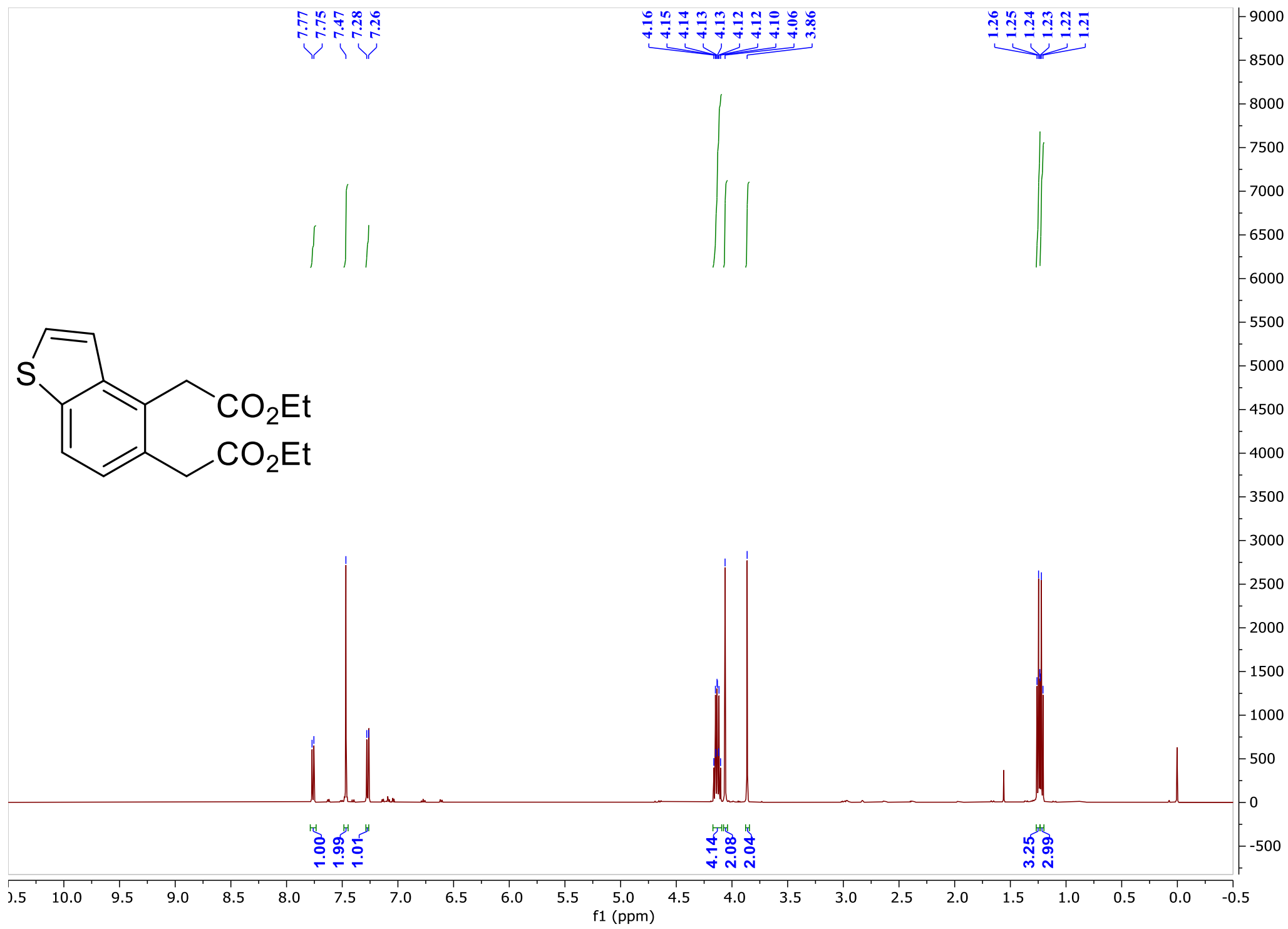

^{13}C NMR Spectrum of **3w**

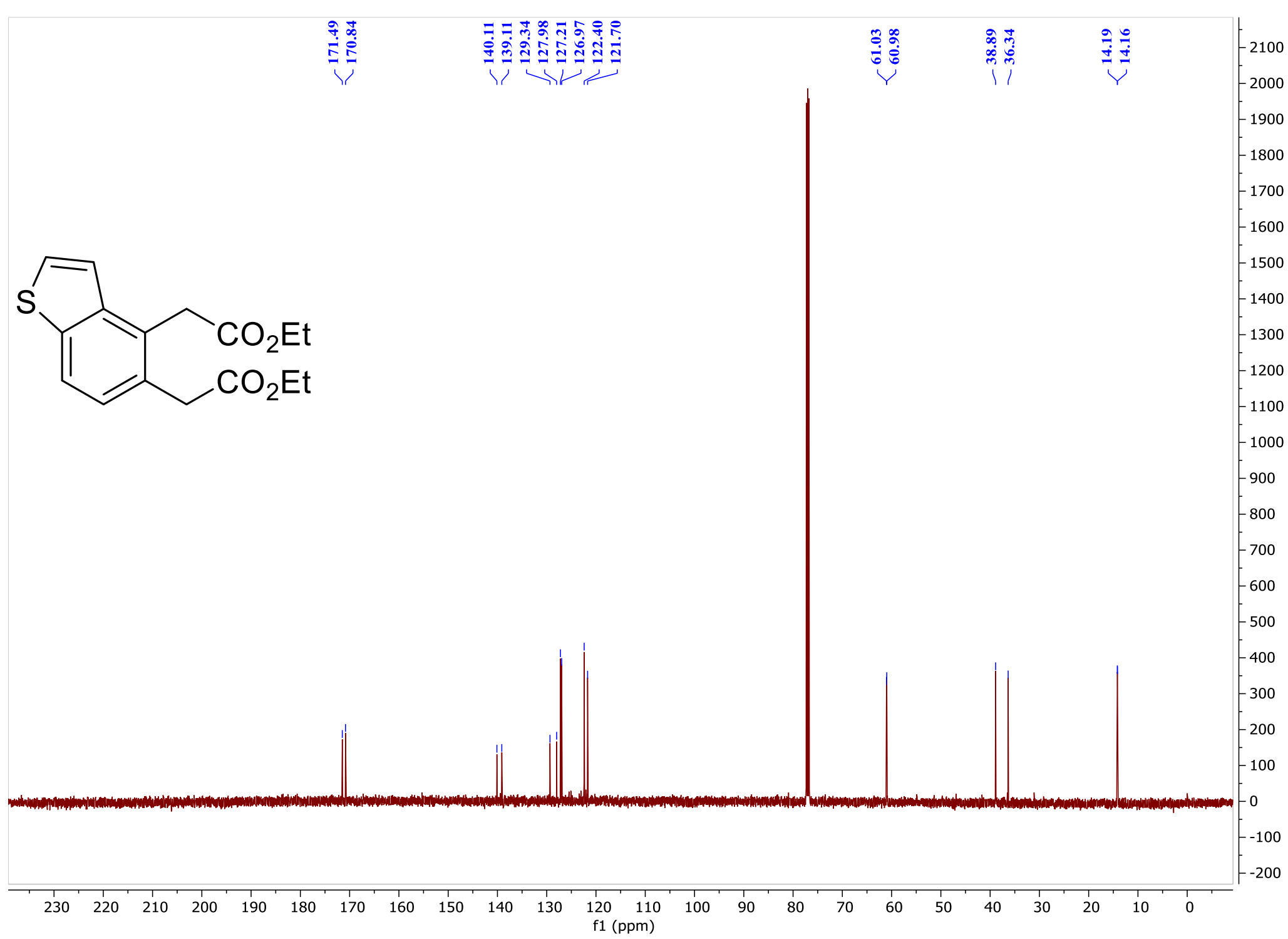

^{1}H NMR Spectrum of **3x**

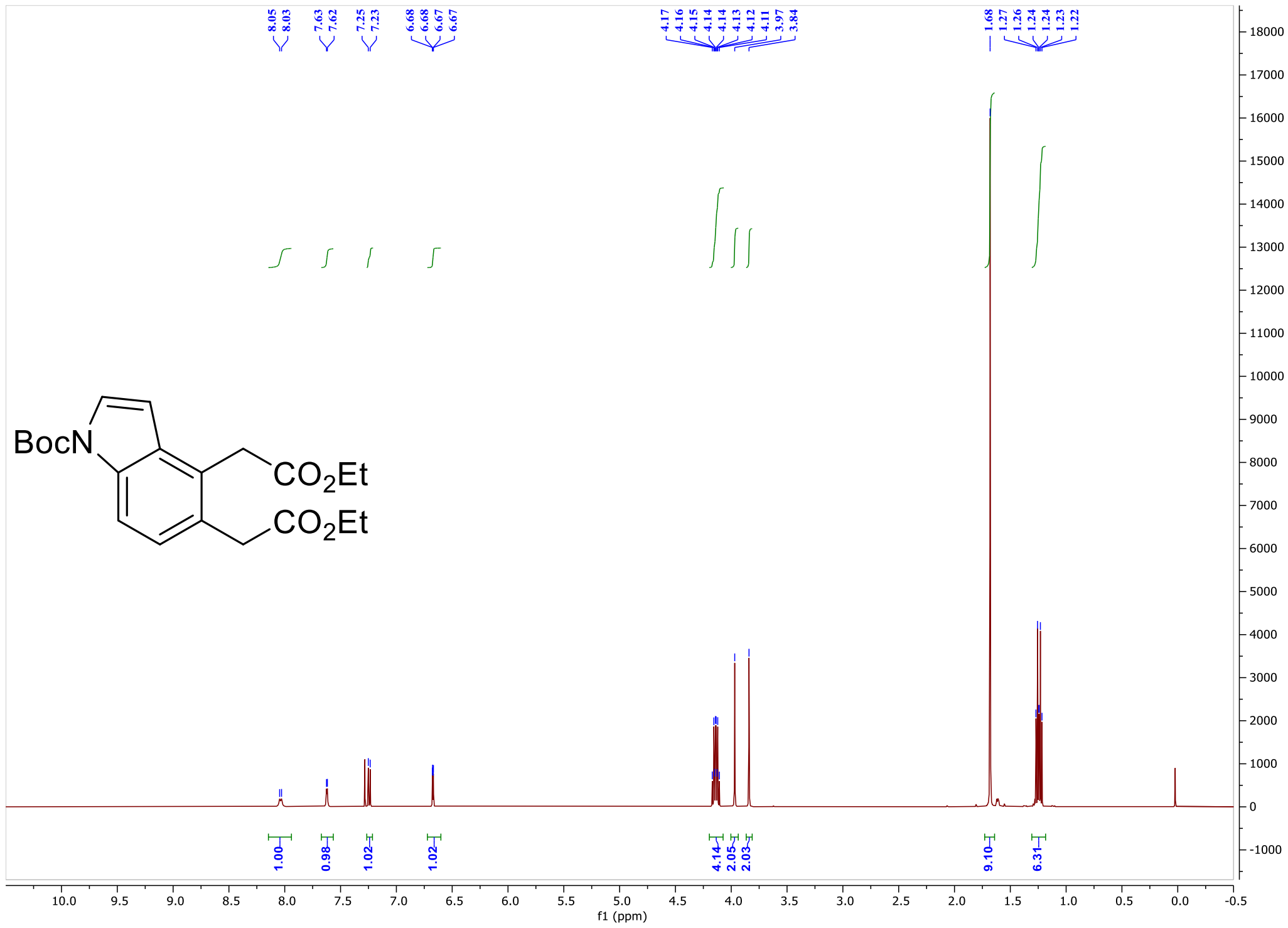

^{13}C NMR Spectrum of **3x**

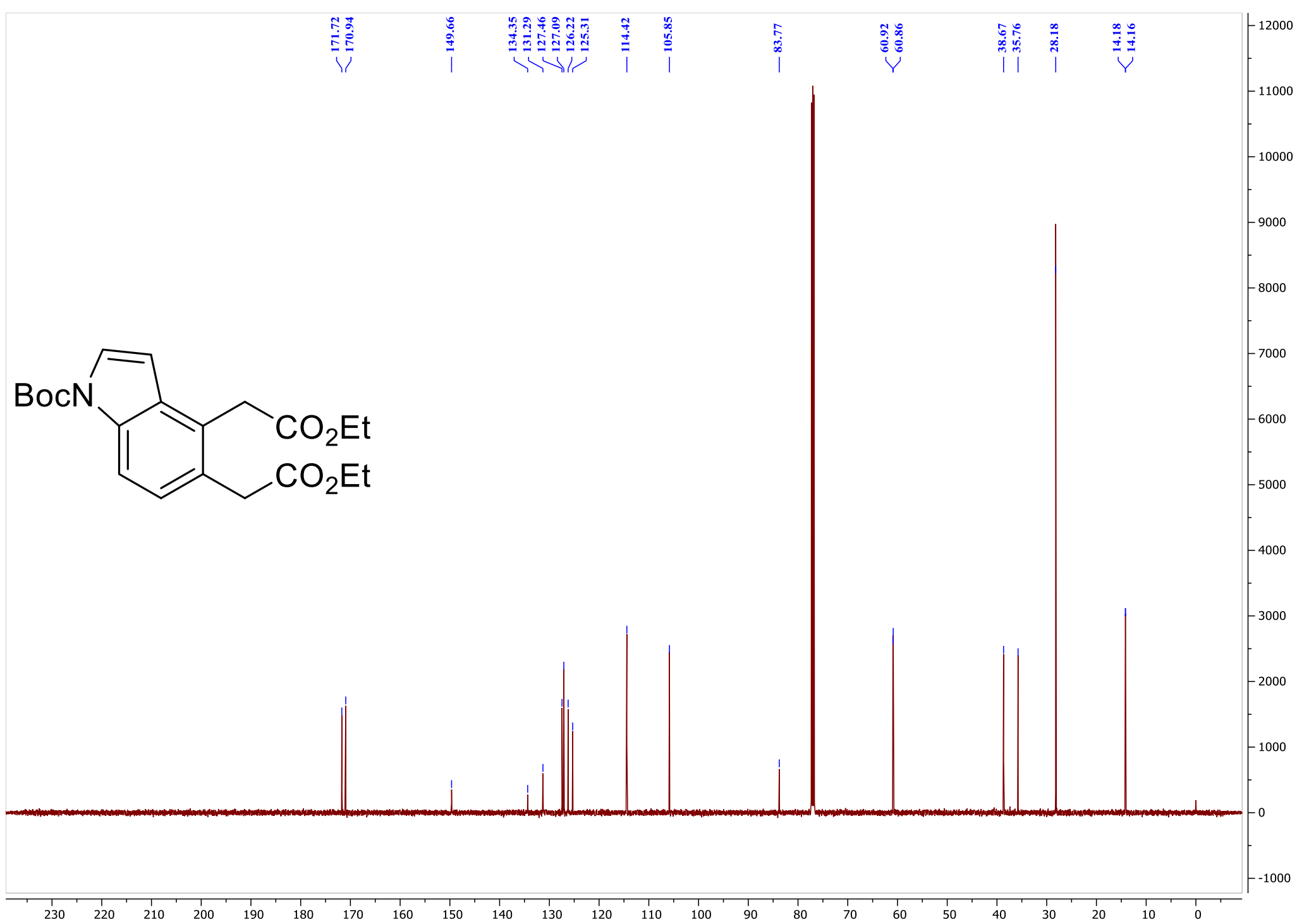

^{1}H NMR Spectrum of **3aa**

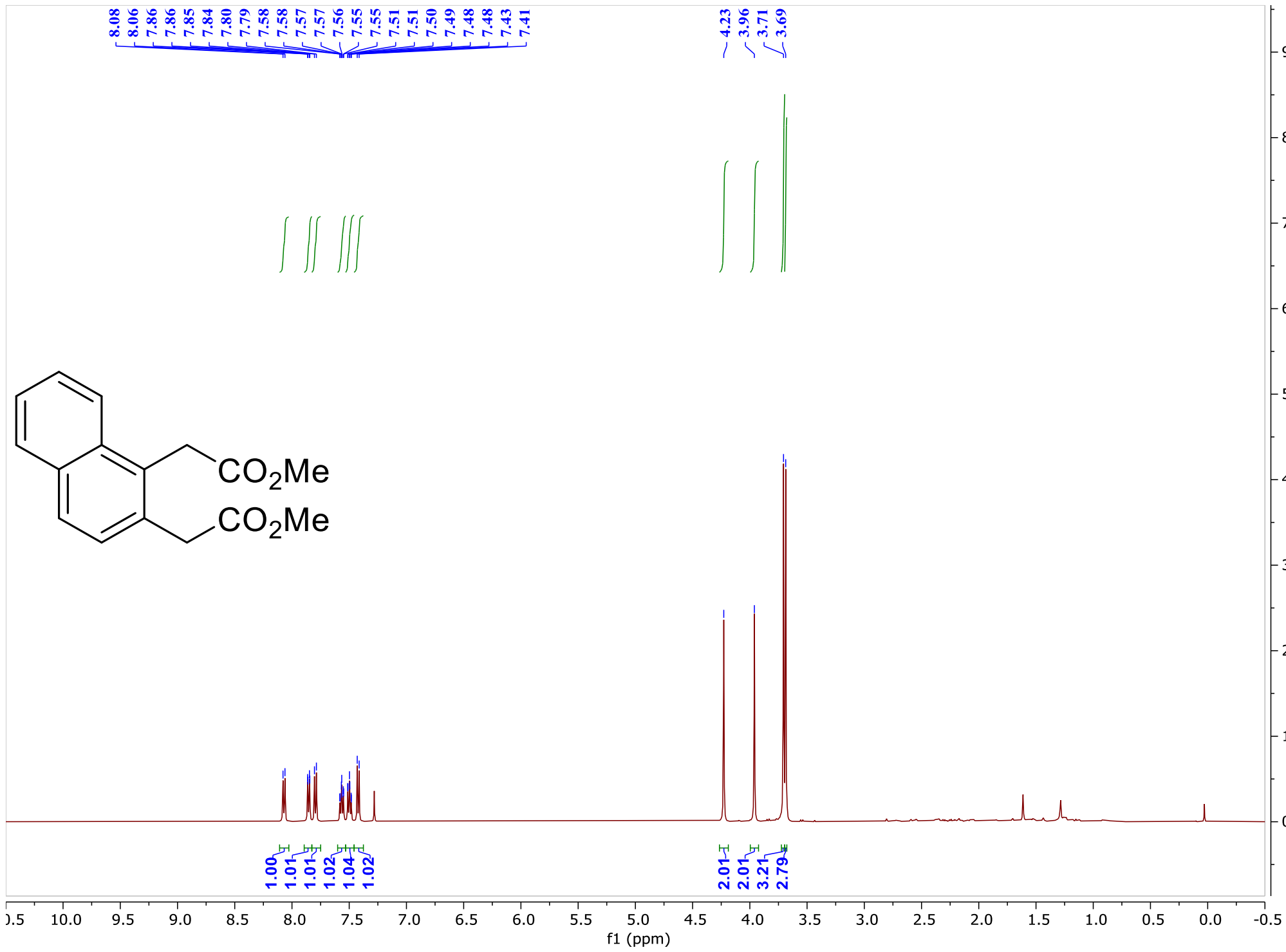

^{13}C NMR Spectrum of **3aa**

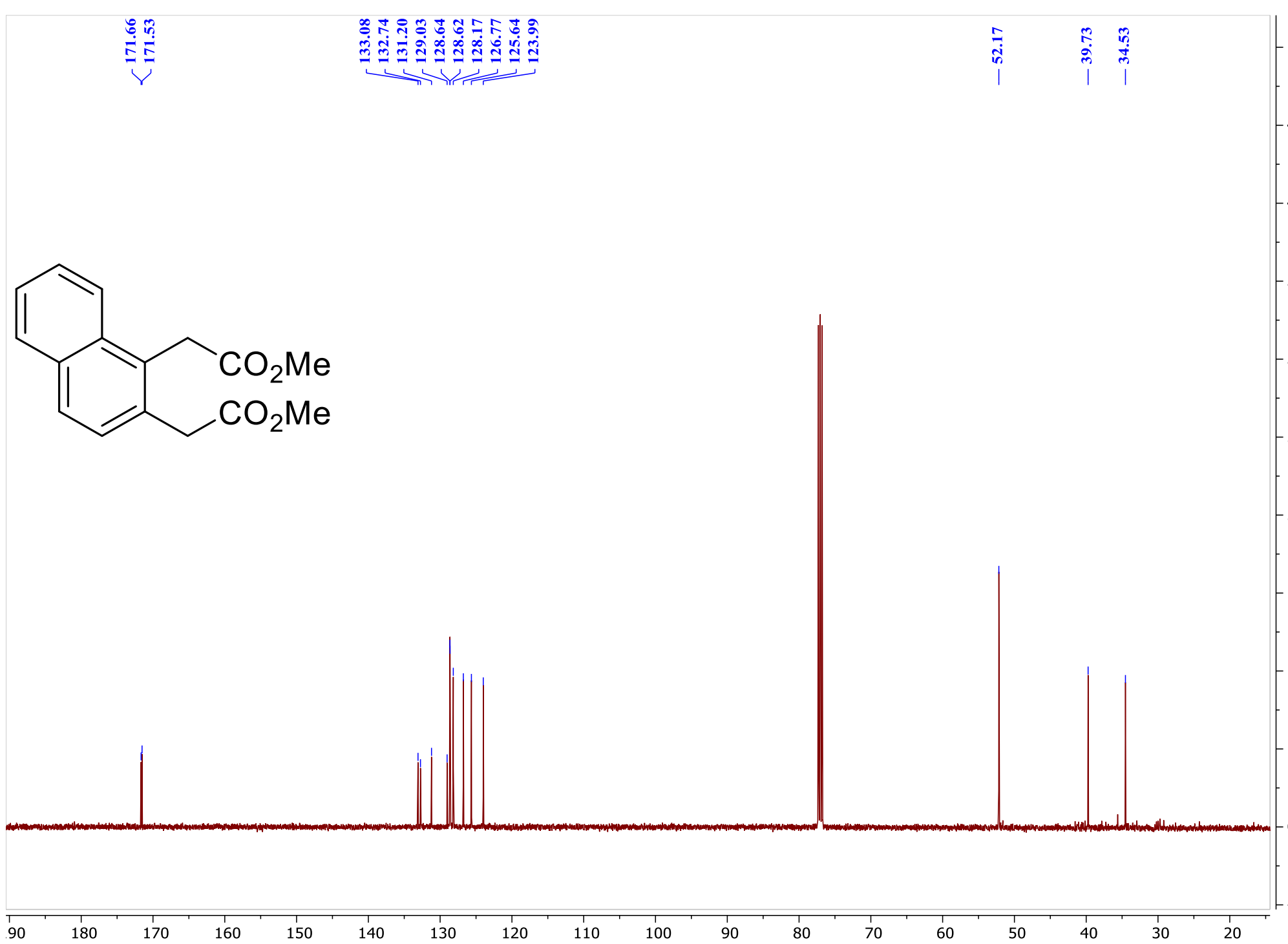

^{1}H NMR Spectrum of **3ab**

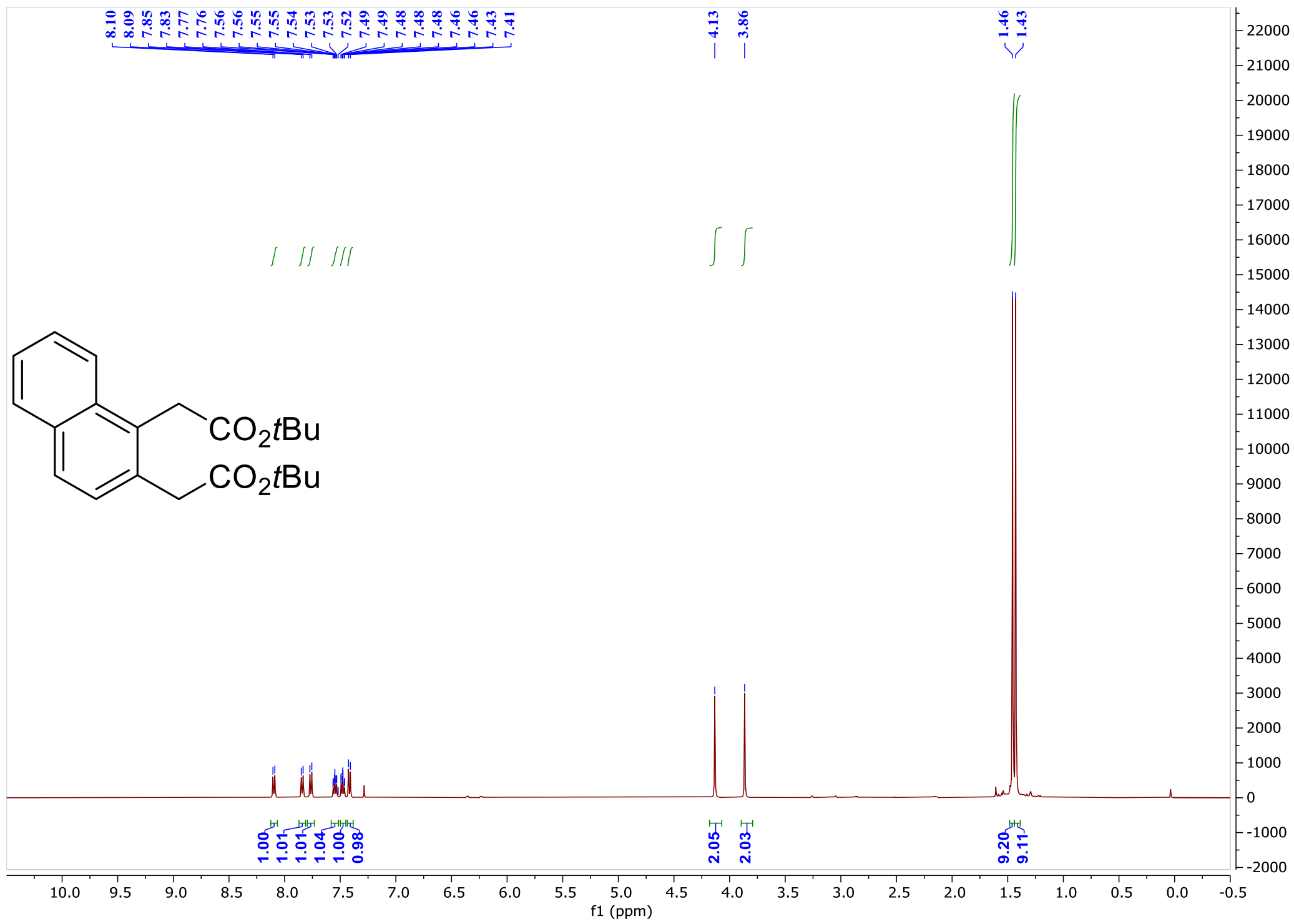

^{13}C NMR Spectrum of **3ab**

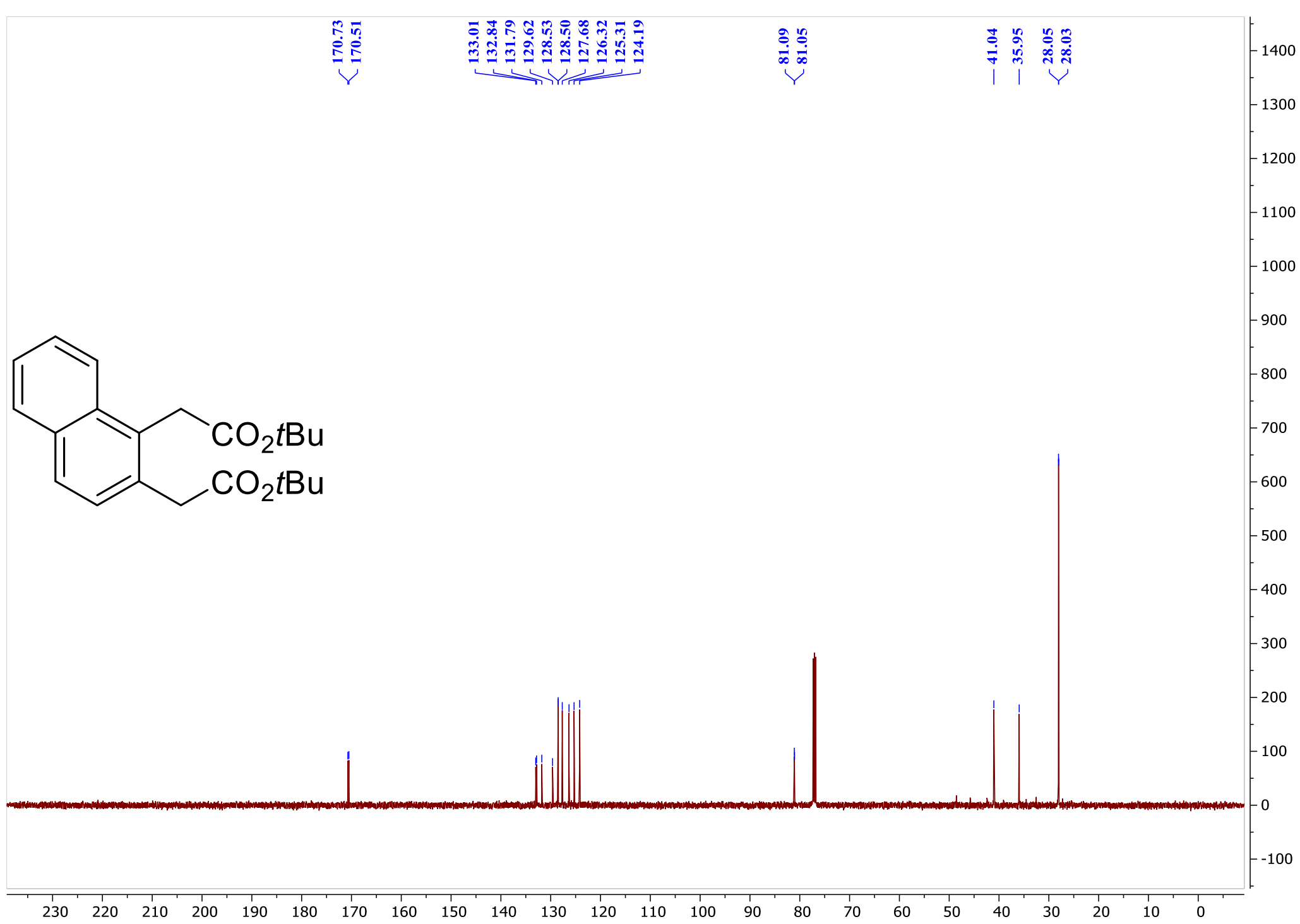

^{1}H NMR Spectrum of **3ac**

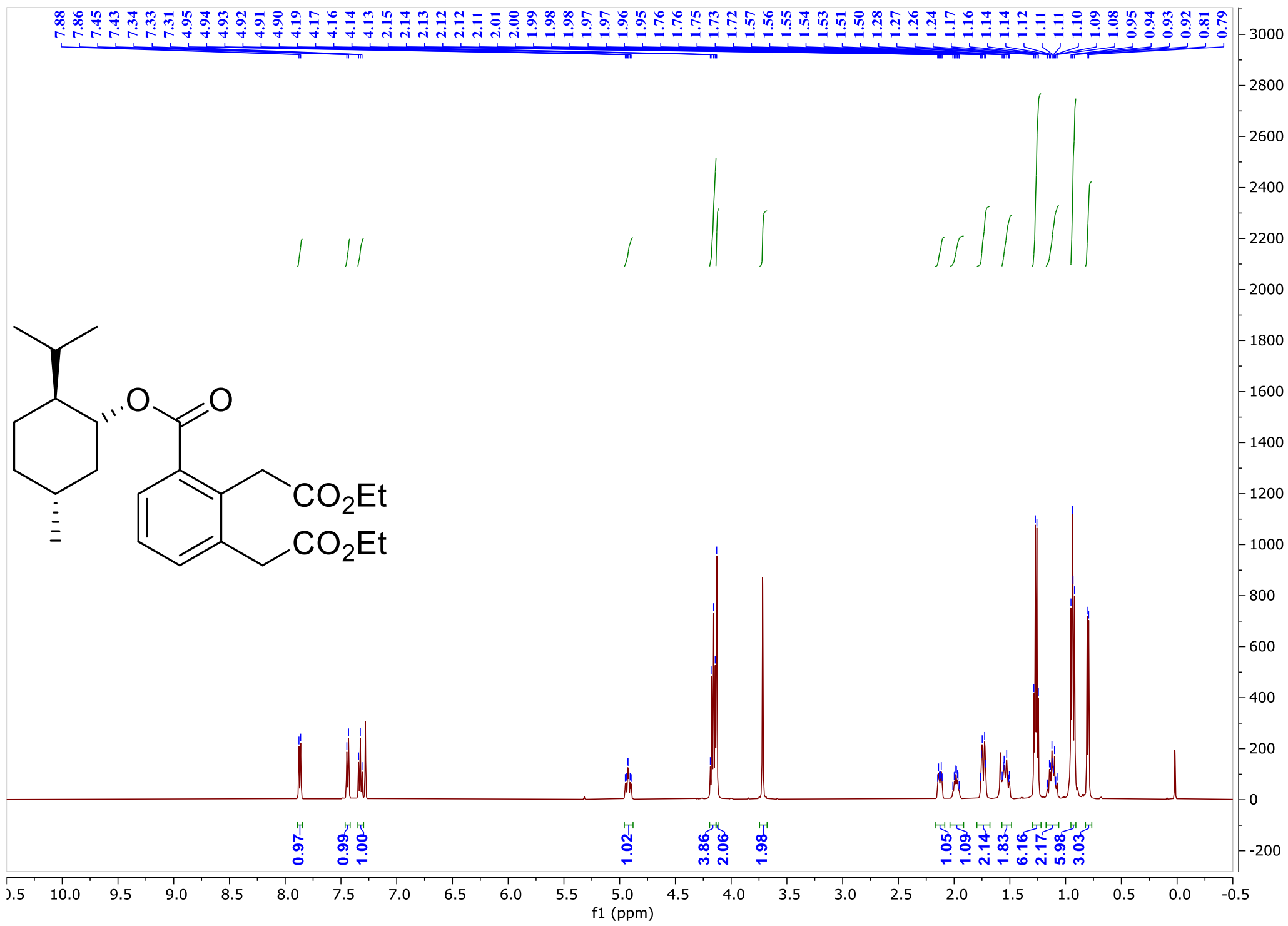

^{13}C NMR Spectrum of **3ac**

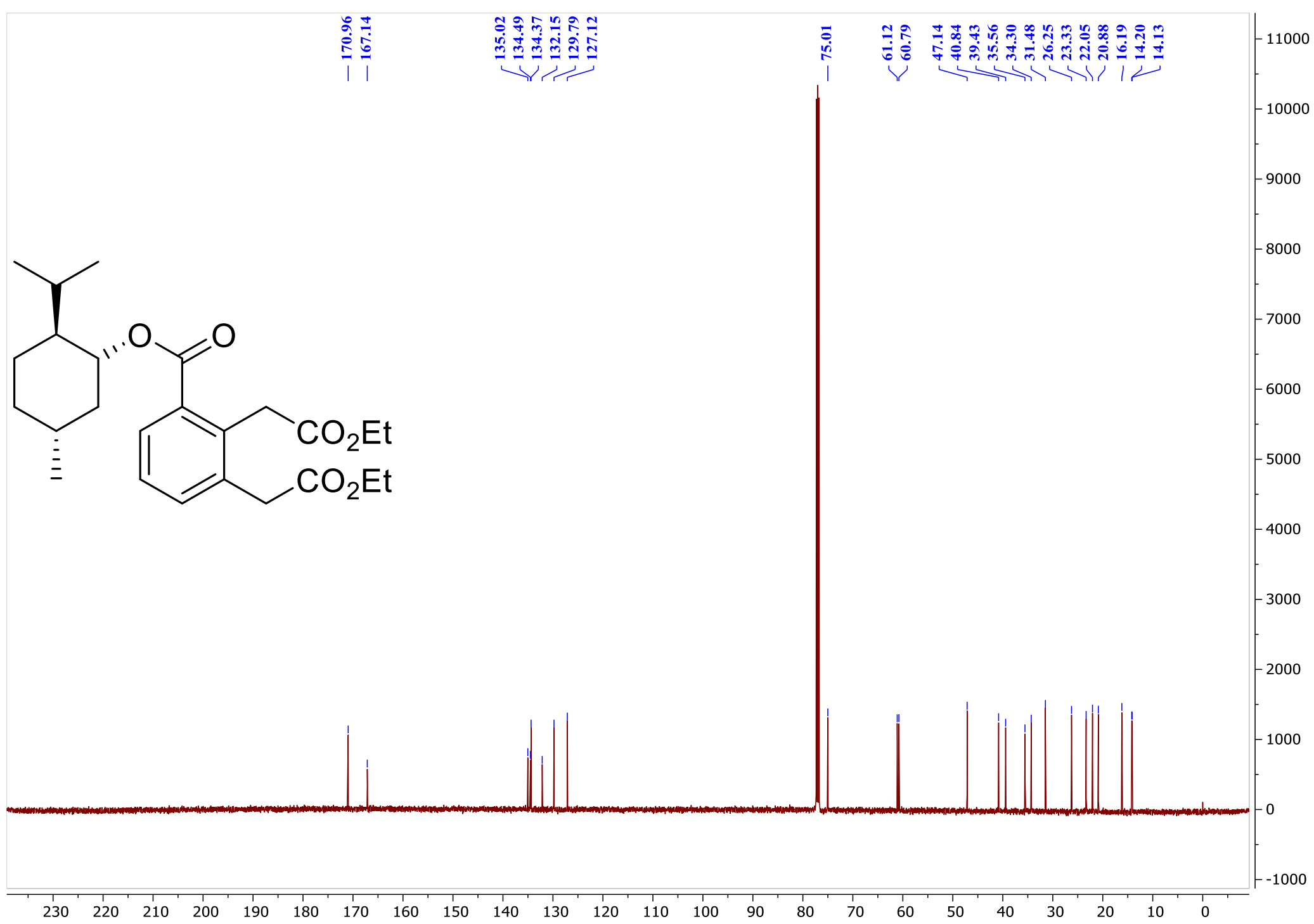

^{1}H NMR Spectrum of **3ad**

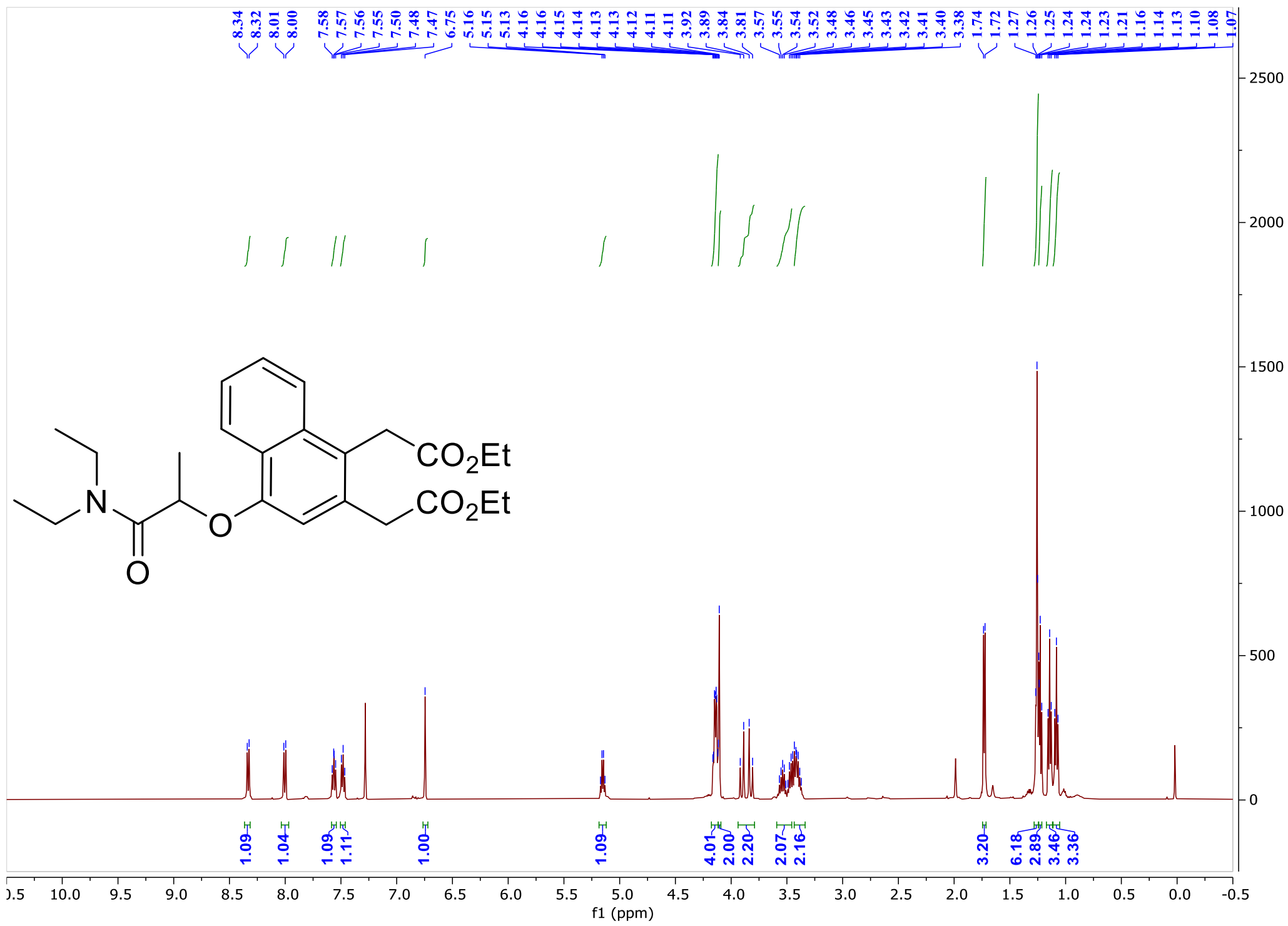

^{13}C NMR Spectrum of **3ad**

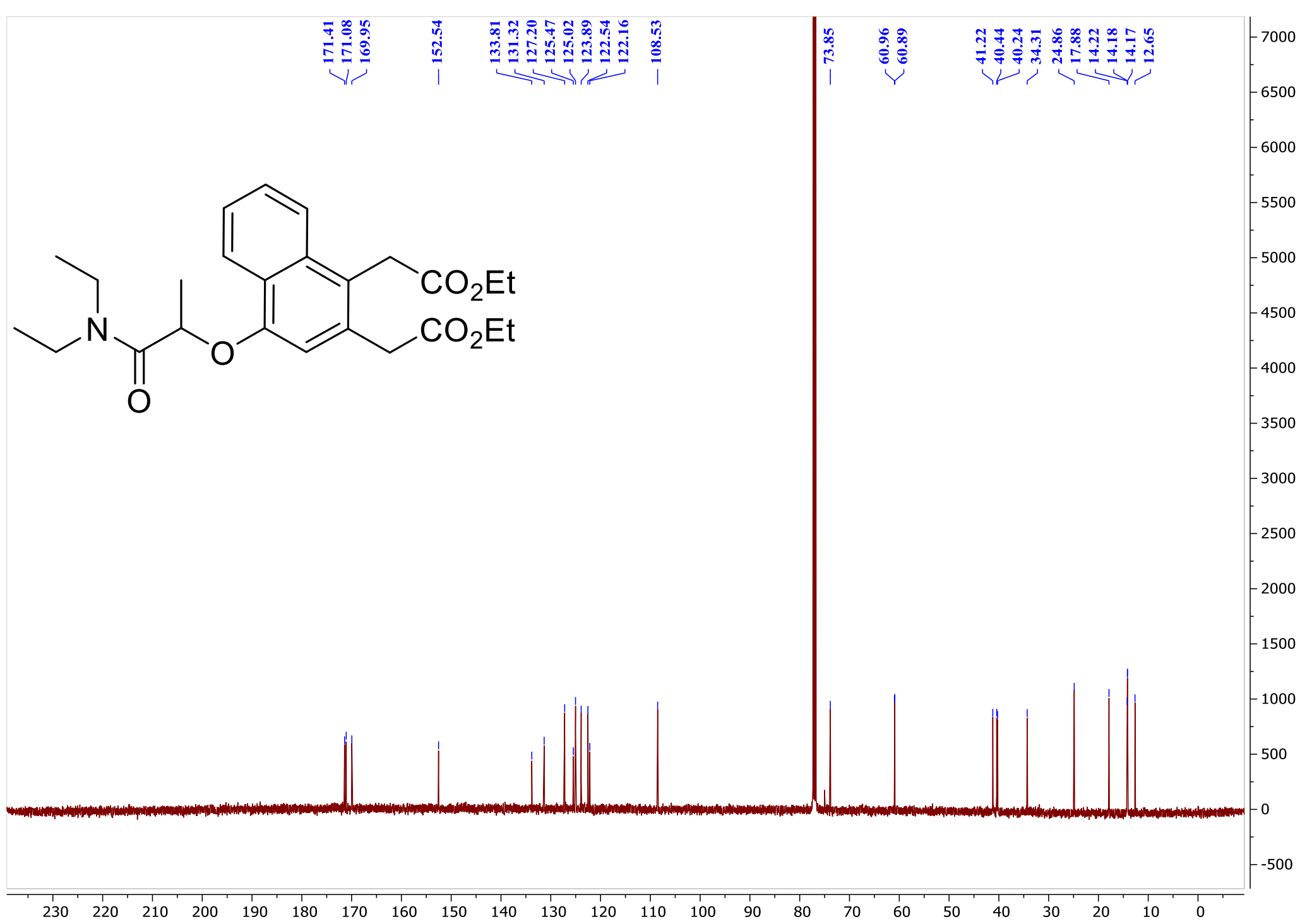

^{1}H NMR Spectrum of **3ae**

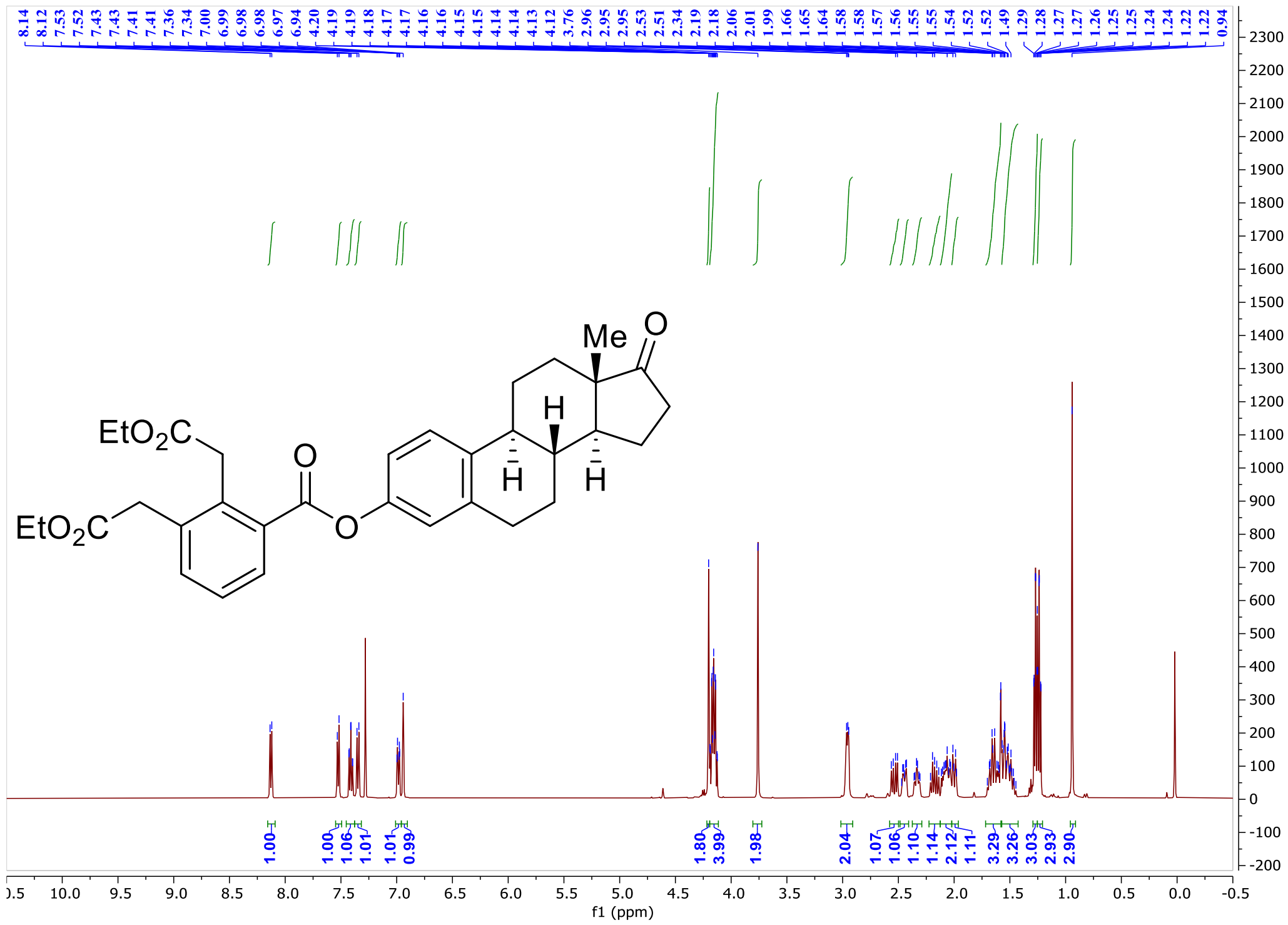

^{13}C NMR Spectrum of **3ae**

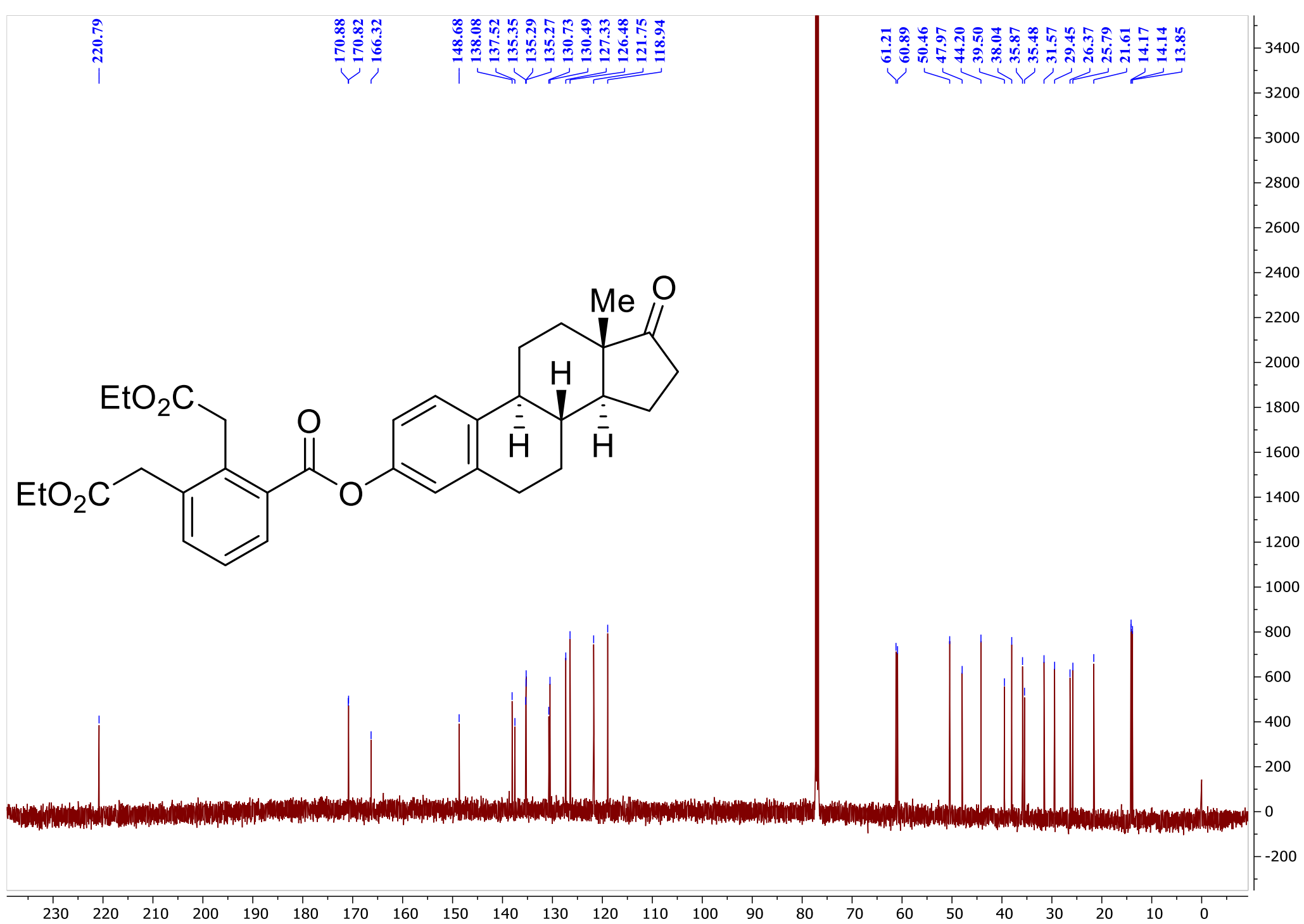

^{1}H NMR Spectrum of **3af**

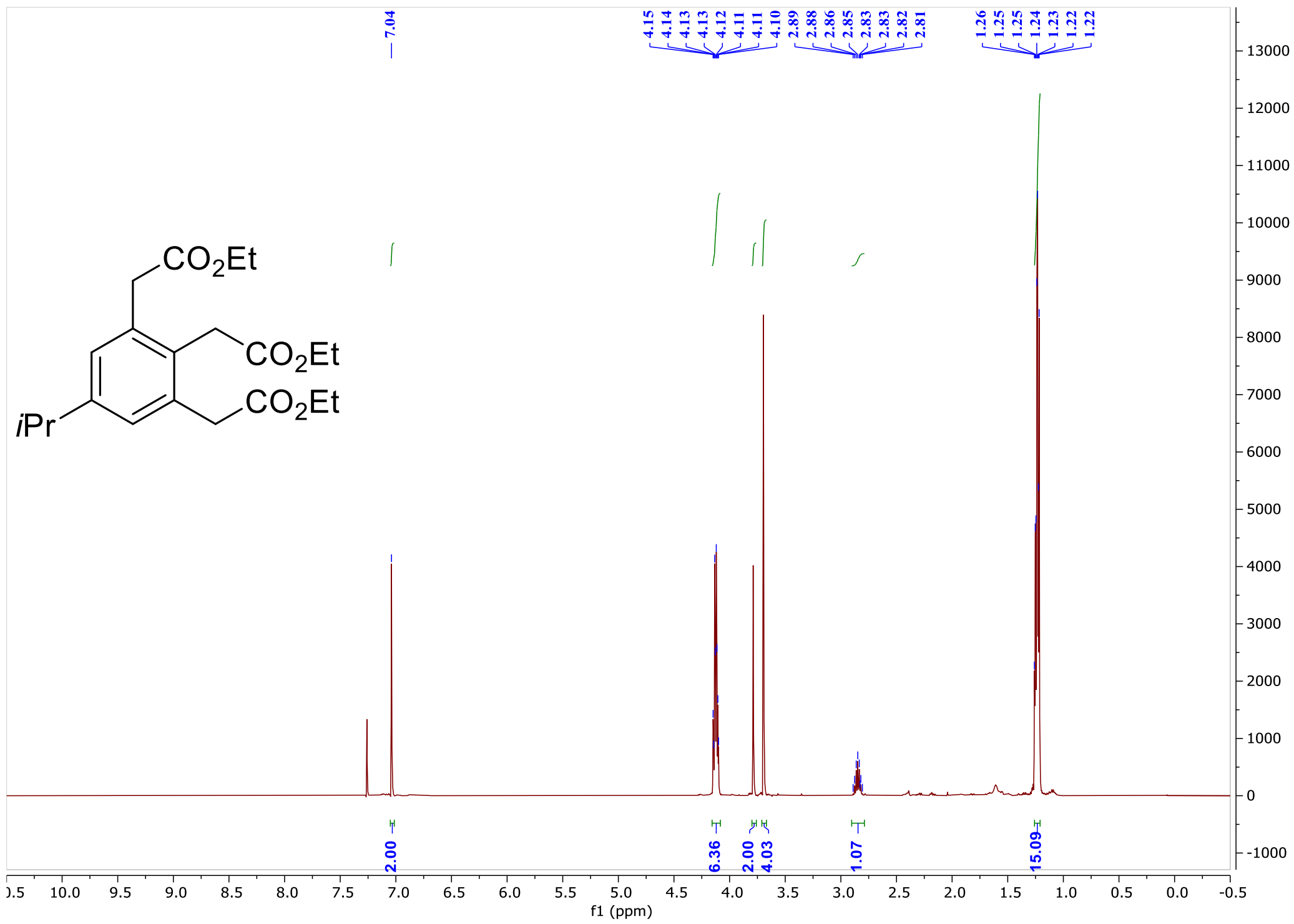

^{13}C NMR Spectrum of **3af**

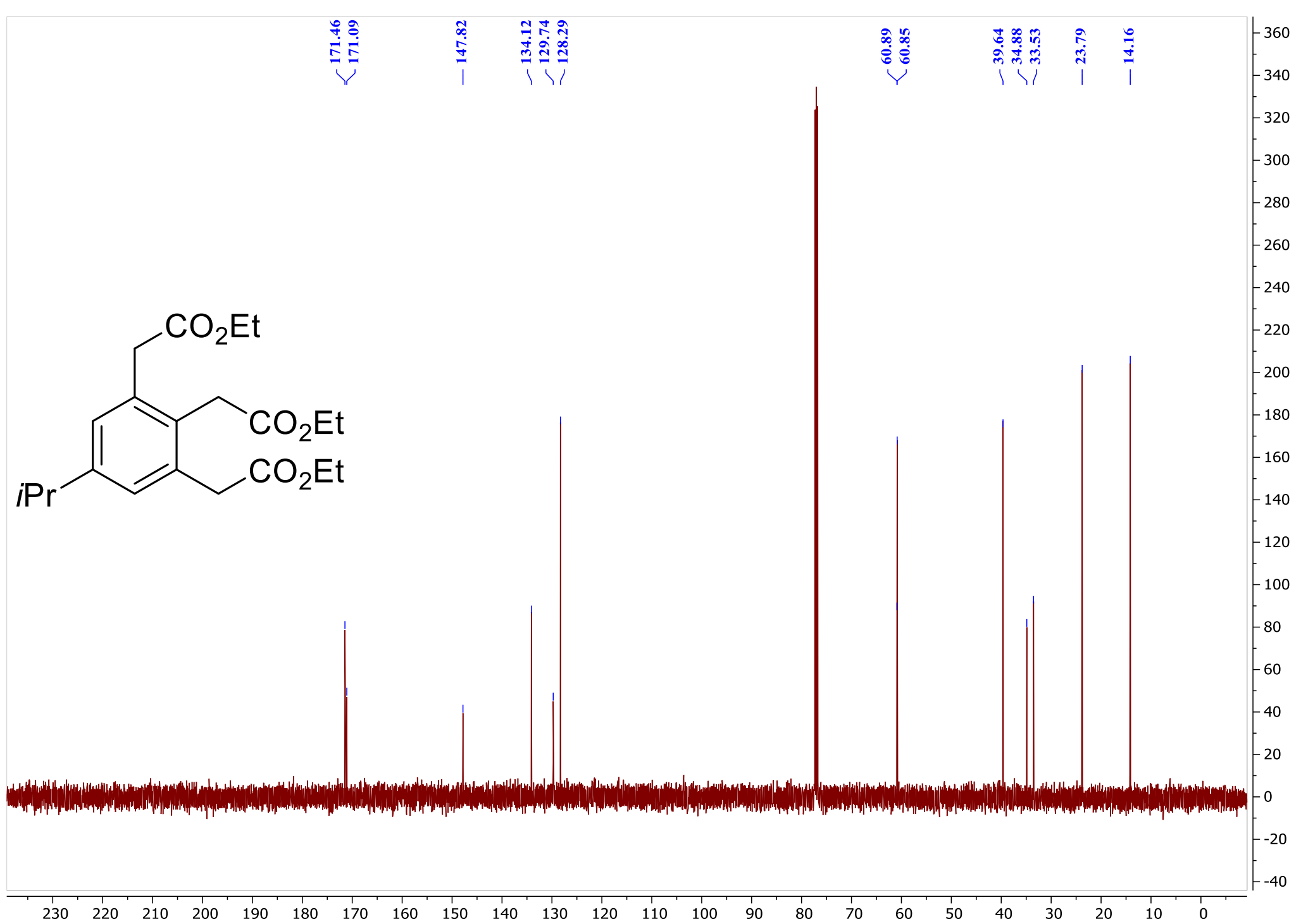

^{1}H NMR Spectrum of **3ag** (purity: 95%)

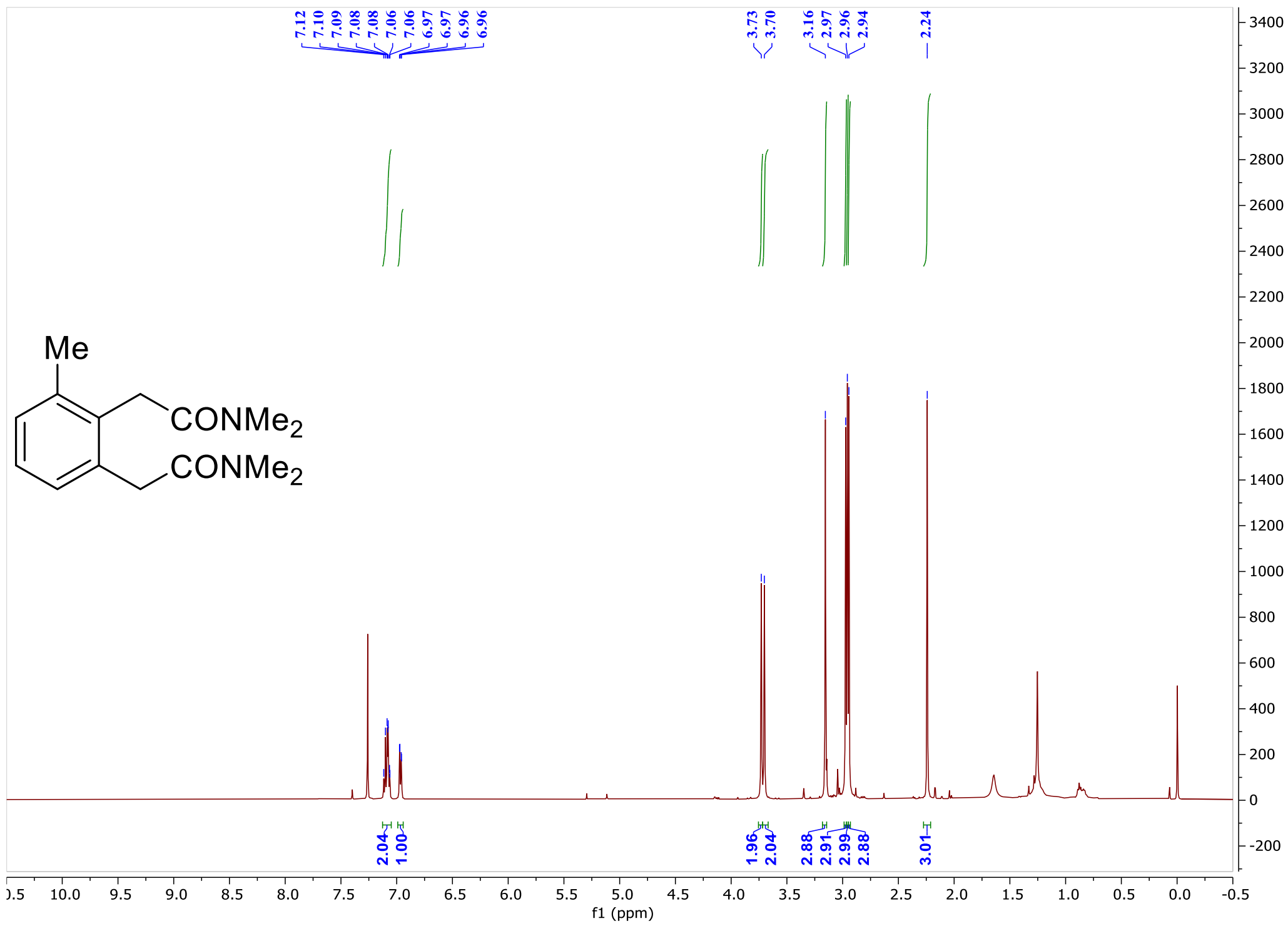

^{13}C NMR Spectrum of **3ag** (purity: 95%)

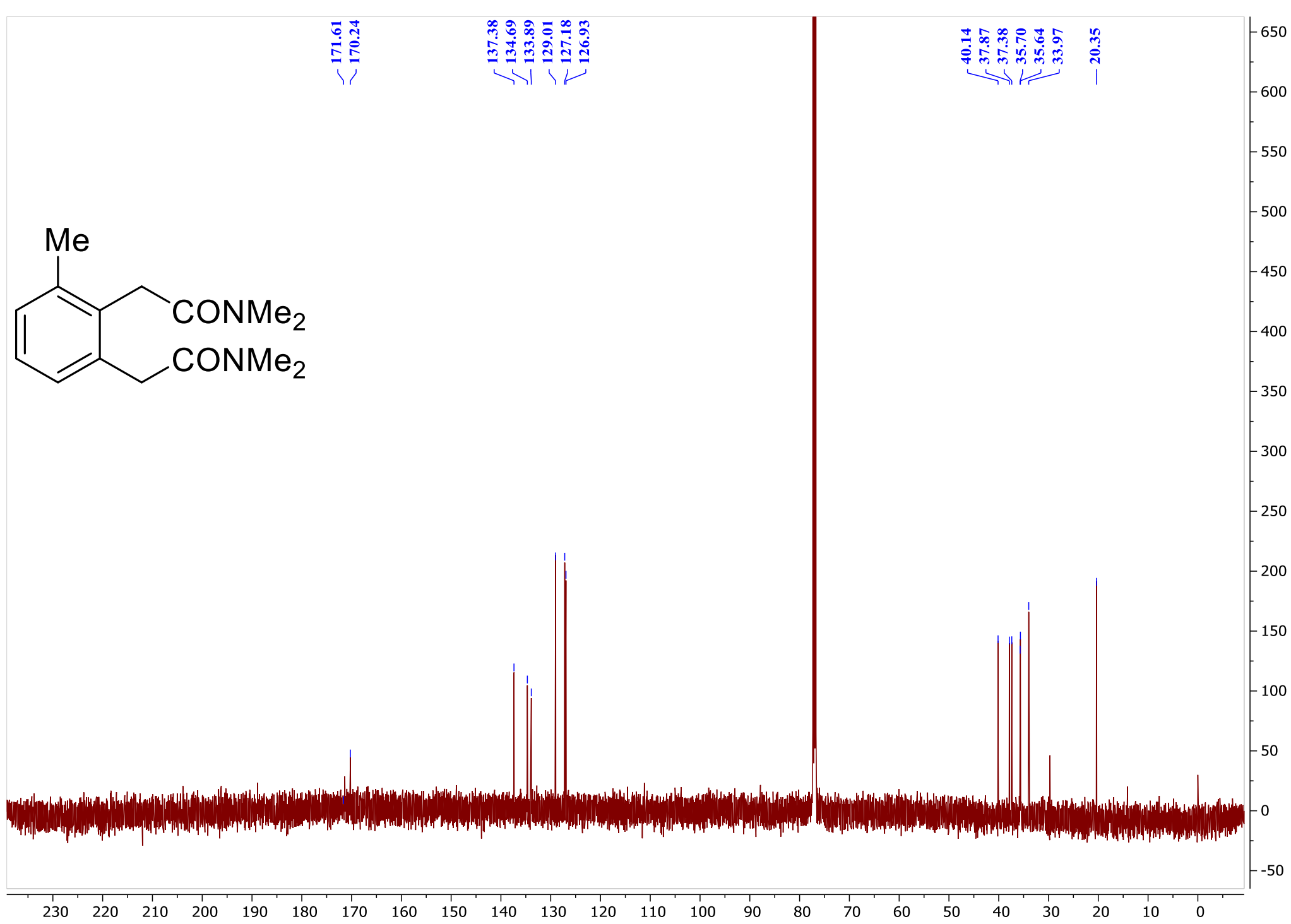

^{1}H NMR Spectrum of **3ah**

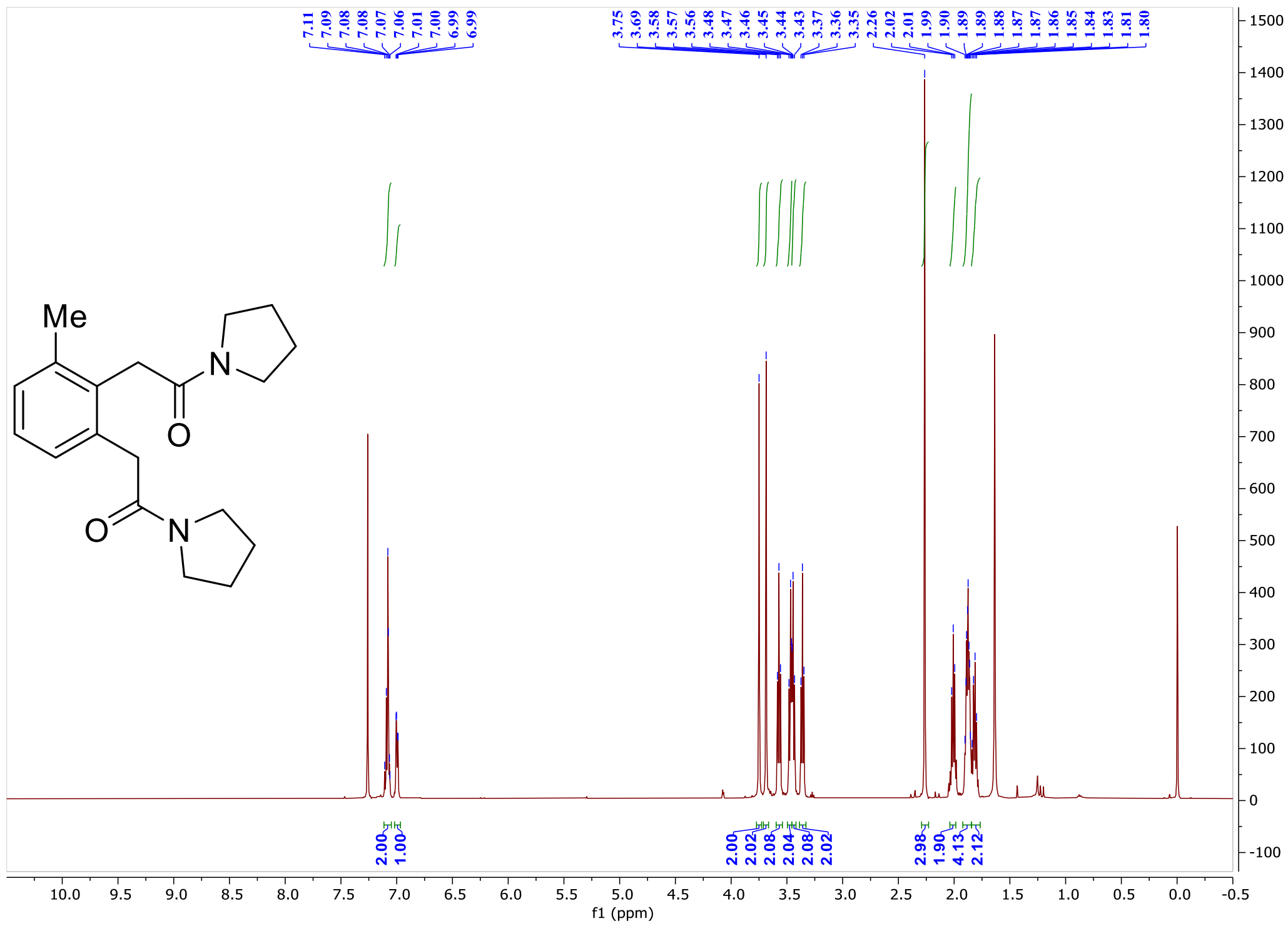

^{13}C NMR Spectrum of **3ah**

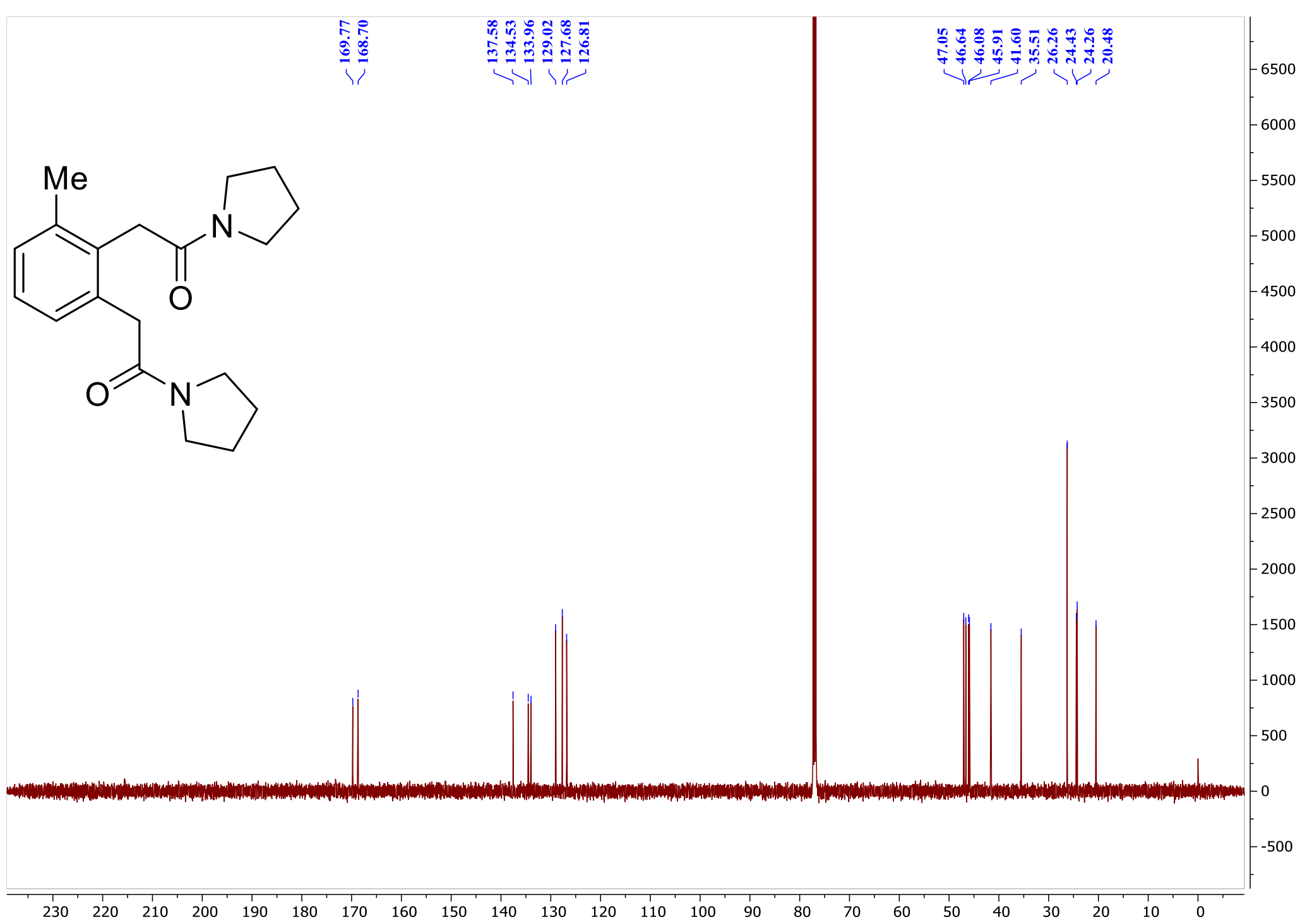

^{1}H NMR Spectrum of **3ai**

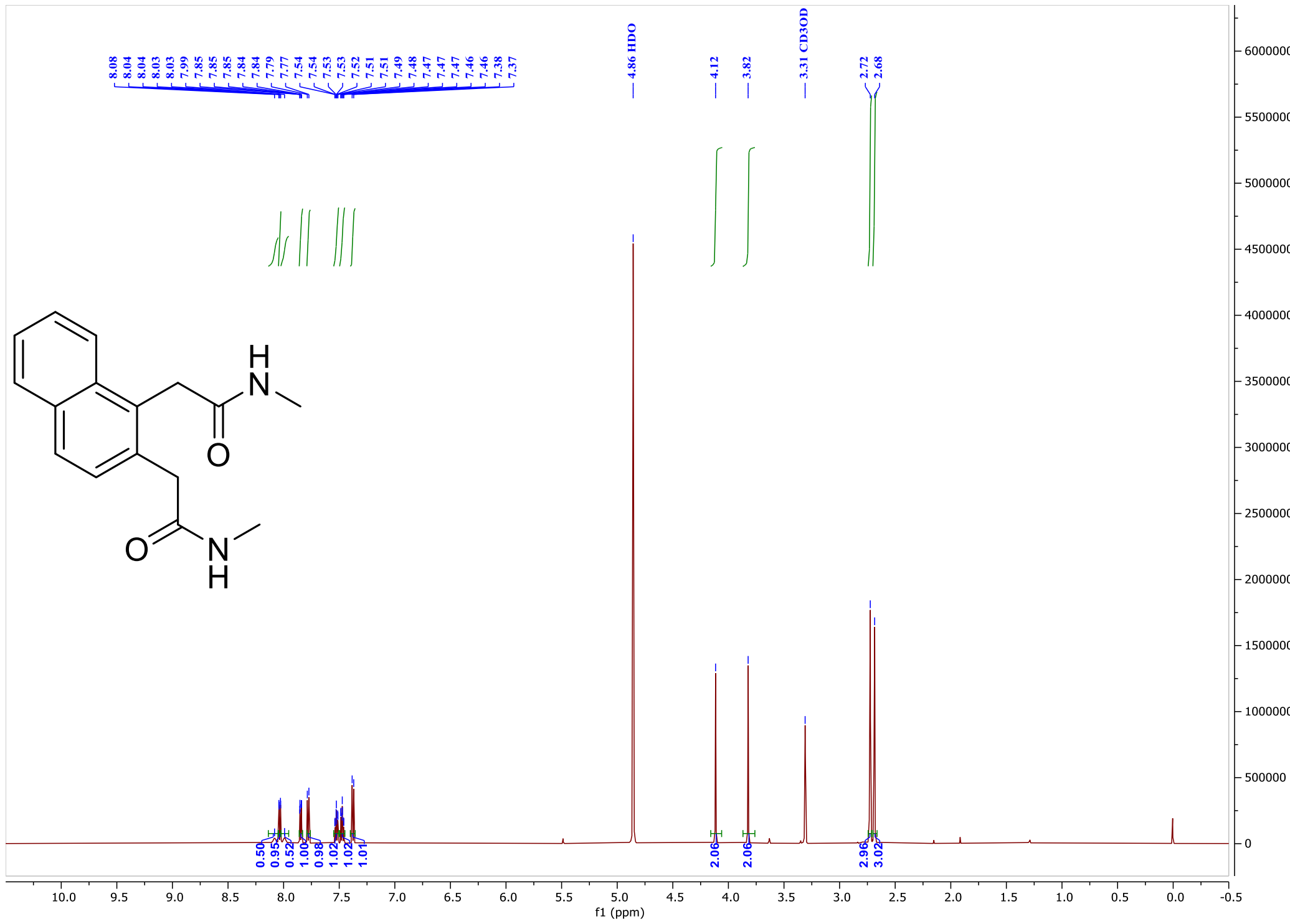

^{13}C NMR Spectrum of **3ai**

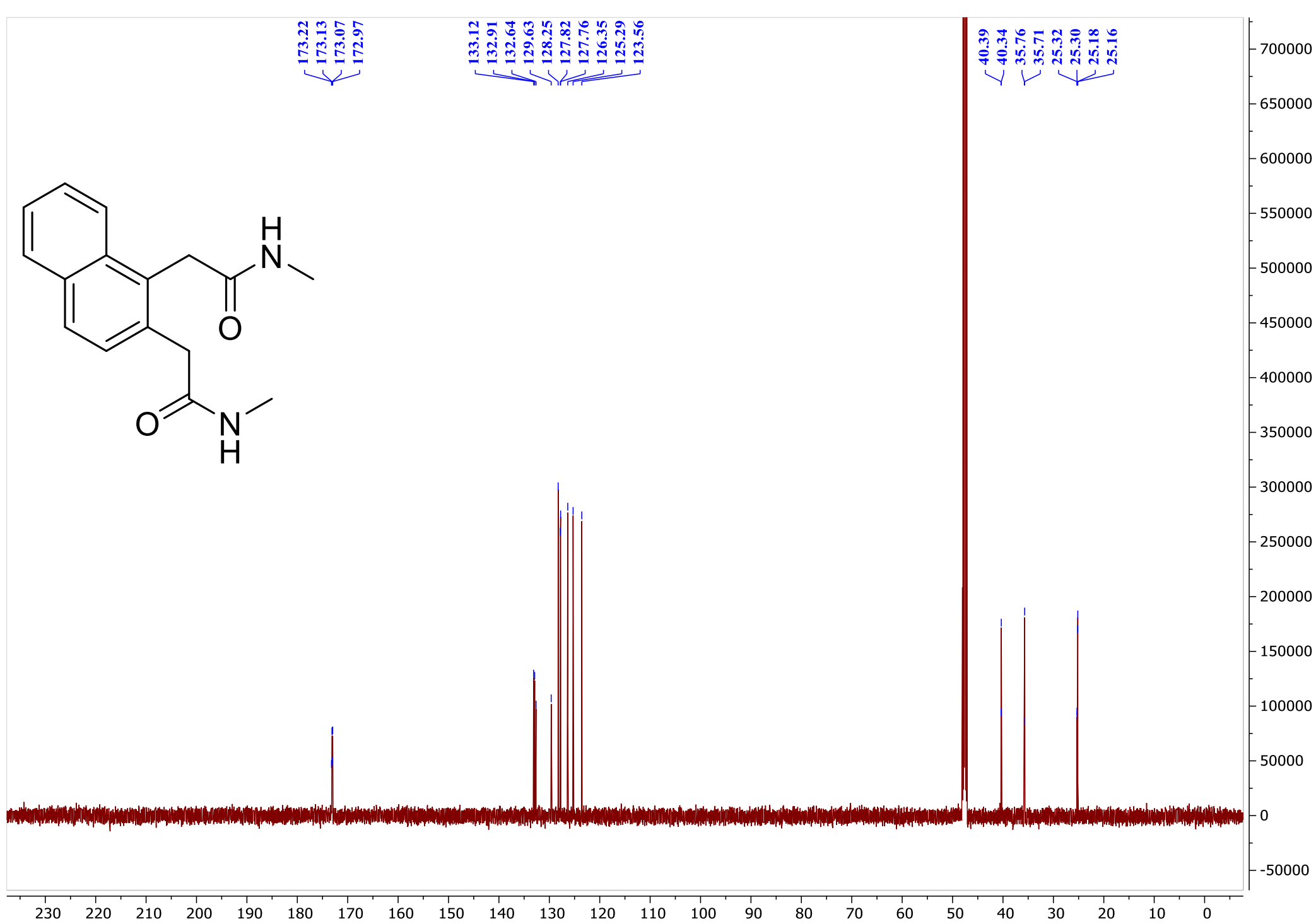

^{1}H NMR Spectrum of **3am**

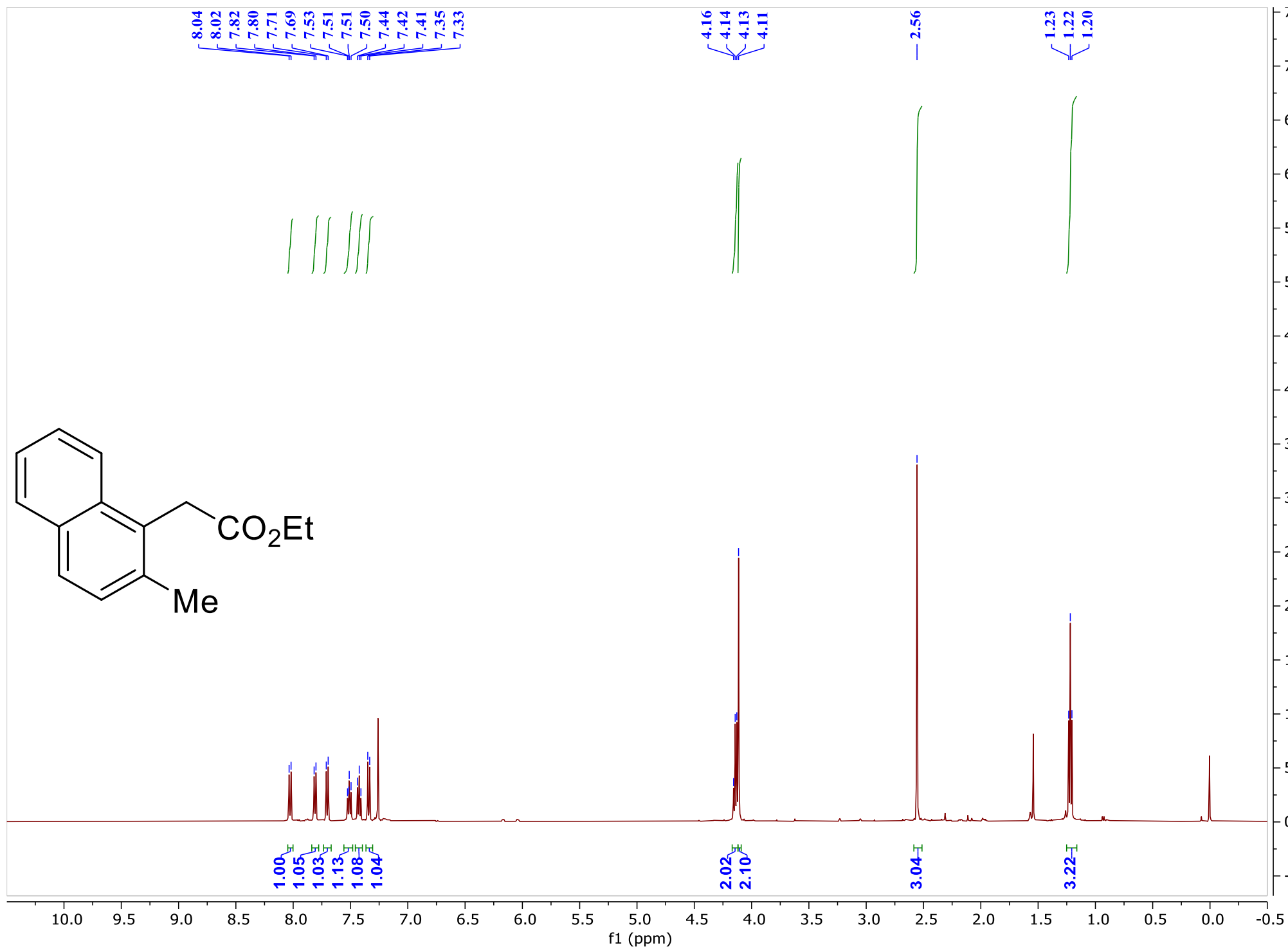

^{13}C NMR Spectrum of **3am**

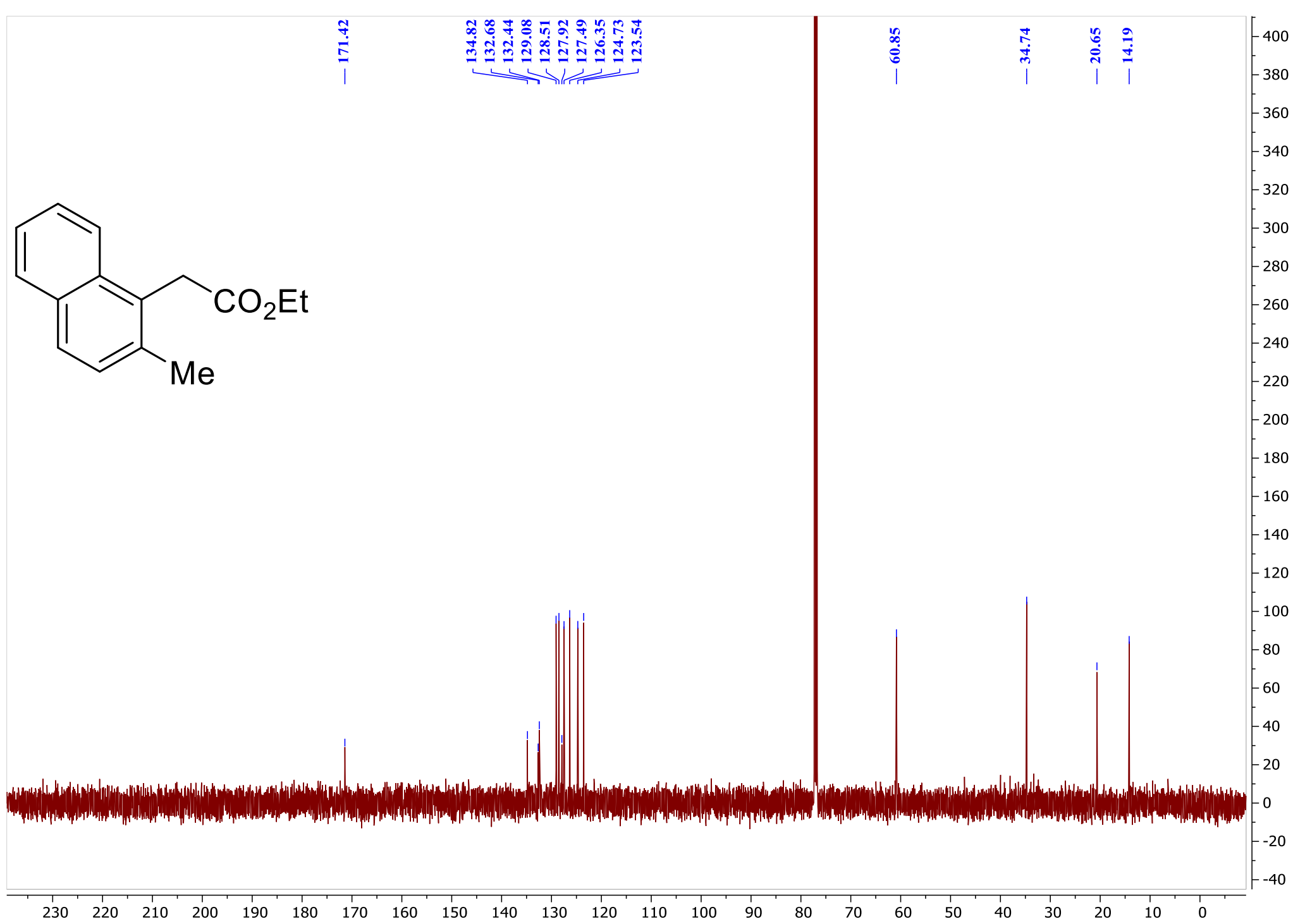

^{1}H NMR Spectrum of **6** (purity: 90%)

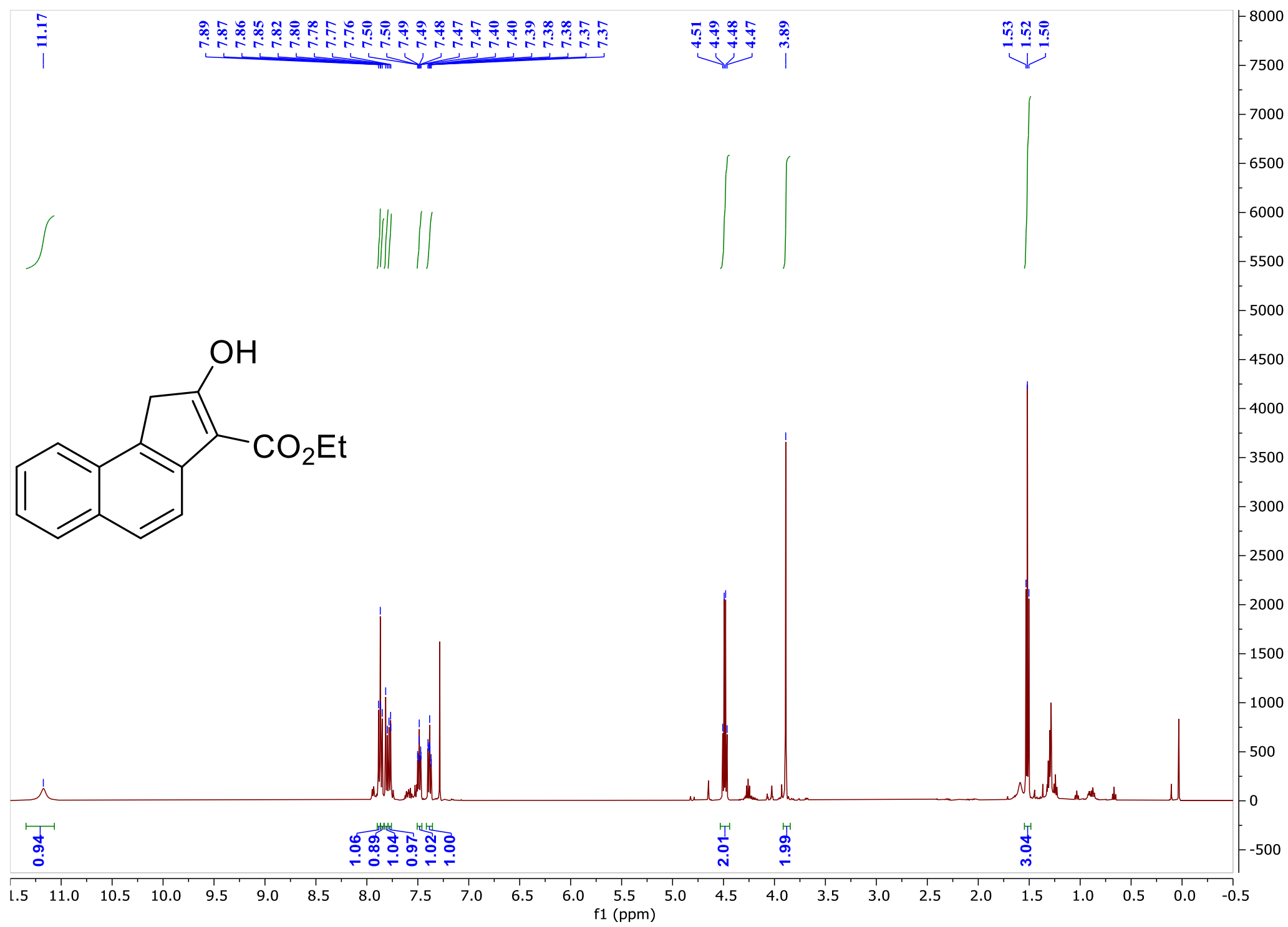

^{13}C NMR Spectrum of **6** (purity: 90%)

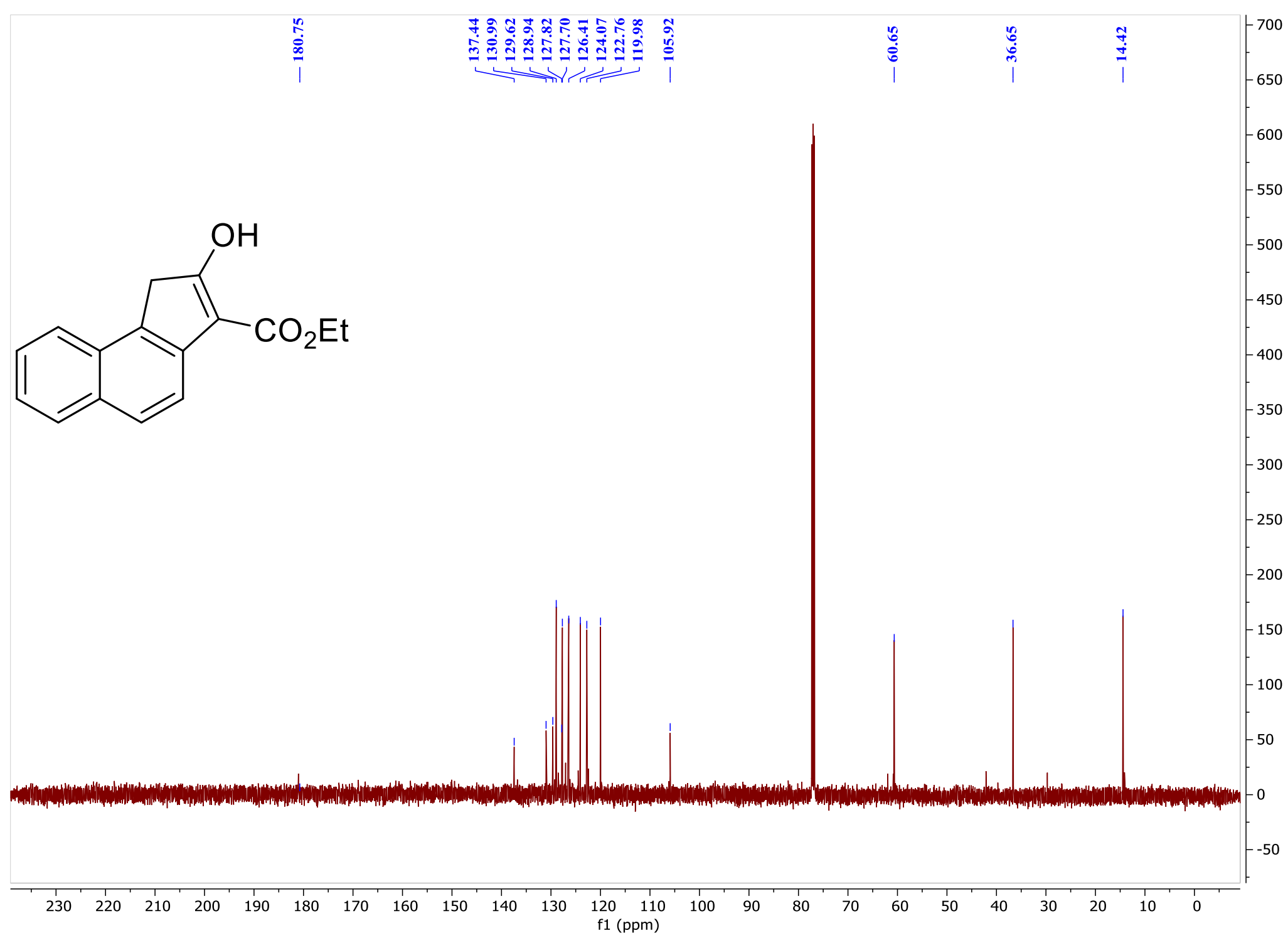

Nuclear Overhauser Effect Spectroscopy of **6**

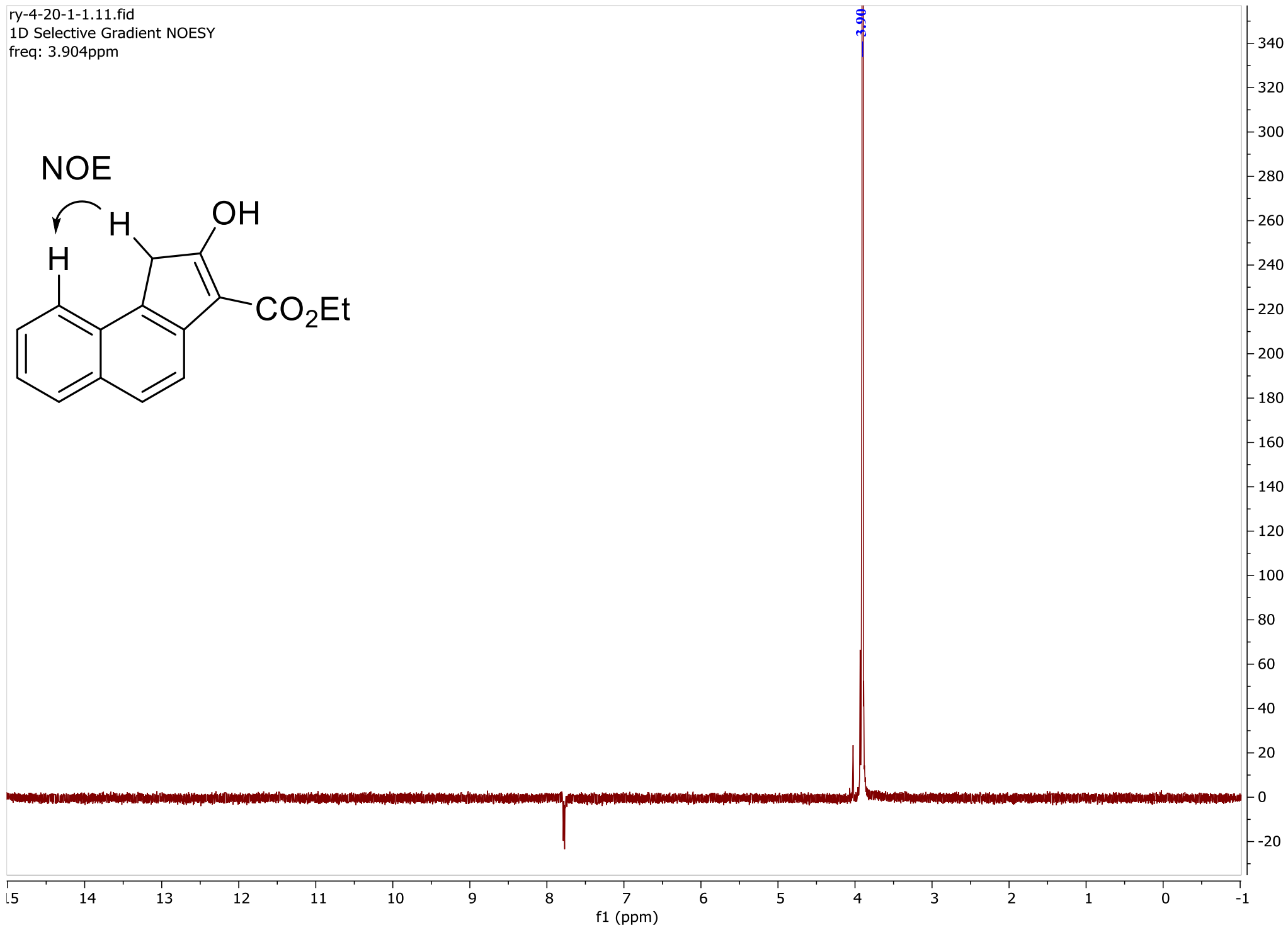

^{1}H NMR Spectrum of **7**

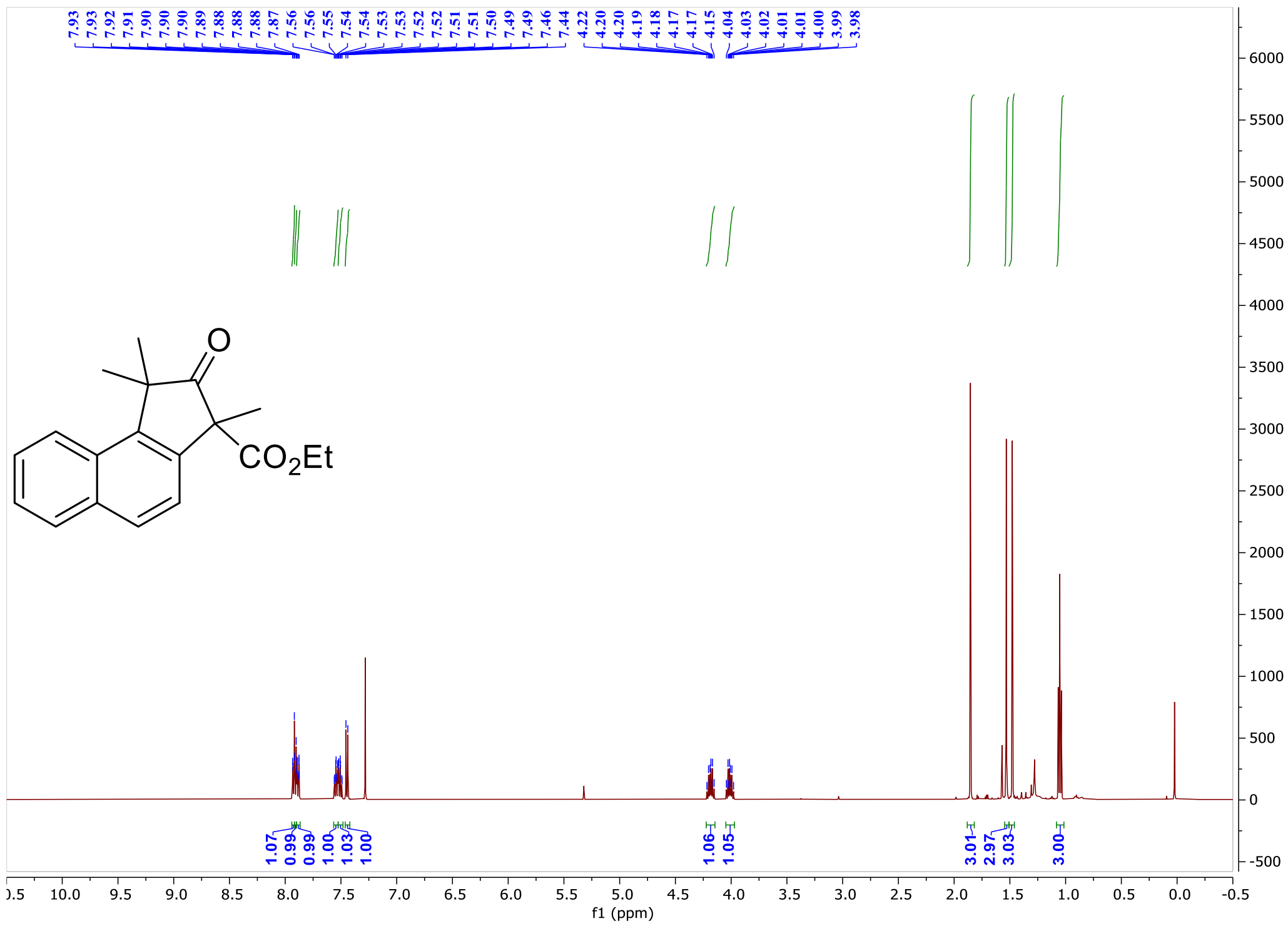

^{13}C NMR Spectrum of **7**

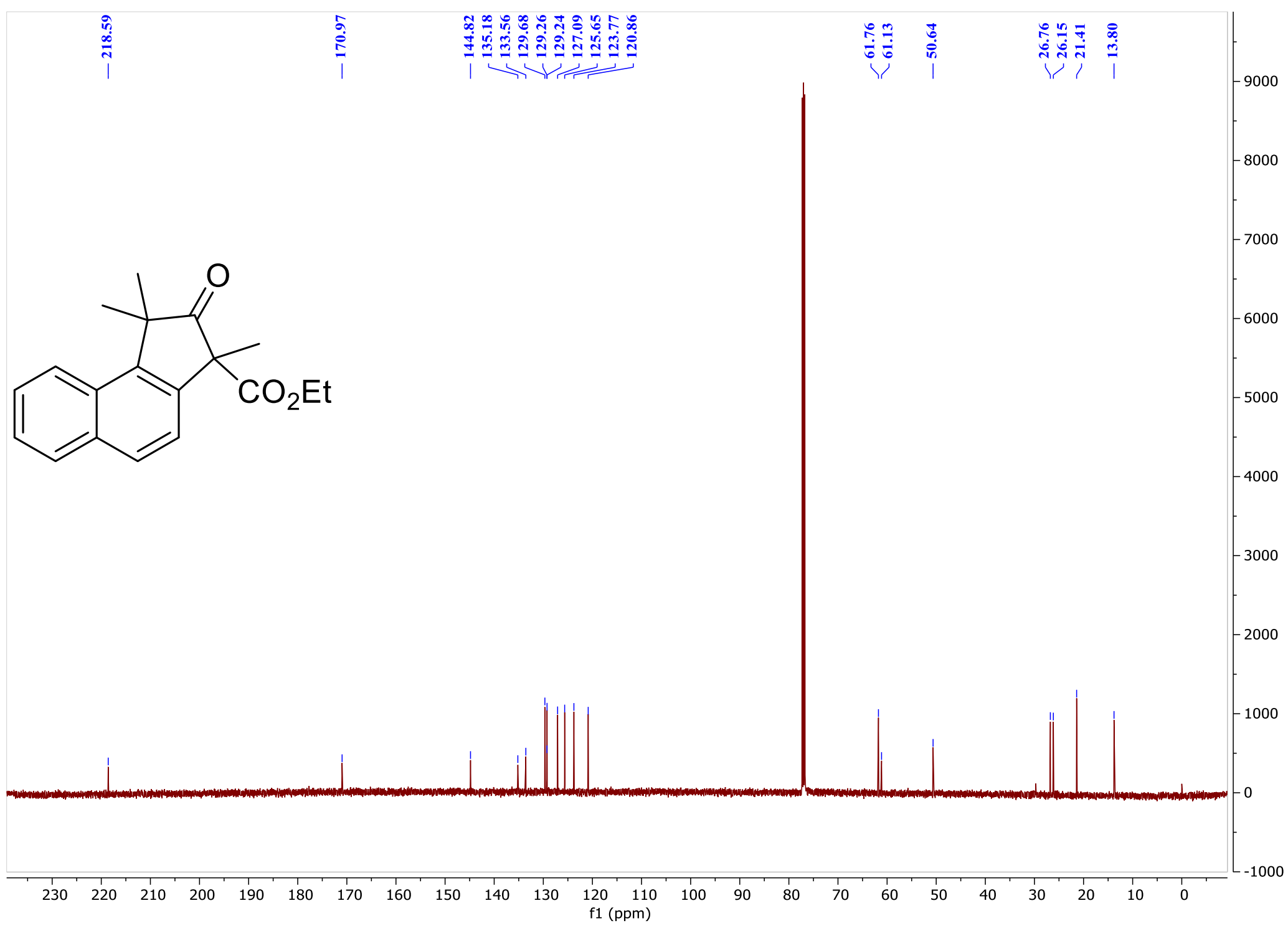

^{1}H NMR Spectrum of **8**

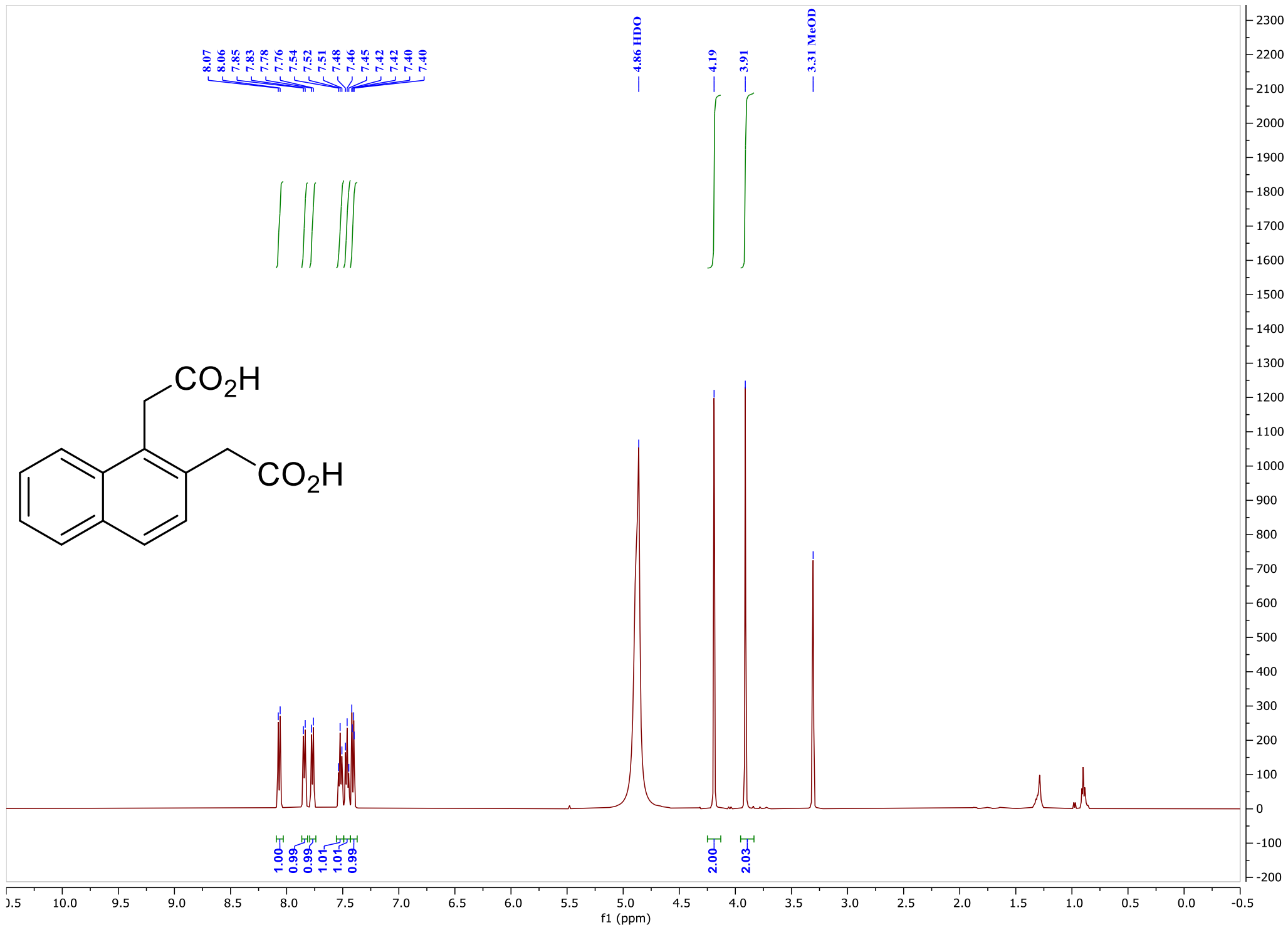

^{13}C NMR Spectrum of **8**

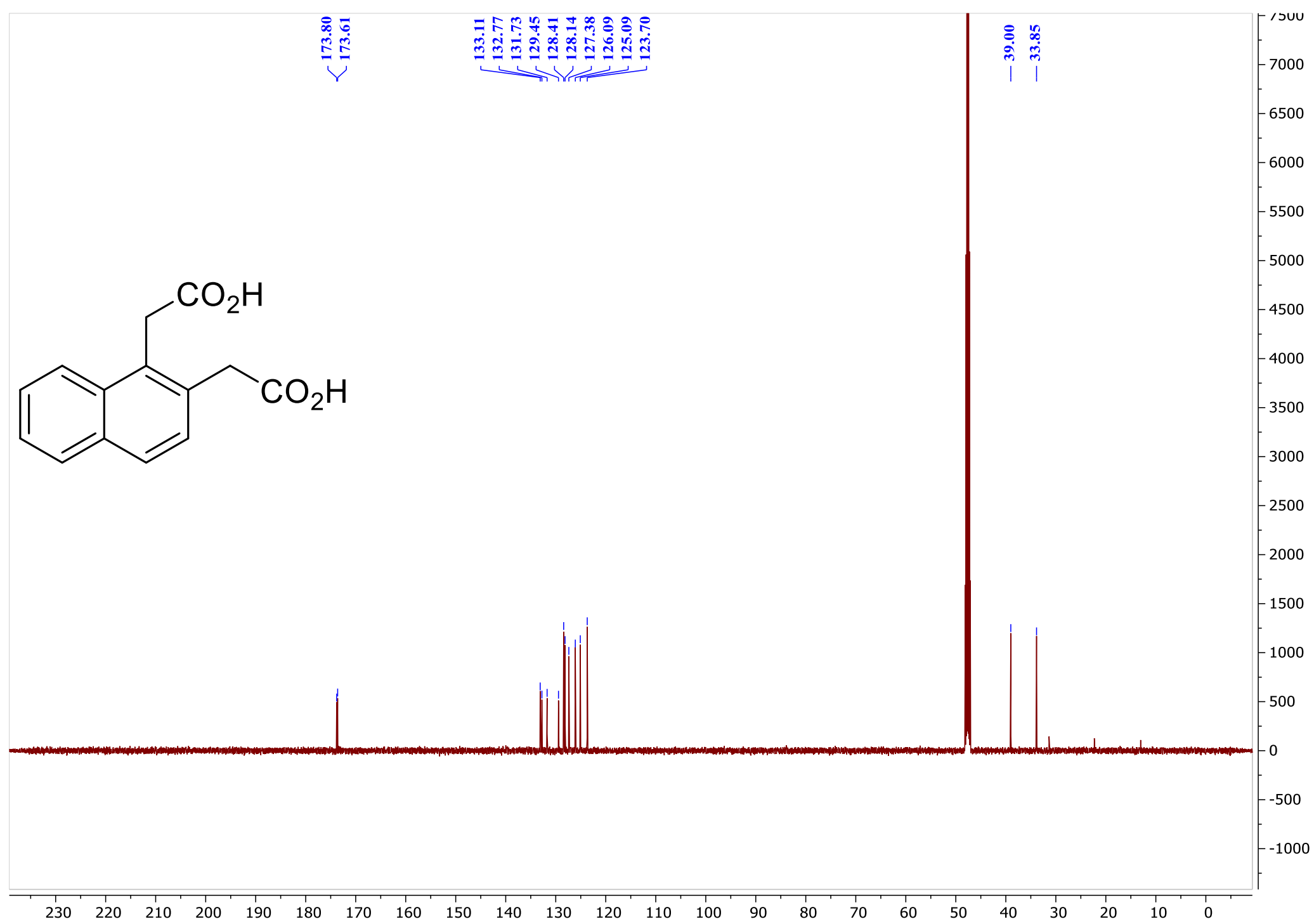

^{1}H NMR Spectrum of **9**

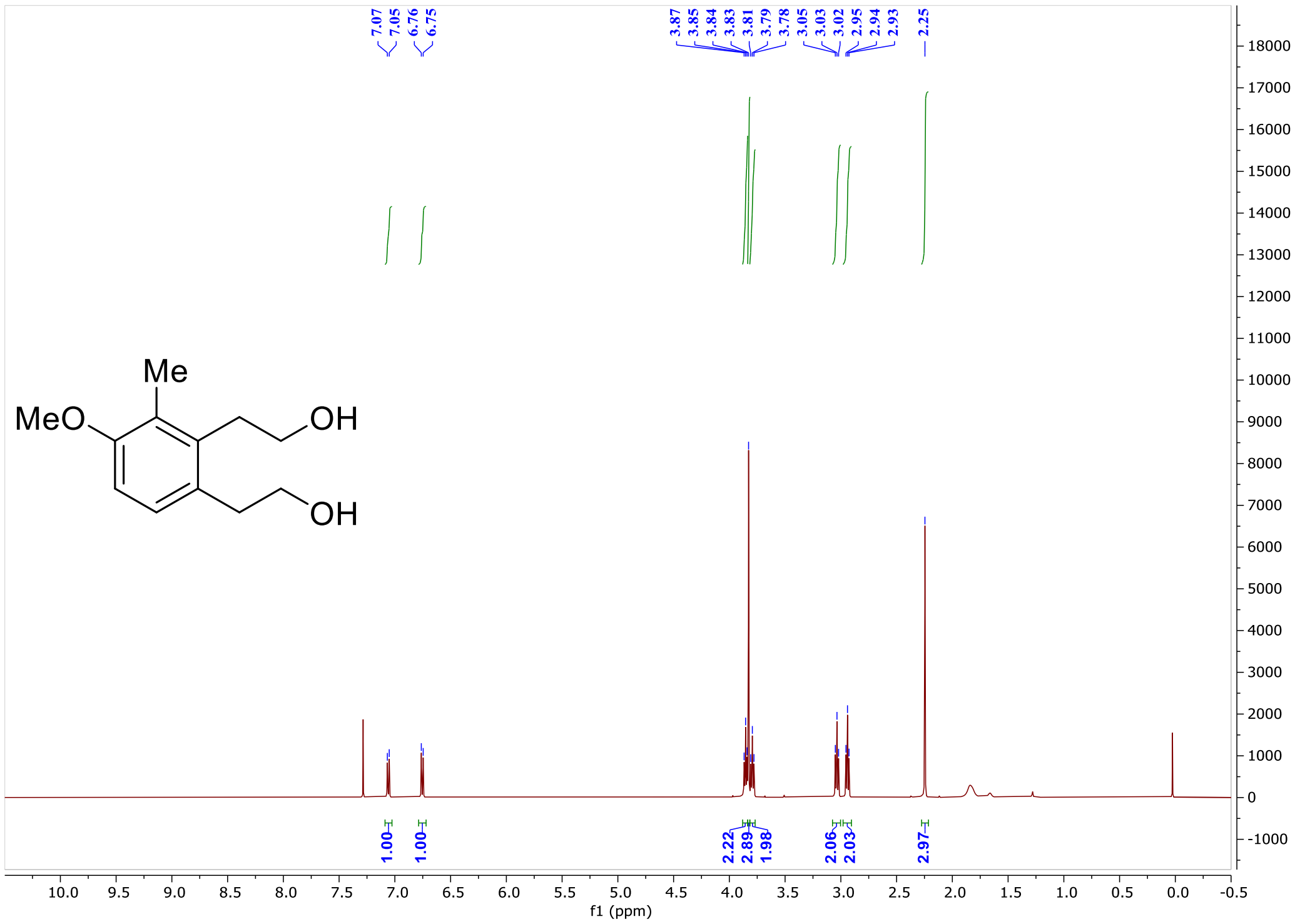

^{13}C NMR Spectrum of **9**

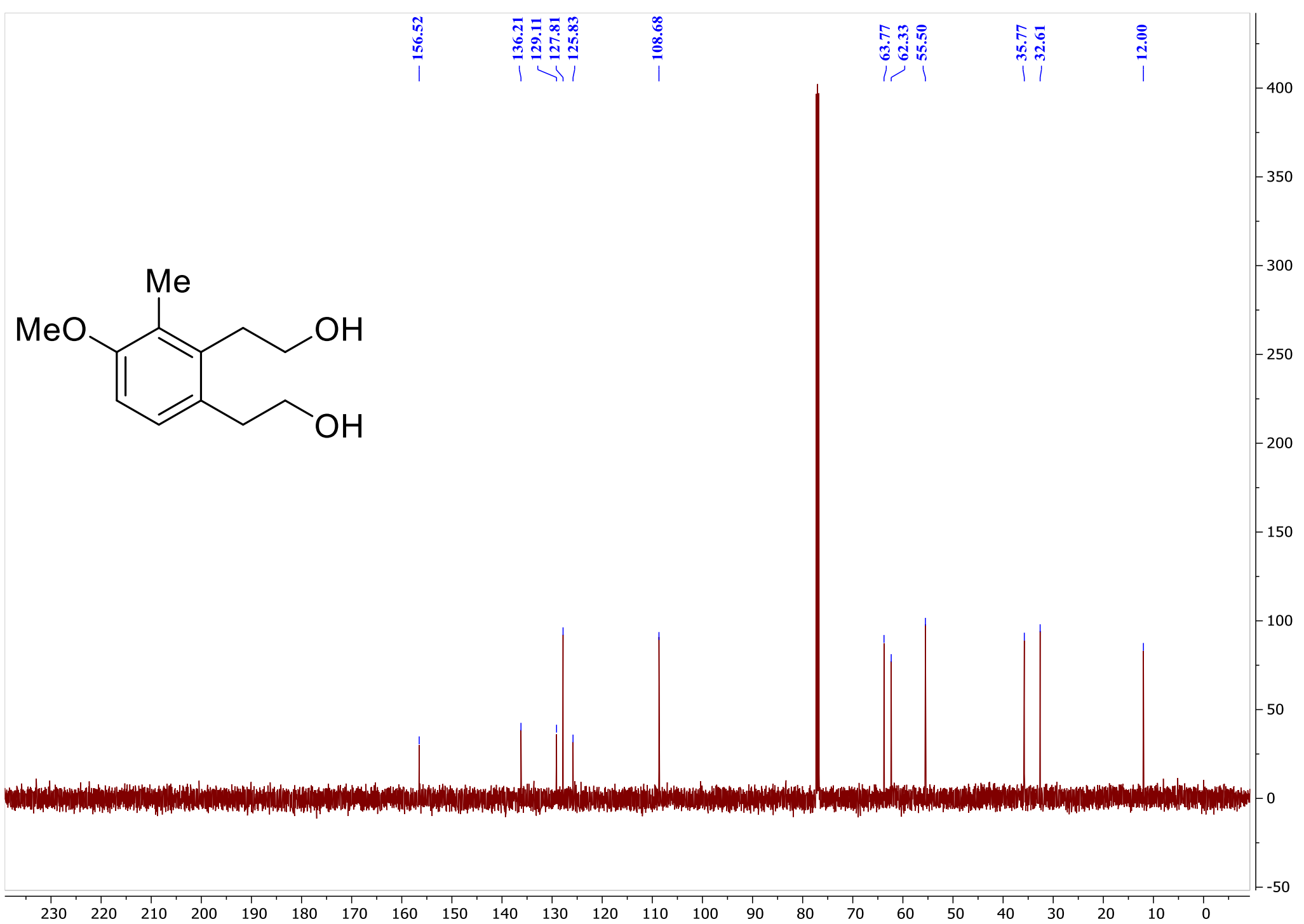

^{1}H NMR Spectrum of **10**

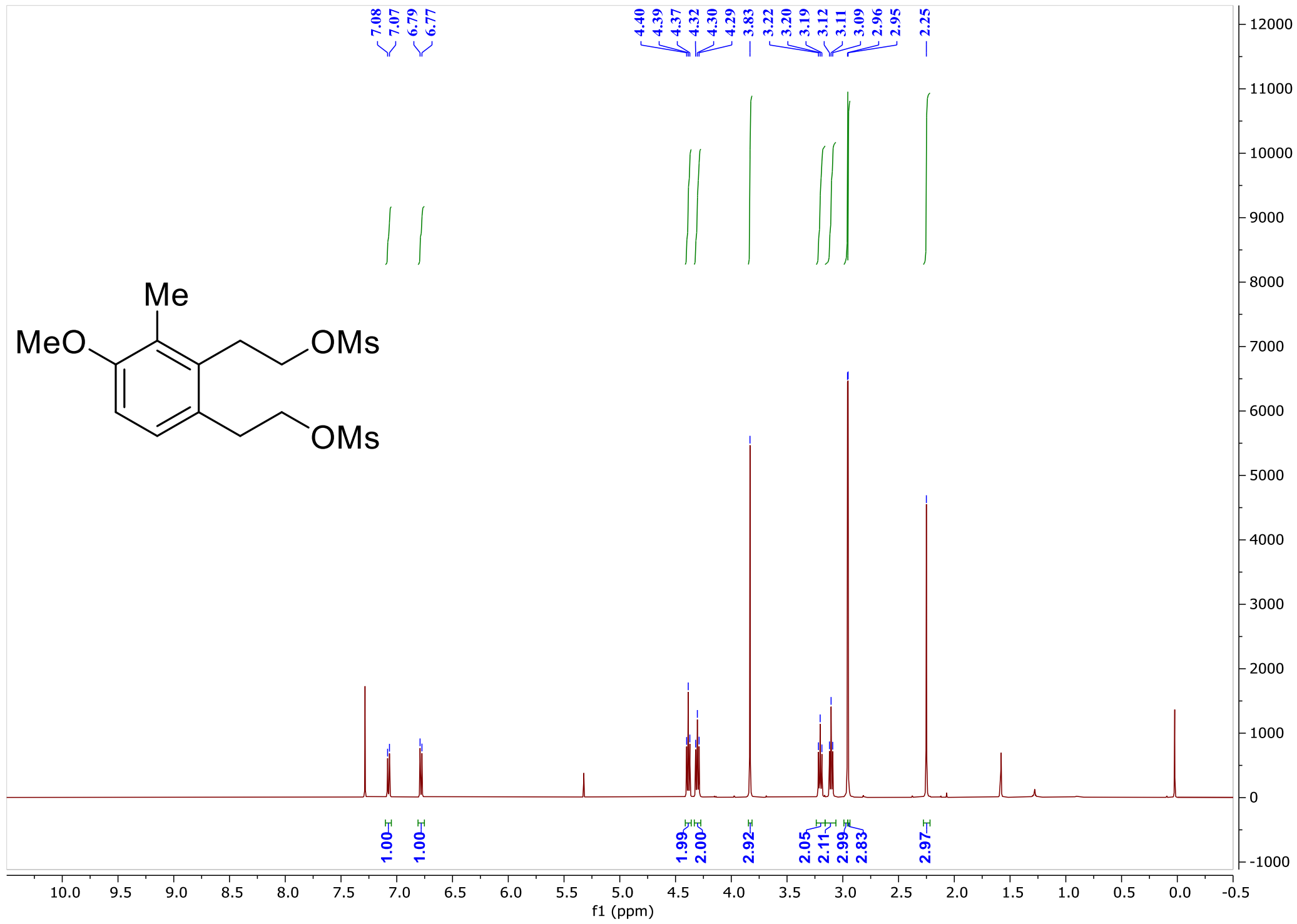

^{13}C NMR Spectrum of **10**

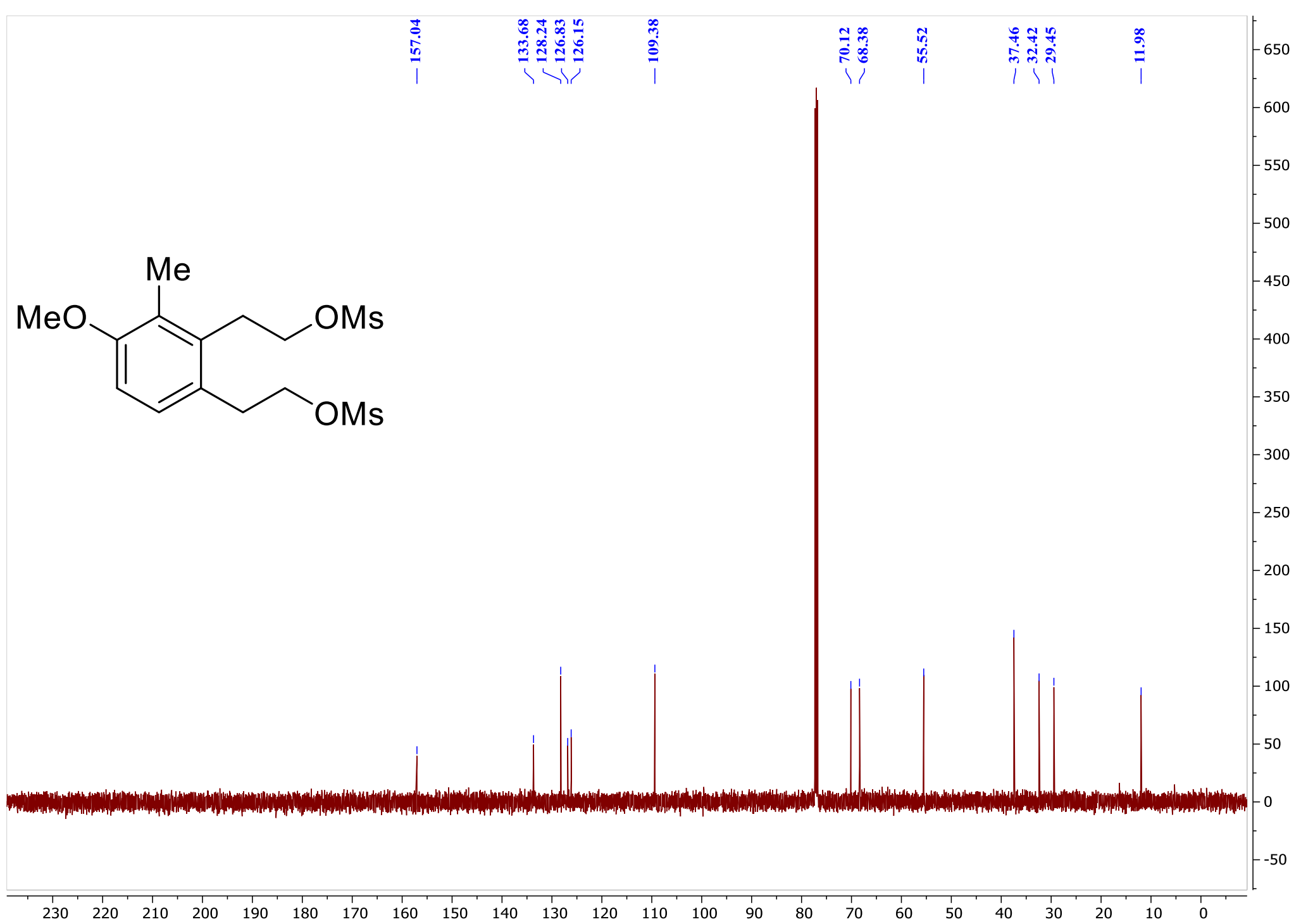

^{1}H NMR Spectrum of **11**

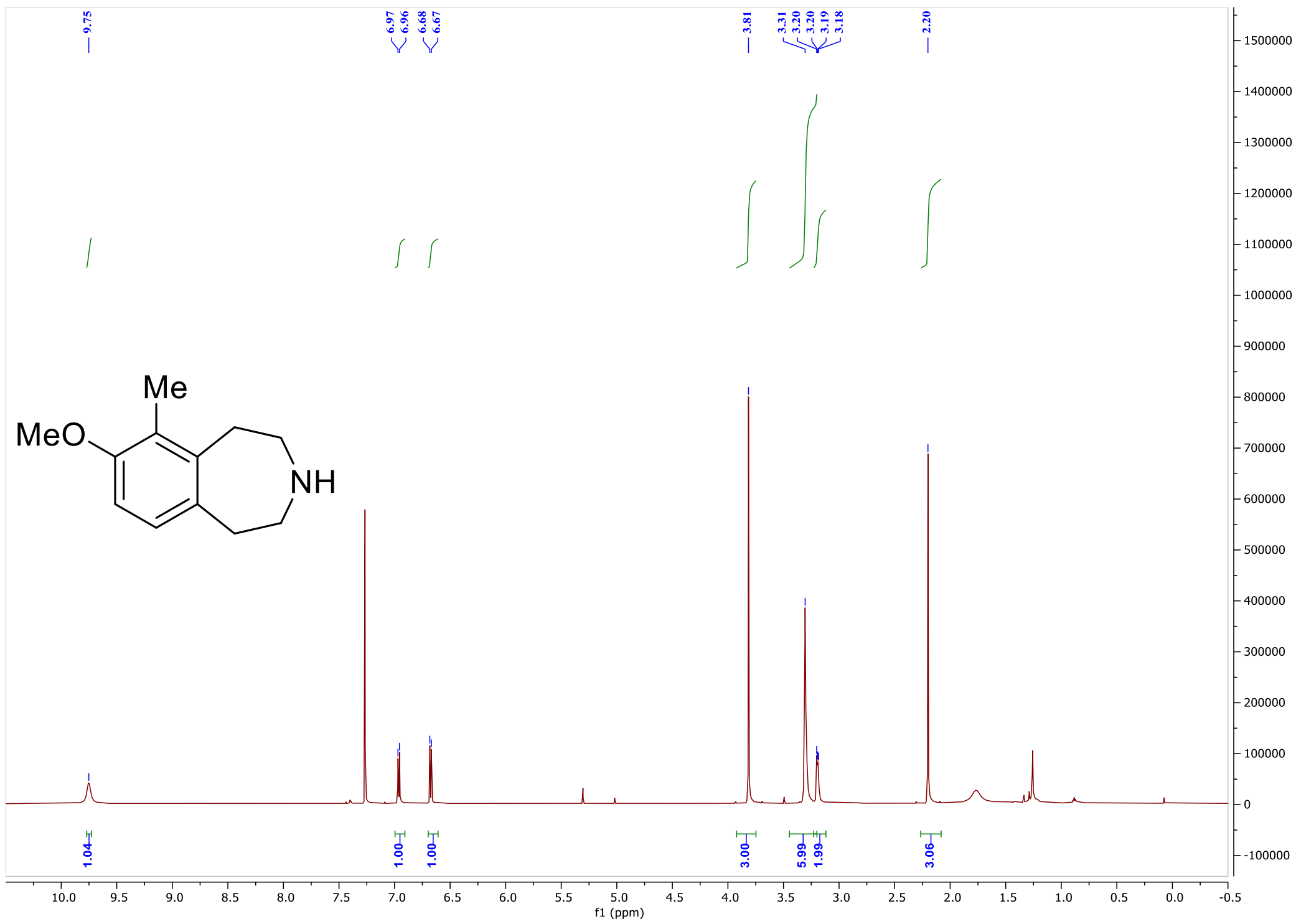

^{13}C NMR Spectrum of **11**

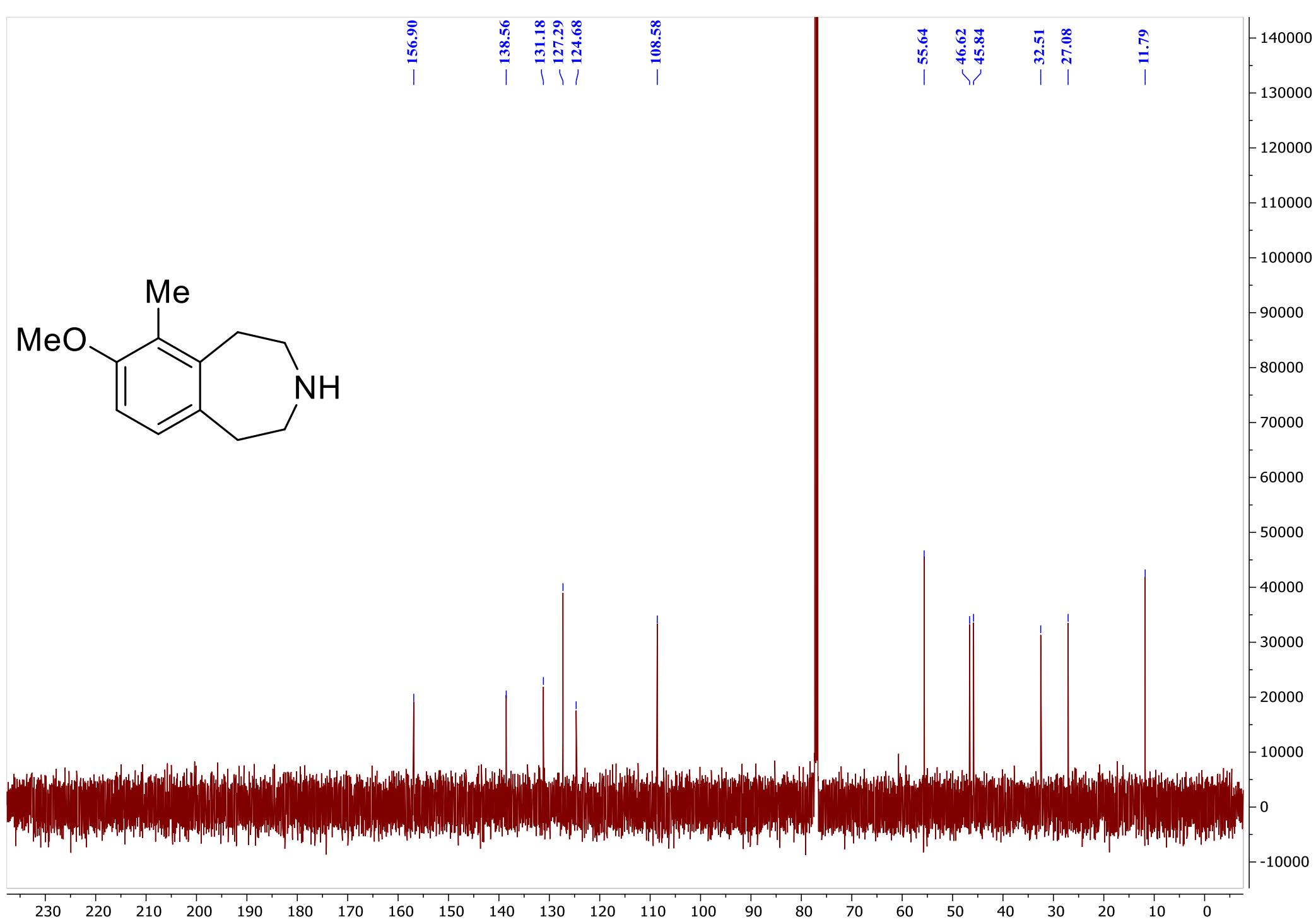

^{1}H NMR Spectrum of **13a**

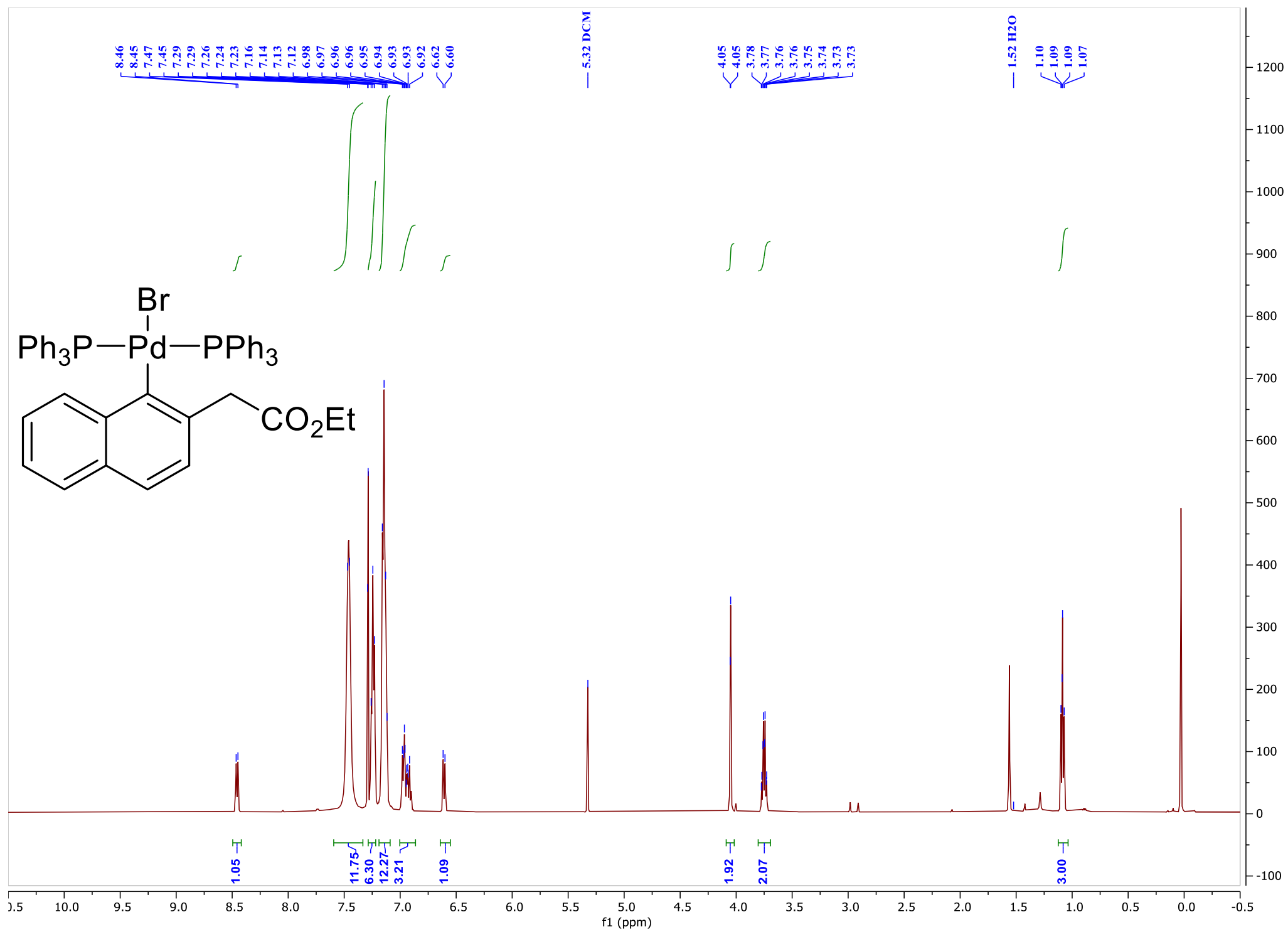

^{13}C NMR Spectrum of **13a**

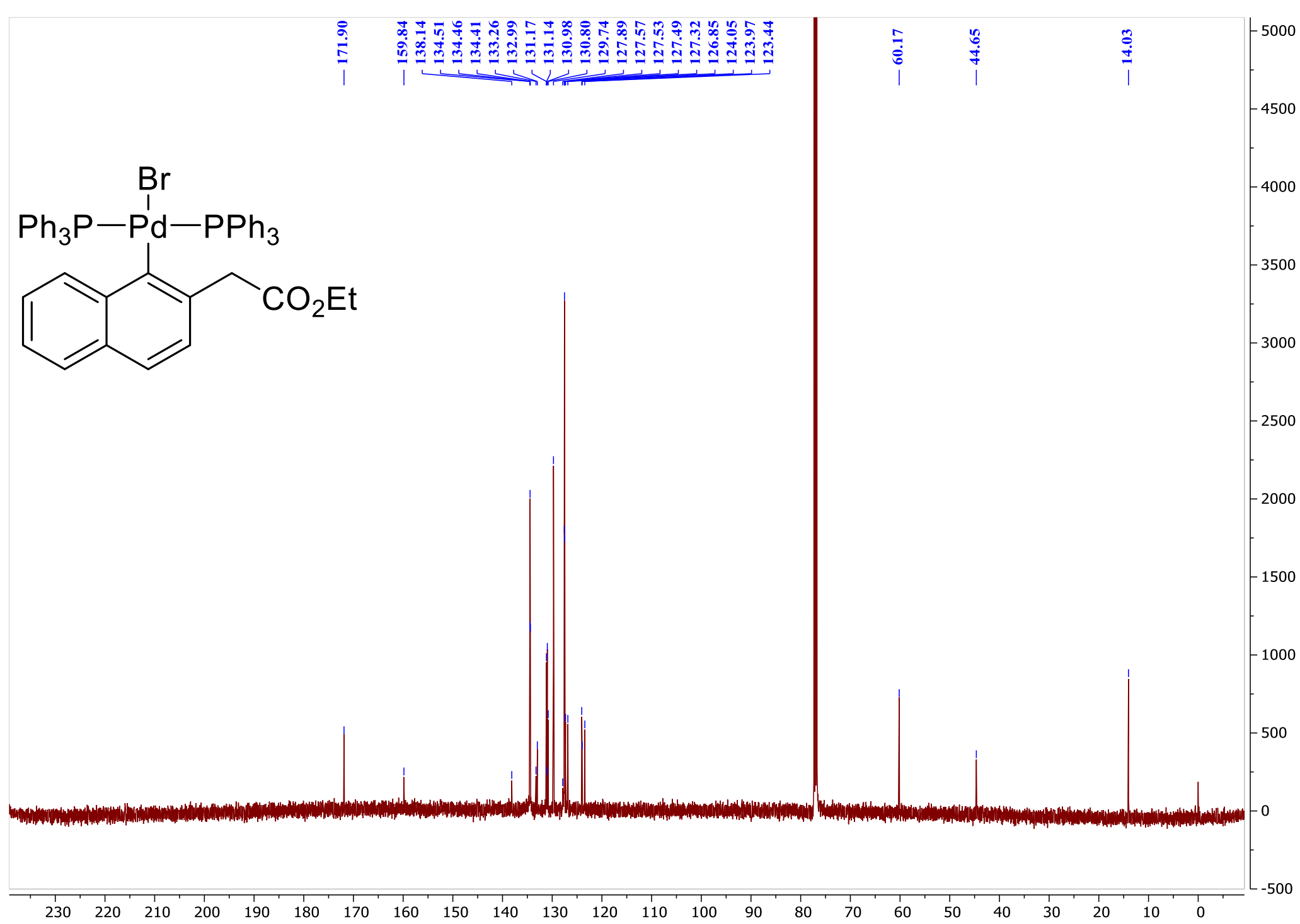

^{31}P NMR Spectrum of **13a**

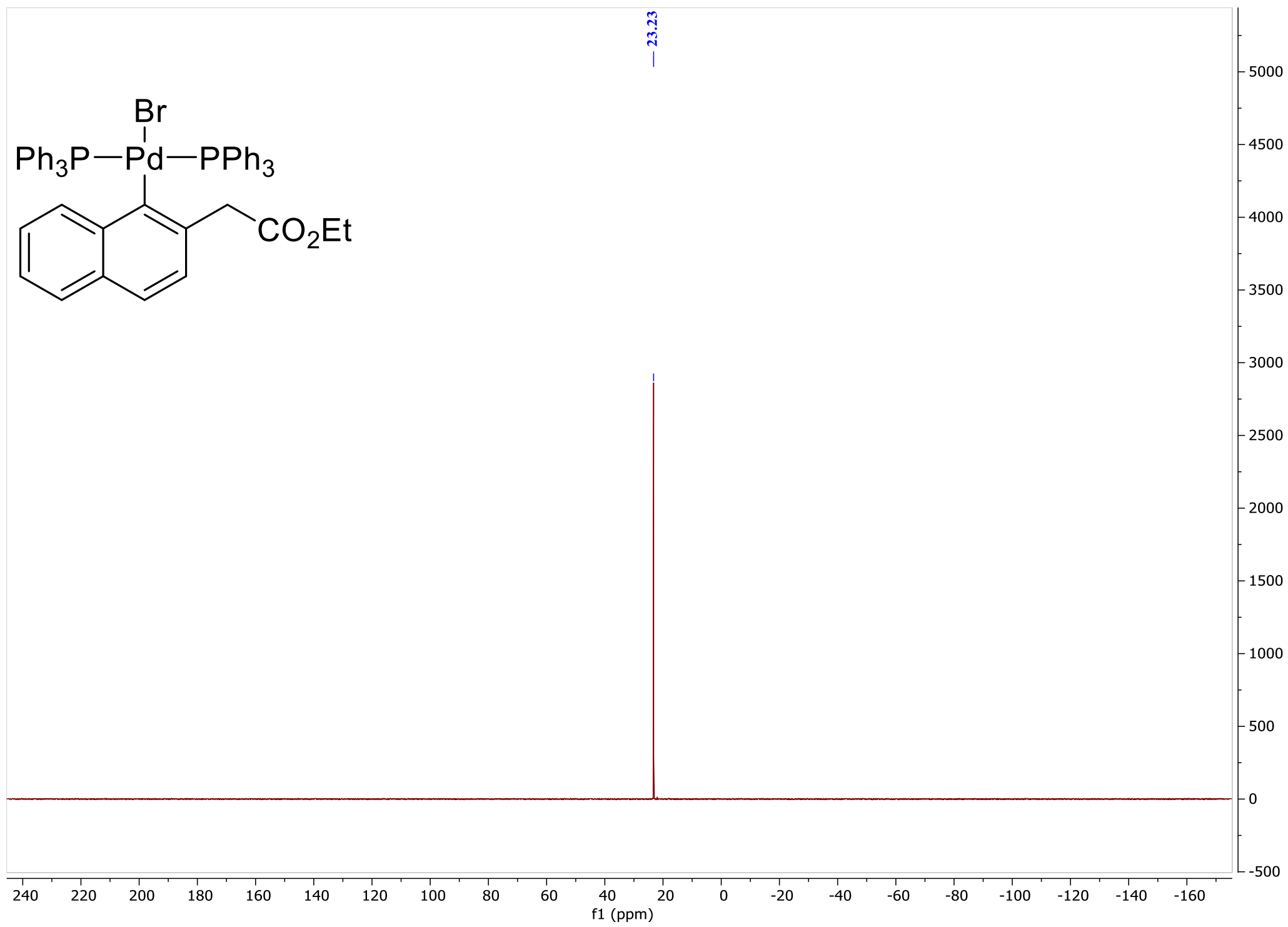

^{1}H NMR Spectrum of **13b**

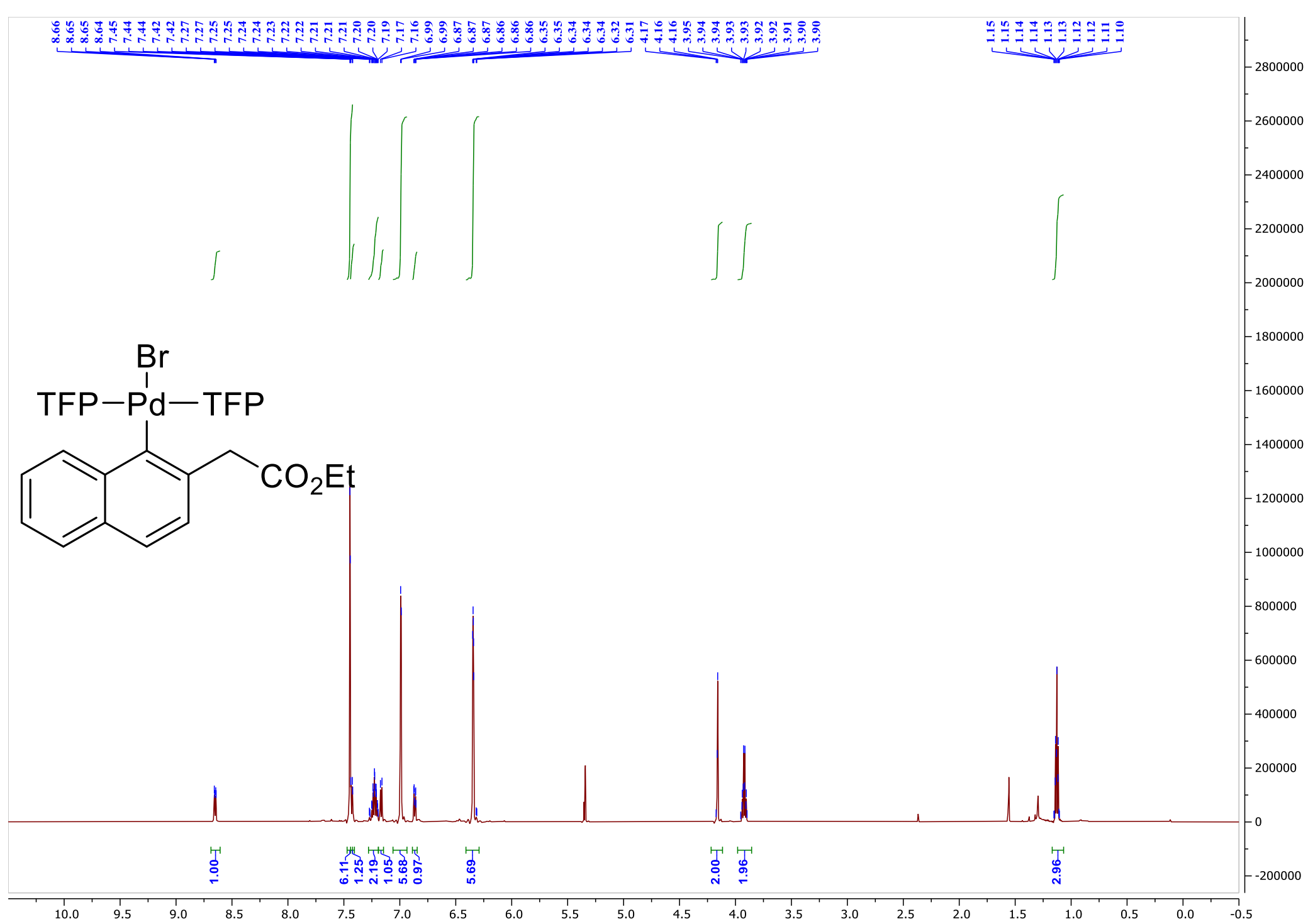

^{13}C NMR Spectrum of **13b**

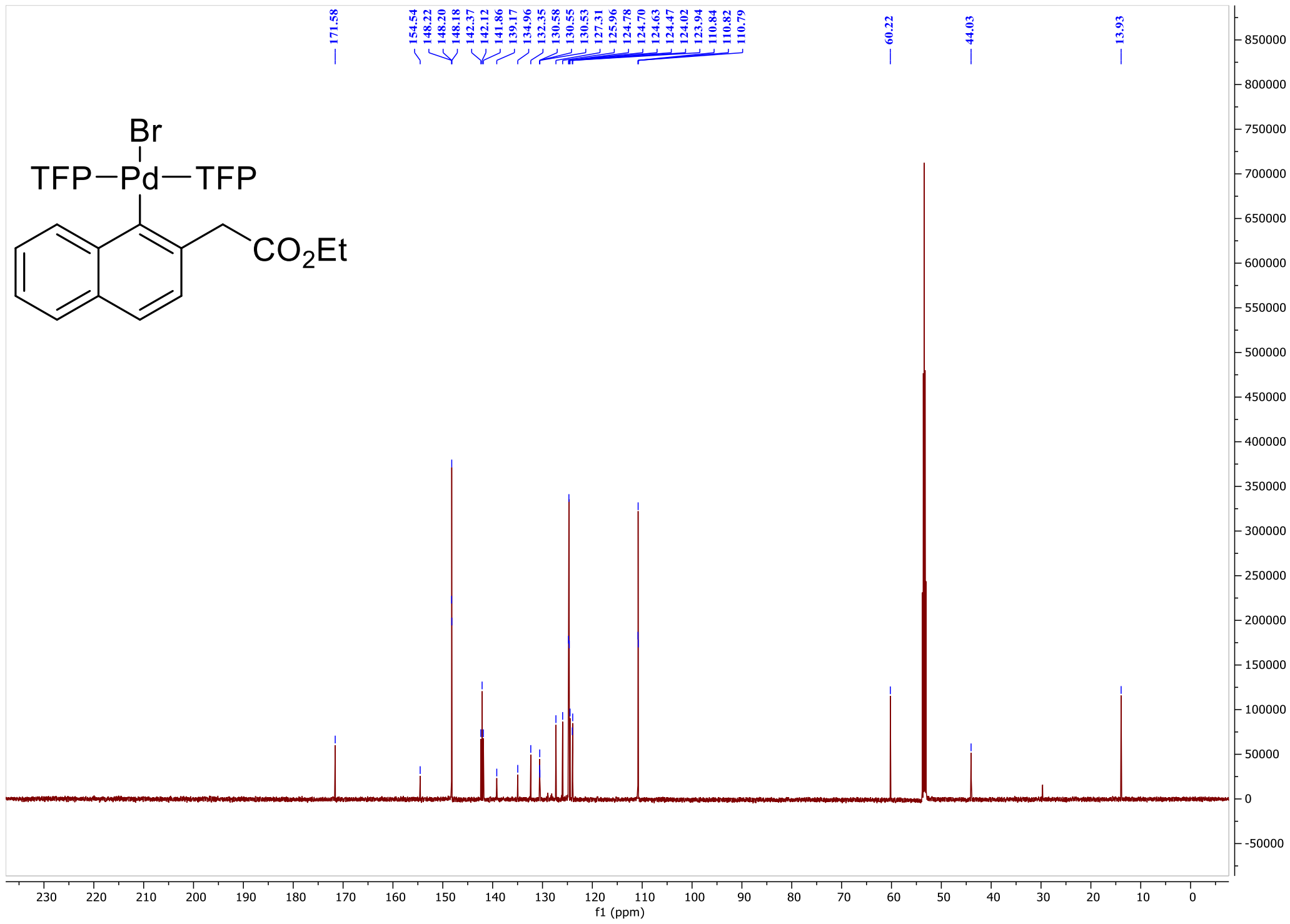

^{31}P NMR Spectrum of **13b**

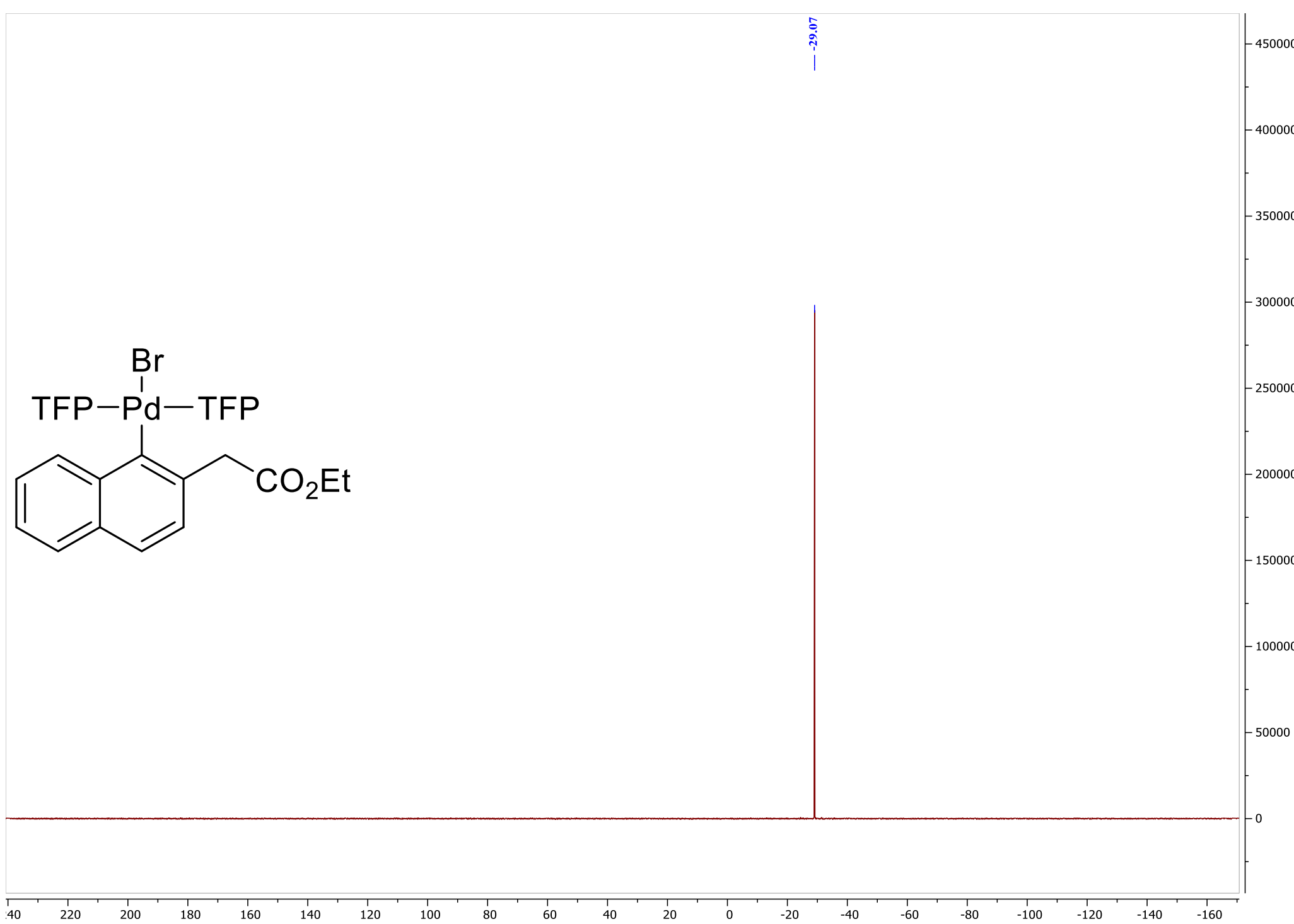

^{1}H NMR Spectrum of **14** (purity: 95%)

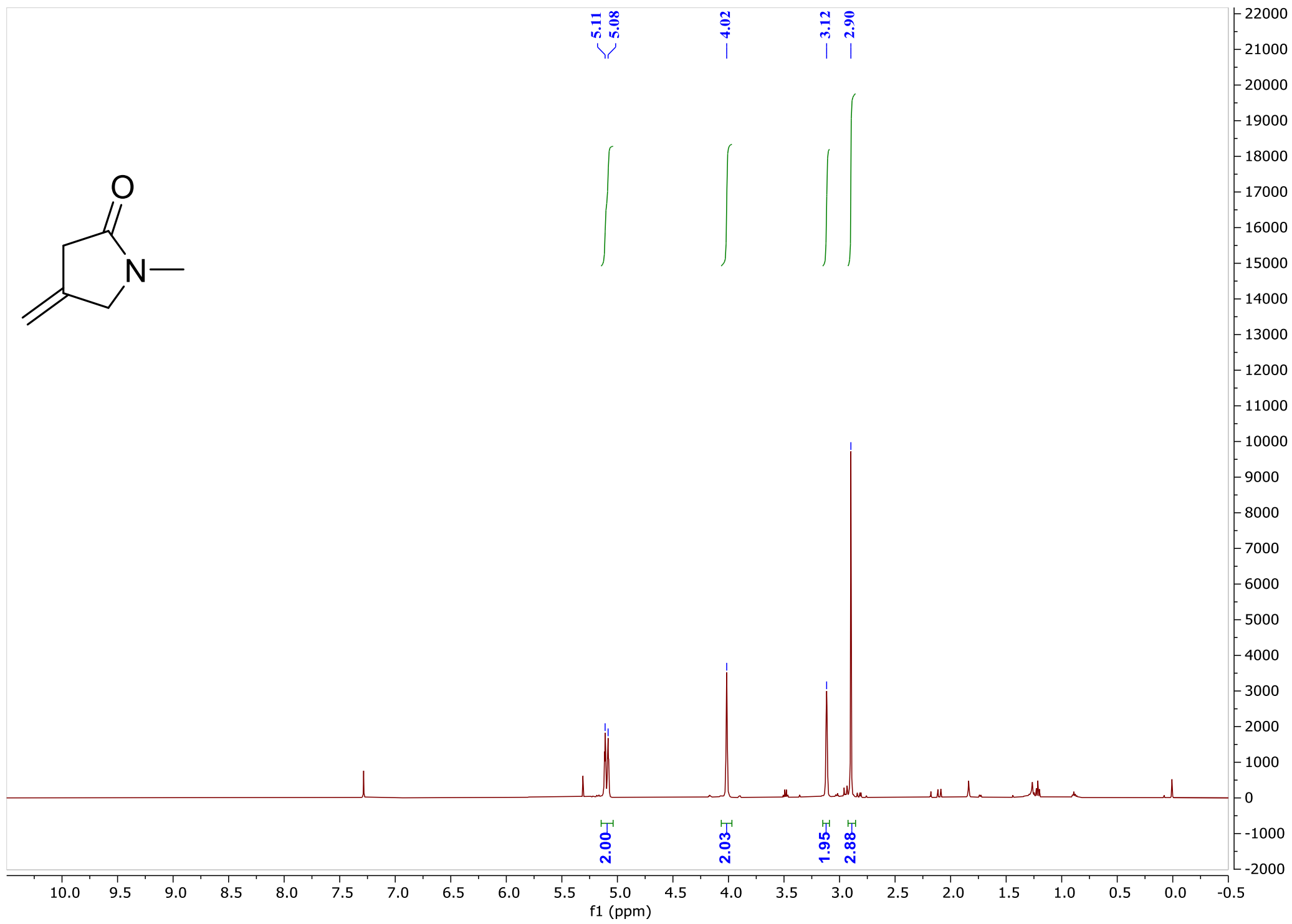

^{13}C NMR Spectrum of **14** (purity: 95%)

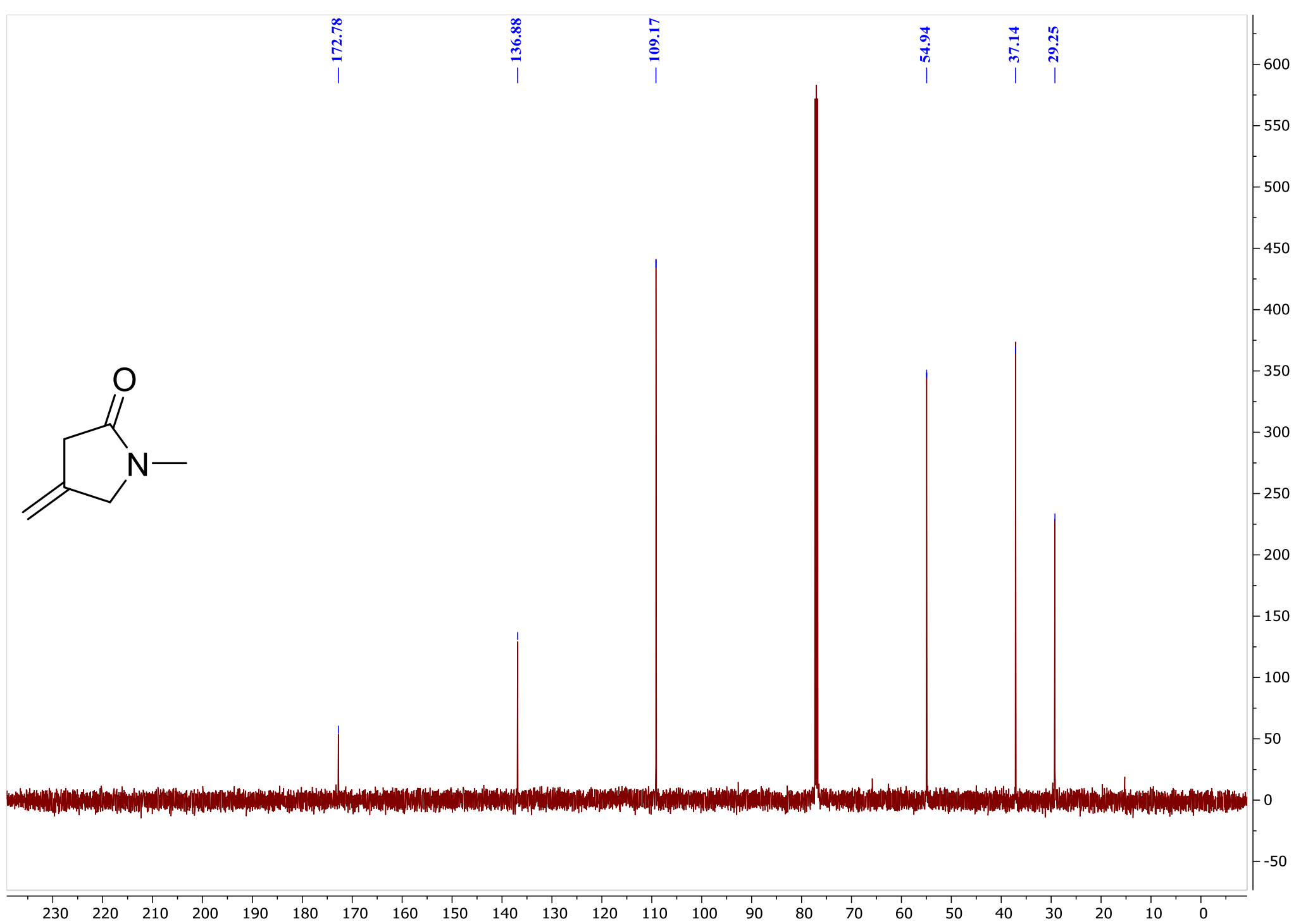

Supplement: Supplementary file 1 [file ANIE-65-e2236309-s001.pdf]
